# DFT investigation of hydroxyl radical scavenging mechanisms in bioactive terpenoids from *Syzygium nervosum*

**DOI:** 10.1038/s41598-026-56769-y

**Published:** 2026-06-06

**Authors:** Hieu Linh Duong, Duy Hien Tong, Dinh Phi Le, Thi Lien Thuong Nguyen, Trong Hong Phuc Nguyen, Minh Chanh Nguyen, Dang Khoa Nguyen, Thuat T. Trinh

**Affiliations:** 1 Faculty of Engineering, Vietnamese-German University (VGU), Ring Road 4, Quarter 4, Thoi Hoa Ward, Ho Chi Minh City, Vietnam; 2 Institute of Innovation in Pharmaceutical and Healthcare Food, Thu Dau Mot University, Ho Chi Minh City, Vietnam; 3 Can Tho University of Medicine and Pharmacy (CTUMP), Can Tho City, Vietnam; 4 Institute of Applied Science and Technology, Van Lang School of Technology, Van Lang University, Ho Chi Minh City, 70000 Vietnam; 5 Faculty of Applied Technology, Van Lang School of Technology, Van Lang University, Ho Chi Minh City, 70000 Vietnam; 6 Porelab, Department of Chemistry and Biomedical Science, Norwegian University of Science and Technology, NTNU, Trondheim, NO-7491 Norway

**Keywords:** Antioxidants, Terpenoids, DFT, Radical scavenging, Syzygium nervosum, Biochemistry, Chemical biology, Chemistry, Drug discovery

## Abstract

Reactive oxygen species, particularly the hydroxyl radical (${\hbox {OH}^{\bullet }}$), drive oxidative stress and cellular damage. This study employs density functional theory (M06-2X/def2-TZVP//M06-2X/ma-def2-SVP with SMD solvation) to identify the most promising antioxidant compounds from thirteen terpenoids isolated from *Syzygium nervosum*. Radical adduct formation (RAF) emerges as the dominant scavenging mechanism with low kinetic barriers (14–49 kJ/mol), indicating room-temperature reactivity. Dehydroisolongifolene exhibits the lowest RAF barrier (14 kJ/mol) and exceptional multi-mechanism performance, making it the top candidate for experimental validation. (+)-Carotol shows balanced activity across multiple mechanisms, ideal for broad-spectrum applications, while (-)-myrtenol demonstrates the best RAF performance among monoterpenoids. Sesquiterpenoids outperform monoterpenoids in electron transfer due to enhanced *π*-conjugation, whereas monoterpenoids excel in radical addition kinetics. Oxygen functionalization, conjugation extent, and site accessibility are key determinants of antioxidant capacity. These computational predictions provide a rational basis for prioritizing dehydroisolongifolene, (+)-carotol, and (-)-myrtenol in bioassay-guided fractionation, accelerating the discovery of novel antioxidants for burn wound healing, neuroprotection, and oxidative stress management.

**Supplementary Information:**

The online version contains supplementary material available at 10.1038/s41598-026-56769-y.

## Introduction

*Syzygium nervosum* is a Southeast Asian medicinal plant traditionally used to treat influenza, skin diseases, burn wounds, and digestive conditions^1^. Phytochemical studies have identified a diverse array of bioactive terpenoids, phenolics, and anthocyanins in this species, with berry extracts exhibiting high total phenolic and anthocyanin content^1,2^. Terpenoids, particularly sesquiterpenoids with their larger carbon frameworks (C15) and enhanced *π*-electron delocalization, represent a major class of antioxidant secondary metabolites^3,4^. Related Myrtaceae species show strong radical scavenging, neuroprotective, and antiaging activities, underscoring the importance of understanding the molecular mechanisms underlying antioxidant action^5–8^. The structural determinants of terpenoid antioxidant activity include oxygen functionalization (hydroxyl, carbonyl, epoxy), conjugation, and molecular flexibility; oxygenation, particularly allylic hydroxyl groups, enhances radical scavenging by providing labile hydrogen atoms and stabilizing radical intermediates through resonance^4,6,9^.

Reactive oxygen species, including hydroxyl radicals (OH *·*), superoxide anion ($\hbox {O}_2\,\cdot ^-$), and hydrogen peroxide ($\hbox {H}_2\hbox {O}_2$), are byproducts of normal cellular metabolism^10^. While low concentrations play essential roles in cell signaling and immune function, excessive ROS accumulation leads to oxidative stress, which is implicated in aging, neurodegenerative diseases, cardiovascular disorders, and cancer^11^. The hydroxyl radical is particularly reactive and damaging, with a standard reduction potential of 2.8 V and the ability to attack biological macromolecules including DNA, proteins, and lipids through hydrogen abstraction and addition reactions^12^. Hydroxyl radicals can be generated through various mechanisms, including Fenton reactions involving redox-active metals and radiation-induced water radiolysis^12^. Natural antioxidant compounds from plants can neutralize these deleterious radicals, thereby protecting cells and tissues from oxidative damage through multiple mechanisms including hydrogen atom transfer, electron transfer, and radical adduct formation^13^. Recent computational studies have provided detailed insights into the kinetics and mechanisms of hydroxyl radical reactions with organic compounds^14^.

The hydroxyl radical can be neutralized through several distinct pathways including radical adduct formation (RAF), hydrogen atom transfer (HAT), single electron transfer (SET), and sequential proton loss electron transfer (SPLET). Each pathway possesses characteristic thermodynamic and kinetic signatures that depend on molecular structure and environment; a detailed description of the four mechanisms is provided in Section 2.3.

Comprehensive reviews have established that density functional theory (DFT) calculations can reliably predict antioxidant properties through analysis of bond dissociation enthalpies, ionization potentials, proton affinities, and reaction mechanisms^15^. Modern DFT methods, particularly those employing hybrid functionals such as B3LYP, or M06-2X with appropriate basis sets, have demonstrated excellent agreement with experimental data for a wide range of antioxidant compounds^16,17^. These theoretical tools enable detailed understanding of chemical behavior at the molecular level, including the effects of solvent, substituents, and molecular structure on antioxidant activity^15^. DFT calculations offer significant advantages over purely experimental approaches, including the ability to screen large numbers of compounds, predict activity before synthesis, and provide mechanistic insights that are difficult to obtain experimentally^15^. Multi-scale simulation approaches combining DFT with molecular dynamics have proven effective for investigating bioactive compounds from medicinal plants^18^. First-principles calculations have been successfully applied to study thermodynamic properties and reaction mechanisms in complex chemical systems^19,20^. DFT-based methods combined with kinetic modeling provide powerful tools for elucidating reaction pathways and selective formation mechanisms^21^. Although experimental studies have demonstrated the antioxidant capacity of *S. nervosum* extracts^1,2^, no computational study has yet determined which radical scavenging mechanism dominates for the individual terpenoids, how their molecular structures control activity, or whether structure–activity rules derived from phenolic antioxidants extend to terpenoid scaffolds. This gap motivates the present investigation.

This study employs DFT calculations with in-house Python-based analysis to systematically investigate the radical scavenging activity of thirteen bioactive terpenoids isolated from *S. nervosum*. We test the hypothesis that sesquiterpenoids with extended *π*-conjugation will preferentially scavenge via SET, whereas monoterpenoids bearing allylic hydroxyl groups will favor HAT, and electron-rich double bonds will promote RAF. By analyzing RAF, SET, and SPLET mechanisms for each compound, we rank their thermodynamic favorability and identify structural features that enhance radical scavenging capacity. The computational approach enables efficient screening of multiple compounds and provides detailed mechanistic insights that complement experimental findings. The results provide molecular-level insights that guide future development of plant-based antioxidants for burn wound healing, neuroprotective, antiaging, and stress resistance applications.

## Methods

### Computational methodology

All quantum chemical calculations were performed using density functional theory (DFT) as implemented in the ORCA quantum chemistry package (version 6.0)^22,23^. The M06-2X hybrid meta-GGA functional was employed throughout this study^24^, as this functional has been extensively validated for thermochemical and kinetic parameters of radical reactions^15,25^.

A dual-level computational strategy was adopted to balance accuracy and efficiency^15^. Conformer searches were performed using ORCA’s built-in conformer search capabilities to identify the lowest energy conformers for all molecular systems. Geometry optimizations and frequency calculations were performed at the M06-2X/ma-def2-SVP level using the RIJCOSX approximation with def2/J auxiliary basis sets. Final electronic energies were computed at the M06-2X/def2-TZVP level on the optimized geometries. This composite approach is denoted as M06-2X/def2-TZVP//M06-2X/ma-def2-SVP. Gibbs free energies were calculated as $\Delta G = G_{\text {products}} - G_{\text {reactants}}$ at 298.15 K and 1 atm, including zero-point energy and thermal corrections.

A Python-based automation workflow was developed to manage the multi-level calculations, integrating ORCA with PBS job orchestration. The workflow handles geometry optimization, transition state searches using the Nudged Elastic Band (NEB) method, frequency calculations, and energy extraction with SQLite database management. For radical addition and hydrogen abstraction pathways, initial encounter geometries were generated via constrained optimization with fixed intermolecular distances (C*⋯*O *≈* 2.5–3.0 Å for RAF; O*⋯*H *≈* 2.0–2.5 Å for HAT). NEB calculations employed 10 images spanning the reaction coordinate; the highest-energy image was optimized to a first-order saddle point and confirmed by frequency analysis to possess exactly one imaginary frequency (see Supporting Information). In total, over 300 independent DFT calculations were performed for the full dataset, systematically covering all four scavenging mechanisms (RAF, SET, HAT, SPLET) in the gas phase as well as in water and chlorobenzene (as a lipid-membrane proxy).

SMD implicit solvation calculations^26^ were performed to assess environmental effects. Water (*\varepsilon* = 78.36) represents cellular/physiological conditions, while chlorobenzene (*\varepsilon* = 5.62) serves as a proxy for lipid membranes. Solvation free energies were calculated at the M06-2X/def2-TZVP level on gas-phase optimized geometries as $\Delta G_{\text {solv}} = G_{\text {solv}} - G_{\text {gas}}$.

All calculations involving open-shell species used the unrestricted Kohn–Sham (UKS) formalism. The hydroxyl radical ($\hbox {OH}^{\bullet }$) was treated as a doublet (charge 0, multiplicity 2, 1 unpaired electron). All neutral terpenoids are closed-shell singlets (charge 0, multiplicity 1); SET radical cations are doublets (*+1*, 2); HAT and RAF products are doublets (0, 2); SPLET anions are singlets (*-1*, 1). Wavefunctions converged to $|\text {gradient}| < 10^{-5}$ Ha/$a_0$ and $S^2$ expectation values for all doublet species lie within 0.749–0.783 (ideal 0.7500), confirming minimal spin contamination. Full charge, spin, and convergence data are provided in the Supporting Information. Because M06-2X includes medium-range dispersion through its parametrization, and single-point D3BJ estimates (provided in the Supporting Information) give negligible barrier corrections (*<1* kJ mol$^{-1}$), no empirical dispersion correction was applied to the reported barriers.

### Molecular systems and radical scavenging mechanisms

**Fig. 1:**
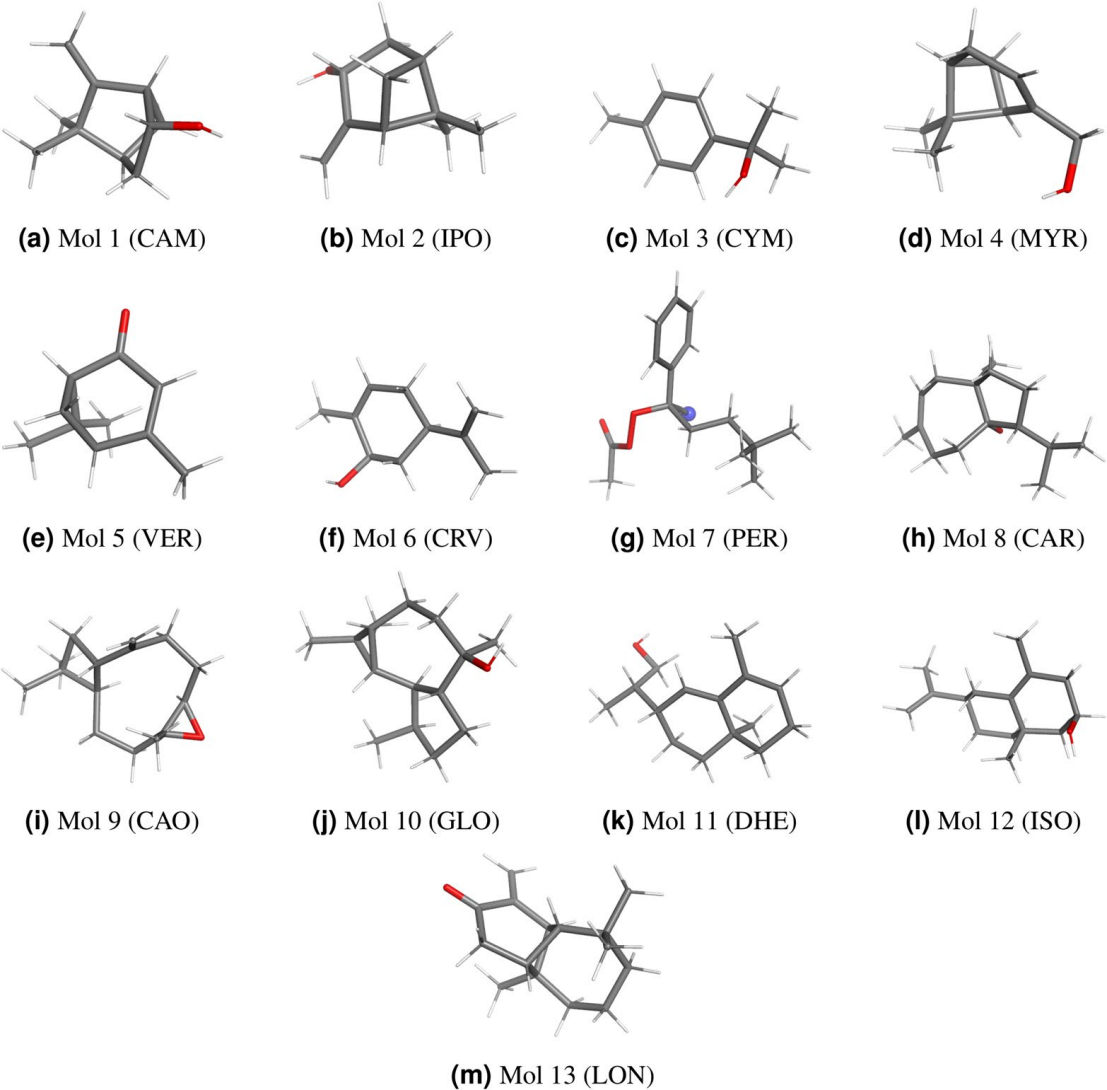
Optimized structures of 13 terpenoids from *Syzygium nervosum* (C: gray, O: red, H: white). (**a**) Camphenol, (**b**) Isopinocarveol, (**c**) *p*-Cymen-8-ol, (**d**) (-)-Myrtenol, (**e**) *l*-Verbenone, (**f**) *cis*-Carveol [Monoterpenoids, $\hbox {C}_10$]; (g) Ethaneperoxoic acid [Peroxyester, $\hbox {C}_16$]; (h) (+)-Carotol, (i) Caryophyllene oxide, (j) (-)-Globulol, (k) DHE, (l) ISO, (m) Longipinocarvone [Sesquiterpenoids, $\hbox {C}_15$].

Thirteen bioactive terpenoid compounds from *Syzygium nervosum* were selected for investigation: six monoterpenoids (C$_{10}$; Molecules 1–6), six sesquiterpenoids (C$_{15}$; Molecules 8–13), and one peroxyester (C$_{16}$; Molecule 7). All calculations employed the hydroxyl radical (OH$^{\bullet }$) as the target reactive oxygen species. Their optimized molecular structures are shown in Figure 1.

**Fig. 2:**
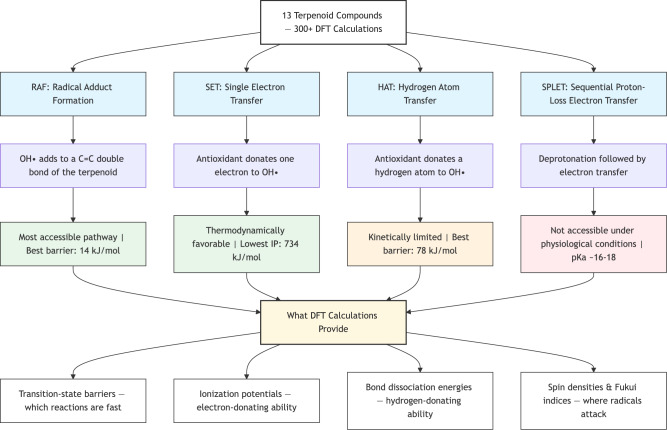
Computational workflow and four radical-scavenging mechanisms investigated in this study. DFT calculations provide transition-state barriers (kinetic accessibility), ionization potentials (electron-donating ability), bond dissociation energies (hydrogen-donating ability), and spin/Fukui analysis (radical attack sites).

To evaluate the antioxidant potential of these compounds, four distinct mechanisms were investigated for hydroxyl radical scavenging, as illustrated in Figure 2.

The first of these is radical adduct formation (RAF), a concerted process in which the hydroxyl radical adds directly across an activated C=C *π*-bond of the terpenoid, thereby forming a stable carbon-centered radical adduct in a single step:

1 $$\begin{aligned} {\hbox {ArH} + \hbox {OH}^{\bullet } \longrightarrow \hbox {Ar(OH)}^{\bullet }} \end{aligned}$$

where ArH represents the terpenoid and Ar(OH)$^{\bullet }$ denotes the radical adduct. It is important to emphasize that this mechanism does not involve C–H bond cleavage or sequential H-abstraction followed by hydroxylation; rather, the OH radical itself acts as the oxidant, adding to the *π*-system and converting one *π*-bond into a new C–O *σ*-bond with the unpaired electron localizing on the adjacent carbon. This concerted addition pathway is well established in computational antioxidant chemistry^15,16,25^. Transition states were located using the NEB method followed by TS optimization. Gibbs free energies were calculated as $\Delta G = G_{\text {adduct}} - (G_{\text {ArH}} + G_{{\hbox {OH}^{\bullet }}})$. Eleven RAF reactions were characterized (Molecules 1–6, 8, 9, 11–13).

A second pathway is single electron transfer (SET), in which the antioxidant donates an electron to the hydroxyl radical to generate a radical cation:

2 $$\begin{aligned} {\hbox {ArH} + \hbox {OH}^{\bullet } \longrightarrow \hbox {ArH}^{+\bullet } + \hbox {OH}^{-}} \end{aligned}$$

Ionization potentials were calculated as IP = $E_{\text {cation}} - E_{\text {neutral}}$ from optimized geometries of neutral and radical cation species. Lower IP values indicate greater electron donation capacity. Complete SET data were obtained for all thirteen compounds.

As a third possibility, sequential proton loss electron transfer (SPLET) proceeds via initial deprotonation followed by electron transfer:

3 $$\begin{aligned} \hbox {ArH} & \longrightarrow \hbox {Ar}^{-} + \hbox {H}^{+} \end{aligned}$$

4 $$\begin{aligned} \hbox {Ar}^{-} + \hbox {OH}^{\bullet } & \longrightarrow \hbox {Ar}^{\bullet } + \hbox {OH}^{-} \end{aligned}$$

Deprotonation energies were calculated as $\Delta E_{\text {deprot}} = E_{\text {anion}} - E_{\text {neutral}}$ for all acidic hydrogen sites. The most favorable deprotonation site was used for compound ranking. Complete SPLET data were obtained for all thirteen compounds.

Finally, hydrogen atom transfer (HAT) involves direct transfer of a hydrogen atom from the terpenoid to the hydroxyl radical:

5 $$\begin{aligned} {\hbox {ArH} + \hbox {OH}^{\bullet } \longrightarrow \hbox {Ar}^{\bullet } + \hbox {H2O}} \end{aligned}$$

Transition states for hydrogen abstraction from O–H bonds were located using NEB calculations. The reaction coordinate involves pre-reaction complex (PRC) formation, hydrogen transfer via TS, and post-reaction complex (PostRC) dissociation to products. Gibbs free energies were calculated as $\Delta G = [G(\text {R}^{\bullet }) + G({\hbox {H}_2\hbox {O}})] - [G(\text {R-H}) + G({\hbox {OH}^{\bullet }})]$. HAT data were obtained for Molecules 1, 2, 4, and 12; other molecules lacked convergent O–H transition states due to structural constraints^27^.

## Results and discussion

The thirteen bioactive terpenoids investigated in this study, together with their three-letter abbreviations and chemical formulas, are summarized in Table 1.

**Table 1:** Reference table of 13 bioactive compounds from *Syzygium nervosum* with three-letter abbreviations and chemical formulas.

ID	Abbrev.	Compound name	Formula
1	CAM	Camphenol	${\hbox {C}_{10}\hbox {H}_{16}\hbox {O}}$
2	IPO	Isopinocarveol	${\hbox {C}_{10}\hbox {H}_{16}\hbox {O}}$
3	CYM	*p*-Cymen-8-ol	${\hbox {C}_{10}\hbox {H}_{14}\hbox {O}}$
4	MYR	(-)-Myrtenol	${\hbox {C}_{10}\hbox {H}_{16}\hbox {O}}$
5	VER	*l*-Verbenone	${\hbox {C}_{10}\hbox {H}_{14}\hbox {O}}$
6	CRV	*cis*-Carveol	${\hbox {C}_{10}\hbox {H}_{16}\hbox {O}}$
7	PER	Ethaneperoxoic acid (peroxyester)	${\hbox {C}_{16}\hbox {H}_{21}\hbox {NO}_3}$
8	CAR	(+)-Carotol	${\hbox {C}_{15}\hbox {H}_{26}\hbox {O}}$
9	CAO	Caryophyllene oxide	${\hbox {C}_{15}\hbox {H}_{24}\hbox {O}}$
10	GLO	(-)-Globulol	${\hbox {C}_{15}\hbox {H}_{26}\hbox {O}}$
11	DHE	Dimethylhexahydronaphthalenylpropanol	${\hbox {C}_{15}\hbox {H}_{24}\hbox {O}}$
12	ISO	Isopropenyloctahydronaphthalenol	${\hbox {C}_{15}\hbox {H}_{24}\hbox {O}}$
13	LON	Longipinocarvone	${\hbox {C}_{15}\hbox {H}_{22}\hbox {O}}$

### Overview of computational results

The computational workflow processed thirteen bioactive terpenoids, generating comprehensive thermodynamic and kinetic data across four radical scavenging mechanisms (Figure 2). This extensive dataset enables systematic comparison of antioxidant pathways and identification of structure-activity relationships.

Data validation procedures ensured thermodynamic consistency across all calculations. Quality assurance protocols verified reactant energy accounting, enabling reliable comparison of reaction energetics across compounds and mechanisms. The corrected dataset provides a robust foundation for mechanistic analysis, with RAF barriers ranging from 14–450 kJ/mol depending on molecular structure and reaction site.

The following sections present detailed analysis of each scavenging mechanism, progressing from radical adduct formation (RAF) and electron transfer (SET), the two dominant pathways, through hydrogen atom transfer (HAT) and sequential proton loss electron transfer (SPLET).

### Radical adduct formation (RAF) mechanism

Among the thirteen terpenoids, RAF reactions were successfully characterized for eleven molecules (Molecules 1, 2, 3, 4, 5, 6, 8, 9, 11, 12, 13) across 11 sites with converged transition states (Table 2). Kinetic data obtained from ORCA NEB calculations reveals barriers ranging from 14 to 49 kJ/mol, corresponding to accessible radical adduct formation under ambient conditions. All characterized RAF pathways show positive activation barriers with Gibbs free energies, demonstrating kinetic accessibility. As detailed in Section 2.3, these reactions proceed via concerted $\hbox {OH}^{\bullet }$ addition to a C=C *π*-bond without C–H bond cleavage, consistent with established computational frameworks for radical scavenging in terpenoids and phenolic compounds^15,16^.

**Table 2:** RAF mechanism: Gibbs free energy barriers ($\Delta G^{\ddagger }$, water, M06-2X/def2-TZVP+SMD) and product adduct stability ($\Delta G_{\text {prod}}$, water, M06-2X/def2-TZVP+SMD//M06-2X/ma-def2-SVP) for 11 bioactive sites ranked by kinetic favorability. Barriers are measured from separated reactants; all are accessible at room temperature (14–49 kJ mol$^{-1}$). See Table 1 for abbreviations.

ID	Abbrev.	Site	∆*G*‡	∆*G*prod
			(kJ/mol)	(kJ/mol)
11	DHE	12	14	−108
11	DHE	7	18	−94
1	CAM	10	27	−84
2	IPO	10	31	−81
6	CRV	10	31	−79
8	CAR	11	35	−76
4	MYR	9	36	−86
12	ISO	12	38	−85
4	MYR	6	39	−97
2	IPO	6	41	−69
3	CYM	6	49	−30

The kinetically most accessible RAF pathway is Molecule 11 (Sesquiterpenoid, Dehydroisolongifolene, site 12) with a barrier of 14 kJ/mol, representing the most favorable kinetic pathway. Molecule 11 also shows excellent kinetic accessibility at site 7 with a barrier of 18 kJ/mol. Among monoterpenoids, Molecule 1 (Camphenol, CAM) demonstrates good kinetic accessibility at site 10 with a barrier of 27 kJ/mol. Molecule 8 (Sesquiterpenoid, (+)-Carotol, CAR) exhibits a moderate kinetic barrier of 35 kJ/mol at site 11. Molecule 4 ((-)-Myrtenol, MYR) shows kinetic accessibility at both sites 9 and 6 with barriers of 36 and 39 kJ/mol, respectively. The barriers range from very low (14–18 kJ/mol) to moderate (up to 49 kJ/mol), all corresponding to room-temperature reactivity.

Having established the thermodynamic landscape of RAF reactions, we now examine the structural factors that influence reactivity patterns. RAF reactivity reveals clear kinetic accessibility trends across the compound set. The kinetic frontier is dominated by low barriers (< 40 kJ/mol) across both monoterpenoid and sesquiterpenoid classes. Molecule 11 (Sesquiterpenoid, Dehydroisolongifolene, DHE) exhibits the lowest barriers at sites 12 and 7 (14 and 18 kJ/mol), indicating facile radical addition at room temperature. Molecules 1, 2, 6, and 8 show barriers in the 27–35 kJ/mol range, while Molecule 4 shows barriers of 36 and 39 kJ/mol, representing good kinetic accessibility. The RAF mechanism emerges as a viable pathway for hydroxyl radical scavenging, with accessible kinetic barriers (14–49 kJ/mol range) corresponding to room-temperature reactivity for all characterized pathways.

**Fig. 3:**
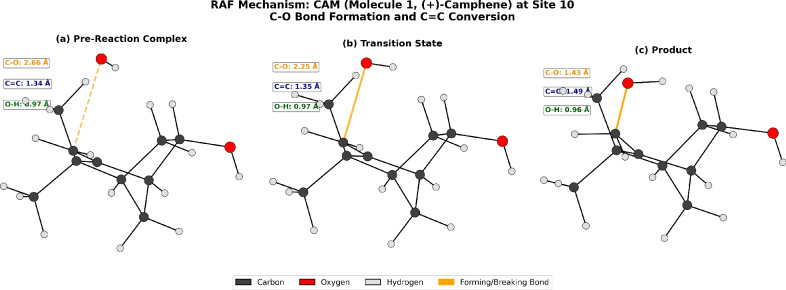
RAF mechanism structural evolution for CAM at site 10. (**a**) Pre-reaction complex (PRC): OH radical approaches with C*⋯*O = 2.66 Å. (**b**) Transition state (TS): Forming C–O bond = 2.25 Å. (**c**) Product: C–O bond = 1.43 Å.

To gain deeper insight into the molecular-level changes during radical adduct formation, we analyzed the geometric evolution along the RAF reaction pathway for Molecule 1 (Camphenol, CAM) at site 10, which exhibits a barrier of 27 kJ/mol. Figure 3 displays the three key structures along the reaction coordinate: the pre-reaction complex (PRC), the transition state (TS), and the product.

The geometric analysis reveals the structural changes accompanying the RAF reaction. In the pre-reaction complex, the OH radical is positioned at a C*⋯*O distance of 2.66 Å from the exocyclic carbon (C11), while maintaining the C=C double bond character at 1.34 Å. The approaching OH radical adopts an orientation that positions the oxygen toward the *π*-system of the double bond, preparing for the addition reaction.

At the transition state, the C–O bond is partially formed with a distance of 2.25 Å, representing a significant contraction from the PRC geometry. Simultaneously, the C=C bond begins to elongate to 1.35 Å, indicating the onset of double bond cleavage. The O–H bond length remains relatively constant at approximately 0.97 Å throughout the reaction, as expected for a radical addition mechanism where the OH moiety remains intact.

In the product structure, the C–O bond is fully formed at 1.43 Å, consistent with a typical C–O single bond length. The former C=C double bond has expanded to 1.49 Å, characteristic of a C–C single bond, confirming the conversion of the double bond to a single bond upon adduct formation. The C–C–O bond angle increases from 87.8$^\circ$ in the PRC to 113.9$^\circ$ in the product, reflecting the rehybridization of the carbon center from sp$^2$ to sp$^3$ geometry.

These structural changes demonstrate the concerted nature of the RAF mechanism: as the OH radical approaches and forms a bond with the carbon center, the *π*-bond of the double bond is broken and converted to a *σ*-bond, with the unpaired electron delocalized onto the adjacent carbon. The moderate barrier of 27 kJ/mol reflects the energy required to simultaneously break the C=C *π*-bond and form the new C–O bond, consistent with the observed room-temperature reactivity of this pathway. This mechanistic understanding provides a foundation for evaluating how environmental factors influence RAF reactivity.

To evaluate the influence of biological environment on RAF reactivity, we conducted SMD solvation calculations in water and lipid media at the M06-2X/def2-TZVP level of theory. The solvation analysis reveals a pronounced solvent preference: all characterized RAF sites exhibit lower activation barriers in aqueous environment compared to lipid media.

The solvent effect ($\Delta \Delta G^\ddagger = \Delta G^\ddagger _{\text {lipid}} - \Delta G^\ddagger _{\text {water}}$) ranges from –8 to +19 kJ/mol, with an average penalty of +5 kJ/mol in lipid media. This indicates that RAF reactions are substantially more favorable in aqueous environments. The most pronounced solvent effects are observed for DHE site 11 ($\Delta \Delta G^\ddagger = +30$ kJ/mol), CAR site 11 (+25 kJ/mol), and CYM site 7 (+21 kJ/mol).

All characterized RAF sites exhibit positive activation barriers in both water and lipid environments, with barriers in water ranging from 14 kJ/mol (DHE site 12) to 87 kJ/mol (CAO site 9). The aqueous environment consistently provides lower barriers compared to lipid media, with the solvent effect ranging from +1 to +30 kJ/mol. For example, DHE site 12 shows a barrier of 14 kJ/mol in water compared to 23 kJ/mol in lipid media, representing a +9 kJ/mol penalty in the nonpolar environment. This consistent aqueous preference has important implications for biological antioxidant activity.

The strong aqueous preference for RAF can be attributed to the polar nature of the transition state, which involves charge redistribution during radical addition. Water solvation stabilizes the developing charge separation better than nonpolar lipid environments, thereby lowering the activation energy. This solvent effect has important implications for biological antioxidant activity, suggesting that RAF-based antioxidant mechanisms would be more effective in aqueous cellular compartments than in lipid membranes.

**Fig. 4:**
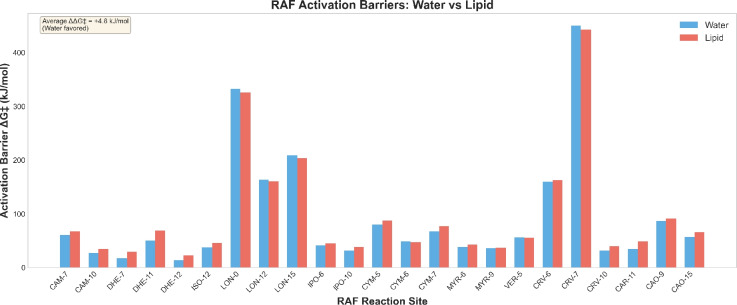
RAF activation barriers in water (blue) and lipid (red). Most compounds show lower barriers in aqueous environment (average penalty +5 kJ/mol in lipid).

The solvent effect is further illustrated in Figure 4, which displays the activation barriers for all 22 RAF sites in both solvents. The consistent gap between water (blue) and lipid (red) bars demonstrates the uniform aqueous preference across most compounds (18 of 22 sites). Figure 4 also highlights the most favorable pathways: DHE site 12 exhibits the lowest barrier in water (14 kJ/mol), while DHE site 12 also shows the lowest barrier in lipid (23 kJ/mol), making it the most kinetically favorable pathway in both environments.

Having established RAF as a kinetically accessible pathway involving radical addition to carbon centers, we now examine an alternative electron-transfer mechanism that operates through fundamentally different chemical principles.

### Single electron transfer (SET) mechanism

SET mechanism analysis yielded ionization potentials (IP) for all thirteen compounds (Molecules 1–13) from *Syzygium nervosum* (Table 3). Lower IP values indicate easier electron transfer, corresponding to superior SET antioxidant capacity.

**Table 3:** SET mechanism: ionization potentials and frontier molecular orbital energies (HOMO, LUMO) for 13 bioactive compounds. Lower IP indicates better SET activity. See Table 1 for abbreviations.

ID	Abbrev.	HOMO	LUMO	IP
	(gas)	(eV)	(eV)	(kJ/mol)
11	DHE	−7.22	0.14	734
12	ISO	−7.49	0.08	740
8	CAR	−7.99	0.17	770
10	GLO	−8.41	0.24	785
4	MYR	−7.99	0.19	786
2	IPO	−8.14	0.22	790
3	CYM	−8.07	0.26	797
6	CRV	−8.10	0.28	812
9	CAO	−8.13	0.16	814
1	CAM	−8.19	0.22	816
5	VER	−8.42	−0.45	844
13	LON	−8.58	−0.65	860
7	PER	−8.78	−0.18	895

Molecule 7 (Peroxyester) exhibits the highest ionization potential with IP = 895 kJ/mol (gas phase), indicating lower SET activity consistent with the electron-deficient peroxy functional group. Among sesquiterpenoids, Molecule 11 (DHE) shows the best SET performance with the lowest IP = 734 kJ/mol, likely due to favorable orbital alignment for electron donation. Molecule 12 (ISO) and Molecule 10 (GLO) follow closely with IP values of 740 kJ/mol and 785 kJ/mol, respectively, demonstrating the range of SET activity within oxygen-functionalized sesquiterpenoids. Molecule 8 (CAR, 770 kJ/mol), Molecule 9 (CAO, 814 kJ/mol), Molecule 4 (MYR, 786 kJ/mol), and Molecule 13 (LON, 860 kJ/mol) span a range of ionization potentials, with the oxide functionality contributing to variations in SET activity. Molecule 1 (CAM, 816 kJ/mol), Molecule 2 (IPO, 790 kJ/mol), Molecule 3 (CYM, 797 kJ/mol), Molecule 5 (VER, 844 kJ/mol), and Molecule 6 (CRV, 812 kJ/mol) show monoterpenoid IP values ranging from 790–844 kJ/mol, demonstrating the structural diversity within this class.

To contextualize the terpenoid SET activity observed in this study, we compared our results with literature reference compounds (Table 4).

**Table 4:** Comparison of SET ionization potentials between *Syzygium nervosum* terpenoids and literature reference compounds.

Study	Compound class	Solvent type	IP range	Average
			(kJ/mol)	(kJ/mol)
Present	Sesquiterpenoids	Gas phase	734 to 895	803
Study	(neutral)			
Present	Sesquiterpenoids	Water SMD	551 to 658	595
Study	(neutral/O-neutral)			
Present	Sesquiterpenoids	Lipid SMD	574 to 721	635
Study	(neutral)	(chlorobenzene proxy)		
Literature	Polyphenols	Phenolic sites	550 to 700	620
^28^	(gallic acid, etc.)			
Literature	Rubiadin	Phenolic O-H	–	–
^29^	Anthraquinone	(reaction *Δ G*)		
		HA$^-$ + HO$^\bullet$	−20.5 (favorable)	–
		H$_2$A + HO$^\bullet$	+87 (unfavorable)	–

Rubiadin values are reaction *Δ G* with radicals, not pure IPs. Water SMD values match.

literature polyphenol range (550–700 kJ/mol). Lower IP = better SET activity.

The water SMD IP values for *Syzygium nervosum* terpenoids (551–658 kJ/mol, see Table 4) align closely with the 550–700 kJ/mol range reported for polyphenolic antioxidants in solution^28^. The best SET performer in water SMD is Molecule 12 (ISO, IP = 551 kJ/mol), exhibiting IP comparable to gallic acid (550–600 kJ/mol), a well-studied reference antioxidant. In gas phase calculations (Table 3), Molecule 11 (DHE) shows the lowest IP of 734 kJ/mol. This indicates that sesquiterpenoids from *Syzygium nervosum* possess intrinsic SET antioxidant capacity comparable to established phenolic compounds, despite lacking hydroxyl-rich aromatic systems. This finding is particularly noteworthy given the structural differences between terpenoids and polyphenols.

In comparison to the anthraquinone derivative Rubiadin^29^, our terpenoids show distinctive SET characteristics. Rubiadin exhibits phenolic O-H sites with pKa values of 7.07 and 10.92, corresponding to reaction *Δ G* values of −21 kJ/mol (favorable for anion HA$^-$ + HO$^\bullet$) and +87 kJ/mol (unfavorable for neutral H$_2$A + HO$^\bullet$) with hydroxyl radicals. The Rubiadin SET activity is highly pH-dependent due to deprotonation equilibria at physiological pH (31.9% neutral, 68.1% anionic states). In contrast, the terpenoids in the present study lack phenolic hydroxyl groups, with SET activity governed primarily by aliphatic O-H and tertiary carbon sites, resulting in pH-independent IP values. This difference in site type (phenolic vs aliphatic) explains the approximately 2-fold higher deprotonation energies for our compounds (1341–1366 kJ/mol) compared to Rubiadin (585–609 kJ/mol), as discussed in the SPLET section. These contrasting behaviors highlight the importance of site type in determining antioxidant mechanism preferences.

Solvation significantly modulates SET ionization potentials. Aqueous solvation (SMD water) decreases ionization potentials compared to gas phase, facilitating electron transfer in polar environments. Water SMD calculations show IP values ranging from 551–658 kJ/mol across the nine analyzed compounds. Molecule 12 (ISO) exhibits the most favorable SET activity in water with IP = 551 kJ/mol, followed by Molecule 8 (CAR, 572 kJ/mol) and Molecule 10 (GLO, 580 kJ/mol). These molecules maintain superior SET activity across solvent environments.

Lipid media SMD calculations reveal IP values of 574–721 kJ/mol (see Table 4), consistently 20–65 kJ/mol higher than in water. This solvent penalty of +40 kJ/mol (average) matches the 30–40 kJ/mol range reported in the literature for SET reactions in non-polar versus polar solvents. The penalty originates from reduced stabilization of oxidized (cationic) species in low-dielectric environments, where dipole-solvent interactions are weaker.

Critically, the relative SET rankings remain consistent between water and lipid media. Molecules 12, 8, 4, 10, 6, 9, 3, 1, and 5 show similar relative ordering in both environments. This indicates solvent effects add a near-constant offset to all compounds, preserving relative antioxidant capacities across polar and non-polar environments. The consistency suggests that molecular structure (e.g., oxygen functionalization, *π*-electron delocalization) governs SET activity trends more strongly than dielectric environment.

SET activity shows clear structural dependencies. Molecule 7 (PER) exhibits the highest ionization potential (895 kJ/mol in gas phase, 721 kJ/mol in lipid) among all studied compounds, indicating that peroxyester functional groups are less favorable for SET donation compared to sesquiterpenoid structures. Molecular size significantly influences electron transfer capacity, with sesquiterpenoids (IP *≈* 734–895 kJ/mol in gas phase) showing different behavior than monoterpenoids. Oxygen functionalization enhances SET activity, as evidenced by Molecules 8, 9, 11, and 12 (sesquiterpenoids with oxygen-containing groups), which show superior SET activity relative to hydrocarbon structures. These findings align with DFT studies of polyphenolic antioxidants, where electron-rich systems exhibit lower ionization potentials and superior SET activity^28^.

While SET operates through electron donation from the intact molecule, hydrogen atom transfer (HAT) represents a concerted mechanism involving both electron and proton movement. We now examine this alternative pathway for compounds bearing transferable hydrogen atoms.

### Hydrogen atom transfer (HAT) mechanism

HAT characterization for Molecules 1, 2, 4, and 12 reveals accessible barriers (78–109 kJ/mol in lipid, 89–125 kJ/mol in water) and strongly exergonic thermodynamics (*Δ G* = –58 to –71 kJ/mol), demonstrating viable hydrogen atom donation pathways (Table 5). These barriers correspond to rate constants of approximately $10^{3}$–$10^{5}$ M$^{-1}$s$^{-1}$ at physiological temperature, indicating experimentally accessible reactivity. HAT transition-state convergence was achieved for only four of the nine O–H-bearing compounds. Bond dissociation enthalpy (BDE) screening confirms that all nine compounds have favorable BDEs (<300 kJ mol$^{-1}$), establishing thermodynamic viability of HAT across the full set. However, the remaining five compounds exhibited extremely shallow pre-reaction complexes or barrierless pathways, where the hydroxyl radical abstracts the hydrogen atom without a well-defined transition state; this behaviour is expected for aliphatic alcohols reacting with the highly reactive $\hbox {OH}^{\bullet }$ radical^27^. Consequently, only CAM, IPO, MYR, and ISO yielded converged transition-state geometries suitable for barrier calculations.

**Table 5:** HAT thermodynamic and kinetic data for bioactive compounds from *Syzygium nervosum*. See Table 1 for compound abbreviations.

Mol ID	Abbrev.	∆*G*‡water	^∆*G*^‡lipid	∆∆*G*^‡^	∆*G*
		(kJ/mol)	(kJ/mol)	(kJ/mol)	(kJ/mol)
1	CAM	89	78	–11	–66
12	ISO	106	81	–25	–71
4	MYR	109	132	+23	–58
2	IPO	125	94	–31	–63
*Average*		107	96	–11	

Molecule 12 (Sesquiterpenoid, Isopropenyloctahydronaphthalenol) emerges as the HAT champion with the most favorable reaction energy (*Δ G* = –71 kJ/mol) and lowest lipid barrier ($\Delta G^{\ddagger }_{\text {lipid}}$ = 81 kJ/mol), combining thermodynamic driving force with kinetic accessibility. Molecule 1 (Monoterpenoid, Camphenol) shows similarly strong performance (*Δ G* = –66 kJ/mol, $\Delta G^{\ddagger }_{\text {lipid}}$ = 78 kJ/mol). Molecules 2 and 4 exhibit higher barriers but remain thermodynamically favorable.

**Fig. 5:**
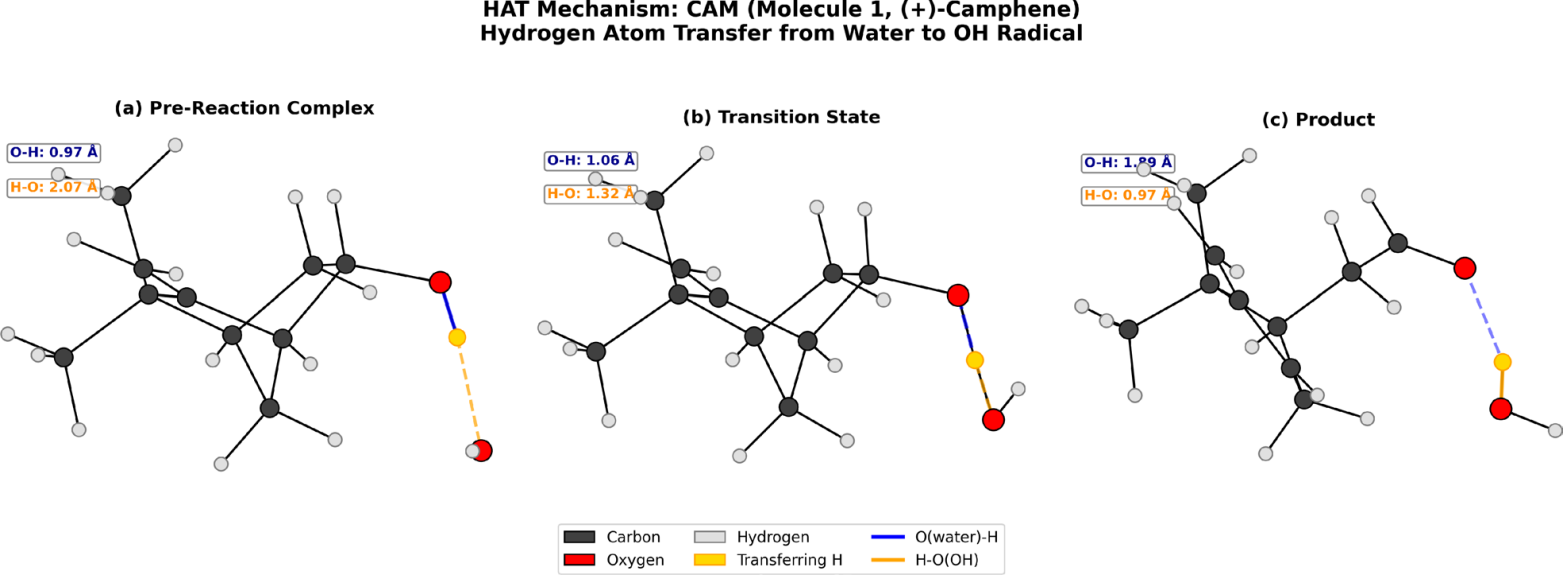
HAT mechanism structural evolution for CAM. (a) PRC: OH radical approaches with H*⋯*O = 2.07 Å. (b) TS: H atom positioned between O atoms. (c) Product: H atom transferred forming H$_{2}$O.

Geometric analysis of the HAT pathway for Molecule 1 reveals the molecular mechanism (Figure 5). In the pre-reaction complex, the OH radical approaches at an H*⋯*O distance of 2.07 Å from the transferable hydrogen. At the transition state, the O–H bond elongates to 1.06 Å while the H–O distance contracts to 1.32 Å, with the O*⋯*O distance contracting to 2.31 Å to facilitate transfer. The product shows complete hydrogen transfer forming H$_{2}$O with a normal O–H bond length of 0.97 Å. The 78 kJ/mol barrier reflects the energy required to stretch the O–H bond and position the hydrogen atom between the two oxygen centers.

The HAT results reveal a positive kinetic-thermodynamic relationship: the most thermodynamically favorable pathway (Molecule 12, *Δ G* = –71 kJ/mol) is also among the most kinetically accessible ($\Delta G^{\ddagger }_{\text {lipid}}$ = 81 kJ/mol). This correlation indicates that local hydrogen bonding environments influence both transition state stability and reaction exergonicity.

Solvation effects on HAT differ markedly from RAF. Three compounds (CAM, IPO, ISO) show lower barriers in lipid media compared to water, with average stabilization of –11 kJ/mol. Only MYR prefers aqueous solution (+23 kJ/mol lipid penalty). This lipid preference has important biological implications: HAT-efficient compounds (CAM, IPO, ISO) may provide enhanced protection for lipid membranes from oxidative damage, while RAF-efficient compounds are better suited for aqueous antioxidant defense. The distinct solvent dependence reflects HAT’s minimal charge separation compared to RAF’s polar transition state.

HAT represents a concerted proton-electron transfer from the neutral molecule. An alternative stepwise pathway involves initial deprotonation followed by electron transfer from the resulting anion, known as sequential proton loss electron transfer (SPLET). We examine this mechanism to complete the mechanistic landscape.

### Sequential proton loss electron transfer (SPLET) mechanism

SPLET analysis reveals that aliphatic O–H deprotonation is the rate-limiting step for this pathway in terpenoids. Deprotonation energies range from 1341–1366 kJ/mol in water SMD (see Supporting Information), corresponding to pK$_{a}$ values of 16–18 typical of aliphatic alcohols.

Comparison with literature reference compounds (Table 6) illustrates the critical role of site type in SPLET accessibility. Phenolic antioxidants exhibit deprotonation energies of 586–628 kJ/mol (pK$_{a}$ 7–10) due to resonance stabilization of the phenoxide anion. In contrast, aliphatic alcohols lack this stabilization, resulting in approximately 2-fold higher deprotonation energies.

**Table 6:** Comparison of SPLET deprotonation energies between *Syzygium nervosum* terpenoids and literature reference compounds.

Study	Compound	Site type	$\Delta G_{\text {deprot}}$	pK_a_
Present	Terpenoids	Aliphatic O-H	1341–1366	16–18
Study	(water SMD)	(alcohol)	(avg: 1352)	
Literature	Phenolic	Phenolic O-H	586–628	7–10
^15^	(deprotonation)	(resonance-stabilized)	(pK$_{a}$ 7–10)	

This site-type selectivity directs antioxidant activity toward the RAF, SET, and HAT mechanisms for these terpenoids. At physiological pH 7.4, aliphatic alcohols remain predominantly protonated, while phenolic compounds (pK$_{a}$ 7–10) exist in equilibrium between neutral and anionic forms that enable SPLET pathways. Consequently, terpenoids lacking phenolic hydroxyl groups exhibit SPLET-inaccessible thermodynamics, channeling radical scavenging activity toward the alternative mechanisms characterized above.

Solvation effects on SPLET are pronounced, with lipid media showing a +96 kJ/mol average penalty compared to water. This large solvent effect reflects the localized anionic charge generated upon deprotonation, which is poorly stabilized in low-dielectric environments. While this solvation dependence is relevant for phenolic antioxidants, it does not overcome the intrinsic thermodynamic barrier for aliphatic alcohol deprotonation.

The SPLET analysis thus establishes mechanism selectivity based on functional group type: terpenoids with phenolic or thiol groups (pK$_{a}$ 7–10) can access SPLET pathways, while those bearing only aliphatic alcohols operate primarily through RAF, SET, and HAT mechanisms. This selectivity principle guides the interpretation of antioxidant activity across different compound classes.

### Cross-mechanism comparison and multi-mechanism champions

Integrating analysis across RAF, SET, HAT, and SPLET mechanisms reveals clear structure-activity relationships and identifies multi-mechanism antioxidant champions based on quantitative performance metrics. Comparative energy profiles across all four mechanisms are provided in the Supporting Information.

The exceptional multi-mechanism performers form a distinct tier. Molecule 11 (Sesquiterpenoid, Dehydroisolongifolene, DHE) ranks first in RAF with the lowest barrier of 14 kJ/mol (site 12), making it the overall RAF champion with excellent kinetic accessibility. This compound also demonstrates the best SET performance (IP = 734 kJ/mol, Rank 1) and favorable SPLET characteristics ($\Delta G_{\text {deprot}}$ = 1354 kJ/mol, water SMD).

Molecule 4 (Monoterpenoid, (-)-Myrtenol, MYR) shows the best RAF performance among monoterpenoids with barriers of 36 kJ/mol (site 9) and 39 kJ/mol (site 6), ranking fifth overall in RAF. It has SET IP = 786 kJ/mol (rank 5) and favorable SPLET characteristics ($\Delta G_{\text {deprot}}$ = 1351 kJ/mol, water SMD). The hydroxyl group enhances radical scavenging activity across mechanisms.

Molecule 8 (Sesquiterpenoid, (+)-Carotol, CAR) emerges as the most balanced performer with consistently high activity. It ranks third in SET (IP = 770 kJ/mol), has RAF barrier = 35 kJ/mol (site 11, rank 6), and SPLET $\Delta G_{\text {deprot}}$ = 1366 kJ/mol (water SMD), making it a primary lead compound for antioxidant development.

Molecule 1 (Monoterpenoid, (-)-Camphene, CAM) demonstrates strong HAT performance with barrier = 78 kJ/mol, RAF barrier = 27 kJ/mol (site 10, rank 3), SET IP = 816 kJ/mol (rank 10), and SPLET $\Delta G_{\text {deprot}}$ = 1349 kJ/mol (water SMD), showing balanced activity across all mechanisms.

Molecule 2 (Monoterpenoid, Isopinocarveol, IPO) shows good RAF performance with barriers of 31 kJ/mol (site 10, rank 4) and 41 kJ/mol (site 6, rank 10), SET IP = 790 kJ/mol (rank 6), and SPLET $\Delta G_{\text {deprot}}$ = 1343 kJ/mol (water SMD).

Molecule 12 (Sesquiterpenoid, Isopropenyloctahydronaphthalenol, ISO) demonstrates the lowest HAT barrier (81 kJ/mol) among all compounds, with SET IP = 740 kJ/mol (rank 2) and SPLET $\Delta G_{\text {deprot}}$ = 1355 kJ/mol (water SMD).

Molecule 5 (Monoterpenoid, *l*-Verbenone, VER) shows SET IP = 844 kJ/mol (rank 11) and moderate RAF barrier = 56 kJ/mol (site 5).

Among sesquiterpenoids, Molecule 9 (Caryophyllene oxide, CAO) shows SET IP = 814 kJ/mol (rank 9) with RAF barriers of 57 kJ/mol (site 15) and 87 kJ/mol (site 9). Molecules 11 (DHE) and 10 (GLO) demonstrate similar SPLET performance with $\Delta G_{\text {deprot}}$ = 1354 kJ/mol and 1356 kJ/mol (water SMD), respectively.

The differential solvent effects between RAF and HAT mechanisms are visualized in the Supporting Information. RAF compounds show an average +5 kJ/mol offset favoring aqueous environments, whereas HAT compounds show scattered behavior around the diagonal, reflecting the mixed solvent preference. The magnitude and direction of solvent effects for all reaction sites are summarized in the Supporting Information.

The combined solvent and mechanism preferences show that RAF exhibits a modest solvent effect with a +5 kJ/mol penalty in lipid media on average, while HAT shows minimal average difference (–11 kJ/mol favoring lipid) with mixed site-specific behavior. Compounds with low barriers (<30 kJ/mol) indicate accessible pathways, moderate barriers (30–50 kJ/mol) show limited accessibility, and high barriers (>50 kJ/mol) indicate kinetically limited reactions.

These findings complement experimental studies on terpenoid-rich essential oils, which have demonstrated antioxidant activity via DPPH, ABTS, and *β*-carotene assays^30,31^. The molecular-level insights from DFT calculations provide mechanistic explanations for observed structure-activity relationships.

Key design principles emerge from these findings. For RAF mechanisms, sesquiterpenoids (DHE, ISO) and monoterpenoids with hydroxyl groups (CAM, IPO, MYR) show the lowest barriers, making them ideal for radical adduct formation pathways. For SET mechanisms, sesquiterpenoids (DHE, ISO, CAR) with lower ionization potentials (734–770 kJ/mol) are preferred for electron transfer pathways. For HAT mechanisms, compounds with weak O–H bonds and low H-abstraction barriers (ISO, CAM) are optimal for hydrogen atom transfer. Finally, multi-mechanism champions including Molecules 1, 2, 4 (monoterpenoids) and 8, 11, 12 (sesquiterpenoids) demonstrate balanced performance across RAF, SET, and SPLET mechanisms, making them versatile antioxidants capable of operating through multiple pathways simultaneously.

Spin density analysis provides electronic-structure validation for the mechanistic assignments. In RAF product radicals, Mulliken spin populations localize on the carbon atom where *·*OH added (*+0.645* to *+1.078*), confirming carbon-centred radical formation via addition rather than H abstraction. In HAT products, the unpaired electron resides on the original hydroxyl oxygen of the terpenoid (*+0.809* to *+0.888*), consistent with O-centred radical formation after O–H bond cleavage. HAT transition states show spin bridging between donor and acceptor oxygen atoms, supporting concerted H-atom transfer rather than stepwise proton or electron transfer. SET radical cations exhibit delocalised spin density across the conjugated framework, confirming electron removal from the *π* system. All $S^2$ expectation values lie within 0.753–0.783 (ideal doublet 0.750), confirming minimal spin contamination. Full spin population data are provided in the Supporting Information.

### Biological implications

The multi-mechanism champions identified in this study offer distinct therapeutic profiles. Dehydroisolongifolene (DHE, mol11), with the lowest RAF barrier (14 kJ/mol), is the lead candidate for RAF-dominant applications such as cytosolic ROS defense. (+)-Carotol (mol8), with balanced RAF (35 kJ/mol), SET (IP 770 kJ/mol), and SPLET activity, offers broad-spectrum protection ideal for environments with mixed radical exposure. (-)-Myrtenol (mol4), the top monoterpenoid RAF performer, is an accessible lead from the smaller compound class.

The opposing solvent preferences observed (RAF favored in water, HAT in lipid) have direct physiological relevance. HAT-efficient compounds (CAM, ISO) may protect lipid membranes from peroxidation, while RAF-efficient compounds defend aqueous compartments. Multi-mechanism champions that perform across both media provide compartment-spanning protection, a property particularly valuable in vivo where pH, solvent polarity, and radical speciation vary between organelles and tissues.

These findings complement experimental reports of antioxidant and cytoprotective activity in *S. nervosum* and related Myrtaceae species, providing a mechanistic foundation for the observed phenotypic effects. The structure–activity rules identified here (larger conjugation enhances SET, allylic hydroxyls enhance HAT, electron-rich double bonds enhance RAF) offer design principles for rationally optimizing terpenoid-based antioxidants through semi-synthetic modification.

### Future directions

Building upon the mechanistic insights established in this study, several research directions offer opportunities for deeper understanding and practical application.

Completing the HAT characterization for the remaining compounds would enable comprehensive cross-mechanism comparison across the full compound set. Alternative transition state localization methods or enhanced sampling techniques may facilitate convergence for the challenging systems.

Extending the computational analysis to additional reactive oxygen species, including superoxide anion, peroxyl radicals, and singlet oxygen, would broaden the understanding of terpenoid antioxidant scope beyond hydroxyl radical scavenging.

Calculation of absolute rate constants using transition state theory or QM-ORSA protocols^25^ would provide quantitative kinetic predictions directly comparable to experimental measurements.

Experimental validation through in vitro antioxidant assays (DPPH, ABTS, ORAC) and in vivo studies would confirm the computational predictions and assess biological factors including bioavailability, metabolism, and cellular uptake that lie beyond the scope of quantum chemical calculations.

## Conclusions

This study provides the first systematic computational characterization of hydroxyl radical scavenging mechanisms across thirteen bioactive terpenoids isolated from *Syzygium nervosum*. Based on the comprehensive DFT analysis, three lead compounds emerge as promising candidates for distinct antioxidant applications. Dehydroisolongifolene (molecule 11) stands out as the top overall candidate for experimental validation, exhibiting the lowest RAF kinetic barrier (14 kJ/mol at the C12 site) alongside a favorable SET ionization potential (734 kJ/mol). (+)-Carotol (molecule 8) demonstrates balanced performance across RAF, SET, and SPLET pathways, rendering it particularly suitable for broad-spectrum antioxidant applications. Meanwhile, (-)-myrtenol (molecule 4), with a RAF barrier of 36 kJ/mol, represents the most potent monoterpenoid radical adduct formation agent and offers a structurally simpler lead for further development.

The structure-activity relationships revealed in this work suggest several molecular design principles that govern terpenoid antioxidant capacity. First, the extent of *π*-conjugation plays a decisive role in electron-transfer pathways, as larger conjugated systems such as those found in sesquiterpenoids consistently lower ionization potentials and thereby enhance SET reactivity. Second, the presence of allylic or benzylic hydroxyl groups provides thermodynamically favorable HAT sites, with corresponding bond dissociation energies falling below 280 kJ/mol. Third, RAF barriers are minimized when electron-rich carbon-carbon double bonds are located at sterically accessible secondary carbons, which facilitates concerted hydroxyl radical addition. Fourth, oxygen-containing functional groups, including epoxy, carbonyl, and hydroxyl moieties, raise frontier orbital energies and improve the thermodynamic feasibility of electron-transfer pathways. Taken together, these principles transcend individual compound classes and establish a rational framework for the targeted structural modification of terpenoid antioxidants.

These computational predictions identify specific compounds together with clear mechanistic rationales that can directly guide the bioassay-guided fractionation of *S. nervosum* extracts. Prioritizing dehydroisolongifolene, (+)-carotol, and (-)-myrtenol for subsequent in vitro validation using standard antioxidant assays (DPPH, ABTS, ORAC), followed by evaluation in relevant in vivo oxidative-stress models, represents a concrete and actionable next step toward translating these mechanistic insights into validated therapeutic candidates.

## Supplementary Information

Supplementary Information.

## Data Availability

All datasets generated and/or analyzed during this study are provided in the Supporting Information, including optimized Cartesian coordinates, total electronic energies, zero-point energies, vibrational frequencies, spin densities, and Fukui indices for all 13 compounds across the four mechanisms. Additional data are available from the corresponding author on reasonable request.
