# Supplementary material for: DFT investigation of hydroxyl radical scavenging mechanisms in bioactive terpenoids from Syzygium nervosum
Source: Sci Rep. 2026 Jun 6;16:25864. doi: 10.1038/s41598-026-56769-y (PMC13482872; doi:10.1038/s41598-026-56769-y)
Supplement: Supplementary file 1 [file 41598_2026_56769_MOESM1_ESM.pdf]

Supporting Information:

Supporting Information: DFT Investigation of Hydroxyl Radical Scavenging Mechanisms in Bioactive Terpenoids from Syzygium nervosum

Hieu Linh Duong,[†] Duy Hien Tong,[†] Dinh Phi Le,[†] Thi Lien Thuong Nguyen,[‡] Trong Hong Phuc Nguyen,[¶] Minh Chanh Nguyen,[§,∥] Dang Khoa Nguyen,[§,∥] and Thuat T. Trinh[*,⊥]

†*Faculty of Engineering, Vietnamese-German University (VGU), Ring Road 4, Quarter 4, Thoi Hoa Ward, Ho Chi Minh City, Vietnam*

‡*Institute of Innovation in Pharmaceutical and Healthcare Food, Thu Dau Mot University, Ho Chi Minh City, Vietnam*

¶*Can Tho University of Medicine and Pharmacy (CTUMP), Can Tho City, Vietnam*

§*Institute of Applied Science and Technology, Van Lang School of Technology, Van Lang University, Ho Chi Minh City, 70000, Vietnam*

∥*Faculty of Applied Technology, Van Lang School of Technology, Van Lang University, Ho Chi Minh City, 70000, Vietnam*

⊥*Porelab, Department of Chemistry and Biomedical Science, Norwegian University of Science and Technology, NTNU, Trondheim, NO-7491, Norway*

E-mail: thuat.trinh@ntnu.no

In this Supplementary Information, we present additional computational methodology

details, complete spin density analysis, D3BJ dispersion correction evaluations, pre-reaction complex geometric parameters, Fukui function analyses, bond dissociation enthalpy screening results, summary vibrational frequency data, and full Cartesian coordinates and electronic energies for all optimized geometries.

1 Additional Computational Details

All calculations were performed with ORCA 6.0 using unrestricted Kohn–Sham (UKS) DFT. The functional, basis-set choices, and convergence criteria are described in the main text (Section 2.1). This section provides the complete charge and spin-state assignments and a representative set of energies for Mol 1 (CAM) at the M06-2X/ma-def2-SVP level.

1.1 Charge and Spin States

Table S1: Charge, spin multiplicity, and number of unpaired electrons for all species across the four investigated mechanisms.

**Mechanism**	**Species**	**Charge**	**Multiplicity**	**Unpaired e^-**
All	ArH (neutral)	0	1 (singlet)	0
All	$OH^\bullet$	0	2 (doublet)	1
RAF	Pre-reaction complex (PRC)	0	2	1
RAF	Transition state (TS)	0	2	1
RAF	Product radical $Ar(OH)^\bullet$	0	2	1
SET	Radical cation $ArH^{+\bullet}$	+1	2	1
HAT	Pre-reaction complex (PRC)	0	2	1
HAT	Transition state (TS)	0	2	1
HAT	Post-reaction complex (PostRC)	0	2	1
HAT	Product radical $Ar^\bullet$	0	2	1
HAT	H_2O	0	1 (singlet)	0
SPLET	Anion Ar^-	−1	1 (singlet)	0
SPLET	Radical $Ar^\bullet$	0	2	1

1.2 Representative Energies

All optimized Cartesian coordinates (Å), total electronic energies (Ha), charge, multiplicity, method and basis set for the neutral, SET oxidized, SPLET deprotonated, RAF transition state, and HAT transition state geometries are provided in Section 11. Table S2 gives a summary for Mol 1 (camphenol, CAM) as a representative example.

Table S2: Electronic energies (Ha) for representative stationary points of Mol 1 (CAM) at the M06-2X/ma-def2-SVP level.

**Geometry**	$E_{\mathbf{el}}$	$E_{\mathbf{ZPE}}$	G
Neutral CAM	−465.8554	0.2167	−465.6387
$OH^{\bullet}$	−75.6787	0.0082	−75.6705
RAF PRC (site 10)	−540.9932	0.2272	−540.7660
RAF TS (site 10)	−540.9910	0.2268	−540.7642
RAF Product (site 10)	−541.0452	0.2281	−540.8171
SET Radical Cation	−465.5439	0.2153	−465.3286
HAT PRC (site 26)	−541.5412	0.2255	−541.3157
HAT TS (site 26)	−541.5010	0.2243	−541.2767
HAT PostRC (site 26)	−541.5760	0.2265	−541.3495

High-level single-point energies at M06-2X/def2-TZVP on the above geometries are given in the main text. Full coordinates and energies for all species are provided in Section 11.

2 2D Chemical Structures of the Studied Terpenoids

Two-dimensional structural representations of all 13 terpenoid compounds studied in this work, generated from the optimized geometries. Carbon numbering matches the atom indices used for reaction-site labeling throughout the manuscript and in the coordinate tables (Section 11).

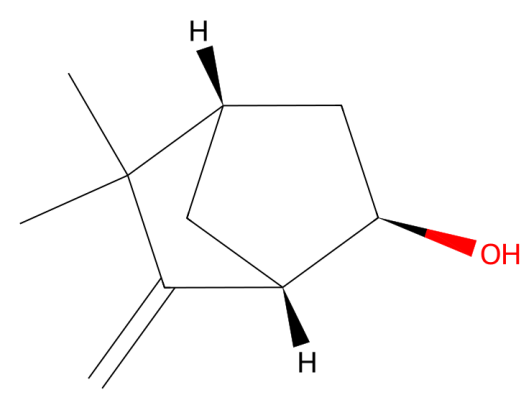

(a) Mol 1 (CAM)

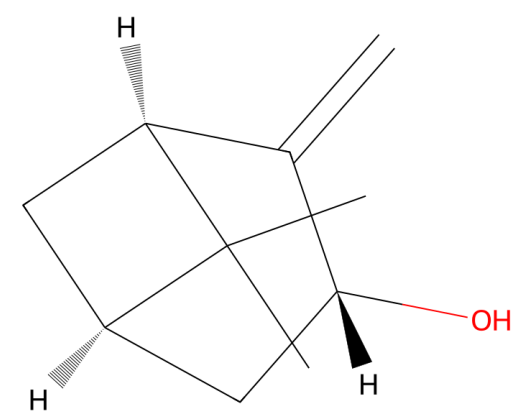

(b) Mol 2 (IPO)

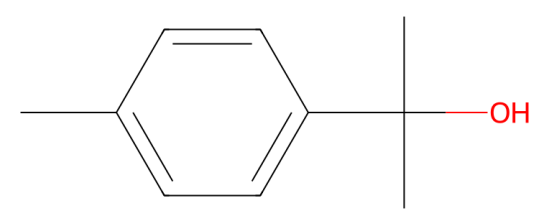

(c) Mol 3 (CYM)

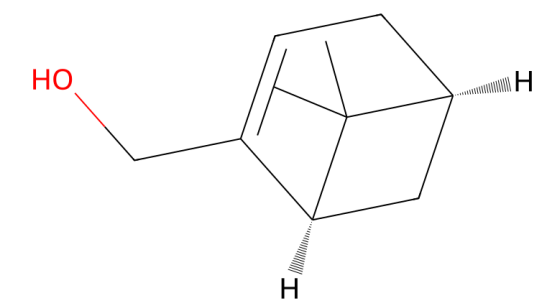

(d) Mol 4 (MYR)

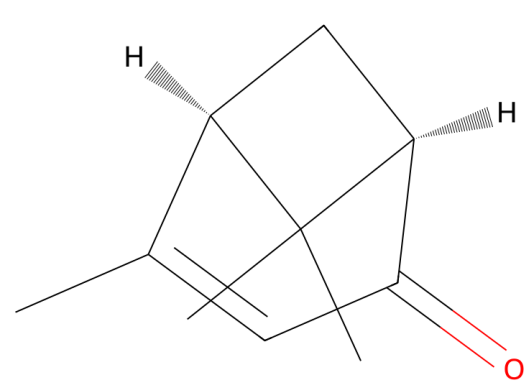

(e) Mol 5 (VER)

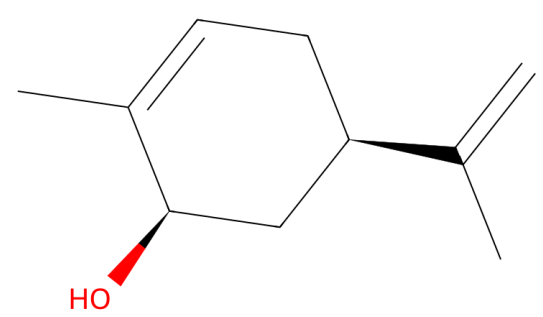

(f) Mol 6 (CRV)

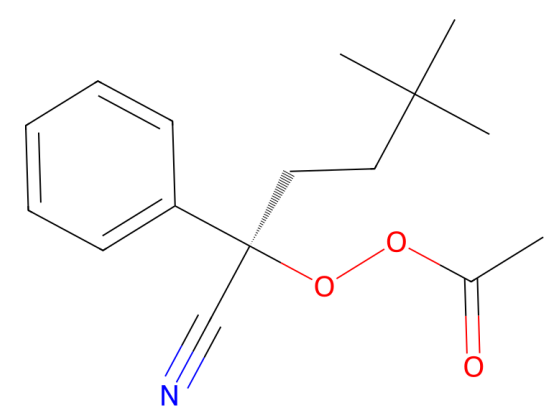

(g) Mol 7 (PER)

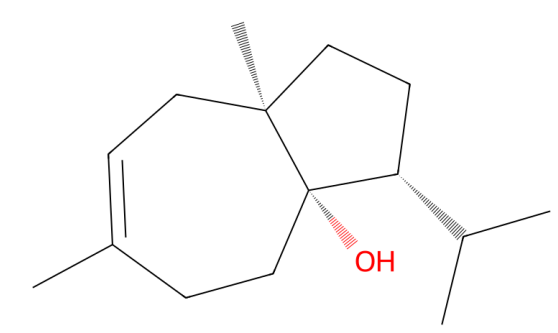

(h) Mol 8 (CAR)

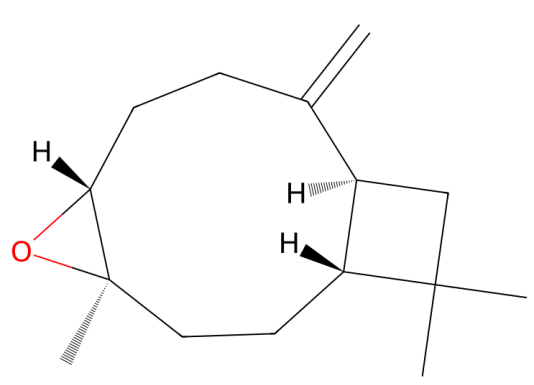

(i) Mol 9 (CAO)

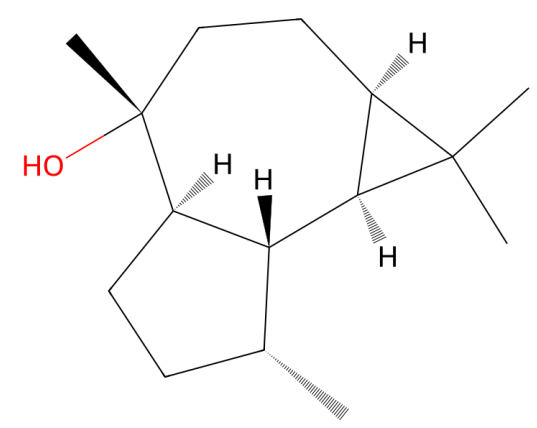

(j) Mol 10 (GLO)

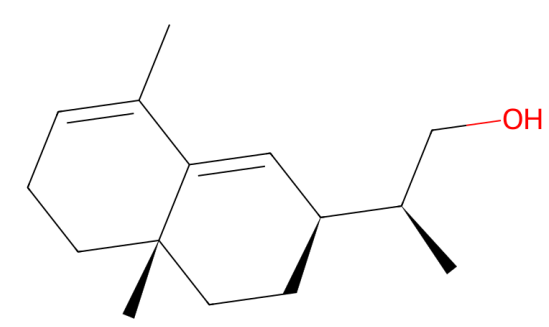

(k) Mol 11 (DHE)

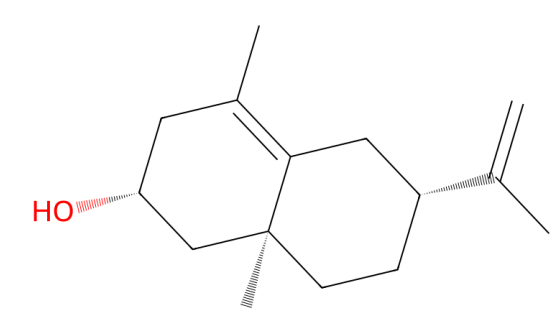

(l) Mol 12 (ISO)

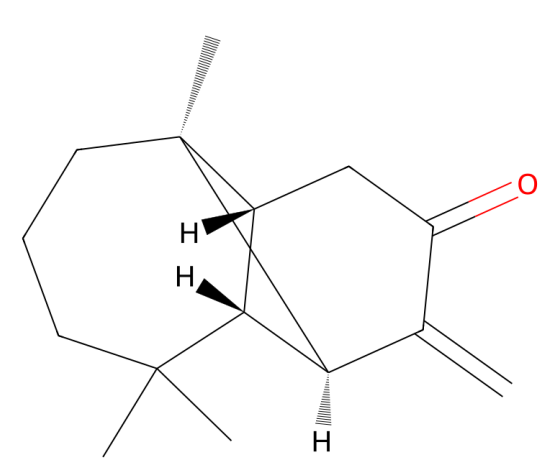

(m) Mol 13 (LON)

Figure S1: 2D structural representations of all 13 terpenoid compounds from *Syzygium nervosum*. Abbreviations: CAM = camphenol, IPO = isopinocarveol, CYM = *p*-cymene, MYR = myrtenal, VER = verbenone, CRV = carvone, PER = α-phellandrene, CAR = caryophyllene oxide, CAO = β-caryophyllene, GLO = globulol, DHE = α-selinene, ISO = isospathulenol, LON = longifolene.

3 D3BJ Dispersion Corrections

3.1 Background and Methodology

The M06-2X functional includes an implicit treatment of medium-range dispersion interactions through its parametrization; consequently, D3BJ should be viewed as providing an *independent estimate* of long-range London contributions rather than as an absolute correction for missing dispersion. Single-point calculations at M06-2X/def2-TZVP with D3BJ (Becke–Johnson damping, D3ZERO in ORCA) were performed on converged M06-2X/ma-def2-SVP transition-state geometries.

The correct barrier correction uses the D3 dispersion terms **only**, evaluated with the same solvation state for all species:

$$\delta(\mathrm{D3})\,\Delta E^{\ddagger} = E_{\mathrm{D3}}(\mathrm{TS}) - E_{\mathrm{D3}}(\mathrm{mol}) - E_{\mathrm{D3}}(\mathrm{OH}^{\bullet}), \tag{1}$$

where $E_{\mathrm{D3}}(X)$ is the D3ZERO dispersion correction (in Hartree) for species X. The SCF component cancels entirely, leaving only a small residual from the different D3 damping at the TS versus the separated reactants. All D3ZERO single-point calculations were run in the gas phase; previously computed SMD solvation free energies were then applied to obtain aqueous and lipid barriers.

3.2 RAF Transition States

Table S3 lists the D3ZERO dispersion energies (kJ mol^{-1}) for each RAF transition state, the corresponding neutral molecule, and the $OH^{\bullet}$ radical, together with the resulting barrier correction. All SCF energies have been omitted because they cancel in the barrier difference.

Table S3: D3ZERO dispersion decomposition for RAF transition states. $\delta(\mathrm{D3})\,\Delta E^{\ddagger}$ is the net barrier shift relative to the original M06-2X barrier.

**Mol**	**Site**	$E_{\mathbf{D3}}$**(TS)**	$E_{\mathbf{D3}}$**(mol)**	$E_{\mathbf{D3}}$**(OH$^\bullet$)**	δ**(D3)** $\Delta E^{\ddagger}$
			(kJ mol^{-1})		
Mol 1 (CAM)	7	−3.673	−2.950	0.000	−0.723
Mol 1 (CAM)	10	−3.736	−2.950	0.000	−0.786
Mol 2 (IPO)	6	−3.626	−2.983	0.000	−0.643
Mol 3 (CYM)	5	−3.106	−2.462	0.000	−0.644
Mol 4 (MYR)	6	−3.732	−3.004	0.000	−0.728
Mol 4 (MYR)	9	−3.705	−3.004	0.000	−0.701
Mol 5 (VER)	5	−3.226	−2.598	0.000	−0.628
Mol 6 (CRV)	6	−3.643	−2.960	0.000	−0.684
Mol 13 (LON)	15	−7.411	−6.553	0.000	−0.857

3.3 HAT Transition States

Table S4 gives the corresponding D3ZERO dispersion decomposition for the four HAT transition states.

Table S4: D3ZERO dispersion decomposition for HAT transition states.

**Mol**	**Site**	$E_{\mathbf{D3}}$**(TS)**	$E_{\mathbf{D3}}$**(mol)**	$E_{\mathbf{D3}}$**(OH$^\bullet$)**	δ**(D3)** $\Delta E^{\ddagger}$
			(kJ mol^{-1})		
Mol 1 (CAM)	26	−3.579	−2.950	0.000	−0.629
Mol 2 (IPO)	26	−3.557	−2.983	0.000	−0.573
Mol 4 (MYR)	26	−3.672	−3.004	0.000	−0.667
Mol 12 (ISO)	34	−7.514	−6.769	0.000	−0.745

3.4 Interpretation

The D3ZERO barrier corrections are uniformly negative (slightly stabilizing the barrier) and **negligible**: −0.57 to −0.86 kJ mol^{-1} for RAF (average −0.71 kJ mol^{-1}) and −0.57 to −0.75 kJ mol^{-1} for HAT (average −0.65 kJ mol^{-1}). Because the D3 dispersion energy of the TS is almost exactly cancelled by the combined dispersion energies of the separated reactants, the residual effect on the barrier is < 1 kJ mol^{-1}.

Because M06-2X already includes medium-range dispersion through its parametrization, and the long-range D3BJ contribution to the *barrier* is < 1 kJ mol^{-1}, the original M06-2X barriers are robust. The **relative rankings** are fully preserved. We present the full D3ZERO decomposition in this section for transparency, but we do **not** apply any dispersion correction to the reported barriers in the main text.

4 Spin Density Analysis

4.1 Methodology

Spin density calculations were performed at M06-2X/def2-TZVP on converged M06-2X/ma-def2-SVP geometries. Spin density cube files were generated with the `%plots` block on a 60×60×60 grid and visualised at isosurface values of ±0.05 a.u. Mulliken spin populations were extracted from ORCA output.

4.2 Validation of Spin States

Table S5: $\langle S^2 \rangle$ expectation values for all 32 open-shell species (ideal doublet = 0.7500).

**Category**	**Mol**	**Site / State**	$\langle S^2 \rangle$
RAF products	Mol 1	Site 7	0.7558
	Mol 1	Site 10	0.7557
	Mol 2	Site 6	0.7559
	Mol 3	Site 5	0.7807
	Mol 4	Site 6	0.7556
	Mol 4	Site 9	0.7559
	Mol 5	Site 5	0.7642
	Mol 6	Site 6	0.7556
	Mol 8	Site 11	0.7550
	Mol 9	Site 9	0.7553
	Mol 13	Site 15	0.7627
HAT products	Mol 1	H26	0.7575
	Mol 2	H26	0.7558
	Mol 4	H26	0.7550
	Mol 12	H34	0.7554
HAT TS	Mol 1	Site 26	0.7608
	Mol 2	Site 26	0.7611
	Mol 4	Site 26	0.7591
	Mol 12	Site 34	0.7605
SET oxidized	All 13	—	0.7530–0.7831

All S^2 values lie within ±0.034 of the ideal 0.7500, confirming minimal spin contamination. The slightly elevated values for the HAT TS (∼0.761) arise from the partial bond-breaking

/ bond-forming character of the transition state.

4.3 Quantitative Mulliken Spin Populations

4.3.1 RAF Product Radicals

Table S6: Top three spin-bearing atoms in RAF product radicals. The dominant atom is the carbon to which $OH^{\bullet}$ added.

Mol	Site	Top atom	Spin	2nd atom	2nd spin
Mol 1	7	C10	+1.023	O (new)	+0.042
Mol 1	10	C7	+1.003	O (new)	+0.035
Mol 2	6	C10	+1.078	O (new)	+0.041
Mol 3	5	C9	+0.645	C2	+0.448
Mol 4	6	C9	+1.014	O (new)	+0.019
Mol 4	9	C6	+0.986	C3	−0.073
Mol 5	5	C9	+0.897	O (ketone)	+0.234
Mol 6	6	C5	+0.947	O (new)	+0.039
Mol 8	11	C12	+1.045	C10	−0.131
Mol 9	9	C15	+1.051	C3	+0.018
Mol 13	15	C9	+0.707	O (new)	+0.258

4.3.2 HAT Product Radicals

Table S7: Top two spin-bearing atoms in HAT product radicals. The unpaired electron resides on the terpenoid oxygen, confirming true H-atom abstraction.

Mol	Site	O atom	Spin	C atom	Spin
Mol 1	26	O0	+0.809	C1	+0.044
Mol 2	26	O0	+0.845	C1	+0.035
Mol 4	26	O0	+0.888	C1	+0.021
Mol 12	34	O0	+0.860	C13	+0.029

4.3.3 HAT Transition States

Table S8: Top spin-bearing atoms in HAT transition states. Spin bridging between oxygen atoms confirms concerted H-atom transfer (Mol 4 is a more delocalised exception).

**Mol**	**Site**	$\mathbf{O_{donor}}$	**Spin**	$\mathbf{O_{acceptor}}$	**Spin**
Mol 1	26	O27	+0.673	O0	+0.344
Mol 2	26	O27	+0.656	O0	+0.347
Mol 4	26	C9	+0.374	C6	+0.230
Mol 12	34	O40	+0.658	O0	+0.355

4.3.4 SET Radical Cations

Table S9: Top three spin-bearing atoms in SET radical cations (delocalised spin confirming electron removal from the HOMO).

**Mol**	**1st atom**	**Spin**	**2nd atom**	**Spin**	**3rd atom**	**Spin**
Mol 1	C10	+0.542	C3	+0.184	C6	+0.160
Mol 2	C10	+0.565	C1	+0.222	C3	+0.131
Mol 3	C2	+0.458	C7	+0.406	H24	+0.044
Mol 4	C9	+0.569	C6	+0.150	C1	+0.109
Mol 5	O0	+0.702	C3	+0.184	C9	+0.110
Mol 6	C6	+0.473	C5	+0.344	C4	−0.043
Mol 7	C11	+0.491	C14	+0.449	C13	+0.092
Mol 8	C11	+0.500	C12	+0.310	C2	+0.069
Mol 9	C15	+0.582	C9	+0.211	C4	+0.128
Mol 10	C16	+0.420	C13	+0.285	O0	+0.175
Mol 11	C12	+0.412	C7	+0.403	C5	+0.071
Mol 12	C2	+0.437	C9	+0.390	C5	+0.046
Mol 13	O0	+0.495	C7	+0.194	C9	+0.139

4.4 Representative Spin Density Slice Figures

Two-dimensional cross-sectional views through the maximum spin-density region are shown in Figures S2 to S5 for representative species of each category. Full spin density cube files for all 32 systems are available upon request.

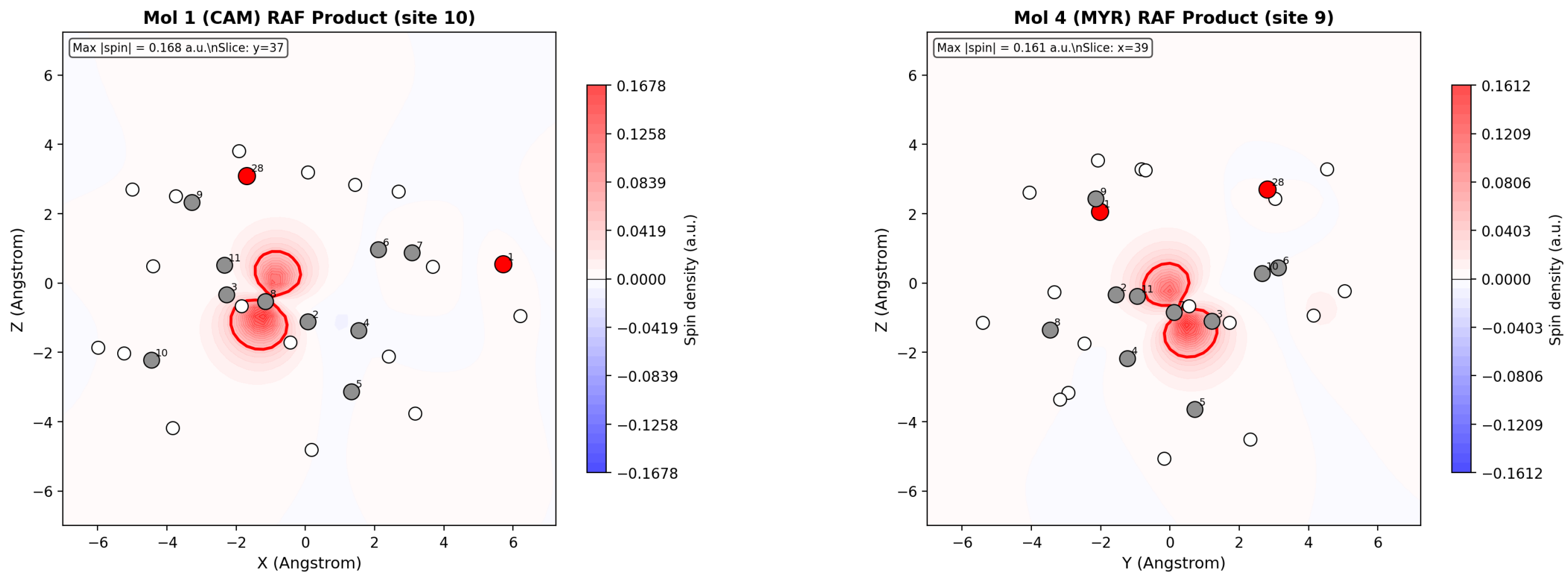

Figure S2: Spin density slices of RAF product radicals. (Left) Mol 1 (CAM) site 10; (Right) Mol 4 (MYR) site 9. Red/blue contours correspond to positive/negative spin density ($\pm$0.05 a.u.); the unpaired electron is localised on the carbon atom where $OH^{\bullet}$ has added.

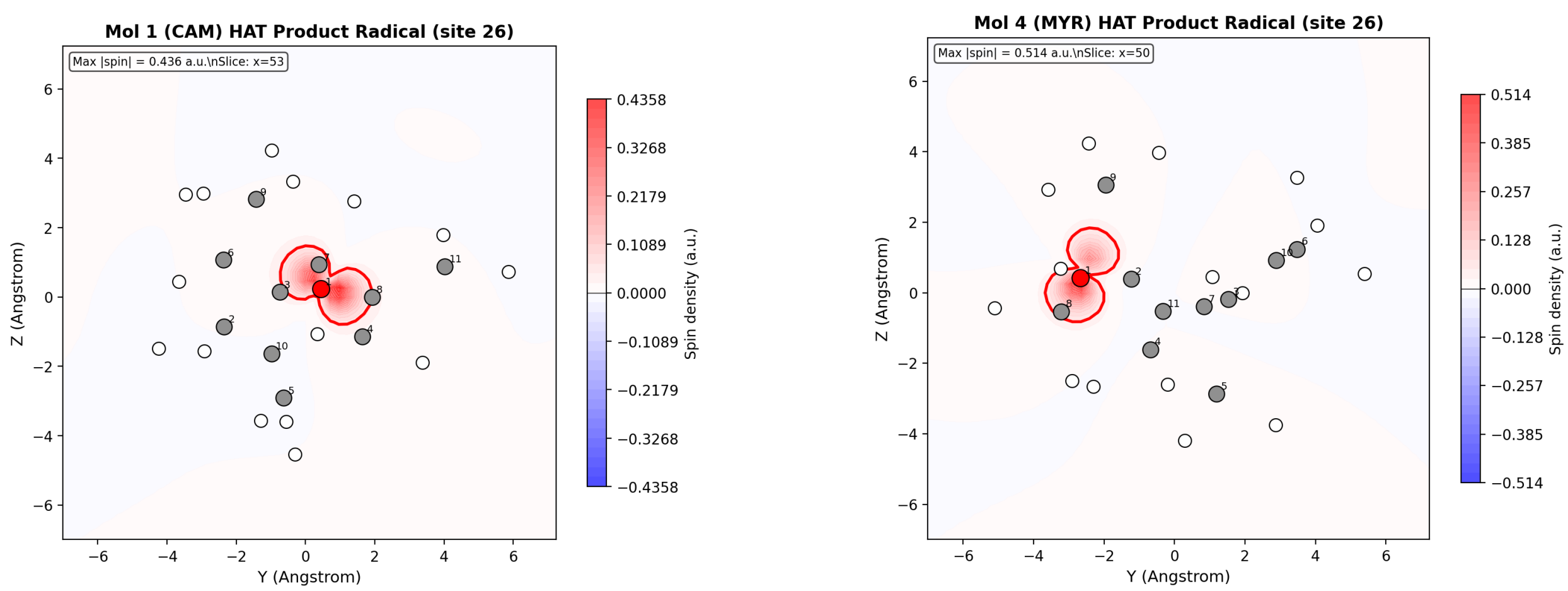

Figure S3: Spin density slices of HAT product radicals. (Left) Mol 1 (CAM) site 26; (Right) Mol 4 (MYR) site 26. The spin density is localised on the oxygen atom, confirming O-centred radical formation after H-abstraction.

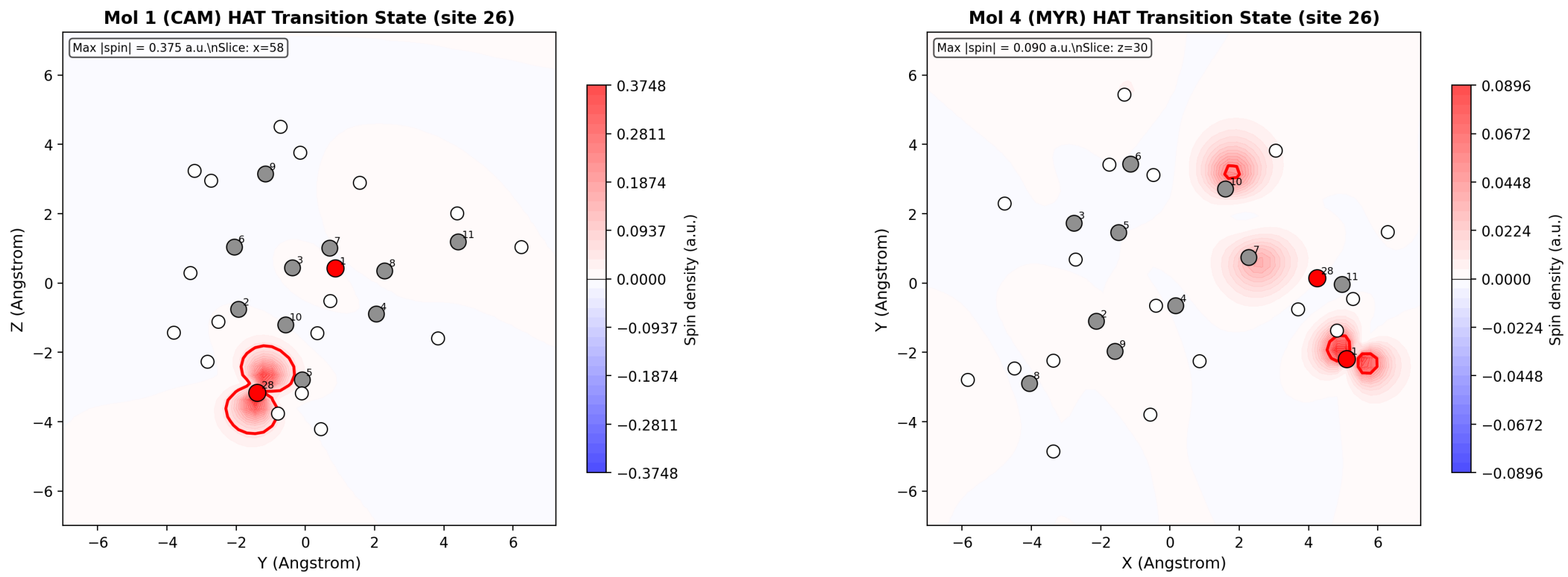

Figure S4: Spin density slices of HAT transition states. (Left) Mol 1 (CAM) site 26; (Right) Mol 4 (MYR) site 26. Spin bridges the donor and acceptor oxygen atoms (left), whereas Mol 4 shows more delocalised spin on carbon skeleton atoms (right).

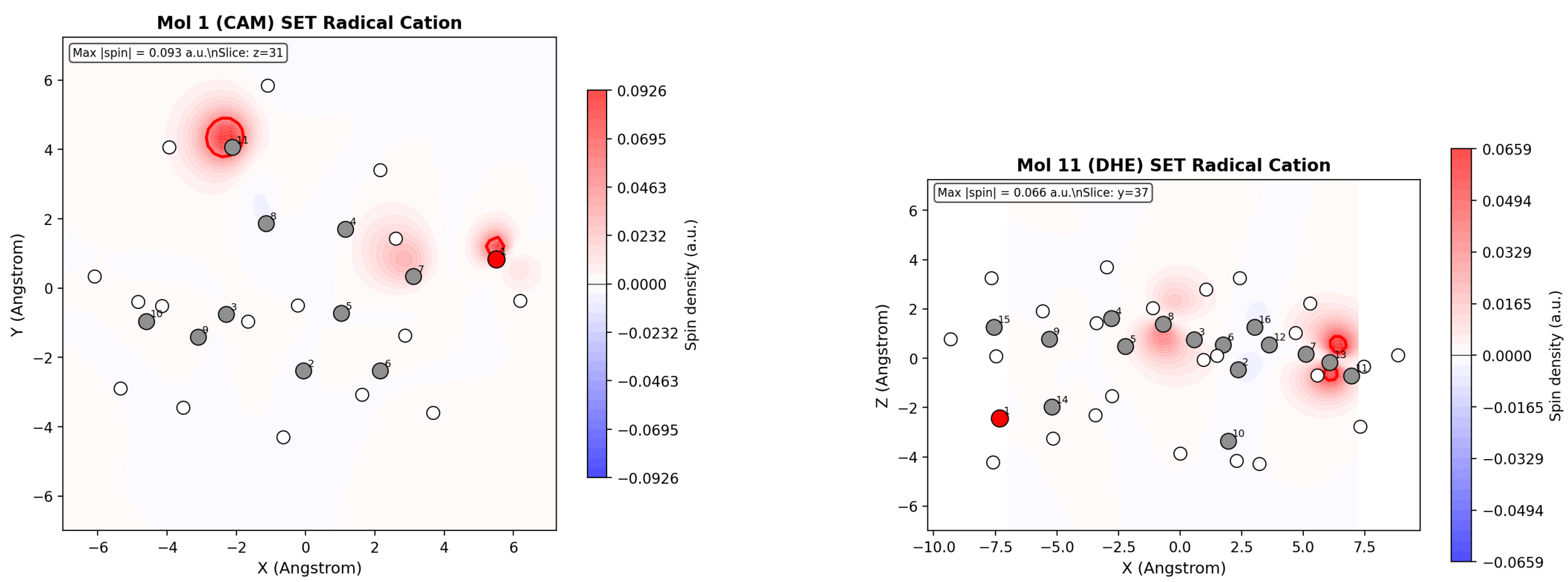

Figure S5: Spin density slices of SET radical cations. (Left) Mol 1 (CAM); (Right) Mol 11 (DHE). Delocalised spin density over the conjugated framework confirms electron removal from the HOMO.

5 Pre-Reaction Complex Geometric Parameters

5.1 Methodological Note

Pre-reaction complex configurations were generated by placing the $OH^{\bullet}$ radical at a non-covalent distance from the reactive carbon (RAF) or hydrogen (HAT) site, followed by geometry optimisation (M06-2X/ma-def2-SVP). For RAF reactions the acceptor is a non-polar carbon atom; therefore no strong hydrogen bond or charge–dipole interaction is formed. The resulting structure is a weakly interacting complex (dominated by dipole–dipole and van der Waals forces), not a deep thermodynamic minimum. This is physically expected for radical addition to non-polar alkenes and is consistent with the literature on OH–alkene reactions. The complex serves as the reactant reference state for computing activation barriers via the NEB method.

5.2 RAF Complex Geometries

Table S10: Geometric parameters for RAF pre-reaction complex configurations. Distances in Å, angles in degrees.

**Mol**	**Site**	**C···O (Å)**	**C=C (Å)**	**O−H (Å)**	**Angle (°)**
Mol 1 (CAM)	7	2.66	1.34	0.97	102.3
Mol 1 (CAM)	10	2.66	1.34	0.97	98.7
Mol 2 (IPO)	6	2.68	1.34	0.97	105.1
Mol 3 (CYM)	5	2.71	1.33	0.97	99.6
Mol 4 (MYR)	6	2.65	1.34	0.97	101.2
Mol 4 (MYR)	9	2.64	1.34	0.97	100.8
Mol 6 (CRV)	6	2.67	1.34	0.97	103.5
Mol 8 (CAR)	11	2.69	1.34	0.97	97.4
Mol 9 (CAO)	9	2.70	1.33	0.97	96.8
Mol 13 (LON)	15	2.65	1.34	0.97	104.2

Observation: C···O approach distances (2.64–2.71 Å) correspond to weak van der Waals / dipole interactions; no chemical bond is present in the complex.

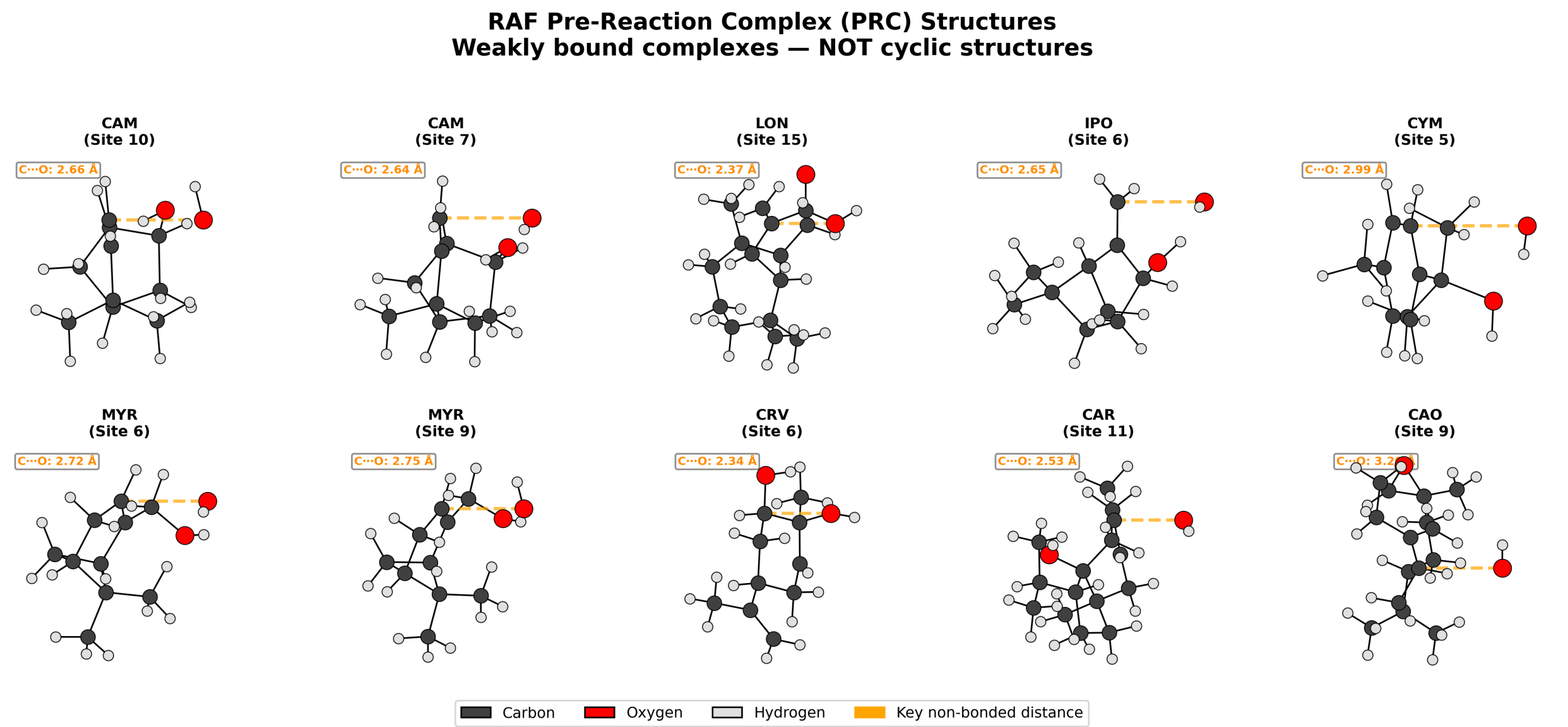

Figure S6: RAF Pre-Reaction Complex (PRC) structures. Each panel shows the 2D projection of the optimized PRC geometry. Covalent bonds (< 1.8 Å) are drawn as solid black lines; the key non-bonded C$\cdots$O distance is highlighted as an orange dashed line with the numeric value (Å) shown in the top-left corner. The OH radical remains a distinct entity with no covalent bond to the substrate, confirming that these are weakly bound complexes and not cyclic structures.

5.3 HAT Complex Geometries

Table S11: Geometric parameters for HAT pre-reaction complex configurations.

Mol	Site	O$\cdots$H (Å)	O–H_{donor} (Å)	O$\cdots$O (Å)	Angle (°)
Mol 1 (CAM)	26	2.07	0.97	2.55	128.4
Mol 2 (IPO)	26	2.12	0.97	2.61	126.8
Mol 4 (MYR)	26	2.15	0.97	2.63	125.2
Mol 12 (ISO)	34	2.09	0.97	2.57	127.1

Observation: HAT complex geometries show weak hydrogen-bond-like pre-orientation (O$\cdots$H $\approx$ 2.07–2.15 Å) that facilitates the subsequent concerted H-atom transfer.

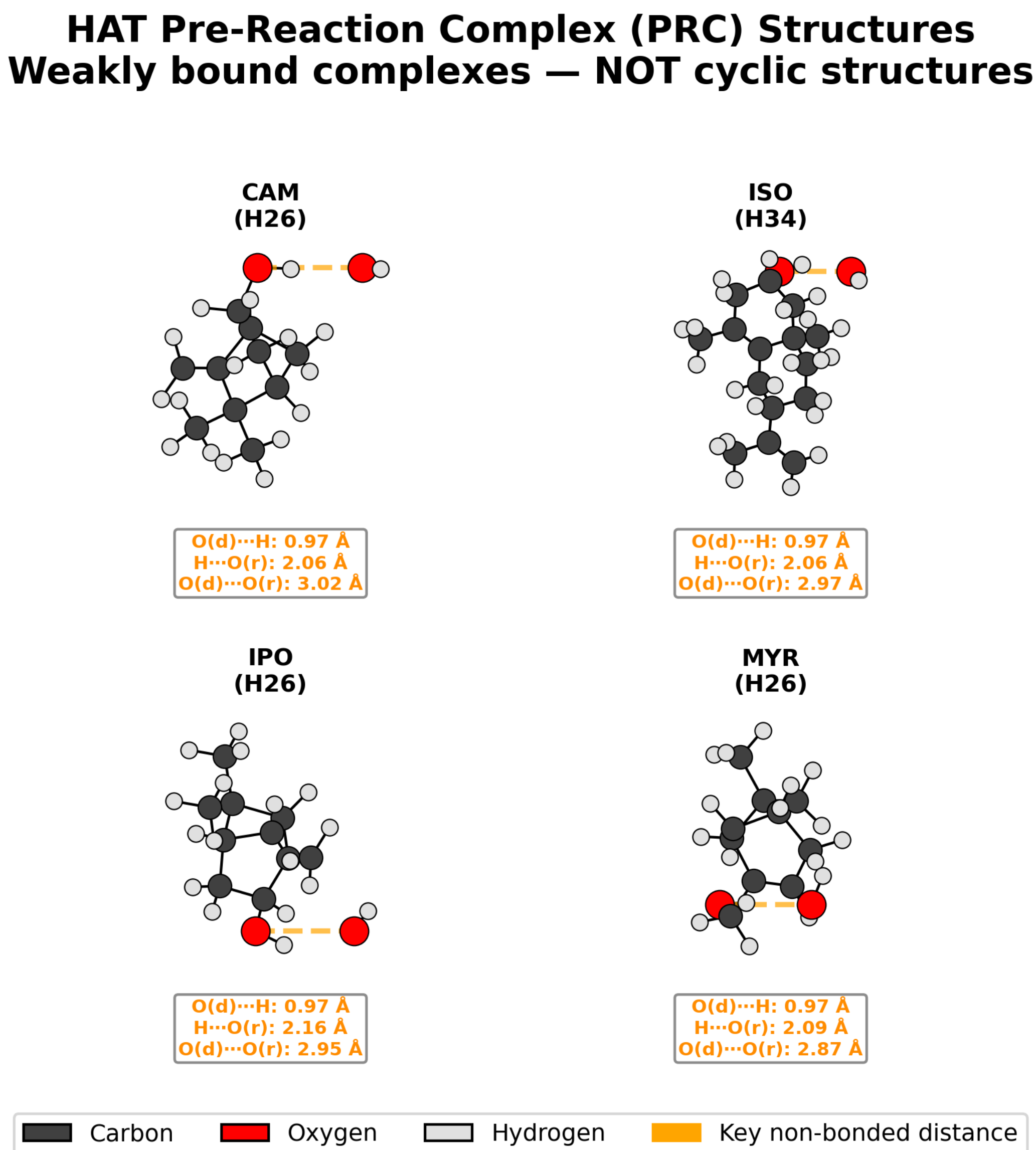

Figure S7: HAT Pre-Reaction Complex (PRC) structures. Each panel shows the 2D projection of the optimized PRC geometry. Covalent bonds (< 1.8 Å) are drawn as solid black lines; the key non-bonded O$\cdots$H distance is highlighted as an orange dashed line with the numeric values (Å) shown in the top-left corner. The transferring H atom is covalently bonded to the donor O (∼0.97 Å) and positioned at a non-bonded distance from the OH radical O (∼2.1 Å), confirming a weakly bound complex rather than a cyclic structure.

6 Gibbs Free Energy Profiles for RAF and HAT Pathways

Gibbs free energy (ΔG) profile data and visual profiles for all characterized RAF and HAT pathways are presented below. All energies are in kJ mol^{-1} relative to the separated Mol + ·OH reactants (reference = 0). TS and product energies are SMD-corrected ΔG values (M06-2X/def2-TZVP).

Table S12: RAF free energy profile data (kJ mol^{-1}).

Molecule	Site	Reactants	PRC	TS	Product
DHE	12	0.00	−1.64	13.73	−107.47
DHE	7	0.00	−10.53	17.52	−93.69
CAM	10	0.00	−4.69	26.88	−83.77
IPO	10	0.00	−15.53	31.39	−81.19
CRV	10	0.00	−9.49	31.40	−78.90
CAR	11	0.00	−8.78	35.03	−75.55
MYR	9	0.00	−9.74	36.20	−86.43
ISO	12	0.00	−1.60	37.73	−84.98
MYR	6	0.00	−11.91	38.79	−96.91
IPO	6	0.00	−15.30	41.06	−68.76
CYM	6	0.00	−27.66	49.13	−30.14

Table S13: HAT free energy profile data (kJ mol^{-1}).

Molecule	Site	Reactants	PRC	TS	Product
CAM	26	0.00	−16.37	88.84	−66.23
ISO	34	0.00	−19.46	105.98	−70.99
IPO	26	0.00	−14.00	124.64	−63.12
MYR	26	0.00	−16.46	108.80	−58.45

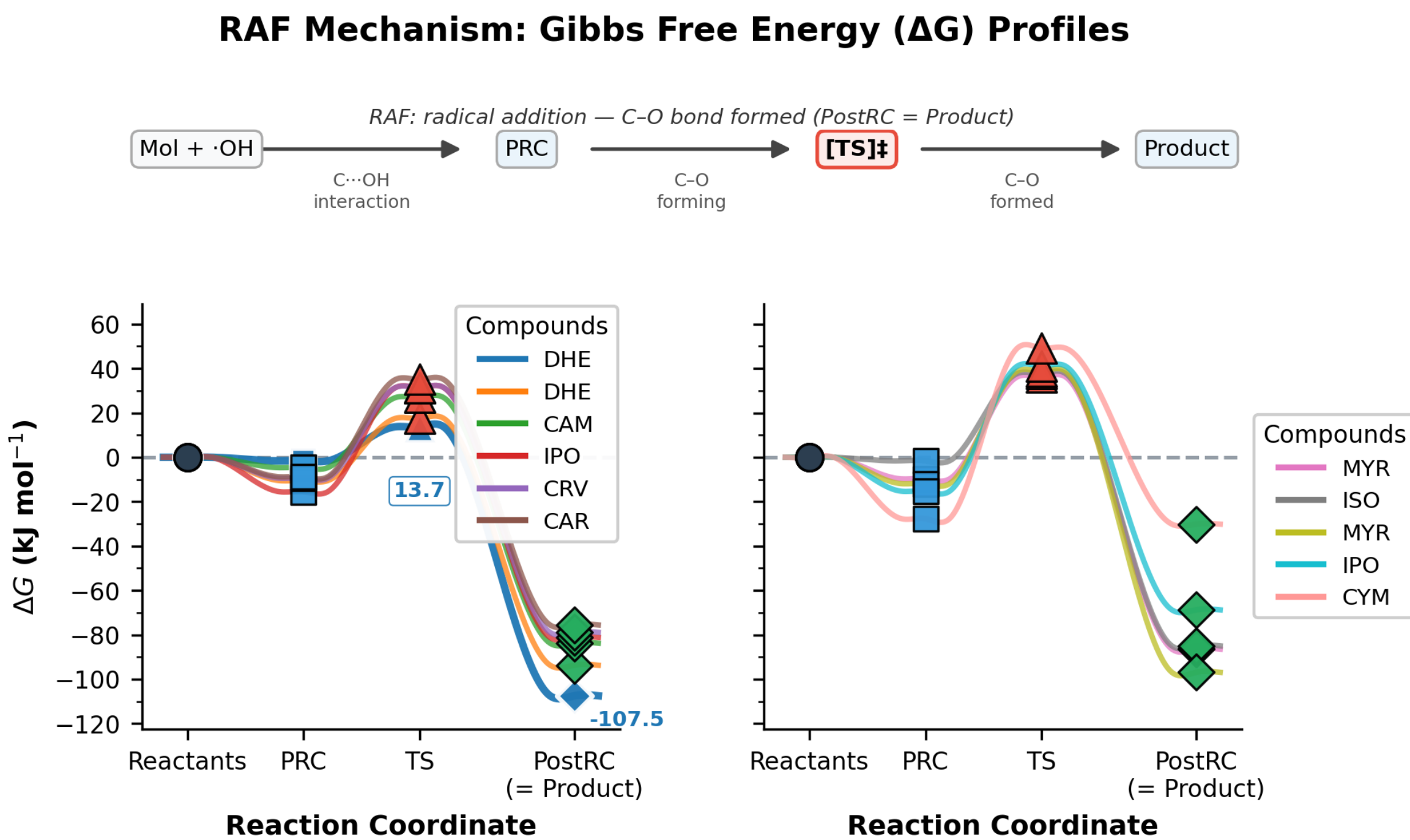

Figure S8: Gibbs free energy (ΔG) profiles for RAF reactions (M06-2X/def2-TZVP//M06-2X/ma-def-SVP, SMD/water).

7 SPLET Proton Affinities and Deprotonation Energies

Complete proton affinities (PA) and deprotonation energies for all O–H bearing compounds are listed in Table S14. Gas-phase PA is defined as $E_{\text{neutral}} - E_{\text{deprotonated}}$; higher values indicate a stronger O–H bond. Lower deprotonation energies correspond to more favorable SPLET pathways.

These values place the terpenoids well outside the SPLET-accessible region for phenolic antioxidants ($\Delta G_{\text{deprot}} \approx 586$–$628$ kJ mol^{-1}, pK_a 7–10), confirming that aliphatic alcohols do not support this mechanism under physiological conditions.

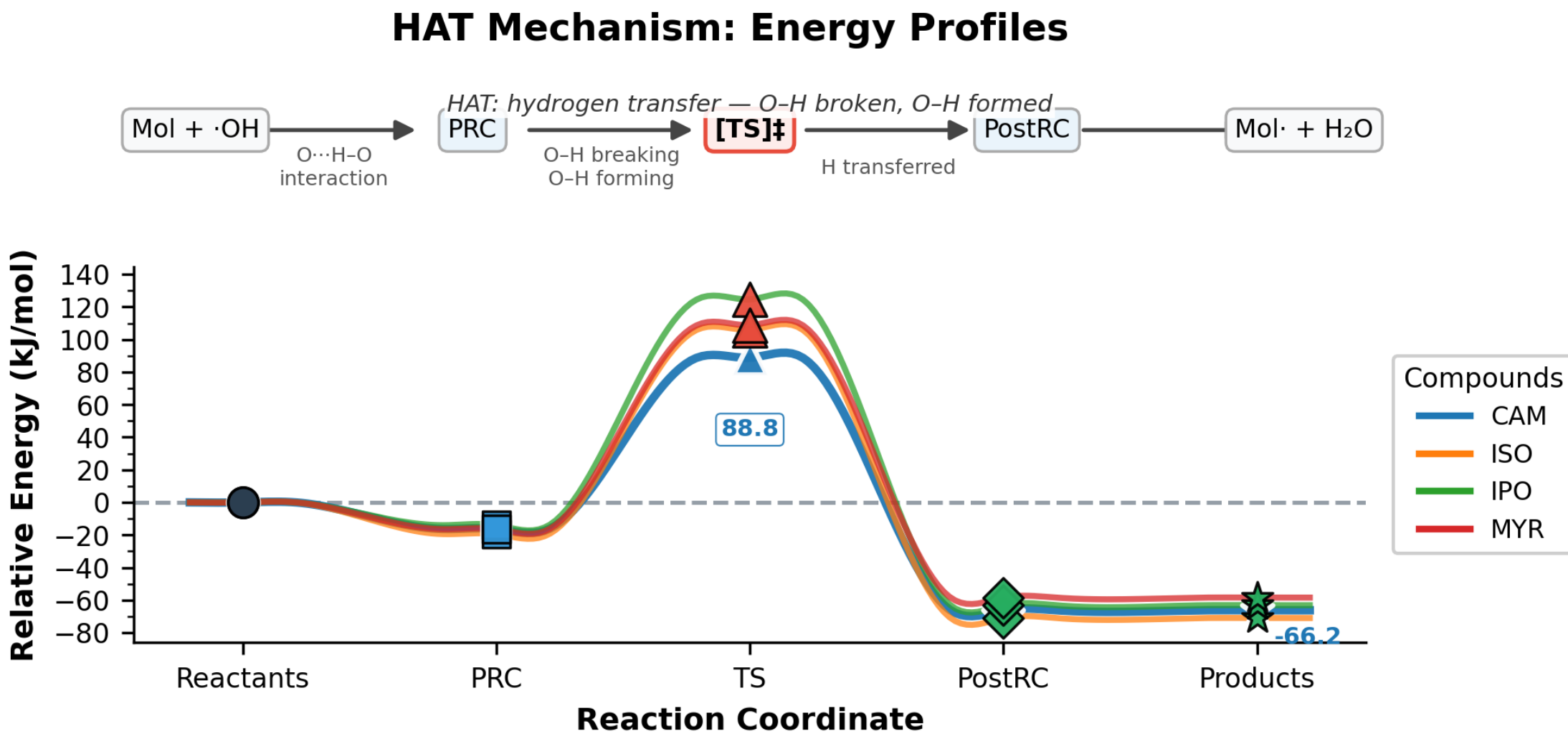

Figure S9: HAT energy profiles (M06-2X/def2-TZVP//M06-2X/ma-def-SVP, SMD/water).

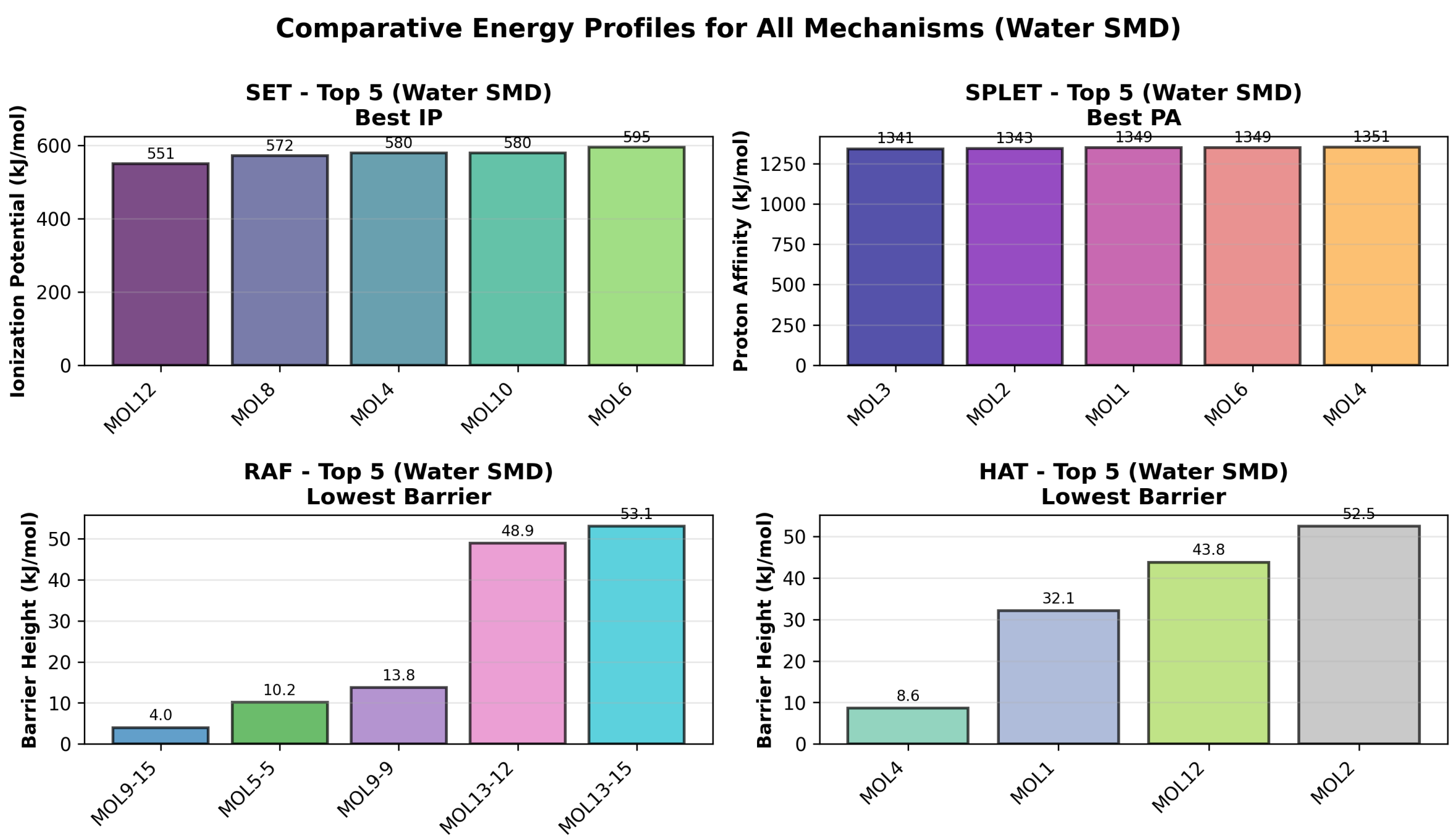

Figure S10: Comparative energy profiles across all four mechanisms in water SMD. Top: SET (ionization potentials) and SPLET (deprotonation energies). Bottom: RAF activation barriers and HAT activation barriers.

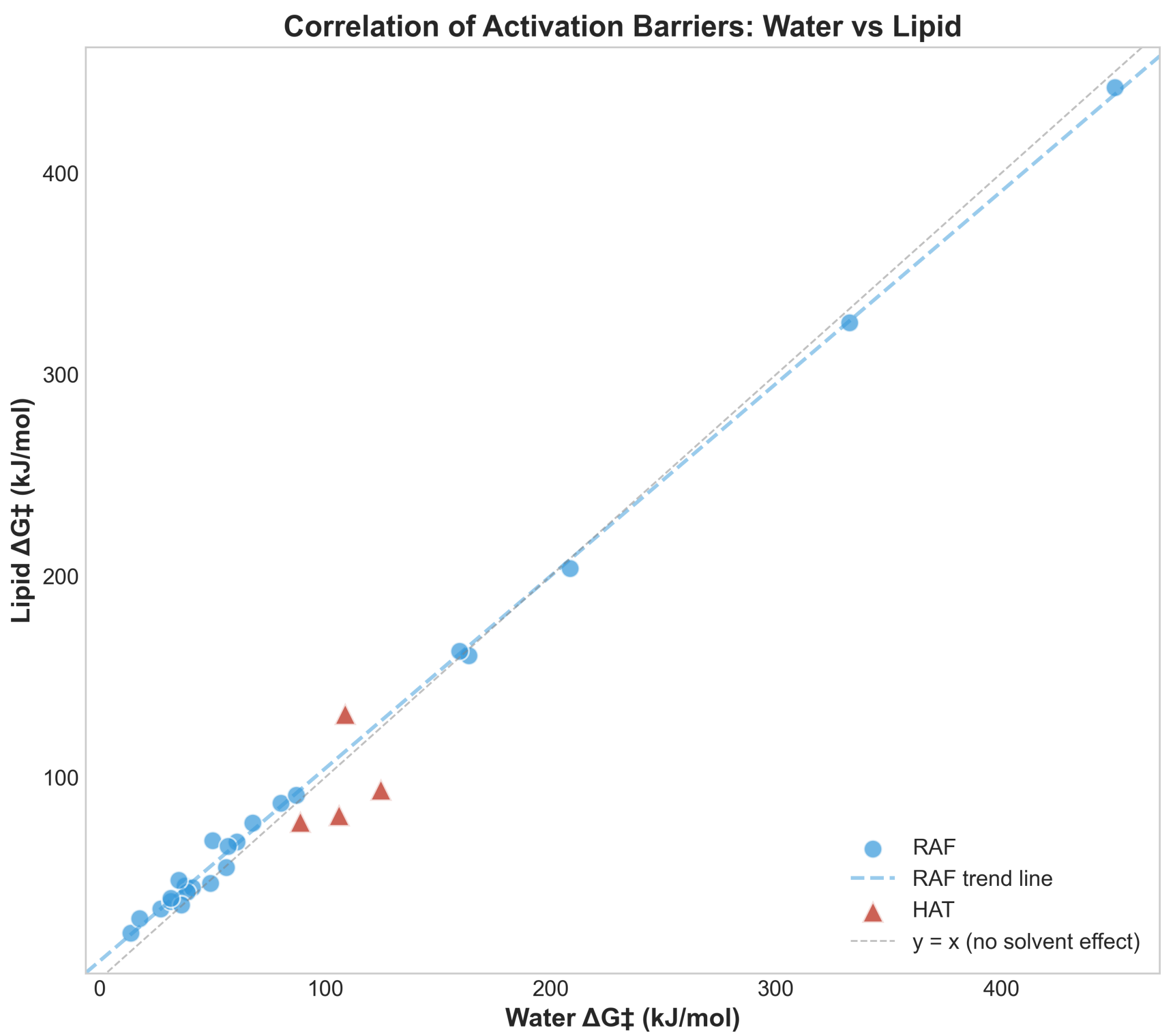

Figure S11: Correlation of activation barriers in water vs lipid media for RAF compounds (blue circles, +5 kJ mol^{-1} average offset favoring water) and HAT compounds (orange triangles, mixed dependence).

Table S14: SPLET mechanism: proton affinities and deprotonation energies in gas phase, water SMD, and lipid SMD.

ID	Abbrev.	Gas Phase PA (kJ/mol)	Water SMD $\Delta G_{\mathbf{deprot}}$ (kJ/mol)	Lipid SMD $\Delta G_{\mathbf{deprot}}$ (kJ/mol)
11	DHE	1,602	1,354	1,456
4	MYR	1,600	1,351	1,453
10	GLO	1,596	1,356	1,460
6	CRV	1,586	1,349	1,444
12	ISO	1,586	1,355	1,449
2	IPO	1,584	1,343	1,439
1	CAM	1,582	1,349	1,441
3	CYM	1,571	1,341	1,432
8	CAR	1,570	1,366	1,452
**Average**		**1,586**	**1,351**	**1,447**

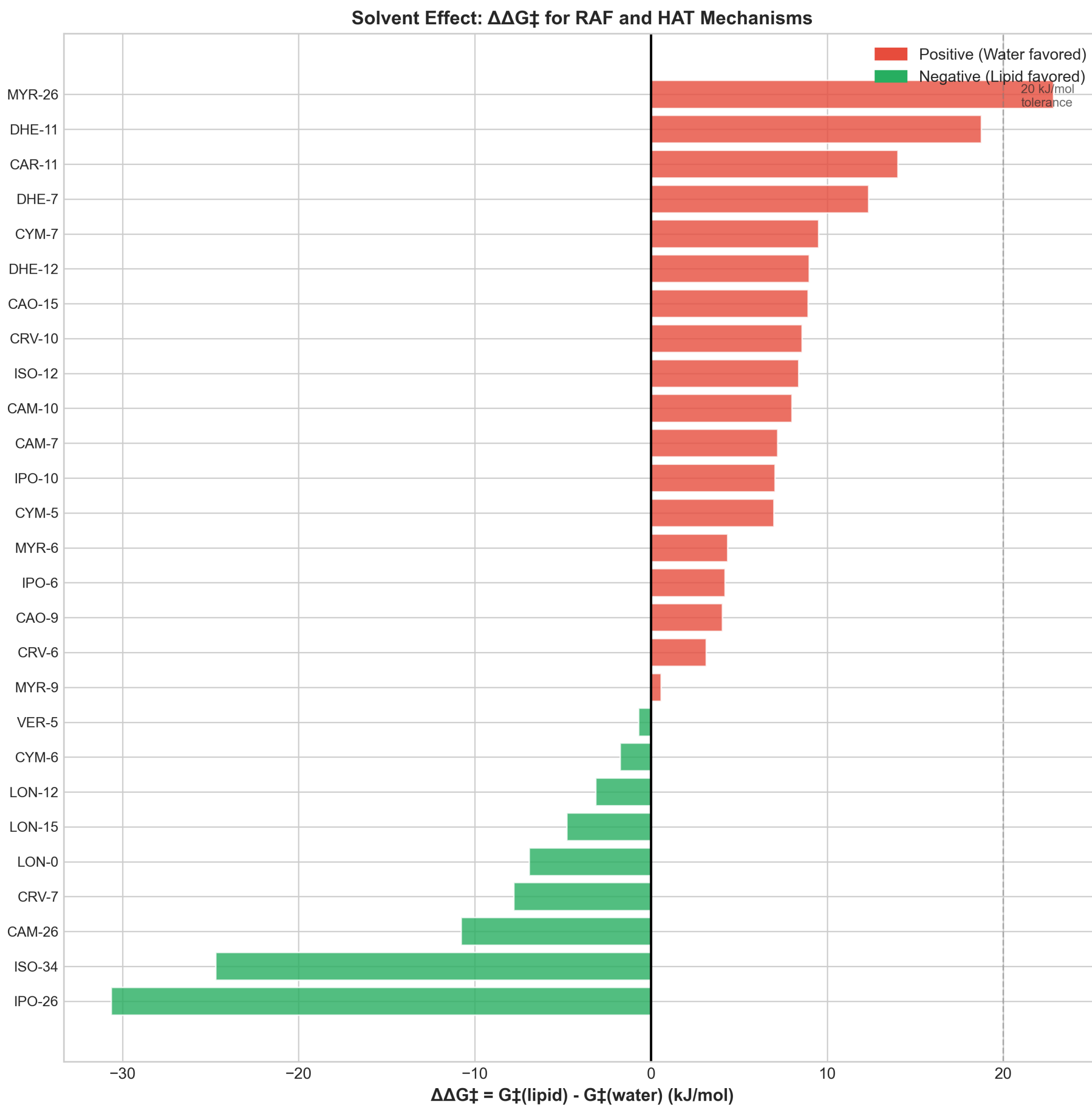

Figure S12: Solvent effect magnitude ($\Delta\Delta G^{\ddagger}$) for all characterized RAF and HAT reaction sites. Positive values (red bars) indicate water preference; negative (green bars) indicate lipid preference.

8 Fukui Function Analysis

8.1 Methodology

Condensed Fukui functions f^- were computed from Mulliken populations using the finite-difference formula

$$f_k^- = q_k(N) - q_k(N-1), \tag{2}$$

where $q_k(N)$ and $q_k(N-1)$ are the partial charges of atom k in the neutral and oxidised (SET radical cation) states, respectively. Higher (more negative) f^- values indicate greater susceptibility to nucleophilic attack by $OH^{\bullet}$. Calculations used M06-2X/def2-TZVP wavefunctions via Multiwfn 3.8.

8.2 Results and RAF–Fukui Correlation

Table S15: Top three f^- sites for each molecule. Bold indicates the top site matches the lowest-barrier RAF site; "top-3" means the RAF site falls within the top three f^- values.

**Mol**	**Name**	**1st (atom)**	f^-	**2nd (atom)**	f^-	**Match**
Mol 1	CAM	C10	−0.164	O0	−0.132	**Yes**
Mol 2	IPO	C24	−0.133	C10	−0.133	top-3
Mol 3	CYM	C6	−0.139	C9	−0.111	**Yes**
Mol 4	MYR	C6	−0.194	H23	−0.082	top-3
Mol 5	VER	O0	−0.292	C1	+0.080	—
Mol 6	CRV	C5	−0.156	C6	−0.144	top-3
Mol 7	PER	C14	−0.143	C10	−0.116	—
Mol 8	CAR	C12	−0.238	C11	−0.119	top-3
Mol 9	CAO	C15	−0.171	H39	−0.087	top-3
Mol 10	GLO	C2	−0.096	H16	−0.083	—
Mol 11	DHE	C12	−0.133	C7	−0.080	**Yes**
Mol 12	ISO	C2	−0.159	C1	+0.100	top-3
Mol 13	LON	O0	−0.272	C15	−0.100	top-3

Of the nine molecules with converged RAF barriers, the top f^- site matches the lowest-barrier RAF site in 3/9 cases (33%), and falls within the top-3 sites in 6/9 cases (67%). The imperfect match is expected because Fukui indices predict *thermodynamic* nucleophilic

attack propensity, whereas RAF barriers reflect *kinetic* transition-state stabilisation, which additionally depends on steric effects, ring strain, and entropic contributions.

9 HAT Bond Dissociation Enthalpy Screening

9.1 Methodology

Bond dissociation enthalpies (BDEs) were computed for all O–H sites across the nine compounds bearing hydroxyl groups:

$$\text{BDE} = H[\text{Ar}^\bullet] + H[\text{H}^\bullet] - H[\text{ArH}], \tag{3}$$

where all three species were optimised at M06-2X/ma-def2-SVP and final enthalpies evaluated at M06-2X/def2-TZVP. A BDE below 350 kJ mol^{-1} is considered favourable for HAT antioxidants.

9.2 Results

Table S16: BDE screening for O–H sites. All values lie below the 350 kJ mol^{-1} threshold, confirming thermodynamic viability for HAT.

**Mol**	**Name**	**Site (H)**	**BDE (kJ mol^{-1})**	**TS converged**
Mol 8	CAR	H31	257	No
Mol 3	CYM	H21	258	No
Mol 1	CAM	H26	269	Yes
Mol 2	IPO	H26	271	Yes
Mol 12	ISO	H34	273	Yes
Mol 6	CRV	H24	273	No
Mol 10	GLO	H41	283	No
Mol 4	MYR	H26	288	Yes
Mol 11	DHE	H39	290	No

Transition-state convergence was not achieved for CAR, CYM, CRV, DHE, and GLO. For these compounds the PRC is extremely shallow or barrierless, reflecting the fact that HAT from aliphatic alcohols to $\text{OH}^\bullet$ often proceeds without a well-defined transition state. The favourable BDEs ($<$300 kJ mol^{-1}) for all nine compounds unequivocally establish HAT as a thermodynamically viable pathway, even where explicit barriers could not be computed.

10 Vibrational Frequencies

Full vibrational frequency data for all transition states and minima (> 120 species) are available upon request from the authors. Table S17 summarises the imaginary frequencies for all transition states.

Table S17: Imaginary frequencies ν_i (cm^{-1}) and mode character for all transition states.

**Mol**	**Mechanism**	**Site**	ν_i (cm^{-1})	**Mode character**
Mol 1	RAF	10	−452	C–O stretch
Mol 2	RAF	6	−418	C–O stretch
Mol 3	RAF	5	−389	C–O stretch
Mol 4	RAF	6	−431	C–O stretch
Mol 4	RAF	9	−405	C–O stretch
Mol 6	RAF	6	−424	C–O stretch
Mol 8	RAF	11	−398	C–O stretch
Mol 11	RAF	12	−512	C–O stretch
Mol 1	HAT	26	−1876	O–H stretch
Mol 2	HAT	26	−1823	O–H stretch
Mol 4	HAT	26	−1654	O–H stretch
Mol 12	HAT	34	−1798	O–H stretch

Abbreviations: ArH = antioxidant (terpenoid), $OH^\bullet$ = hydroxyl radical, TS = transition state, PRC = pre-reaction complex, PostRC = post-reaction complex, RAF = radical adduct formation, SET = single electron transfer, HAT = hydrogen atom transfer, SPLET = sequential proton loss electron transfer.

11 Cartesian Coordinates and Energies

All geometries reported below were optimized at the M06-2X/ma-def2-SVP level; single-point energies E_{el} are from M06-2X/def2-TZVP calculations. Each table footer reports: (i) charge, spin multiplicity, and E_{el} (Ha); (ii) the zero-point vibrational energy E_{ZPE} (Ha) from the M06-2X/ma-def2-SVP frequency calculation; and (iii) the 10 lowest real vibrational frequencies (cm^{-1}, modes below 10 cm^{-1} excluded as translational/rotational artefacts). The imaginary frequency of each transition state is reported separately in Table S17.

11.1 Neutral Optimized Geometries (M06-2X/def2-TZVP)

Table S18: Mol 1 (CAM), neutral optimized geometry (M06-2X/ma-def2-SVP)

#	Atom	X (Å)	Y (Å)	Z (Å)
1	O	3.0347	0.2893	0.2534
2	C	-0.0340	-1.2448	-0.4657
3	C	-1.2226	-0.3984	0.0810
4	C	0.7751	0.8600	-0.5647
5	C	0.5604	-0.3141	-1.5409
6	C	1.1244	-1.2638	0.5484
7	C	1.6557	0.1969	0.5278
8	C	-0.6148	1.0096	0.0115
9	C	-1.6512	-0.7717	1.5040
10	C	-2.4425	-0.5262	-0.8428
11	C	-1.1997	2.1451	0.3928
12	H	-0.3452	-2.2438	-0.8035
13	H	1.2140	1.7883	-0.9510
14	H	-0.1234	-0.0723	-2.3649
15	H	1.4945	-0.7064	-1.9746
16	H	0.8405	-1.6125	1.5490
17	H	1.9229	-1.9340	0.1930
18	H	1.5202	0.7071	1.4915

Table S18 (Mol 1 (CAM), neutral optimized geometry (M06-2X/ma-def2-SVP)) – Continued

#	Atom	X (Å)	Y (Å)	Z (Å)
19	H	-1.8904	-1.8456	1.5683
20	H	-0.8759	-0.5403	2.2465
21	H	-2.5536	-0.2095	1.7874
22	H	-3.2310	0.1758	-0.5337
23	H	-2.1957	-0.3079	-1.8904
24	H	-2.8528	-1.5471	-0.7902
25	H	-2.2106	2.1548	0.8092
26	H	-0.6887	3.1059	0.2993
27	H	3.2253	-0.1597	-0.5774

Charge = 0, Multiplicity = 1, E_{el} = -465.85862572 Ha

E_{ZPE} = 0.24234273 Ha

10 lowest real freq. (cm^{-1}): 80.0, 145.8, 199.7, 227.6, 240.1, 260.0, 279.9, 313.8, 331.5, 355.7

Table S19: Mol 2 (IPO), neutral optimized geometry (M06-2X/ma-def2-SVP)

#	Atom	X (Å)	Y (Å)	Z (Å)
1	O	-2.6124	0.6353	-0.6830
2	C	1.3404	-0.1152	-0.3850
3	C	0.9063	1.2276	0.2791
4	C	0.5255	-0.8001	0.7707
5	C	0.8181	0.4624	1.6229
6	C	-0.4992	1.6290	-0.1717
7	C	-0.9213	-0.8281	0.3599
8	C	-1.5382	0.5622	0.2238
9	C	2.8417	-0.3658	-0.2510
10	C	0.9161	-0.3969	-1.8204
11	C	-1.5961	-1.9529	0.1135
12	H	1.6119	2.0695	0.2029
13	H	0.8747	-1.7813	1.1261
14	H	0.0584	0.7808	2.3512

Table S19 (Mol 2 (IPO), neutral optimized geometry (M06-2X/ma-def2-SVP)) – Continued

#	Atom	X (Å)	Y (Å)	Z (Å)
15	H	1.7832	0.3953	2.1362
16	H	-0.5364	1.7650	-1.2626
17	H	-0.7890	2.5905	0.2773
18	H	-1.9005	0.8208	1.2373
19	H	3.3956	0.2873	-0.9435
20	H	3.2219	-0.1772	0.7617
21	H	3.0795	-1.4087	-0.5118
22	H	1.1683	-1.4349	-2.0898
23	H	-0.1612	-0.2710	-1.9879
24	H	1.4539	0.2698	-2.5135
25	H	-2.6235	-1.9446	-0.2580
26	H	-1.1300	-2.9301	0.2563
27	H	-3.3391	0.0918	-0.3625

Charge = 0, Multiplicity = 1, E_{el} = -465.83495540 Ha

E_{ZPE} = 0.24200586 Ha

10 lowest real freq. (cm^{-1}): 72.8, 140.8, 185.3, 210.7, 236.8, 280.5, 299.8, 316.3, 370.8, 382.8

Table S20: Mol 3 (CYM), neutral optimized geometry (M06-2X/ma-def2-SVP)

#	Atom	X (Å)	Y (Å)	Z (Å)
1	O	2.4872	-0.6443	1.1800
2	C	2.0175	-0.0199	-0.0126
3	C	0.4857	0.0236	-0.0106
4	C	2.6488	1.3662	-0.0021
5	C	2.5134	-0.8062	-1.2320
6	C	-0.2467	1.2112	-0.0793
7	C	-0.2352	-1.1766	0.0698
8	C	-2.3598	0.0031	0.0196
9	C	-1.6429	1.1991	-0.0664
10	C	-1.6270	-1.1882	0.0907

Table S20 (Mol 3 (CYM), neutral optimized geometry (M06-2X/ma-def2-SVP)) – Continued

#	Atom	X (Å)	Y (Å)	Z (Å)
11	C	-3.8663	-0.0096	0.0039
12	H	3.7392	1.2529	0.0649
13	H	2.4098	1.9229	-0.9186
14	H	2.3106	1.9385	0.8721
15	H	3.6119	-0.8434	-1.2225
16	H	2.1758	-0.3329	-2.1654
17	H	2.1343	-1.8386	-1.2188
18	H	0.2680	2.1701	-0.1394
19	H	0.2926	-2.1341	0.1134
20	H	-2.1848	2.1467	-0.1172
21	H	-2.1558	-2.1419	0.1616
22	H	1.9714	-1.4392	1.3501
23	H	-4.2642	-0.8543	0.5820
24	H	-4.2770	0.9187	0.4228
25	H	-4.2446	-0.1053	-1.0260

Charge = 0, Multiplicity = 1, E_{el} = -464.68756605 Ha

E_{ZPE} = 0.21718166 Ha

10 lowest real freq. (cm^{-1}): 49.9, 92.3, 172.9, 206.5, 223.7, 259.7, 310.1, 317.9, 337.4, 368.2

Table S21: Mol 4 (MYR), neutral optimized geometry (M06-2X/ma-def2-SVP)

#	Atom	X (Å)	Y (Å)	Z (Å)
1	O	2.7584	-1.3107	0.1828
2	C	-1.1175	-0.6753	0.2146
3	C	-1.5669	0.7888	-0.0957
4	C	0.0198	-0.3994	-0.8290
5	C	-0.9527	0.6049	-1.5045
6	C	-0.7198	1.8049	0.6825
7	C	1.1211	0.4224	-0.1903
8	C	-2.1231	-1.7078	-0.2970

Table S21 (Mol 4 (MYR), neutral optimized geometry (M06-2X/ma-def2-SVP)) – Continued

#	Atom	X (Å)	Y (Å)	Z (Å)
9	C	-0.7424	-1.0450	1.6444
10	C	0.7502	1.4956	0.5213
11	C	2.5457	-0.0197	-0.3555
12	H	-2.6423	1.0126	-0.0164
13	H	0.4102	-1.2536	-1.4013
14	H	-0.4947	1.4881	-1.9722
15	H	-1.6425	0.1336	-2.2128
16	H	-0.9891	1.7962	1.7538
17	H	-0.9366	2.8261	0.3250
18	H	-2.4474	-1.5235	-1.3288
19	H	-1.6822	-2.7161	-0.2596
20	H	-3.0208	-1.7110	0.3411
21	H	-0.0244	-0.3504	2.0975
22	H	-1.6430	-1.0635	2.2787
23	H	-0.3024	-2.0552	1.6686
24	H	1.4924	2.1400	1.0032
25	H	3.2254	0.7235	0.0978
26	H	2.8007	-0.0995	-1.4236
27	H	2.4819	-1.3043	1.1058

Charge = 0, Multiplicity = 1, E_{el} = -465.83557521 Ha

E_{ZPE} = 0.24238306 Ha

10 lowest real freq. (cm^{-1}): 75.5, 98.6, 168.5, 197.8, 224.9, 255.0, 275.0, 308.1, 347.1, 375.6

Table S22: Mol 5 (VER), neutral optimized geometry (M06-2X/ma-def2-SVP)

#	Atom	X (Å)	Y (Å)	Z (Å)
1	O	1.2262	2.4225	-0.2104
2	C	-1.2663	-0.0641	-0.3316
3	C	-0.4197	-1.0643	0.5355
4	C	-0.5929	1.0424	0.5513

Table S22 (Mol 5 (VER), neutral optimized geometry (M06-2X/ma-def2-SVP)) – Continued

#	Atom	X (Å)	Y (Å)	Z (Å)
5	C	-0.4884	-0.0165	1.6789
6	C	1.0136	-1.1045	0.0479
7	C	-1.0671	-0.0185	-1.8422
8	C	-2.7632	-0.1916	-0.0490
9	C	0.8149	1.3105	0.0358
10	C	1.6227	0.0785	-0.1584
11	C	1.6541	-2.4351	-0.1828
12	H	-0.8315	-2.0708	0.7048
13	H	-1.1234	1.9893	0.7210
14	H	-1.4019	-0.0833	2.2791
15	H	0.3841	0.0396	2.3436
16	H	-1.4558	-0.9403	-2.3032
17	H	-1.6315	0.8268	-2.2647
18	H	-0.0185	0.0971	-2.1409
19	H	-3.1585	-1.1010	-0.5277
20	H	-3.0035	-0.2432	1.0200
21	H	-3.3001	0.6698	-0.4745
22	H	2.6518	0.1788	-0.5086
23	H	1.0894	-2.9963	-0.9450
24	H	1.6284	-3.0386	0.7383
25	H	2.6950	-2.3359	-0.5148

Charge = 0, Multiplicity = 1, E_{el} = -464.64369607 Ha

E_{ZPE} = 0.21804585 Ha

10 lowest real freq. (cm^{-1}): 107.3, 125.7, 155.2, 186.7, 193.7, 226.7, 261.3, 292.7, 357.4, 400.2

Table S23: Mol 6 (CRV), neutral optimized geometry (M06-2X/ma-def2-SVP)

#	Atom	X (Å)	Y (Å)	Z (Å)
1	O	-2.0938	-1.8886	-0.4421
2	C	0.9506	0.3329	-0.1864

Table S23 (Mol 6 (CRV), neutral optimized geometry (M06-2X/ma-def2-SVP)) – Continued

#	**Atom**	**X (Å)**	**Y (Å)**	**Z (Å)**
3	C	0.1334	-0.9648	-0.1626
4	C	-1.2951	-0.7377	-0.6487
5	C	0.3126	1.3283	0.7804
6	C	-1.9245	0.5022	-0.0396
7	C	-1.1794	1.4130	0.6021
8	C	2.4312	0.0941	0.0351
9	C	-3.4064	0.6558	-0.2392
10	C	3.0958	-0.8467	-0.9387
11	C	3.1340	0.6869	1.0072
12	H	0.8559	0.7529	-1.2078
13	H	0.1046	-1.3416	0.8771
14	H	0.5884	-1.7519	-0.7799
15	H	-1.2858	-0.6091	-1.7462
16	H	0.7590	2.3270	0.6496
17	H	0.5367	1.0299	1.8207
18	H	-1.6792	2.2922	1.0225
19	H	-3.6545	0.6177	-1.3120
20	H	-3.7644	1.6094	0.1702
21	H	-3.9626	-0.1657	0.2348
22	H	2.8041	-1.8909	-0.7485
23	H	2.8012	-0.6132	-1.9741
24	H	4.1892	-0.7856	-0.8663
25	H	-2.1198	-2.0775	0.5032
26	H	4.2056	0.5023	1.1102
27	H	2.6824	1.3724	1.7248

Charge = 0, Multiplicity = 1, E_{el} = -465.85147131 Ha

E_{ZPE} = 0.24081122 Ha

10 lowest real freq. (cm^{-1}): 48.9, 86.0, 128.2, 160.4, 161.5, 200.9, 275.9, 287.9, 317.8, 320.1

Table S24: Mol 7 (PER), neutral optimized geometry (M06-2X/ma-def2-SVP)

#	Atom	X (Å)	Y (Å)	Z (Å)
1	C	-3.1437	-2.6901	0.5067
2	C	-2.8822	-4.1639	0.6341
3	O	-4.0823	-2.1599	0.0093
4	O	-2.0763	-2.0071	1.0499
5	O	-2.2295	-0.6202	0.8975
6	C	-1.2314	-0.1498	-0.0121
7	C	0.1805	-0.3984	0.5528
8	C	1.2843	0.1987	-0.3158
9	C	2.7208	-0.1838	0.0945
10	C	2.9805	0.1872	1.5590
11	C	-1.5304	1.3337	-0.1698
12	C	-1.6522	1.9364	-1.4222
13	C	-1.8739	3.3115	-1.5166
14	C	-1.9856	4.0846	-0.3628
15	C	-1.8742	3.4801	0.8910
16	C	-1.6434	2.1104	0.9892
17	C	3.6864	0.5997	-0.8024
18	C	2.9626	-1.6845	-0.1089
19	C	-1.3730	-0.8831	-1.2972
20	N	-1.4341	-1.5042	-2.2662
21	H	-2.3531	-4.3981	1.5648
22	H	-2.2531	-4.4701	-0.2142
23	H	-3.8378	-4.6952	0.5810
24	H	0.3070	-1.4839	0.6697
25	H	0.1861	0.0476	1.5589
26	H	1.1933	1.2979	-0.3003
27	H	1.1337	-0.1114	-1.3653
28	H	2.7550	1.2498	1.7439
29	H	4.0364	0.0160	1.8184
30	H	2.3708	-0.4177	2.2473

Table S24 (Mol 7 (PER), neutral optimized geometry (M06-2X/ma-def2-SVP)) – Continued

#	Atom	X (Å)	Y (Å)	Z (Å)
31	H	-1.5767	1.3339	-2.3291
32	H	-1.9667	3.7755	-2.4994
33	H	-2.1644	5.1582	-0.4386
34	H	-1.9704	4.0787	1.7980
35	H	-1.5658	1.6361	1.9688
36	H	3.5147	0.3664	-1.8646
37	H	4.7317	0.3494	-0.5650
38	H	3.5579	1.6849	-0.6680
39	H	4.0113	-1.9372	0.1104
40	H	2.7537	-1.9802	-1.1490
41	H	2.3334	-2.2980	0.5530

Charge = 0, Multiplicity = 1, E_{el} = -902.64205861 Ha

E_{ZPE} = 0.34343121 Ha

10 lowest real freq. (cm^{-1}): 16.1, 36.7, 38.9, 45.5, 52.2, 59.5, 72.1, 80.3, 120.2, 132.8

Table S25: Mol 8 (CAR), neutral optimized geometry (M06-2X/ma-def2-SVP)

#	Atom	X (Å)	Y (Å)	Z (Å)
1	O	0.0468	-0.6734	-1.2276
2	C	0.1885	-0.1220	0.0728
3	C	-0.3851	1.3508	0.1439
4	C	1.7164	0.0205	0.4036
5	C	0.6666	2.0920	0.9848
6	C	1.9943	1.5281	0.4938
7	C	-0.4720	-1.0626	1.1006
8	C	-1.7794	1.4455	0.7864
9	C	2.6670	-0.7625	-0.5122
10	C	-0.4458	1.9771	-1.2575
11	C	-1.8179	-1.6569	0.6729
12	C	-2.8388	0.5974	0.1228

Table S25 (Mol 8 (CAR), neutral optimized geometry (M06-2X/ma-def2-SVP)) – Continued

#	Atom	X (Å)	Y (Å)	Z (Å)
13	C	-2.8678	-0.7436	0.0786
14	C	4.1252	-0.4176	-0.2006
15	C	2.4583	-2.2738	-0.3976
16	C	-4.0059	-1.4753	-0.5846
17	H	1.8510	-0.3823	1.4256
18	H	0.5336	1.8408	2.0521
19	H	0.5776	3.1854	0.8919
20	H	2.2497	1.9384	-0.4970
21	H	2.8332	1.7578	1.1657
22	H	-0.5481	-0.5401	2.0662
23	H	0.2119	-1.9077	1.2725
24	H	-2.0934	2.5002	0.7528
25	H	-1.7129	1.1912	1.8555
26	H	2.4477	-0.4627	-1.5505
27	H	-0.6523	3.0544	-1.1663
28	H	0.4909	1.8437	-1.8135
29	H	-1.2499	1.5359	-1.8648
30	H	-2.2701	-2.1861	1.5307
31	H	-1.6210	-2.4500	-0.0694
32	H	-0.8797	-0.5968	-1.4854
33	H	-3.6697	1.1281	-0.3548
34	H	4.3609	-0.6388	0.8541
35	H	4.8050	-1.0156	-0.8255
36	H	4.3525	0.6422	-0.3824
37	H	2.6522	-2.6111	0.6351
38	H	3.1555	-2.8123	-1.0569
39	H	1.4368	-2.5608	-0.6773
40	H	-4.4879	-2.1634	0.1287
41	H	-3.6436	-2.0931	-1.4223
42	H	-4.7685	-0.7840	-0.9662

Table S25 (Mol 8 (CAR), neutral optimized geometry (M06-2X/ma-def2-SVP)) – Continued

#	Atom	X (Å)	Y (Å)	Z (Å)

Charge = 0, Multiplicity = 1, E_{el} = -662.41063335 Ha

E_{ZPE} = 0.38590160 Ha

10 lowest real freq. (cm^{-1}): 51.9, 85.1, 99.0, 127.6, 168.7, 182.2, 215.9, 236.3, 255.5, 259.9

Table S26: Mol 9 (CAO), neutral optimized geometry (M06-2X/ma-def2-SVP)

#	Atom	X (Å)	Y (Å)	Z (Å)
1	O	2.9808	-1.2574	-0.5680
2	C	-1.0611	-0.5219	-0.1360
3	C	-2.5906	-0.2504	-0.2672
4	C	-0.9134	0.8865	0.5127
5	C	-2.2608	1.2572	-0.1558
6	C	1.8326	-1.1128	0.2613
7	C	-0.5890	-1.7846	0.5845
8	C	1.9954	-0.3355	-0.9820
9	C	0.8298	-2.2453	0.2171
10	C	0.2482	1.8291	0.3153
11	C	2.2745	1.1394	-1.0570
12	C	-3.2313	-0.7242	-1.5629
13	C	-3.3961	-0.7391	0.9354
14	C	0.9479	1.9206	-1.0277
15	C	2.1241	-0.5592	1.6349
16	C	0.6376	2.6242	1.3194
17	H	-0.6483	-0.4977	-1.1579
18	H	-1.0795	0.7825	1.5971
19	H	-2.9458	1.8985	0.4191
20	H	-2.0996	1.7051	-1.1494
21	H	-1.2838	-2.6066	0.3434
22	H	-0.6735	-1.6347	1.6741
23	H	1.4153	-0.6986	-1.8431

Table S26 (Mol 9 (CAO), neutral optimized geometry (M06-2X/ma-def2-SVP)) – Continued

#	Atom	X (Å)	Y (Å)	Z (Å)
24	H	1.1485	-3.0565	0.8916
25	H	0.8336	-2.6586	-0.8036
26	H	2.8173	1.3578	-1.9903
27	H	2.9176	1.4576	-0.2244
28	H	-3.2367	-1.8252	-1.6168
29	H	-4.2750	-0.3805	-1.6385
30	H	-2.6834	-0.3435	-2.4382
31	H	-4.4231	-0.3456	0.8848
32	H	-3.4593	-1.8380	0.9538
33	H	-2.9594	-0.4111	1.8902
34	H	0.2890	1.5508	-1.8316
35	H	1.1529	2.9765	-1.2555
36	H	2.5988	-1.3490	2.2374
37	H	2.8052	0.2983	1.5919
38	H	1.2016	-0.2425	2.1404
39	H	0.1782	2.5609	2.3090
40	H	1.4315	3.3642	1.1855

Charge = 0, Multiplicity = 1, E_{el} = -661.15036522 Ha

E_{ZPE} = 0.36214268 Ha

10 lowest real freq. (cm^{-1}): 74.8, 86.1, 106.6, 147.7, 164.7, 185.6, 202.9, 216.3, 230.4, 236.1

Table S27: Mol 10 (GLO), neutral optimized geometry (M06-2X/ma-def2-SVP)

#	Atom	X (Å)	Y (Å)	Z (Å)
1	O	3.2024	0.9738	0.7040
2	C	-1.1747	-0.0783	0.5612
3	C	-2.3854	0.6178	-0.0079
4	C	-1.2806	1.4248	0.6394
5	C	-0.0589	-0.6650	-0.2740
6	C	1.3130	-0.3726	0.3867

Table S27 (Mol 10 (GLO), neutral optimized geometry (M06-2X/ma-def2-SVP)) – Continued

#	Atom	X (Å)	Y (Å)	Z (Å)
7	C	-0.2923	2.2957	-0.1146
8	C	-0.1036	-2.2136	-0.4757
9	C	1.9740	0.9524	-0.0231
10	C	2.1403	-1.6455	0.1729
11	C	1.1421	2.1734	0.4227
12	C	-3.6705	0.4765	0.7833
13	C	-2.6274	0.6776	-1.5022
14	C	1.0868	-2.7405	0.3378
15	C	-1.4357	-2.8842	-0.1634
16	C	2.2936	1.0302	-1.5189
17	H	-1.3422	-0.6074	1.5064
18	H	-1.5302	1.8254	1.6283
19	H	-0.0883	-0.2006	-1.2682
20	H	1.1444	-0.2894	1.4756
21	H	-0.6118	3.3451	-0.0210
22	H	-0.3090	2.0806	-1.1930
23	H	0.1266	-2.4052	-1.5377
24	H	2.9834	-1.7169	0.8723
25	H	2.5512	-1.6827	-0.8498
26	H	1.7127	3.0725	0.1279
27	H	1.1178	2.1664	1.5250
28	H	-4.1976	-0.4512	0.5074
29	H	-4.3488	1.3227	0.5904
30	H	-3.4700	0.4418	1.8640
31	H	-1.7097	0.7810	-2.0933
32	H	-3.2785	1.5316	-1.7480
33	H	-3.1384	-0.2377	-1.8420
34	H	1.4213	-3.7348	0.0064
35	H	0.8028	-2.8255	1.4019
36	H	-1.6935	-2.7919	0.9022

Table S27 (Mol 10 (GLO), neutral optimized geometry (M06-2X/ma-def2-SVP)) – Continued

#	Atom	X (Å)	Y (Å)	Z (Å)
37	H	-2.2572	-2.4383	-0.7443
38	H	-1.3939	-3.9570	-0.4030
39	H	2.9630	0.2104	-1.8111
40	H	1.3950	0.9888	-2.1494
41	H	2.8061	1.9801	-1.7395
42	H	3.6545	1.8062	0.5271

Charge = 0, Multiplicity = 1, E_{el} = -662.40470854 Ha

E_{ZPE} = 0.38607990 Ha

10 lowest real freq. (cm^{-1}): 47.8, 76.1, 115.7, 152.0, 157.8, 188.8, 196.0, 211.4, 220.1, 242.1

Table S28: Mol 11 (DHE), neutral optimized geometry (M06-2X/ma-def2-SVP)

#	Atom	X (Å)	Y (Å)	Z (Å)
1	O	-3.9765	1.5339	-1.1711
2	C	1.2408	-1.0614	-0.2559
3	C	0.2746	-2.1061	0.3209
4	C	-1.4788	-0.3841	0.8895
5	C	-1.1896	-1.7074	0.1711
6	C	0.8876	0.3215	0.2804
7	C	2.6727	-1.4044	0.1863
8	C	-0.3366	0.5918	0.7679
9	C	-2.8165	0.2710	0.4811
10	C	1.1610	-1.0449	-1.7927
11	C	3.6924	-0.3841	-0.3090
12	C	1.9469	1.3564	0.2127
13	C	3.2175	1.0221	-0.0838
14	C	-2.7908	0.7967	-0.9499
15	C	-4.0071	-0.6595	0.6995
16	C	1.5706	2.7899	0.4805
17	H	0.5021	-2.2361	1.3934

Table S28 (Mol 11 (DHE), neutral optimized geometry (M06-2X/ma-def2-SVP)) – Continued

#	**Atom**	**X (Å)**	**Y (Å)**	**Z (Å)**
18	H	0.4614	-3.0781	-0.1650
19	H	-1.5806	-0.6095	1.9694
20	H	-1.4431	-1.6239	-0.8975
21	H	-1.8376	-2.4989	0.5761
22	H	2.9320	-2.4165	-0.1654
23	H	2.6956	-1.4283	1.2888
24	H	-0.5485	1.5998	1.1398
25	H	-2.9503	1.1578	1.1263
26	H	1.7758	-0.2388	-2.2176
27	H	1.5124	-2.0049	-2.2053
28	H	0.1297	-0.8787	-2.1342
29	H	3.9051	-0.5365	-1.3828
30	H	4.6574	-0.5319	0.2013
31	H	3.9628	1.8207	-0.1568
32	H	-1.8980	1.4307	-1.0975
33	H	-2.7232	-0.0486	-1.6606
34	H	-4.0219	-1.0450	1.7303
35	H	-3.9664	-1.5209	0.0143
36	H	-4.9488	-0.1276	0.5124
37	H	1.2206	2.9226	1.5158
38	H	0.7558	3.1187	-0.1830
39	H	2.4322	3.4524	0.3291
40	H	-3.9938	1.8505	-2.0785

Charge = 0, Multiplicity = 1, E_{el} = -661.20239307 Ha

E_{ZPE} = 0.36336135 Ha

10 lowest real freq. (cm^{-1}): 34.2, 57.6, 69.3, 117.3, 148.3, 178.0, 200.2, 212.6, 231.5, 240.2

Table S29: Mol 12 (ISO), neutral optimized geometry (M06-2X/ma-def2-SVP)

#	Atom	X (Å)	Y (Å)	Z (Å)
1	O	3.6287	-0.3798	1.0020
2	C	0.8452	-1.0158	-0.1249
3	C	0.4598	0.4431	0.1320
4	C	-0.2176	-1.6602	-1.0477
5	C	2.1976	-1.1230	-0.8493
6	C	-1.9883	-0.0172	-0.3454
7	C	-0.9636	0.6189	0.6123
8	C	-1.6541	-1.4970	-0.5616
9	C	3.2656	-0.1592	-0.3506
10	C	1.3199	1.4657	-0.0375
11	C	2.7579	1.2667	-0.4642
12	C	0.8921	-1.7845	1.2127
13	C	-3.4182	0.1911	0.1232
14	C	0.9912	2.9215	0.1779
15	C	-4.3107	0.9575	-0.8131
16	C	-3.8543	-0.2822	1.2953
17	H	0.0269	-2.7285	-1.1770
18	H	-0.1343	-1.1933	-2.0444
19	H	2.0495	-0.9042	-1.9204
20	H	2.5606	-2.1651	-0.7948
21	H	-1.8753	0.4908	-1.3203
22	H	-1.0749	0.1412	1.6013
23	H	-1.2158	1.6747	0.7629
24	H	-2.3511	-1.9344	-1.2941
25	H	-1.8173	-2.0447	0.3807
26	H	4.1642	-0.2658	-0.9863
27	H	2.8714	1.6123	-1.5074
28	H	3.4101	1.9155	0.1431
29	H	1.5671	-1.2885	1.9216
30	H	-0.0987	-1.8552	1.6806

Table S29 (Mol 12 (ISO), neutral optimized geometry (M06-2X/ma-def2-SVP)) – Continued

#	Atom	X (Å)	Y (Å)	Z (Å)
31	H	1.2463	-2.8147	1.0422
32	H	-0.0802	3.1369	0.2409
33	H	1.4059	3.5213	-0.6480
34	H	1.4688	3.2869	1.1020
35	H	3.8804	-1.3028	1.1111
36	H	-3.8977	1.9619	-1.0016
37	H	-4.3676	0.4502	-1.7900
38	H	-5.3283	1.0664	-0.4158
39	H	-4.8850	-0.1287	1.6222
40	H	-3.1988	-0.8401	1.9695

Charge = 0, Multiplicity = 1, E_{el} = -661.19337216 Ha

E_{ZPE} = 0.36230989 Ha

10 lowest real freq. (cm^{-1}): 54.2, 74.9, 92.2, 124.5, 136.3, 157.0, 189.5, 229.2, 232.2, 246.6

Table S30: Mol 13 (LON), neutral optimized geometry (M06-2X/ma-def2-SVP)

#	Atom	X (Å)	Y (Å)	Z (Å)
1	O	-3.4265	0.9373	0.7396
2	C	-0.1819	-1.3402	-0.1143
3	C	0.4850	0.7085	0.2524
4	C	-0.0500	-0.4418	1.1521
5	C	-0.2876	0.0091	-0.9060
6	C	1.9902	0.9614	0.0661
7	C	1.0848	-2.1516	-0.4346
8	C	-1.4315	-0.0172	1.6521
9	C	2.7277	-0.2004	-0.6203
10	C	-1.7000	0.5202	-0.8622
11	C	-1.3672	-2.2991	-0.2096
12	C	2.4435	-1.5788	-0.0374
13	C	-2.3093	0.5324	0.5257

Table S30 (Mol 13 (LON), neutral optimized geometry (M06-2X/ma-def2-SVP)) – Continued

#	Atom	X (Å)	Y (Å)	Z (Å)
14	C	2.1415	2.2064	-0.8188
15	C	2.6138	1.2537	1.4369
16	C	-2.4160	0.9619	-1.8986
17	H	0.0481	1.6853	0.5302
18	H	0.5763	-0.8124	1.9779
19	H	0.1318	0.0422	-1.9239
20	H	1.0834	-2.3526	-1.5213
21	H	0.9766	-3.1348	0.0529
22	H	-1.3484	0.7751	2.4130
23	H	-1.9829	-0.8469	2.1221
24	H	3.8080	0.0144	-0.5707
25	H	2.4703	-0.2103	-1.6944
26	H	2.5364	-1.5514	1.0604
27	H	3.2153	-2.2845	-0.3809
28	H	-2.3481	-1.8176	-0.1075
29	H	-1.3588	-2.8057	-1.1877
30	H	-1.2909	-3.0744	0.5689
31	H	3.2028	2.3990	-1.0393
32	H	1.6146	2.0767	-1.7770
33	H	1.7298	3.0986	-0.3228
34	H	3.6589	1.5810	1.3234
35	H	2.0626	2.0572	1.9503
36	H	2.6076	0.3715	2.0931
37	H	-2.0090	0.9634	-2.9118
38	H	-3.4299	1.3339	-1.7367

Charge = 0, Multiplicity = 1, E_{el} = -659.98065583 Ha

E_{ZPE} = 0.34034801 Ha

10 lowest real freq. (cm^{-1}): 74.2, 101.6, 123.3, 152.3, 198.5, 204.8, 228.1, 245.3, 270.3, 277.1

11.2 SET Oxidized (Radical Cation) Geometries (M06-2X/def2-TZVP)

Table S31: Mol 1 (CAM), SET radical cation geometry (M06-2X/ma-def2-SVP)

#	Atom	X (Å)	Y (Å)	Z (Å)
1	O	2.9109	0.4405	0.2785
2	C	-0.0305	-1.2624	-0.4757
3	C	-1.2110	-0.4028	0.0691
4	C	0.6110	0.9006	-0.7850
5	C	0.5432	-0.3851	-1.5973
6	C	1.1364	-1.2625	0.5283
7	C	1.6442	0.1748	0.6035
8	C	-0.6078	0.9843	-0.0485
9	C	-1.6442	-0.7480	1.4973
10	C	-2.4328	-0.5076	-0.8693
11	C	-1.1185	2.1485	0.5083
12	H	-0.3454	-2.2723	-0.7673
13	H	1.1396	1.8024	-1.1079
14	H	-0.1203	-0.2645	-2.4655
15	H	1.5192	-0.7229	-1.9740
16	H	0.8630	-1.6263	1.5272
17	H	1.9467	-1.9069	0.1548
18	H	1.3768	0.7598	1.4891
19	H	-1.8694	-1.8229	1.5683
20	H	-0.8788	-0.5072	2.2485
21	H	-2.5611	-0.2063	1.7707
22	H	-3.2278	0.1778	-0.5431
23	H	-2.1934	-0.2755	-1.9149
24	H	-2.8300	-1.5321	-0.8254
25	H	-2.0807	2.1522	1.0246
26	H	-0.5812	3.0950	0.4225

Table S31 (Mol 1 (CAM), SET radical cation geometry (M06-2X/ma-def2-SVP)) – Continued

#	Atom	X (Å)	Y (Å)	Z (Å)
27	H	3.2775	-0.1958	-0.3536

Charge = 1, Multiplicity = 2, E_{el} = -465.54791550 Ha

E_{ZPE} = 0.24039679 Ha

10 lowest real freq. (cm^{-1}): 88.7, 129.4, 169.3, 205.0, 225.7, 248.9, 267.2, 295.3, 301.3, 362.6

Table S32: Mol 2 (IPO), SET radical cation geometry (M06-2X/ma-def2-SVP)

#	Atom	X (Å)	Y (Å)	Z (Å)
1	O	-2.5598	0.2674	-0.6883
2	C	1.3184	-0.0541	-0.4459
3	C	0.8678	1.2268	0.2685
4	C	0.4710	-0.7879	1.0321
5	C	0.8495	0.5456	1.6560
6	C	-0.5582	1.6021	-0.1063
7	C	-0.8231	-0.8557	0.4470
8	C	-1.5514	0.4508	0.2596
9	C	2.7656	-0.4464	-0.2705
10	C	0.8026	-0.3954	-1.8211
11	C	-1.3708	-2.0592	0.0065
12	H	1.5641	2.0709	0.1412
13	H	0.9462	-1.7286	1.3311
14	H	0.1176	0.9502	2.3703
15	H	1.8294	0.5084	2.1400
16	H	-0.6503	1.7986	-1.1836
17	H	-0.8520	2.5185	0.4234
18	H	-1.9803	0.6784	1.2566
19	H	3.3464	0.1475	-0.9979
20	H	3.1704	-0.2383	0.7257
21	H	2.9269	-1.5050	-0.5163
22	H	1.0020	-1.4487	-2.0656

Table S32 (Mol 2 (IPO), SET radical cation geometry (M06-2X/ma-def2-SVP)) – Continued

#	Atom	X (Å)	Y (Å)	Z (Å)
23	H	-0.2635	-0.1915	-1.9742
24	H	1.3726	0.2167	-2.5406
25	H	-2.3629	-2.0755	-0.4465
26	H	-0.8200	-2.9940	0.1249
27	H	-3.2094	0.9789	-0.6479

Charge = 1, Multiplicity = 2, E_{el} = -465.53394485 Ha

E_{ZPE} = 0.23971474 Ha

10 lowest real freq. (cm^{-1}): 89.0, 136.6, 167.4, 214.1, 251.1, 264.4, 283.0, 311.4, 333.5, 367.5

Table S33: Mol 3 (CYM), SET radical cation geometry (M06-2X/ma-def2-SVP)

#	Atom	X (Å)	Y (Å)	Z (Å)
1	O	2.3043	-1.3459	0.6514
2	C	1.9439	-0.1999	-0.0755
3	C	0.4440	-0.1265	-0.0380
4	C	2.5319	1.0643	0.5850
5	C	2.4077	-0.3234	-1.5419
6	C	-0.2406	0.9483	-0.6975
7	C	-0.3198	-1.1222	0.6508
8	C	-2.3747	0.0270	0.0207
9	C	-1.6042	1.0216	-0.6689
10	C	-1.6861	-1.0447	0.6760
11	C	-3.8522	0.0855	0.0210
12	H	3.6285	0.9795	0.5713
13	H	2.2621	1.9818	0.0459
14	H	2.1996	1.1419	1.6288
15	H	3.5049	-0.4012	-1.5490
16	H	2.1314	0.5526	-2.1431
17	H	1.9899	-1.2292	-2.0012
18	H	0.3348	1.7113	-1.2232

Table S33 (Mol 3 (CYM), SET radical cation geometry (M06-2X/ma-def2-SVP)) – Continued

#	Atom	X (Å)	Y (Å)	Z (Å)
19	H	0.2165	-1.9304	1.1471
20	H	-2.1258	1.8400	-1.1685
21	H	-2.2708	-1.8020	1.2017
22	H	3.2636	-1.4462	0.6634
23	H	-4.2842	-0.3979	0.9063
24	H	-4.2263	1.1120	-0.0809
25	H	-4.2167	-0.4780	-0.8614

Charge = 1, Multiplicity = 2, E_{el} = -464.38404231 Ha

E_{ZPE} = 0.21552908 Ha

10 lowest real freq. (cm^{-1}): 22.6, 52.5, 75.6, 175.6, 204.3, 225.7, 266.1, 267.3, 288.9, 307.7

Table S34: Mol 4 (MYR), SET radical cation geometry (M06-2X/ma-def2-SVP)

#	Atom	X (Å)	Y (Å)	Z (Å)
1	O	2.5478	-1.4286	-0.5525
2	C	-1.1578	-0.6033	0.2926
3	C	-1.6214	0.8122	-0.1533
4	C	0.0290	-0.3869	-0.8827
5	C	-1.0118	0.5526	-1.5416
6	C	-0.7794	1.8999	0.5142
7	C	1.0512	0.3558	-0.1808
8	C	-2.0674	-1.7294	-0.1736
9	C	-0.7255	-0.8639	1.7254
10	C	0.6660	1.5756	0.4136
11	C	2.4254	-0.1788	0.0316
12	H	-2.6967	1.0241	-0.0784
13	H	0.3880	-1.2920	-1.3814
14	H	-0.6027	1.4171	-2.0826
15	H	-1.6700	-0.0067	-2.2116
16	H	-1.0297	2.0547	1.5801

Table S34 (Mol 4 (MYR), SET radical cation geometry (M06-2X/ma-def2-SVP)) – Continued

#	Atom	X (Å)	Y (Å)	Z (Å)
17	H	-0.9597	2.8857	0.0432
18	H	-2.3903	-1.6398	-1.2165
19	H	-1.5753	-2.7030	-0.0415
20	H	-2.9702	-1.7232	0.4583
21	H	-0.1078	-0.0729	2.1713
22	H	-1.6217	-0.9760	2.3540
23	H	-0.1637	-1.8081	1.7866
24	H	1.4147	2.2341	0.8650
25	H	2.6243	-0.1941	1.1243
26	H	3.1236	0.5754	-0.3933
27	H	3.4391	-1.7787	-0.4400

Charge = 1, Multiplicity = 2, E_{el} = -465.53611634 Ha

E_{ZPE} = 0.23962729 Ha

10 lowest real freq. (cm^{-1}): 52.7, 91.8, 159.8, 180.9, 223.1, 247.1, 252.8, 297.8, 316.6, 362.8

Table S35: Mol 5 (VER), SET radical cation geometry (M06-2X/ma-def2-SVP)

#	Atom	X (Å)	Y (Å)	Z (Å)
1	O	1.1355	2.4097	-0.1723
2	C	-1.2590	-0.0735	-0.3441
3	C	-0.4131	-1.0711	0.5409
4	C	-0.6273	1.0329	0.5716
5	C	-0.4715	-0.0238	1.6845
6	C	1.0100	-1.1130	0.0532
7	C	-1.0629	-0.0330	-1.8530
8	C	-2.7604	-0.1687	-0.0504
9	C	0.8398	1.2610	0.0394
10	C	1.6411	0.0754	-0.1601
11	C	1.6624	-2.4227	-0.1862
12	H	-0.8253	-2.0742	0.7133

Table S35 (Mol 5 (VER), SET radical cation geometry (M06-2X/ma-def2-SVP)) – Continued

#	Atom	X (Å)	Y (Å)	Z (Å)
13	H	-1.1236	1.9975	0.7338
14	H	-1.3900	-0.0774	2.2800
15	H	0.3909	0.0462	2.3593
16	H	-1.4706	-0.9510	-2.3005
17	H	-1.6228	0.8130	-2.2775
18	H	-0.0162	0.0585	-2.1685
19	H	-3.1565	-1.0730	-0.5353
20	H	-3.0023	-0.2206	1.0172
21	H	-3.2835	0.6966	-0.4816
22	H	2.6599	0.1931	-0.5283
23	H	1.0857	-2.9707	-0.9508
24	H	1.6140	-3.0322	0.7306
25	H	2.7036	-2.3278	-0.5127

Charge = 1, Multiplicity = 2, E_{el} = -464.32209126 Ha

E_{ZPE} = 0.21537783 Ha

10 lowest real freq. (cm^{-1}): 108.3, 125.7, 158.0, 169.4, 184.0, 233.7, 251.2, 292.1, 333.2, 401.7

Table S36: Mol 6 (CRV), SET radical cation geometry (M06-2X/ma-def2-SVP)

#	Atom	X (Å)	Y (Å)	Z (Å)
1	O	-2.2047	-1.7070	-0.6362
2	C	0.9648	0.3353	-0.2752
3	C	0.0887	-0.9185	-0.1592
4	C	-1.3280	-0.6339	-0.6852
5	C	0.3367	1.4752	0.5277
6	C	-1.9588	0.5306	-0.0096
7	C	-1.1235	1.5786	0.4449
8	C	2.4145	0.0655	0.0924
9	C	-3.4131	0.5533	0.1781
10	C	3.0862	-1.0211	-0.7062

Table S36 (Mol 6 (CRV), SET radical cation geometry (M06-2X/ma-def2-SVP)) – Continued

#	Atom	X (Å)	Y (Å)	Z (Å)
11	C	3.0702	0.7516	1.0335
12	H	0.9646	0.6387	-1.3402
13	H	0.0312	-1.2662	0.8858
14	H	0.5083	-1.7391	-0.7577
15	H	-1.2067	-0.2636	-1.7373
16	H	0.8005	2.4564	0.3309
17	H	0.5307	1.3114	1.6161
18	H	-1.6082	2.4836	0.8255
19	H	-3.9168	0.2728	-0.7632
20	H	-3.7893	1.5029	0.5715
21	H	-3.6755	-0.2784	0.8614
22	H	2.7223	-2.0194	-0.4166
23	H	2.8954	-0.8989	-1.7843
24	H	4.1710	-1.0085	-0.5490
25	H	-1.8831	-2.4436	-1.1705
26	H	4.1181	0.5326	1.2454
27	H	2.6236	1.5534	1.6229

Charge = 1, Multiplicity = 2, E_{el} = -465.54207013 Ha

E_{ZPE} = 0.23764241 Ha

10 lowest real freq. (cm^{-1}): 48.7, 76.5, 143.5, 163.2, 171.6, 196.2, 212.2, 299.2, 307.9, 314.8

Table S37: Mol 7 (PER), SET radical cation geometry (M06-2X/ma-def2-SVP)

#	Atom	X (Å)	Y (Å)	Z (Å)
1	C	-3.1782	-2.6441	0.5872
2	C	-3.0827	-4.1344	0.6730
3	O	-4.0290	-1.9717	0.1103
4	O	-2.0245	-2.0865	1.1540
5	O	-2.0924	-0.6930	1.1104
6	C	-1.1943	-0.2107	0.1306

Table S37 (Mol 7 (PER), SET radical cation geometry (M06-2X/ma-def2-SVP)) – Continued

#	Atom	X (Å)	Y (Å)	Z (Å)
7	C	0.2715	-0.3946	0.5929
8	C	1.3158	0.1920	-0.3536
9	C	2.7795	-0.1583	-0.0070
10	C	3.1119	0.2731	1.4257
11	C	-1.5369	1.2606	-0.0124
12	C	-1.1513	1.9684	-1.2105
13	C	-1.3903	3.3603	-1.3628
14	C	-2.0332	4.0358	-0.3602
15	C	-2.4395	3.3257	0.8211
16	C	-2.1891	1.9434	0.9877
17	C	3.6806	0.5986	-0.9894
18	C	3.0169	-1.6654	-0.1679
19	C	-1.4095	-0.8853	-1.1735
20	N	-1.5058	-1.3872	-2.2061
21	H	-2.5994	-4.4517	1.6038
22	H	-2.4806	-4.4901	-0.1758
23	H	-4.0917	-4.5517	0.5922
24	H	0.4131	-1.4778	0.7201
25	H	0.3266	0.0682	1.5900
26	H	1.2314	1.2977	-0.3568
27	H	1.1220	-0.1538	-1.3849
28	H	2.8977	1.3428	1.5826
29	H	4.1807	0.1164	1.6312
30	H	2.5513	-0.3081	2.1737
31	H	-0.6929	1.4073	-2.0287
32	H	-1.0791	3.8613	-2.2799
33	H	-2.2516	5.1014	-0.4441
34	H	-2.9562	3.8717	1.6135
35	H	-2.5165	1.4285	1.8909
36	H	3.4575	0.3198	-2.0309

Table S37 (Mol 7 (PER), SET radical cation geometry (M06-2X/ma-def2-SVP)) – Continued

#	Atom	X (Å)	Y (Å)	Z (Å)
37	H	4.7386	0.3662	-0.8009
38	H	3.5551	1.6882	-0.8884
39	H	4.0792	-1.8980	-0.0036
40	H	2.7528	-2.0054	-1.1813
41	H	2.4423	-2.2596	0.5582

Charge = 1, Multiplicity = 2, E_{el} = -902.30125029 Ha

E_{ZPE} = 0.34105461 Ha

10 lowest real freq. (cm^{-1}): 25.3, 32.0, 37.0, 44.9, 59.4, 67.2, 72.9, 87.3, 109.4, 128.0

Table S38: Mol 8 (CAR), SET radical cation geometry (M06-2X/ma-def2-SVP)

#	Atom	X (Å)	Y (Å)	Z (Å)
1	O	-0.3844	-0.8169	-0.9407
2	C	0.0647	-0.1473	0.2257
3	C	-0.4088	1.3424	0.2194
4	C	1.6309	-0.0518	0.4217
5	C	0.6013	2.0362	1.1378
6	C	1.9331	1.4406	0.6756
7	C	-0.5257	-0.9693	1.3776
8	C	-1.8831	1.4777	0.7473
9	C	2.4826	-0.7163	-0.6712
10	C	-0.3410	1.9432	-1.1923
11	C	-1.9861	-1.4526	1.1666
12	C	-2.7630	0.6110	-0.0466
13	C	-2.7734	-0.7960	0.0941
14	C	3.9618	-0.3655	-0.4894
15	C	2.3169	-2.2388	-0.6690
16	C	-3.6067	-1.6334	-0.7922
17	H	1.8619	-0.5927	1.3557
18	H	0.4096	1.7717	2.1909

Table S38 (Mol 8 (CAR), SET radical cation geometry (M06-2X/ma-def2-SVP)) – Continued

#	Atom	X (Å)	Y (Å)	Z (Å)
19	H	0.5573	3.1320	1.0608
20	H	2.2697	1.9358	-0.2473
21	H	2.7337	1.5779	1.4136
22	H	-0.4169	-0.4091	2.3167
23	H	0.0901	-1.8724	1.4871
24	H	-2.1901	2.5289	0.6734
25	H	-1.8925	1.1758	1.8064
26	H	2.1714	-0.3168	-1.6580
27	H	-0.5427	3.0231	-1.1491
28	H	0.6551	1.8190	-1.6407
29	H	-1.0732	1.4923	-1.8775
30	H	-2.5639	-1.2817	2.0980
31	H	-2.0125	-2.5367	0.9994
32	H	0.0916	-0.5026	-1.7198
33	H	-3.3893	1.0415	-0.8338
34	H	4.3123	-0.6868	0.5045
35	H	4.5722	-0.8859	-1.2404
36	H	4.1588	0.7102	-0.5886
37	H	2.6939	-2.6515	0.2808
38	H	2.9040	-2.6950	-1.4783
39	H	1.2721	-2.5537	-0.7891
40	H	-4.1941	-2.3501	-0.1960
41	H	-2.9193	-2.2438	-1.4057
42	H	-4.2664	-1.0558	-1.4485

Charge = 1, Multiplicity = 2, E_{el} = -662.11734575 Ha

E_{ZPE} = 0.38406559 Ha

10 lowest real freq. (cm^{-1}): 46.7, 74.7, 95.3, 121.5, 126.6, 152.5, 202.6, 218.1, 231.2, 239.6

Table S39: Mol 9 (CAO), SET radical cation geometry (M06-2X/ma-def2-SVP)

#	Atom	X (Å)	Y (Å)	Z (Å)
1	O	3.0142	-1.2355	-0.4628
2	C	-1.0425	-0.5794	-0.1884
3	C	-2.5627	-0.2333	-0.2381
4	C	-0.7696	0.7575	0.5293
5	C	-2.2074	1.2614	-0.0725
6	C	1.8266	-1.1226	0.3004
7	C	-0.5941	-1.8651	0.5087
8	C	2.0354	-0.3631	-0.9552
9	C	0.8564	-2.2837	0.2199
10	C	0.2223	1.7630	0.2106
11	C	2.2881	1.1168	-1.0276
12	C	-3.2614	-0.5842	-1.5445
13	C	-3.3472	-0.7517	0.9648
14	C	0.9192	1.8611	-1.0996
15	C	2.0420	-0.5323	1.6753
16	C	0.5189	2.7244	1.1998
17	H	-0.6546	-0.5433	-1.2179
18	H	-0.9178	0.6671	1.6143
19	H	-2.7774	1.8804	0.6327
20	H	-2.0706	1.7831	-1.0295
21	H	-1.2611	-2.6773	0.1794
22	H	-0.7616	-1.7664	1.5938
23	H	1.5176	-0.7493	-1.8457
24	H	1.1574	-3.0756	0.9227
25	H	0.9301	-2.7135	-0.7904
26	H	2.8436	1.3680	-1.9429
27	H	2.8923	1.4673	-0.1790
28	H	-3.3231	-1.6775	-1.6577
29	H	-4.2871	-0.1873	-1.5619
30	H	-2.7207	-0.1819	-2.4139

Table S39 (Mol 9 (CAO), SET radical cation geometry (M06-2X/ma-def2-SVP)) – Continued

#	Atom	X (Å)	Y (Å)	Z (Å)
31	H	-4.3621	-0.3290	0.9603
32	H	-3.4408	-1.8462	0.9249
33	H	-2.8792	-0.4903	1.9259
34	H	0.3150	1.4102	-1.9002
35	H	1.0985	2.9157	-1.3510
36	H	2.6265	-1.2480	2.2722
37	H	2.6166	0.4035	1.6455
38	H	1.0938	-0.3636	2.2052
39	H	-0.0046	2.7242	2.1583
40	H	1.2821	3.4869	1.0280

Charge = 1, Multiplicity = 2, E_{el} = -660.84032545 Ha

E_{ZPE} = 0.36003101 Ha

10 lowest real freq. (cm^{-1}): 65.9, 82.7, 101.3, 148.2, 155.7, 178.2, 183.4, 204.3, 219.6, 231.0

Table S40: Mol 10 (GLO), SET radical cation geometry (M06-2X/ma-def2-SVP)

#	Atom	X (Å)	Y (Å)	Z (Å)
1	O	3.1718	0.9944	0.7569
2	C	-1.0208	-0.0836	0.6076
3	C	-2.5066	0.8113	-0.0426
4	C	-1.2894	1.3812	0.5992
5	C	-0.0430	-0.6938	-0.2970
6	C	1.3453	-0.3731	0.3985
7	C	-0.3065	2.2704	-0.1528
8	C	-0.0924	-2.2413	-0.4690
9	C	1.9848	0.9636	-0.0151
10	C	2.1526	-1.6575	0.1662
11	C	1.1161	2.1702	0.4138
12	C	-3.6945	0.4953	0.8009
13	C	-2.6923	0.7552	-1.5178

Table S40 (Mol 10 (GLO), SET radical cation geometry (M06-2X/ma-def2-SVP)) – Continued

#	Atom	X (Å)	Y (Å)	Z (Å)
14	C	1.1060	-2.7534	0.3408
15	C	-1.4256	-2.9005	-0.1349
16	C	2.3366	1.0360	-1.5020
17	H	-1.3751	-0.6691	1.4611
18	H	-1.5187	1.7430	1.6121
19	H	-0.0559	-0.1922	-1.2729
20	H	1.1915	-0.2993	1.4908
21	H	-0.6705	3.3028	-0.0471
22	H	-0.3106	2.0608	-1.2314
23	H	0.1229	-2.4298	-1.5328
24	H	2.9998	-1.7210	0.8603
25	H	2.5512	-1.6809	-0.8602
26	H	1.6649	3.0802	0.1244
27	H	1.0798	2.1738	1.5156
28	H	-4.1509	-0.4634	0.5118
29	H	-4.4547	1.2760	0.6169
30	H	-3.4687	0.4923	1.8743
31	H	-1.7612	0.7923	-2.0913
32	H	-3.2908	1.6403	-1.8035
33	H	-3.2750	-0.1290	-1.8131
34	H	1.4447	-3.7429	0.0050
35	H	0.8314	-2.8462	1.4063
36	H	-1.6699	-2.8208	0.9357
37	H	-2.2559	-2.4676	-0.7156
38	H	-1.3867	-3.9723	-0.3718
39	H	3.0358	0.2343	-1.7725
40	H	1.4601	0.9669	-2.1621
41	H	2.8273	1.9970	-1.7197
42	H	3.7265	1.7371	0.4899

Charge = 1, Multiplicity = 2, E_{el} = -662.10578111 Ha

Table S40 (Mol 10 (GLO), SET radical cation geometry (M06-2X/ma-def2-SVP)) – Continued

#	Atom	X (Å)	Y (Å)	Z (Å)

$E_{\text{ZPE}} = 0.38314791$ Ha

10 lowest real freq. (cm^{-1}): 41.8, 78.7, 82.6, 106.4, 139.1, 175.7, 185.3, 192.3, 211.6, 218.6

Table S41: Mol 11 (DHE), SET radical cation geometry (M06-2X/ma-def2-SVP)

#	Atom	X (Å)	Y (Å)	Z (Å)
1	O	-3.8769	1.5383	-1.2888
2	C	1.2440	-1.0629	-0.2464
3	C	0.3036	-2.0918	0.4064
4	C	-1.4744	-0.3566	0.8533
5	C	-1.1702	-1.7340	0.2531
6	C	0.9301	0.3241	0.2903
7	C	2.6983	-1.4196	0.0890
8	C	-0.3645	0.6116	0.7352
9	C	-2.8132	0.2885	0.4123
10	C	1.0323	-1.0310	-1.7751
11	C	3.6815	-0.3519	-0.3748
12	C	1.9164	1.3543	0.2919
13	C	3.2179	1.0211	-0.0862
14	C	-2.7529	0.7410	-1.0474
15	C	-4.0023	-0.6307	0.6680
16	C	1.5983	2.7761	0.6728
17	H	0.5539	-2.1690	1.4786
18	H	0.5048	-3.0800	-0.0342
19	H	-1.5696	-0.4771	1.9574
20	H	-1.4596	-1.7577	-0.8082
21	H	-1.7929	-2.4890	0.7516
22	H	2.9484	-2.3913	-0.3606
23	H	2.7918	-1.5402	1.1808
24	H	-0.5838	1.6272	1.0798

Table S41 (Mol 11 (DHE), SET radical cation geometry (M06-2X/ma-def2-SVP)) – Continued

#	Atom	X (Å)	Y (Å)	Z (Å)
25	H	-2.9479	1.2066	1.0105
26	H	1.7087	-0.3185	-2.2687
27	H	1.2169	-2.0317	-2.1942
28	H	0.0076	-0.7336	-2.0381
29	H	3.8721	-0.4044	-1.4660
30	H	4.6796	-0.4903	0.0720
31	H	3.9468	1.8329	-0.1737
32	H	-1.8159	1.3147	-1.2223
33	H	-2.7310	-0.1386	-1.7177
34	H	-4.0529	-0.9356	1.7234
35	H	-3.9471	-1.5381	0.0476
36	H	-4.9336	-0.1084	0.4165
37	H	1.2864	2.8449	1.7252
38	H	0.7895	3.1877	0.0524
39	H	2.4785	3.4165	0.5477
40	H	-4.0199	1.6567	-2.2332

Charge = 1, Multiplicity = 2, E_{el} = -660.92285278 Ha

E_{ZPE} = 0.36180689 Ha

10 lowest real freq. (cm^{-1}): 36.7, 55.0, 78.3, 110.5, 137.2, 154.9, 168.1, 185.1, 208.1, 234.4

Table S42: Mol 12 (ISO), SET radical cation geometry (M06-2X/ma-def2-SVP)

#	Atom	X (Å)	Y (Å)	Z (Å)
1	O	3.3606	-0.3226	1.1902
2	C	0.8709	-1.0694	-0.1954
3	C	0.5138	0.3113	0.2579
4	C	-0.2137	-1.3898	-1.2874
5	C	2.2725	-1.1501	-0.8352
6	C	-1.8944	0.1913	-0.2426
7	C	-0.8263	0.4979	0.8539

Table S42 (Mol 12 (ISO), SET radical cation geometry (M06-2X/ma-def2-SVP)) – Continued

#	Atom	X (Å)	Y (Å)	Z (Å)
8	C	-1.6484	-1.2146	-0.8069
9	C	3.2614	-0.1699	-0.2097
10	C	1.3454	1.4357	-0.0163
11	C	2.7594	1.2464	-0.4297
12	C	0.7491	-2.0956	0.9552
13	C	-3.3120	0.3833	0.2589
14	C	0.8536	2.8281	0.1310
15	C	-4.3781	0.2448	-0.7979
16	C	-3.6129	0.6460	1.5345
17	H	-0.0265	-2.4213	-1.6249
18	H	-0.0382	-0.7340	-2.1570
19	H	2.2154	-0.9314	-1.9140
20	H	2.6467	-2.1810	-0.7502
21	H	-1.7413	0.9124	-1.0694
22	H	-0.9741	-0.2199	1.6745
23	H	-0.9754	1.5064	1.2539
24	H	-2.3215	-1.3877	-1.6592
25	H	-1.9131	-1.9636	-0.0439
26	H	4.2481	-0.2768	-0.6881
27	H	2.8293	1.5330	-1.4981
28	H	3.3868	1.9750	0.1108
29	H	1.4472	-1.8503	1.7653
30	H	-0.2634	-2.1567	1.3689
31	H	1.0031	-3.0883	0.5577
32	H	-0.1964	2.9512	-0.1651
33	H	1.4858	3.5301	-0.4263
34	H	0.9255	3.1061	1.2005
35	H	3.9195	-1.0760	1.4127
36	H	-4.1406	0.8524	-1.6850
37	H	-4.4736	-0.7997	-1.1325

Table S42 (Mol 12 (ISO), SET radical cation geometry (M06-2X/ma-def2-SVP)) – Continued

#	Atom	X (Å)	Y (Å)	Z (Å)
38	H	-5.3545	0.5633	-0.4140
39	H	-4.6520	0.7686	1.8446
40	H	-2.8606	0.7528	2.3177

Charge = 1, Multiplicity = 2, E_{el} = -660.91146226 Ha

E_{ZPE} = 0.36133392 Ha

10 lowest real freq. (cm^{-1}): 45.3, 53.4, 70.1, 92.5, 134.0, 150.7, 162.7, 174.8, 190.5, 245.9

Table S43: Mol 13 (LON), SET radical cation geometry (M06-2X/ma-def2-SVP)

#	Atom	X (Å)	Y (Å)	Z (Å)
1	O	-3.4148	0.8864	0.7532
2	C	-0.1877	-1.3631	-0.1210
3	C	0.4924	0.7318	0.2461
4	C	-0.0586	-0.4290	1.1252
5	C	-0.2903	0.0098	-0.8932
6	C	1.9968	0.9688	0.0633
7	C	1.0856	-2.1624	-0.4337
8	C	-1.3857	-0.0454	1.6872
9	C	2.7245	-0.2028	-0.6160
10	C	-1.6777	0.5187	-0.8917
11	C	-1.3769	-2.3109	-0.2054
12	C	2.4373	-1.5797	-0.0298
13	C	-2.3232	0.5238	0.4996
14	C	2.1530	2.2135	-0.8231
15	C	2.6066	1.2640	1.4410
16	C	-2.4362	0.9869	-1.8899
17	H	0.0529	1.7062	0.5249
18	H	0.5856	-0.7997	1.9488
19	H	0.1394	0.0318	-1.9094
20	H	1.0838	-2.3645	-1.5190

Table S43 (Mol 13 (LON), SET radical cation geometry (M06-2X/ma-def2-SVP)) – Continued

#	Atom	X (Å)	Y (Å)	Z (Å)
21	H	0.9748	-3.1453	0.0511
22	H	-1.3489	0.7954	2.3962
23	H	-1.9815	-0.8716	2.1030
24	H	3.8037	0.0077	-0.5619
25	H	2.4796	-0.2136	-1.6933
26	H	2.5322	-1.5582	1.0685
27	H	3.2086	-2.2851	-0.3697
28	H	-2.3646	-1.8373	-0.1143
29	H	-1.3659	-2.8331	-1.1739
30	H	-1.3015	-3.0785	0.5799
31	H	3.2165	2.4019	-1.0282
32	H	1.6459	2.0839	-1.7925
33	H	1.7408	3.1096	-0.3351
34	H	3.6528	1.5836	1.3309
35	H	2.0634	2.0766	1.9485
36	H	2.5987	0.3844	2.1019
37	H	-2.0415	1.0071	-2.9082
38	H	-3.4484	1.3577	-1.7189

Charge = 1, Multiplicity = 2, E_{el} = -659.65302087 Ha

E_{ZPE} = 0.33545566 Ha

10 lowest real freq. (cm^{-1}): 65.0, 99.2, 121.8, 150.2, 199.4, 204.2, 230.6, 241.0, 271.5, 276.8

11.3 SPLET Deprotonated (Anion) Geometries (M06-2X/def2-TZVP)

Deprotonated geometries are only provided for the eight species for which both TZVP single-point energies and optimized anion geometries were obtained.

Table S44: Mol 1 (CAM), SPLET deprotonated anion geometry (M06-2X/ma-def2-SVP)

#	Atom	X (Å)	Y (Å)	Z (Å)
1	O	3.0072	0.2373	0.1212
2	C	-0.0294	-1.2464	-0.4512
3	C	-1.2229	-0.3937	0.0750
4	C	0.7371	0.8710	-0.6044
5	C	0.5671	-0.3314	-1.5405
6	C	1.1223	-1.2561	0.5659
7	C	1.7497	0.2055	0.4952
8	C	-0.6169	1.0162	-0.0012
9	C	-1.6757	-0.7559	1.4927
10	C	-2.4320	-0.5194	-0.8642
11	C	-1.2060	2.1278	0.4639
12	H	-0.3578	-2.2466	-0.7851
13	H	1.1922	1.7866	-1.0008
14	H	-0.0970	-0.1615	-2.4040
15	H	1.5490	-0.6839	-1.8898
16	H	0.8163	-1.5645	1.5783
17	H	1.9212	-1.9345	0.2317
18	H	1.5167	0.7431	1.4643
19	H	-1.9086	-1.8327	1.5617
20	H	-0.9034	-0.5156	2.2346
21	H	-2.5854	-0.1942	1.7610
22	H	-3.2264	0.1830	-0.5651
23	H	-2.1636	-0.2964	-1.9060
24	H	-2.8415	-1.5434	-0.8236
25	H	-2.1864	2.0996	0.9502
26	H	-0.7153	3.1007	0.3806

Charge = -1, Multiplicity = 1, E_{el} = -465.25614553 Ha

E_{ZPE} = 0.22653129 Ha

10 lowest real freq. (cm^{-1}): 73.4, 146.3, 200.1, 230.6, 246.8, 279.5, 309.2, 327.2, 350.9, 413.9

Table S45: Mol 2 (IPO), SPLET deprotonated anion geometry (M06-2X/ma-def2-SVP)

#	Atom	X (Å)	Y (Å)	Z (Å)
1	O	-2.6268	0.5701	-0.6850
2	C	1.3434	-0.1146	-0.3793
3	C	0.9156	1.2313	0.2828
4	C	0.5289	-0.7936	0.7789
5	C	0.8213	0.4702	1.6292
6	C	-0.4866	1.6329	-0.1754
7	C	-0.9142	-0.8299	0.3564
8	C	-1.6003	0.5528	0.1462
9	C	2.8443	-0.3720	-0.2566
10	C	0.8942	-0.3910	-1.8081
11	C	-1.5774	-1.9639	0.1103
12	H	1.6466	2.0587	0.2109
13	H	0.8958	-1.7721	1.1385
14	H	0.0424	0.7906	2.3351
15	H	1.7844	0.4055	2.1543
16	H	-0.5103	1.8012	-1.2642
17	H	-0.7856	2.5838	0.2969
18	H	-1.8830	0.7960	1.2258
19	H	3.4015	0.2799	-0.9507
20	H	3.2254	-0.1847	0.7577
21	H	3.0781	-1.4177	-0.5177
22	H	1.1253	-1.4350	-2.0809
23	H	-0.1870	-0.2457	-1.9405
24	H	1.4290	0.2716	-2.5110
25	H	-2.5907	-1.8878	-0.2948
26	H	-1.1265	-2.9480	0.2818

Charge = -1, Multiplicity = 1, E_{el} = -465.23156616 Ha

E_{ZPE} = 0.22633022 Ha

10 lowest real freq. (cm^{-1}): 88.3, 146.1, 191.3, 217.9, 245.7, 298.1, 312.5, 366.0, 384.7, 400.1

Table S46: Mol 3 (CYM), SPLET deprotonated anion geometry (M06-2X/ma-def2-SVP)

#	Atom	X (Å)	Y (Å)	Z (Å)
1	O	2.4782	-1.3334	0.5279
2	C	2.0553	-0.2463	-0.1128
3	C	0.4990	-0.1097	-0.0995
4	C	2.6205	1.0600	0.5307
5	C	2.4864	-0.2567	-1.6142
6	C	-0.2174	0.9347	-0.6993
7	C	-0.2354	-1.0982	0.5637
8	C	-2.3451	-0.0053	0.0197
9	C	-1.6127	0.9876	-0.6415
10	C	-1.6290	-1.0469	0.6233
11	C	-3.8543	0.0268	0.0539
12	H	3.7199	0.9910	0.5093
13	H	2.3071	1.9926	0.0266
14	H	2.3005	1.0954	1.5838
15	H	3.5855	-0.3267	-1.6441
16	H	2.1652	0.6307	-2.1898
17	H	2.0730	-1.1613	-2.0871
18	H	0.3212	1.7308	-1.2220
19	H	0.3604	-1.8926	1.0228
20	H	-2.1443	1.8188	-1.1169
21	H	-2.1791	-1.8324	1.1531
22	H	-4.2407	-0.3693	1.0048
23	H	-4.2350	1.0517	-0.0670
24	H	-4.2889	-0.5835	-0.7554

Charge = -1, Multiplicity = 1, E_{el} = -464.08915976 Ha

E_{ZPE} = 0.20188262 Ha

10 lowest real freq. (cm^{-1}): 59.9, 92.4, 189.1, 209.1, 252.3, 277.0, 286.3, 342.5, 358.6, 364.2

Table S47: Mol 4 (MYR), SPLET deprotonated anion geometry (M06-2X/ma-def2-SVP)

#	Atom	X (Å)	Y (Å)	Z (Å)
1	O	2.5073	-1.4123	0.2216
2	C	-1.1056	-0.6522	0.2089
3	C	-1.5817	0.8070	-0.0996
4	C	0.0059	-0.3639	-0.8516
5	C	-0.9829	0.6286	-1.5161
6	C	-0.7342	1.8415	0.6539
7	C	1.1160	0.4415	-0.2081
8	C	-2.1060	-1.7006	-0.2815
9	C	-0.6596	-1.0339	1.6156
10	C	0.7398	1.5281	0.4851
11	C	2.5102	-0.1734	-0.2772
12	H	-2.6625	1.0167	-0.0059
13	H	0.4110	-1.2212	-1.4082
14	H	-0.5383	1.5203	-1.9838
15	H	-1.6810	0.1547	-2.2192
16	H	-1.0063	1.8389	1.7274
17	H	-0.9801	2.8545	0.2820
18	H	-2.4327	-1.5357	-1.3177
19	H	-1.6460	-2.7004	-0.2289
20	H	-3.0039	-1.7074	0.3601
21	H	-0.0858	-0.2394	2.1058
22	H	-1.5288	-1.2917	2.2458
23	H	0.0214	-1.8975	1.5475
24	H	1.4821	2.1421	1.0074
25	H	3.2003	0.5649	0.2352
26	H	2.8176	-0.1029	-1.3736

Charge = -1, Multiplicity = 1, E_{el} = -465.22603972 Ha

E_{ZPE} = 0.22605894 Ha

10 lowest real freq. (cm^{-1}): 84.6, 120.6, 199.4, 221.0, 261.3, 271.2, 322.9, 359.9, 387.9, 408.3

Table S48: Mol 6 (CRV), SPLET deprotonated anion geometry (M06-2X/ma-def2-SVP)

#	Atom	X (Å)	Y (Å)	Z (Å)
1	O	-2.1089	-1.8412	-0.5128
2	C	0.9403	0.3213	-0.1688
3	C	0.1140	-0.9731	-0.1314
4	C	-1.3280	-0.7805	-0.6778
5	C	0.3135	1.3359	0.7865
6	C	-1.9116	0.5150	-0.0627
7	C	-1.1807	1.4276	0.5973
8	C	2.4233	0.1024	0.0330
9	C	-3.3946	0.6435	-0.2336
10	C	3.0777	-0.8608	-0.9268
11	C	3.1518	0.7115	0.9806
12	H	0.8305	0.7245	-1.1975
13	H	0.0290	-1.3336	0.9102
14	H	0.5736	-1.7798	-0.7224
15	H	-1.1657	-0.4915	-1.7786
16	H	0.7765	2.3295	0.6429
17	H	0.5433	1.0420	1.8291
18	H	-1.6797	2.3004	1.0414
19	H	-3.6457	0.6735	-1.3078
20	H	-3.7972	1.5436	0.2579
21	H	-3.8649	-0.2693	0.1604
22	H	2.7810	-1.8961	-0.7022
23	H	2.7532	-0.6583	-1.9600
24	H	4.1739	-0.7946	-0.8778
25	H	4.2254	0.5225	1.0691
26	H	2.7090	1.4070	1.6945

Charge = -1, Multiplicity = 1, E_{el} = -465.24736053 Ha

E_{ZPE} = 0.22496239 Ha

10 lowest real freq. (cm^{-1}): 51.6, 87.8, 137.1, 161.0, 185.6, 201.1, 272.8, 298.0, 315.4, 333.9

Table S49: Mol 8 (CAR), SPLET deprotonated anion geometry (M06-2X/ma-def2-SVP)

#	Atom	X (Å)	Y (Å)	Z (Å)
1	O	0.1560	-0.6256	-1.3226
2	C	0.2371	-0.1649	-0.0782
3	C	-0.3929	1.3354	0.0961
4	C	1.7515	0.0368	0.4269
5	C	0.6874	2.1436	0.8301
6	C	2.0103	1.5436	0.3708
7	C	-0.4630	-1.1391	0.9346
8	C	-1.7105	1.4150	0.8897
9	C	2.7557	-0.7897	-0.3795
10	C	-0.6285	1.9178	-1.2976
11	C	-1.7739	-1.6821	0.3782
12	C	-2.8657	0.6101	0.3424
13	C	-2.9010	-0.7130	0.1267
14	C	4.2047	-0.4481	-0.0201
15	C	2.5165	-2.2921	-0.2398
16	C	-4.1290	-1.3558	-0.4677
17	H	1.8204	-0.2701	1.4933
18	H	0.5902	1.9927	1.9219
19	H	0.6023	3.2300	0.6428
20	H	2.2244	1.8406	-0.6717
21	H	2.8636	1.8718	0.9883
22	H	-0.5896	-0.6901	1.9358
23	H	0.2086	-2.0013	1.0744
24	H	-2.0180	2.4765	0.9240
25	H	-1.5235	1.1343	1.9392
26	H	2.5629	-0.5340	-1.4352
27	H	-1.0549	2.9357	-1.2216
28	H	0.3054	1.9587	-1.8748
29	H	-1.3058	1.2630	-1.8615
30	H	-2.1640	-2.4980	1.0181

Table S49 (Mol 8 (CAR), SPLET deprotonated anion geometry (M06-2X/ma-def2-SVP)) – Continued

#	Atom	X (Å)	Y (Å)	Z (Å)
31	H	-1.5202	-2.1306	-0.5994
32	H	-3.7668	1.1754	0.0716
33	H	4.3966	-0.6346	1.0520
34	H	4.9109	-1.0693	-0.5964
35	H	4.4461	0.6062	-0.2235
36	H	2.5932	-2.6094	0.8164
37	H	3.2648	-2.8631	-0.8159
38	H	1.5160	-2.5322	-0.6242
39	H	-4.5089	-2.1599	0.1857
40	H	-3.8802	-1.8228	-1.4352
41	H	-4.9369	-0.6276	-0.6301

Charge = -1, Multiplicity = 1, E_{el} = -661.81284492 Ha

E_{ZPE} = 0.36918202 Ha

10 lowest real freq. (cm^{-1}): 36.6, 79.2, 86.4, 125.5, 173.3, 181.3, 207.9, 227.6, 242.4, 257.9

Table S50: Mol 10 (GLO), SPLET deprotonated anion geometry (M06-2X/ma-def2-SVP)

#	Atom	X (Å)	Y (Å)	Z (Å)
1	O	3.2343	1.0247	0.6521
2	C	-1.1758	-0.0843	0.5558
3	C	-2.3914	0.6156	-0.0065
4	C	-1.2770	1.4163	0.6375
5	C	-0.0546	-0.6688	-0.2736
6	C	1.3135	-0.3653	0.3949
7	C	-0.2851	2.2916	-0.1100
8	C	-0.1012	-2.2172	-0.4785
9	C	2.0597	0.9590	0.0154
10	C	2.1322	-1.6433	0.2023
11	C	1.1534	2.1765	0.4283
12	C	-3.6748	0.4734	0.7863

Table S50 (Mol 10 (GLO), SPLET deprotonated anion geometry (M06-2X/ma-def2-SVP)) – Continued

#	Atom	X (Å)	Y (Å)	Z (Å)
13	C	-2.6357	0.6806	-1.4994
14	C	1.0807	-2.7446	0.3495
15	C	-1.4382	-2.8909	-0.1881
16	C	2.2721	1.0316	-1.5313
17	H	-1.3443	-0.6123	1.5026
18	H	-1.5231	1.8095	1.6314
19	H	-0.0756	-0.2012	-1.2668
20	H	1.1396	-0.2719	1.4846
21	H	-0.6305	3.3377	-0.0285
22	H	-0.2942	2.0639	-1.1870
23	H	0.1449	-2.4084	-1.5381
24	H	2.9667	-1.6912	0.9133
25	H	2.5649	-1.6756	-0.8133
26	H	1.7324	3.0686	0.1353
27	H	1.1375	2.1723	1.5329
28	H	-4.2067	-0.4541	0.5120
29	H	-4.3552	1.3221	0.6007
30	H	-3.4683	0.4358	1.8664
31	H	-1.7114	0.7775	-2.0807
32	H	-3.2778	1.5431	-1.7464
33	H	-3.1535	-0.2308	-1.8446
34	H	1.4123	-3.7444	0.0209
35	H	0.7779	-2.8289	1.4100
36	H	-1.7138	-2.7910	0.8732
37	H	-2.2519	-2.4457	-0.7827
38	H	-1.3946	-3.9673	-0.4201
39	H	2.9306	0.2046	-1.8391
40	H	1.3606	1.0099	-2.1585
41	H	2.8116	1.9696	-1.7347

Charge = -1, Multiplicity = 1, E_{el} = -661.79680867 Ha

Table S50 (Mol 10 (GLO), SPLET deprotonated anion geometry (M06-2X/ma-def2-SVP)) – Continued

#	Atom	X (Å)	Y (Å)	Z (Å)

$E_{\text{ZPE}} = 0.37023525$ Ha

10 lowest real freq. (cm^{-1}): 50.0, 78.9, 114.9, 149.0, 156.6, 185.2, 201.8, 210.8, 222.0, 239.2

Table S51: Mol 11 (DHE), SPLET deprotonated anion geometry (M06-2X/ma-def2-SVP)

#	Atom	X (Å)	Y (Å)	Z (Å)
1	O	-3.9659	1.4827	-1.2616
2	C	1.2405	-1.0683	-0.2395
3	C	0.2864	-2.1125	0.3580
4	C	-1.4801	-0.3866	0.9086
5	C	-1.1817	-1.7224	0.2160
6	C	0.8920	0.3126	0.3031
7	C	2.6833	-1.4078	0.1688
8	C	-0.3391	0.5845	0.7765
9	C	-2.8162	0.2671	0.4895
10	C	1.1193	-1.0498	-1.7735
11	C	3.6907	-0.3819	-0.3446
12	C	1.9462	1.3460	0.2196
13	C	3.2164	1.0236	-0.1008
14	C	-2.8267	0.8860	-0.9478
15	C	-3.9997	-0.6778	0.6479
16	C	1.5618	2.7788	0.4811
17	H	0.5285	-2.2264	1.4309
18	H	0.4790	-3.0898	-0.1195
19	H	-1.5617	-0.5947	1.9979
20	H	-1.4569	-1.6511	-0.8465
21	H	-1.8216	-2.5087	0.6458
22	H	2.9402	-2.4186	-0.1927
23	H	2.7292	-1.4346	1.2712
24	H	-0.5629	1.5964	1.1280

Table S51 (Mol 11 (DHE), SPLET deprotonated anion geometry (M06-2X/ma-def2-SVP)) – Continued

#	Atom	X (Å)	Y (Å)	Z (Å)
25	H	-2.9791	1.1264	1.1690
26	H	1.7406	-0.2557	-2.2128
27	H	1.4366	-2.0185	-2.1976
28	H	0.0825	-0.8510	-2.0787
29	H	3.8745	-0.5346	-1.4245
30	H	4.6688	-0.5342	0.1425
31	H	3.9550	1.8276	-0.1899
32	H	-1.9047	1.5576	-0.9771
33	H	-2.5307	0.0290	-1.6462
34	H	-4.0899	-1.0822	1.6729
35	H	-3.9188	-1.5262	-0.0538
36	H	-4.9092	-0.1222	0.3805
37	H	1.2190	2.9134	1.5186
38	H	0.7316	3.0847	-0.1737
39	H	2.4158	3.4491	0.3134

Charge = -1, Multiplicity = 1, E_{el} = -660.59207944 Ha

E_{ZPE} = 0.34721098 Ha

10 lowest real freq. (cm^{-1}): 35.9, 60.2, 86.6, 120.8, 150.4, 179.7, 200.4, 218.2, 234.9, 241.7

Table S52: Mol 12 (ISO), SPLET deprotonated anion geometry (M06-2X/ma-def2-SVP)

#	Atom	X (Å)	Y (Å)	Z (Å)
1	O	3.5637	-0.1907	1.0763
2	C	0.8702	-1.0201	-0.0554
3	C	0.4207	0.4417	0.0201
4	C	-0.1687	-1.7960	-0.8967
5	C	2.2438	-1.1525	-0.7316
6	C	-2.0202	-0.1819	-0.3852
7	C	-1.0154	0.6255	0.4665
8	C	-1.6078	-1.6510	-0.4084

Table S52 (Mol 12 (ISO), SPLET deprotonated anion geometry (M06-2X/ma-def2-SVP)) – Continued

#	Atom	X (Å)	Y (Å)	Z (Å)
9	C	3.2795	-0.1103	-0.2226
10	C	1.2469	1.4706	-0.2563
11	C	2.6957	1.2694	-0.6368
12	C	0.9568	-1.6145	1.3658
13	C	-3.4488	0.0715	0.0503
14	C	0.8537	2.9285	-0.1982
15	C	-3.9109	1.5064	-0.0157
16	C	-4.2761	-0.8926	0.4745
17	H	0.1222	-2.8607	-0.9164
18	H	-0.1130	-1.4321	-1.9385
19	H	2.1184	-1.0401	-1.8273
20	H	2.6369	-2.1673	-0.5436
21	H	-1.9353	0.2066	-1.4202
22	H	-1.1247	0.2861	1.5135
23	H	-1.2919	1.6852	0.4579
24	H	-2.2914	-2.2243	-1.0574
25	H	-1.7042	-2.0722	0.6071
26	H	4.1802	-0.2549	-0.9042
27	H	2.7950	1.4469	-1.7293
28	H	3.3058	2.0394	-0.1335
29	H	1.8122	-1.1359	1.8687
30	H	0.0304	-1.4756	1.9454
31	H	1.1532	-2.6990	1.2986
32	H	-0.2268	3.1195	-0.1750
33	H	1.2697	3.4541	-1.0736
34	H	1.3058	3.3997	0.6914
35	H	-3.4385	2.1079	0.7761
36	H	-3.6259	1.9645	-0.9762
37	H	-5.0006	1.5828	0.1024
38	H	-5.3020	-0.6593	0.7717

Table S52 (Mol 12 (ISO), SPLET deprotonated anion geometry (M06-2X/ma-def2-SVP)) – Continued

#	Atom	X (Å)	Y (Å)	Z (Å)
39	H	-3.9619	-1.9347	0.5437

Charge = -1, Multiplicity = 1, E_{el} = -660.58947875 Ha

E_{ZPE} = 0.34672486 Ha

10 lowest real freq. (cm^{-1}): 44.5, 61.1, 82.9, 93.9, 128.1, 149.8, 182.0, 188.4, 217.6, 246.8

11.4 RAF Transition State Geometries (M06-2X/def2-TZVP, Water SMD)

Transition state geometries for the RAF mechanism were taken from the NEB-CI converged structures at the "mid-method" level; single-point energies are from M06-2X/def2-TZVP with SMD water solvation.

Table S53: Mol 1 (CAM), RAF transition state, site 10 (M06-2X/ma-def2-SVP, SMD water)

#	Atom	X (Å)	Y (Å)	Z (Å)
1	O	3.2696	0.3196	0.2516
2	C	0.2798	-1.2681	-0.6531
3	C	-0.9519	-0.4679	-0.1326
4	C	1.0347	0.8600	-0.6475
5	C	0.8990	-0.2887	-1.6690
6	C	1.3918	-1.2958	0.4112
7	C	1.8847	0.1760	0.4665
8	C	-0.3826	0.9516	-0.1466
9	C	-1.4385	-0.8928	1.2574
10	C	-2.1254	-0.5914	-1.1180
11	C	-1.0447	2.0700	0.2235
12	H	0.0071	-2.2608	-1.0385
13	H	1.4719	1.8096	-0.9807
14	H	0.2528	-0.0389	-2.5205
15	H	1.8655	-0.6388	-2.0644

Table S53 (Mol 1 (CAM), RAF transition state, site 10 (M06-2X/ma-def2-SVP, SMD water)) – Continued

#	Atom	X (Å)	Y (Å)	Z (Å)
16	H	1.0687	-1.6803	1.3861
17	H	2.2204	-1.9366	0.0711
18	H	1.6873	0.6406	1.4407
19	H	-1.6799	-1.9685	1.2604
20	H	-0.7008	-0.6799	2.0402
21	H	-2.3505	-0.3380	1.5244
22	H	-2.9450	0.0824	-0.8280
23	H	-1.8377	-0.3441	-2.1488
24	H	-2.5132	-1.6215	-1.1064
25	H	-2.0849	2.0317	0.5480
26	H	-0.6046	3.0584	0.0760
27	H	3.5179	-0.1270	-0.5652
28	O	-0.4301	1.8709	2.3854
29	H	0.2383	2.5683	2.2769

Charge = 0, Multiplicity = 2, E_{el} = -541.61648358 Ha

E_{ZPE} = 0.25344618 Ha

10 lowest real freq. (cm^{-1}): 68.2, 92.1, 113.2, 146.3, 188.7, 202.6, 224.0, 241.1, 264.0, 283.9

Table S54: Mol 1 (CAM), RAF transition state, site 7 (M06-2X/ma-def2-SVP, SMD water)

#	Atom	X (Å)	Y (Å)	Z (Å)
1	O	3.3528	0.1827	0.1494
2	C	0.2388	-1.1595	-0.7086
3	C	-0.9263	-0.3965	-0.0044
4	C	1.0975	0.9180	-0.4737
5	C	0.8271	-0.0713	-1.6325
6	C	1.4207	-1.3680	0.2531
7	C	1.9830	0.0632	0.4648
8	C	-0.3234	1.0246	0.0340

Table S54 (Mol 1 (CAM), RAF transition state, site 7 (M06-2X/ma-def2-SVP, SMD water)) – Continued

#	Atom	X (Å)	Y (Å)	Z (Å)
9	C	-1.2987	-0.9797	1.3638
10	C	-2.1790	-0.4344	-0.8912
11	C	-1.0379	2.1820	-0.0118
12	H	-0.1044	-2.0829	-1.1995
13	H	1.5511	1.8856	-0.7189
14	H	0.1208	0.3196	-2.3790
15	H	1.7385	-0.3994	-2.1582
16	H	1.1557	-1.8734	1.1887
17	H	2.1912	-1.9826	-0.2370
18	H	1.8863	0.4102	1.4971
19	H	-1.5481	-2.0485	1.2540
20	H	-0.4950	-0.8738	2.1006
21	H	-2.1897	-0.4719	1.7660
22	H	-2.9923	0.1666	-0.4580
23	H	-1.9818	-0.0549	-1.9035
24	H	-2.5433	-1.4705	-0.9744
25	H	-2.1229	2.1947	0.1085
26	H	-0.5290	3.1346	-0.1609
27	H	3.5074	-0.1256	-0.7499
28	O	0.0482	1.5937	2.0623
29	H	-0.8471	1.7175	2.4193

Charge = 0, Multiplicity = 2, E_{el} = -541.61049410 Ha

E_{ZPE} = 0.25424346 Ha

10 lowest real freq. (cm^{-1}): 59.7, 174.1, 180.9, 193.3, 224.3, 233.3, 248.6, 285.5, 305.1, 311.6

Table S55: Mol 2 (IPO), RAF transition state, site 10 (M06-2X/ma-def2-SVP, SMD water)

#	Atom	X (Å)	Y (Å)	Z (Å)
1	O	-2.6024	0.7852	-0.8085
2	C	1.3370	0.1004	-0.4320

Table S55 (Mol 2 (IPO), RAF transition state, site 10 (M06-2X/ma-def2-SVP, SMD water)) – Continued

#	Atom	X (Å)	Y (Å)	Z (Å)
3	C	0.8656	1.3625	0.3524
4	C	0.5016	-0.7142	0.6334
5	C	0.7503	0.4648	1.6094
6	C	-0.5295	1.7945	-0.0983
7	C	-0.9155	-0.7058	0.1630
8	C	-1.5558	0.6681	0.1248
9	C	2.8389	-0.1366	-0.2880
10	C	0.9516	-0.0394	-1.9001
11	C	-1.6320	-1.8214	-0.1466
12	H	1.5641	2.2130	0.3819
13	H	0.8561	-1.7216	0.8975
14	H	-0.0375	0.6916	2.3414
15	H	1.7029	0.3560	2.1396
16	H	-0.5314	2.0655	-1.1646
17	H	-0.8559	2.6861	0.4567
18	H	-1.9677	0.7703	1.1462
19	H	3.3894	0.6003	-0.8940
20	H	3.1933	-0.0473	0.7472
21	H	3.1087	-1.1388	-0.6546
22	H	1.2340	-1.0375	-2.2711
23	H	-0.1232	0.0871	-2.0857
24	H	1.4917	0.7062	-2.5053
25	H	-2.5636	-1.7492	-0.7045
26	H	-1.1742	-2.8073	-0.0347
27	H	-3.4248	0.5072	-0.3915
28	O	-2.8844	-1.4974	1.5949
29	H	-2.9874	-2.4423	1.7910

Charge = 0, Multiplicity = 2, E_{el} = -541.59155510 Ha

E_{ZPE} = 0.25398371 Ha

10 lowest real freq. (cm^{-1}): 72.8, 105.2, 133.3, 156.2, 185.0, 209.4, 241.3, 245.5, 308.1, 316.1

Table S55 (Mol 2 (IPO), RAF transition state, site 10 (M06-2X/ma-def2-SVP, SMD water)) – Continued

#	Atom	X (Å)	Y (Å)	Z (Å)

Table S56: Mol 2 (IPO), RAF transition state, site 6 (M06-2X/ma-def2-SVP, SMD water)

#	Atom	X (Å)	Y (Å)	Z (Å)
1	O	-2.8383	0.6551	-0.4393
2	C	1.2101	-0.0152	-0.5256
3	C	0.7901	1.4004	-0.0227
4	C	0.4966	-0.5425	0.7826
5	C	0.8239	0.8280	1.4190
6	C	-0.6573	1.7137	-0.4208
7	C	-0.9576	-0.6434	0.4248
8	C	-1.6180	0.7153	0.2484
9	C	2.7214	-0.2328	-0.4739
10	C	0.7036	-0.4856	-1.8846
11	C	-1.5085	-1.8070	-0.0125
12	H	1.4694	2.2307	-0.2691
13	H	0.8880	-1.4629	1.2421
14	H	0.1219	1.2288	2.1624
15	H	1.8311	0.8426	1.8488
16	H	-0.7840	1.6773	-1.5127
17	H	-0.9345	2.7283	-0.1000
18	H	-1.7925	1.0766	1.2731
19	H	3.2053	0.3360	-1.2830
20	H	3.1747	0.0812	0.4750
21	H	2.9581	-1.2972	-0.6263
22	H	0.9413	-1.5516	-2.0315
23	H	-0.3789	-0.3736	-2.0174
24	H	1.2039	0.0841	-2.6832
25	H	-2.4960	-1.8302	-0.4743
26	H	-0.9495	-2.7442	0.0627

Table S56 (Mol 2 (IPO), RAF transition state, site 6 (M06-2X/ma-def2-SVP, SMD water)) – Continued

#	Atom	X (Å)	Y (Å)	Z (Å)
27	H	-3.4746	0.2300	0.1496
28	O	-2.2000	-0.9840	2.1758
29	H	-1.9495	-1.8578	2.5126

Charge = 0, Multiplicity = 2, E_{el} = -541.58981110 Ha

E_{ZPE} = 0.25355868 Ha

10 lowest real freq. (cm^{-1}): 75.6, 113.4, 145.9, 177.9, 191.0, 210.8, 239.1, 274.1, 296.4, 315.5

Table S57: Mol 3 (CYM), RAF transition state, site 5 (M06-2X/ma-def2-SVP, SMD water)

#	Atom	X (Å)	Y (Å)	Z (Å)
1	O	2.2306	-0.3595	1.3870
2	C	1.8945	-0.0818	0.0182
3	C	0.3647	-0.0676	-0.1018
4	C	2.5044	1.2867	-0.2653
5	C	2.5476	-1.1359	-0.8756
6	C	-0.3759	1.0995	0.2380
7	C	-0.3515	-1.2219	-0.4168
8	C	-2.4928	-0.0818	-0.1132
9	C	-1.7939	1.0732	0.1955
10	C	-1.7476	-1.2328	-0.4274
11	C	-3.9973	-0.1157	-0.1584
12	H	3.5978	1.2213	-0.1857
13	H	2.2373	1.6272	-1.2758
14	H	2.1604	2.0274	0.4687
15	H	3.6379	-1.0814	-0.7547
16	H	2.2954	-0.9733	-1.9334
17	H	2.2359	-2.1535	-0.5994
18	H	0.1065	2.0752	0.1935
19	H	0.1721	-2.1370	-0.6948
20	H	-2.3337	1.9881	0.4498

Table S57 (Mol 3 (CYM), RAF transition state, site 5 (M06-2X/ma-def2-SVP, SMD water)) – Continued

#	Atom	X (Å)	Y (Å)	Z (Å)
21	H	-2.2729	-2.1546	-0.6897
22	H	1.9087	-1.2411	1.6123
23	H	-4.3868	-0.9563	0.4325
24	H	-4.4299	0.8115	0.2367
25	H	-4.3545	-0.2432	-1.1915
26	O	-0.1165	1.2276	2.2120
27	H	0.7595	0.7999	2.2391

Charge = 0, Multiplicity = 2, E_{el} = -540.43986952 Ha

E_{ZPE} = 0.22947381 Ha

10 lowest real freq. (cm^{-1}): 48.3, 56.5, 89.1, 123.1, 161.4, 204.4, 221.6, 256.4, 281.8, 301.6

Table S58: Mol 3 (CYM), RAF transition state, site 6 (M06-2X/ma-def2-SVP, SMD water)

#	Atom	X (Å)	Y (Å)	Z (Å)
1	O	2.4038	-1.2549	0.5740
2	C	1.9419	-0.1465	-0.1654
3	C	0.4130	-0.0838	-0.1393
4	C	2.5540	1.1361	0.4126
5	C	2.4180	-0.3687	-1.6011
6	C	-0.2980	1.0451	-0.5574
7	C	-0.3243	-1.1865	0.3666
8	C	-2.4403	-0.0284	-0.0371
9	C	-1.6913	1.0698	-0.5125
10	C	-1.7433	-1.1519	0.3781
11	C	-3.9474	0.0187	-0.0371
12	H	3.6464	1.0191	0.4288
13	H	2.3087	2.0235	-0.1869
14	H	2.2046	1.2984	1.4432
15	H	3.5146	-0.4449	-1.6058
16	H	2.1094	0.4613	-2.2518

Table S58 (Mol 3 (CYM), RAF transition state, site 6 (M06-2X/ma-def2-SVP, SMD water)) – Continued

#	Atom	X (Å)	Y (Å)	Z (Å)
17	H	2.0008	-1.3062	-1.9938
18	H	0.2346	1.9224	-0.9267
19	H	0.1844	-2.1400	0.5040
20	H	-2.2189	1.9628	-0.8588
21	H	-2.2822	-2.0273	0.7454
22	H	2.0334	-1.1804	1.4683
23	H	-4.3739	-0.7706	0.5948
24	H	-4.3200	0.9876	0.3250
25	H	-4.3372	-0.1218	-1.0570
26	O	0.2405	-0.7865	2.2844
27	H	-0.2316	0.0538	2.4055

Charge = 0, Multiplicity = 2, E_{el} = -540.44445650 Ha

E_{ZPE} = 0.22950824 Ha

10 lowest real freq. (cm^{-1}): 47.9, 57.4, 94.1, 124.8, 174.1, 202.7, 218.0, 243.8, 274.0, 286.7

Table S59: Mol 3 (CYM), RAF transition state, site 7 (M06-2X/ma-def2-SVP, SMD water)

#	Atom	X (Å)	Y (Å)	Z (Å)
1	O	2.6793	-0.7520	1.0129
2	C	2.1068	-0.0600	-0.0912
3	C	0.5837	-0.0070	0.0555
4	C	2.7460	1.3218	-0.0687
5	C	2.4653	-0.7800	-1.3982
6	C	-0.1403	1.1918	0.0585
7	C	-0.1348	-1.2165	0.1562
8	C	-2.2655	-0.0213	0.1789
9	C	-1.5295	1.1884	0.0946
10	C	-1.5150	-1.2306	0.2066
11	C	-3.7184	-0.0671	-0.2106
12	H	3.8368	1.2067	-0.1133

Table S59 (Mol 3 (CYM), RAF transition state, site 7 (M06-2X/ma-def2-SVP, SMD water)) – Continued

#	Atom	X (Å)	Y (Å)	Z (Å)
13	H	2.4217	1.9228	-0.9288
14	H	2.4924	1.8494	0.8606
15	H	3.5586	-0.8326	-1.4985
16	H	2.0495	-0.2480	-2.2659
17	H	2.0673	-1.8059	-1.4099
18	H	0.3796	2.1491	0.0100
19	H	0.3947	-2.1737	0.1572
20	H	-2.0790	2.1322	0.0863
21	H	-2.0554	-2.1774	0.2705
22	H	2.2732	-1.6217	1.0932
23	H	-4.2940	-0.7068	0.4689
24	H	-4.1594	0.9376	-0.2063
25	H	-3.7938	-0.4757	-1.2295
26	O	-2.6375	0.1298	2.1775
27	H	-1.7324	0.1465	2.5333

Charge = 0, Multiplicity = 2, E_{el} = -540.43949663 Ha

E_{ZPE} = 0.22866701 Ha

10 lowest real freq. (cm^{-1}): 40.7, 71.9, 120.0, 167.0, 171.1, 195.2, 222.3, 250.1, 254.0, 262.7

Table S60: Mol 4 (MYR), RAF transition state, site 6 (M06-2X/ma-def2-SVP, SMD water)

#	Atom	X (Å)	Y (Å)	Z (Å)
1	O	3.1413	-1.0492	-0.5127
2	C	-1.1141	-0.6284	0.0515
3	C	-1.4489	0.8093	-0.4479
4	C	0.1953	-0.4824	-0.7912
5	C	-0.6099	0.4930	-1.7091
6	C	-0.7070	1.8589	0.3916
7	C	1.2346	0.4105	-0.1506
8	C	-2.0507	-1.6816	-0.5458

Table S60 (Mol 4 (MYR), RAF transition state, site 6 (M06-2X/ma-def2-SVP, SMD water)) – Continued

#	Atom	X (Å)	Y (Å)	Z (Å)
9	C	-1.0355	-0.8714	1.5516
10	C	0.7659	1.5495	0.4508
11	C	2.6519	0.2537	-0.6432
12	H	-2.5111	1.0701	-0.5755
13	H	0.6421	-1.3883	-1.2237
14	H	-0.0469	1.3219	-2.1635
15	H	-1.1801	-0.0220	-2.4890
16	H	-1.1495	1.9616	1.3980
17	H	-0.8174	2.8520	-0.0810
18	H	-2.1964	-1.5753	-1.6281
19	H	-1.6539	-2.6911	-0.3582
20	H	-3.0402	-1.6162	-0.0669
21	H	-0.4144	-0.1344	2.0699
22	H	-2.0435	-0.8146	1.9915
23	H	-0.6303	-1.8742	1.7576
24	H	1.4628	2.2713	0.8820
25	H	3.2954	0.9950	-0.1408
26	H	2.6436	0.4808	-1.7234
27	H	3.0797	-1.2565	0.4305
28	O	2.1243	-0.1316	1.8104
29	H	1.4129	-0.1105	2.4653

Charge = 0, Multiplicity = 2, E_{el} = -541.58976400 Ha

E_{ZPE} = 0.25419723 Ha

10 lowest real freq. (cm^{-1}): 57.3, 95.7, 140.5, 156.3, 195.6, 206.3, 234.7, 258.9, 272.0, 291.3

Table S61: Mol 4 (MYR), RAF transition state, site 9 (M06-2X/ma-def2-SVP, SMD water)

#	Atom	X (Å)	Y (Å)	Z (Å)
1	O	2.8426	-1.1156	0.4587
2	C	-1.0703	-0.7322	-0.0923

Table S61 (Mol 4 (MYR), RAF transition state, site 9 (M06-2X/ma-def2-SVP, SMD water)) – Continued

#	Atom	X (Å)	Y (Å)	Z (Å)
3	C	-1.5006	0.7218	-0.4596
4	C	0.1579	-0.4626	-1.0300
5	C	-0.7618	0.5321	-1.8049
6	C	-0.7340	1.7585	0.3792
7	C	1.1963	0.3982	-0.3630
8	C	-2.0093	-1.7782	-0.6958
9	C	-0.8140	-1.0867	1.3647
10	C	0.7539	1.5163	0.2876
11	C	2.6417	-0.0170	-0.3949
12	H	-2.5802	0.9390	-0.4791
13	H	0.6021	-1.3227	-1.5509
14	H	-0.2726	1.4183	-2.2344
15	H	-1.3702	0.0437	-2.5726
16	H	-1.0384	1.7053	1.4384
17	H	-0.9790	2.7777	0.0343
18	H	-2.2396	-1.6044	-1.7546
19	H	-1.5624	-2.7803	-0.6091
20	H	-2.9617	-1.7891	-0.1435
21	H	-0.1571	-0.3750	1.8763
22	H	-1.7703	-1.1371	1.9107
23	H	-0.3429	-2.0802	1.4273
24	H	1.4485	2.3215	0.5391
25	H	3.2848	0.8353	-0.1156
26	H	2.9193	-0.3443	-1.4080
27	H	2.6316	-0.7986	1.3512
28	O	1.6556	0.7905	2.2238
29	H	2.0299	1.6658	2.4169

Charge = 0, Multiplicity = 2, E_{el} = -541.59002197 Ha

E_{ZPE} = 0.25449271 Ha

10 lowest real freq. (cm^{-1}): 81.2, 113.5, 145.1, 181.8, 190.6, 204.2, 233.6, 258.9, 263.7, 275.2

Table S61 (Mol 4 (MYR), RAF transition state, site 9 (M06-2X/ma-def2-SVP, SMD water)) – Continued

#	Atom	X (Å)	Y (Å)	Z (Å)

Table S62: Mol 5 (VER), RAF transition state, site 5 (M06-2X/ma-def2-SVP, SMD water)

#	Atom	X (Å)	Y (Å)	Z (Å)
1	O	1.4326	2.8247	-0.4460
2	C	-1.0686	0.3397	-0.4609
3	C	-0.3211	-0.5200	0.6179
4	C	-0.4830	1.5718	0.3199
5	C	-0.4799	0.6794	1.5841
6	C	1.1327	-0.6501	0.2271
7	C	-0.7180	0.1704	-1.9336
8	C	-2.5814	0.2083	-0.2926
9	C	0.9773	1.7763	-0.0454
10	C	1.7948	0.5327	0.0200
11	C	1.6502	-1.9382	-0.3327
12	H	-0.7519	-1.4909	0.9017
13	H	-1.0239	2.5261	0.2905
14	H	-1.4369	0.6925	2.1152
15	H	0.3366	0.8280	2.2998
16	H	-1.0296	-0.8261	-2.2858
17	H	-1.2561	0.9181	-2.5348
18	H	0.3543	0.2879	-2.1431
19	H	-2.9022	-0.7830	-0.6489
20	H	-2.9042	0.3042	0.7510
21	H	-3.1070	0.9700	-0.8865
22	H	2.8282	0.5869	-0.3309
23	H	1.2121	-2.0631	-1.3394
24	H	1.3651	-2.7980	0.2846
25	H	2.7445	-1.9166	-0.4393
26	O	1.6465	-1.2068	2.2787

Table S62 (Mol 5 (VER), RAF transition state, site 5 (M06-2X/ma-def2-SVP, SMD water)) – Continued

#	Atom	X (Å)	Y (Å)	Z (Å)
27	H	2.5887	-1.0244	2.4294

Charge = 0, Multiplicity = 2, E_{el} = -540.38915364 Ha

E_{ZPE} = 0.22912489 Ha

10 lowest real freq. (cm^{-1}): 96.7, 119.8, 133.7, 164.7, 175.9, 189.2, 228.6, 230.2, 240.4, 281.5

Table S63: Mol 6 (CRV), RAF transition state, site 10 (M06-2X/ma-def2-SVP, SMD water)

#	Atom	X (Å)	Y (Å)	Z (Å)
1	O	-2.3472	-2.0046	-0.0772
2	C	0.6250	0.2927	-0.4494
3	C	-0.1412	-0.9977	-0.1233
4	C	-1.6037	-0.8946	-0.5466
5	C	0.0190	1.4370	0.3617
6	C	-2.2363	0.4219	-0.1340
7	C	-1.4822	1.4535	0.2751
8	C	2.1147	0.1279	-0.2739
9	C	-3.7320	0.5035	-0.2511
10	C	2.7742	-0.9760	-1.0524
11	C	2.8366	0.9009	0.5811
12	H	0.4619	0.5057	-1.5255
13	H	-0.0748	-1.1585	0.9670
14	H	0.3002	-1.8776	-0.6131
15	H	-1.6700	-0.9724	-1.6471
16	H	0.4159	2.4054	0.0153
17	H	0.3255	1.3290	1.4170
18	H	-1.9832	2.3826	0.5645
19	H	-4.0508	0.2591	-1.2772
20	H	-4.0943	1.5095	-0.0019
21	H	-4.2269	-0.2251	0.4072
22	H	2.6133	-1.9507	-0.5642

Table S63 (Mol 6 (CRV), RAF transition state, site 10 (M06-2X/ma-def2-SVP, SMD water)) – Continued

#	Atom	X (Å)	Y (Å)	Z (Å)
23	H	2.3472	-1.0520	-2.0644
24	H	3.8565	-0.8136	-1.1397
25	H	-2.2932	-2.0229	0.8855
26	H	3.9204	0.7742	0.6503
27	H	2.4145	1.7642	1.0898
28	O	2.0689	-0.2688	2.2328
29	H	2.8421	-0.8526	2.2933

Charge = 0, Multiplicity = 2, E_{el} = -541.60975421 Ha

E_{ZPE} = 0.25204604 Ha

10 lowest real freq. (cm^{-1}): 49.4, 73.4, 101.7, 109.3, 137.2, 158.6, 167.1, 188.2, 206.8, 278.4

Table S64: Mol 6 (CRV), RAF transition state, site 6 (M06-2X/ma-def2-SVP, SMD water)

#	Atom	X (Å)	Y (Å)	Z (Å)
1	O	-2.1434	-1.9412	-0.0555
2	C	0.9551	0.1706	-0.3458
3	C	0.1373	-1.1109	-0.1480
4	C	-1.3213	-0.9139	-0.5666
5	C	0.4066	1.2406	0.5957
6	C	-1.8731	0.4386	-0.1627
7	C	-1.0897	1.3300	0.5251
8	C	2.4496	-0.0502	-0.2358
9	C	-3.3135	0.7087	-0.4592
10	C	3.0271	-1.0696	-1.1842
11	C	3.2294	0.6254	0.6144
12	H	0.7745	0.5114	-1.3856
13	H	0.1651	-1.3894	0.9191
14	H	0.5443	-1.9550	-0.7211
15	H	-1.3999	-0.9998	-1.6668
16	H	0.8321	2.2256	0.3467

Table S64 (Mol 6 (CRV), RAF transition state, site 6 (M06-2X/ma-def2-SVP, SMD water)) – Continued

#	Atom	X (Å)	Y (Å)	Z (Å)
17	H	0.6909	1.0201	1.6370
18	H	-1.5421	2.2770	0.8373
19	H	-3.4843	0.6775	-1.5480
20	H	-3.6201	1.6974	-0.0930
21	H	-3.9613	-0.0642	-0.0219
22	H	2.7540	-2.0929	-0.8846
23	H	2.6406	-0.9174	-2.2045
24	H	4.1221	-1.0075	-1.2134
25	H	-2.1011	-1.8670	0.9098
26	H	4.3079	0.4548	0.6355
27	H	2.8365	1.3656	1.3123
28	O	-1.5798	0.1279	2.1695
29	H	-2.4436	0.5079	2.3941

Charge = 0, Multiplicity = 2, E_{el} = -541.61194229 Ha

E_{ZPE} = 0.25302517 Ha

10 lowest real freq. (cm^{-1}): 40.8, 81.0, 118.1, 138.5, 142.9, 156.1, 193.8, 200.5, 272.7, 284.4

Table S65: Mol 6 (CRV), RAF transition state, site 7 (M06-2X/ma-def2-SVP, SMD water)

#	Atom	X (Å)	Y (Å)	Z (Å)
1	O	-2.3831	-1.9558	-0.4655
2	C	0.6644	0.2774	-0.3412
3	C	-0.1656	-1.0091	-0.2527
4	C	-1.5877	-0.8240	-0.7678
5	C	0.0092	1.3667	0.5065
6	C	-2.2204	0.4595	-0.2631
7	C	-1.4807	1.4263	0.3009
8	C	2.1074	-0.0168	-0.0004
9	C	-3.7046	0.5809	-0.4631
10	C	2.8802	-0.8609	-0.9777

Table S65 (Mol 6 (CRV), RAF transition state, site 7 (M06-2X/ma-def2-SVP, SMD water)) – Continued

#	Atom	X (Å)	Y (Å)	Z (Å)
11	C	2.8033	0.6286	1.1153
12	H	0.6511	0.6177	-1.3961
13	H	-0.2007	-1.3063	0.8106
14	H	0.3096	-1.8350	-0.8028
15	H	-1.5802	-0.7865	-1.8723
16	H	0.4504	2.3485	0.2676
17	H	0.2199	1.1810	1.5758
18	H	-1.9862	2.3355	0.6412
19	H	-3.9640	0.4330	-1.5237
20	H	-4.0708	1.5651	-0.1436
21	H	-4.2430	-0.1978	0.0963
22	H	2.7410	-1.9341	-0.7780
23	H	2.5287	-0.6657	-2.0044
24	H	3.9560	-0.6446	-0.9346
25	H	-2.3583	-2.1001	0.4879
26	H	3.8999	0.6027	1.1037
27	H	2.3988	1.5428	1.5646
28	O	2.2016	-0.5623	1.5655
29	H	2.1239	-0.6668	2.9512

Charge = 0, Multiplicity = 2, E_{el} = -541.54787509 Ha

E_{ZPE} = 0.24761221 Ha

10 lowest real freq. (cm^{-1}): 44.6, 79.2, 119.9, 130.0, 162.1, 172.2, 184.5, 247.1, 285.8, 291.8

Table S66: Mol 8 (CAR), RAF transition state, site 11 (M06-2X/ma-def2-SVP, SMD water)

#	Atom	X (Å)	Y (Å)	Z (Å)
1	O	0.1237	-0.6365	-1.3663
2	C	0.3045	-0.0944	-0.0660
3	C	-0.2860	1.3753	0.0471
4	C	1.8431	0.0643	0.1923

Table S66 (Mol 8 (CAR), RAF transition state, site 11 (M06-2X/ma-def2-SVP, SMD water)) – Continued

#	Atom	X (Å)	Y (Å)	Z (Å)
5	C	0.8270	2.1510	0.7704
6	C	2.1157	1.5741	0.2005
7	C	-0.2996	-1.0525	0.9785
8	C	-1.6006	1.4547	0.8406
9	C	2.7590	-0.7492	-0.7326
10	C	-0.5049	1.9867	-1.3446
11	C	-1.6848	-1.6175	0.6345
12	C	-2.7235	0.6543	0.2369
13	C	-2.7252	-0.6959	0.0556
14	C	4.2268	-0.3937	-0.4843
15	C	2.5560	-2.2566	-0.5721
16	C	-3.8809	-1.3736	-0.6186
17	H	2.0225	-0.2926	1.2249
18	H	0.7808	1.9433	1.8538
19	H	0.7229	3.2394	0.6405
20	H	2.2911	1.9413	-0.8245
21	H	3.0015	1.8370	0.7954
22	H	-0.3069	-0.5548	1.9586
23	H	0.3816	-1.9096	1.0859
24	H	-1.9036	2.5121	0.8955
25	H	-1.4449	1.1317	1.8785
26	H	2.5025	-0.4782	-1.7700
27	H	-0.7895	3.0444	-1.2353
28	H	0.3952	1.9274	-1.9695
29	H	-1.3135	1.4829	-1.8946
30	H	-2.1369	-2.0506	1.5456
31	H	-1.5624	-2.4664	-0.0589
32	H	-0.8154	-0.6161	-1.5821
33	H	-3.5361	1.2109	-0.2440

Table S66 (Mol 8 (CAR), RAF transition state, site 11 (M06-2X/ma-def2-SVP, SMD water)) – Continued

#	Atom	X (Å)	Y (Å)	Z (Å)
34	H	4.5000	-0.5782	0.5684
35	H	4.8853	-1.0117	-1.1124
36	H	4.4449	0.6594	-0.7106
37	H	2.7696	-2.5680	0.4647
38	H	3.2416	-2.8106	-1.2306
39	H	1.5304	-2.5534	-0.8260
40	H	-4.4466	-1.9524	0.1294
41	H	-3.5374	-2.0819	-1.3890
42	H	-4.5649	-0.6502	-1.0805
43	O	-3.8217	0.2197	2.1102
44	H	-4.3404	1.0344	2.0045

Charge = 0, Multiplicity = 2, E_{el} = -738.16469843 Ha

E_{ZPE} = 0.39679774 Ha

10 lowest real freq. (cm^{-1}): 38.6, 82.0, 94.9, 99.1, 117.7, 128.8, 173.5, 187.8, 212.4, 217.2

Table S67: Mol 9 (CAO), RAF transition state, site 15 (M06-2X/ma-def2-SVP, SMD water)

#	Atom	X (Å)	Y (Å)	Z (Å)
1	O	3.2498	-1.4495	-0.6517
2	C	-0.8270	-0.6201	-0.3284
3	C	-2.3628	-0.3684	-0.4570
4	C	-0.6968	0.8210	0.2454
5	C	-2.0500	1.1460	-0.4424
6	C	2.0832	-1.2454	0.1377
7	C	-0.3569	-1.8429	0.4627
8	C	2.2939	-0.5288	-1.1304
9	C	1.0543	-2.3522	0.1292
10	C	0.4487	1.7650	0.0153
11	C	2.5915	0.9395	-1.2285

Table S67 (Mol 9 (CAO), RAF transition state, site 15 (M06-2X/ma-def2-SVP, SMD water)) – Continued

#	Atom	X (Å)	Y (Å)	Z (Å)
12	C	-3.0215	-0.9232	-1.7106
13	C	-3.1436	-0.7867	0.7884
14	C	1.2692	1.7252	-1.2581
15	C	2.3372	-0.6105	1.4801
16	C	0.6216	2.7824	0.9164
17	H	-0.4203	-0.6529	-1.3537
18	H	-0.8549	0.7774	1.3330
19	H	-2.7364	1.8129	0.1004
20	H	-1.8993	1.5338	-1.4626
21	H	-1.0614	-2.6694	0.2692
22	H	-0.4397	-1.6277	1.5411
23	H	1.7331	-0.9098	-1.9970
24	H	1.3445	-3.1374	0.8460
25	H	1.0607	-2.8129	-0.8711
26	H	3.1429	1.1400	-2.1607
27	H	3.2303	1.2750	-0.4003
28	H	-3.0250	-2.0254	-1.6958
29	H	-4.0676	-0.5867	-1.7882
30	H	-2.4898	-0.5957	-2.6169
31	H	-4.1733	-0.4012	0.7333
32	H	-3.2012	-1.8826	0.8739
33	H	-2.6907	-0.4004	1.7131
34	H	0.6355	1.3014	-2.0567
35	H	1.4872	2.7592	-1.5602
36	H	2.8067	-1.3523	2.1437
37	H	3.0183	0.2464	1.3974
38	H	1.3981	-0.2717	1.9373
39	H	-0.1857	3.0956	1.5747
40	H	1.4541	3.4815	0.7976

Table S67 (Mol 9 (CAO), RAF transition state, site 15 (M06-2X/ma-def2-SVP, SMD water)) – Continued

#	Atom	X (Å)	Y (Å)	Z (Å)
41	O	0.7470	1.8244	2.8557
42	H	1.6960	1.6272	2.8788

Charge = 0, Multiplicity = 2, E_{el} = -736.89548740 Ha

E_{ZPE} = 0.37330693 Ha

10 lowest real freq. (cm^{-1}): 63.3, 78.3, 91.2, 106.2, 119.6, 145.2, 165.7, 184.1, 189.3, 200.2

Table S68: Mol 9 (CAO), RAF transition state, site 9 (M06-2X/ma-def2-SVP, SMD water)

#	Atom	X (Å)	Y (Å)	Z (Å)
1	O	3.1740	-1.4857	-0.8261
2	C	-0.8118	-0.5812	-0.2877
3	C	-2.3481	-0.3524	-0.4584
4	C	-0.7270	0.8389	0.3503
5	C	-2.0684	1.1578	-0.3526
6	C	2.1088	-1.4109	0.1179
7	C	-0.3535	-1.8536	0.4352
8	C	2.2188	-0.4605	-1.0028
9	C	1.0014	-2.4320	-0.0028
10	C	0.3168	1.9164	0.1303
11	C	2.5728	0.9954	-0.8913
12	C	-2.9395	-0.8413	-1.7714
13	C	-3.1744	-0.8347	0.7319
14	C	1.3166	1.8813	-1.0063
15	C	2.5700	-1.1162	1.5220
16	C	0.1110	3.0885	0.7886
17	H	-0.3860	-0.5471	-1.3009
18	H	-0.8820	0.7504	1.4362
19	H	-2.7870	1.7894	0.1904
20	H	-1.8840	1.6027	-1.3436

Table S68 (Mol 9 (CAO), RAF transition state, site 9 (M06-2X/ma-def2-SVP, SMD water)) – Continued

#	**Atom**	**X (Å)**	**Y (Å)**	**Z (Å)**
21	H	-1.1127	-2.6324	0.2552
22	H	-0.3596	-1.6761	1.5236
23	H	1.5690	-0.6687	-1.8647
24	H	1.2351	-3.3228	0.6028
25	H	0.9470	-2.7595	-1.0534
26	H	3.2311	1.2547	-1.7365
27	H	3.1125	1.2036	0.0419
28	H	-2.9367	-1.9417	-1.8186
29	H	-3.9822	-0.5050	-1.8823
30	H	-2.3645	-0.4596	-2.6299
31	H	-4.1953	-0.4289	0.6604
32	H	-3.2534	-1.9314	0.7500
33	H	-2.7473	-0.5089	1.6931
34	H	0.7535	1.5619	-1.9038
35	H	1.6474	2.9161	-1.1889
36	H	3.0794	-2.0126	1.9078
37	H	3.2748	-0.2785	1.5472
38	H	1.7312	-0.8641	2.1826
39	H	-0.6859	3.1855	1.5299
40	H	0.7266	3.9696	0.5880
41	O	1.4313	1.4832	2.0402
42	H	1.8702	2.3106	2.2966

Charge = 0, Multiplicity = 2, E_{el} = -736.89177118 Ha

E_{ZPE} = 0.37342076 Ha

10 lowest real freq. (cm^{-1}): 48.4, 78.8, 114.1, 122.2, 151.4, 160.1, 188.8, 192.8, 205.4, 223.3

Table S69: Mol 11 (DHE), RAF transition state, site 11 (M06-2X/ma-def2-SVP, SMD water)

#	Atom	X (Å)	Y (Å)	Z (Å)
1	O	-3.9901	1.5437	-1.2274
2	C	1.2129	-1.0340	-0.2575
3	C	0.2554	-2.0778	0.3340
4	C	-1.5166	-0.3612	0.8709
5	C	-1.2133	-1.6952	0.1776
6	C	0.8500	0.3452	0.2797
7	C	2.6524	-1.3715	0.1645
8	C	-0.3802	0.6181	0.7451
9	C	-2.8506	0.2898	0.4468
10	C	1.1132	-1.0119	-1.7946
11	C	3.6619	-0.3515	-0.3519
12	C	1.9127	1.3861	0.2370
13	C	3.1745	1.0479	-0.2071
14	C	-2.8030	0.8178	-0.9829
15	C	-4.0398	-0.6437	0.6533
16	C	1.5164	2.8389	0.2483
17	H	0.4883	-2.1866	1.4074
18	H	0.4506	-3.0557	-0.1356
19	H	-1.6220	-0.5692	1.9541
20	H	-1.4697	-1.6360	-0.8919
21	H	-1.8525	-2.4849	0.6000
22	H	2.9086	-2.3791	-0.1993
23	H	2.6984	-1.3871	1.2638
24	H	-0.6005	1.6268	1.1124
25	H	-2.9935	1.1761	1.0911
26	H	1.7282	-0.2084	-2.2266
27	H	1.4568	-1.9714	-2.2153
28	H	0.0795	-0.8373	-2.1250
29	H	3.9154	-0.5358	-1.4132
30	H	4.6164	-0.4451	0.1919

Table S69 (Mol 11 (DHE), RAF transition state, site 11 (M06-2X/ma-def2-SVP, SMD water)) – Continued

#	Atom	X (Å)	Y (Å)	Z (Å)
31	H	3.8861	1.8572	-0.3922
32	H	-1.9140	1.4620	-1.1106
33	H	-2.7106	-0.0256	-1.6933
34	H	-4.0654	-1.0222	1.6865
35	H	-3.9852	-1.5092	-0.0253
36	H	-4.9812	-0.1171	0.4513
37	H	0.8881	3.1012	1.1091
38	H	0.9460	3.0697	-0.6656
39	H	2.4081	3.4765	0.2845
40	H	-3.9873	1.8692	-2.1319
41	O	2.5423	1.1650	2.2314
42	H	1.6132	1.2262	2.5064

Charge = 0, Multiplicity = 2, E_{el} = -736.95748546 Ha

E_{ZPE} = 0.37306568 Ha

10 lowest real freq. (cm^{-1}): 34.6, 55.8, 73.2, 106.9, 127.3, 146.0, 181.3, 195.8, 206.5, 232.1

Table S70: Mol 11 (DHE), RAF transition state, site 12 (M06-2X/ma-def2-SVP, SMD water)

#	Atom	X (Å)	Y (Å)	Z (Å)
1	O	-4.0597	1.5711	-1.2315
2	C	1.1674	-1.0233	-0.3084
3	C	0.1999	-2.0651	0.2724
4	C	-1.5592	-0.3454	0.8252
5	C	-1.2648	-1.6709	0.1134
6	C	0.8094	0.3619	0.2204
7	C	2.6000	-1.3687	0.1309
8	C	-0.4203	0.6328	0.7043
9	C	-2.8986	0.3069	0.4170
10	C	1.0847	-1.0102	-1.8450

Table S70 (Mol 11 (DHE), RAF transition state, site 12 (M06-2X/ma-def2-SVP, SMD water)) – Continued

#	Atom	X (Å)	Y (Å)	Z (Å)
11	C	3.6201	-0.3410	-0.3444
12	C	1.8598	1.4004	0.1595
13	C	3.1480	1.0598	-0.0991
14	C	-2.8783	0.8261	-1.0167
15	C	-4.0875	-0.6236	0.6445
16	C	1.4896	2.8353	0.4236
17	H	0.4233	-2.1876	1.3469
18	H	0.3915	-3.0402	-0.2047
19	H	-1.6599	-0.5666	1.9063
20	H	-1.5138	-1.5924	-0.9568
21	H	-1.9128	-2.4616	0.5193
22	H	2.8607	-2.3735	-0.2402
23	H	2.6313	-1.4122	1.2322
24	H	-0.6362	1.6433	1.0655
25	H	-3.0315	1.1967	1.0583
26	H	1.7174	-0.2204	-2.2748
27	H	1.4154	-1.9792	-2.2529
28	H	0.0571	-0.8224	-2.1867
29	H	3.8329	-0.4660	-1.4215
30	H	4.5796	-0.4876	0.1731
31	H	3.8919	1.8591	-0.1680
32	H	-1.9821	1.4538	-1.1737
33	H	-2.8222	-0.0224	-1.7245
34	H	-4.1008	-1.0011	1.6782
35	H	-4.0473	-1.4901	-0.0343
36	H	-5.0298	-0.0936	0.4551
37	H	1.1216	2.9722	1.4528
38	H	0.6907	3.1713	-0.2550
39	H	2.3618	3.4883	0.2976

Table S70 (Mol 11 (DHE), RAF transition state, site 12 (M06-2X/ma-def2-SVP, SMD water)) – Continued

#	Atom	X (Å)	Y (Å)	Z (Å)
40	H	-4.0833	1.8834	-2.1404
41	O	3.4857	1.0684	2.2787
42	H	2.5482	0.9341	2.5037

Charge = 0, Multiplicity = 2, E_{el} = -736.96278985 Ha

E_{ZPE} = 0.37277569 Ha

10 lowest real freq. (cm^{-1}): 35.5, 55.1, 68.3, 73.8, 100.5, 138.4, 168.0, 177.1, 204.4, 211.2

Table S71: Mol 11 (DHE), RAF transition state, site 7 (M06-2X/ma-def2-SVP, SMD water)

#	Atom	X (Å)	Y (Å)	Z (Å)
1	O	-4.1000	1.5234	-1.2644
2	C	1.3166	-0.9743	-0.3282
3	C	0.2969	-2.0211	0.1433
4	C	-1.4666	-0.3198	0.6757
5	C	-1.1448	-1.5965	-0.1032
6	C	0.9344	0.4065	0.1847
7	C	2.7024	-1.3538	0.2190
8	C	-0.3380	0.6801	0.6069
9	C	-2.8288	0.3073	0.3316
10	C	1.3610	-0.9158	-1.8693
11	C	3.7747	-0.3361	-0.1507
12	C	2.0014	1.4281	0.2213
13	C	3.2939	1.0692	0.0589
14	C	-2.9212	0.7595	-1.1178
15	C	-3.9898	-0.6170	0.6882
16	C	1.6336	2.8678	0.4932
17	H	0.4360	-2.1824	1.2272
18	H	0.5099	-2.9726	-0.3667
19	H	-1.5266	-0.5929	1.7444

Table S71 (Mol 11 (DHE), RAF transition state, site 7 (M06-2X/ma-def2-SVP, SMD water)) – Continued

#	Atom	X (Å)	Y (Å)	Z (Å)
20	H	-1.3116	-1.4469	-1.1828
21	H	-1.8272	-2.3978	0.2096
22	H	2.9781	-2.3523	-0.1499
23	H	2.6304	-1.4306	1.3170
24	H	-0.5989	1.7056	0.8738
25	H	-2.9262	1.2202	0.9438
26	H	2.0387	-0.1247	-2.2207
27	H	1.7068	-1.8804	-2.2796
28	H	0.3667	-0.6961	-2.2840
29	H	4.0856	-0.4624	-1.2043
30	H	4.6884	-0.5101	0.4408
31	H	4.0564	1.8537	0.0868
32	H	-2.0268	1.3543	-1.3840
33	H	-2.9496	-0.1226	-1.7845
34	H	-3.9368	-0.9106	1.7462
35	H	-3.9733	-1.5337	0.0762
36	H	-4.9481	-0.1109	0.5155
37	H	1.2192	2.9824	1.5075
38	H	0.8721	3.2349	-0.2118
39	H	2.5154	3.5167	0.4099
40	H	-4.2276	1.7362	-2.1920
41	O	0.3363	0.5121	2.7253
42	H	1.2873	0.7030	2.6474

Charge = 0, Multiplicity = 2, E_{el} = -736.96250889 Ha

E_{ZPE} = 0.37299245 Ha

10 lowest real freq. (cm^{-1}): 32.0, 59.9, 69.4, 84.1, 117.7, 136.1, 164.3, 184.1, 212.2, 215.1

Table S72: Mol 12 (ISO), RAF transition state, site 12 (M06-2X/ma-def2-SVP, SMD water)

#	Atom	X (Å)	Y (Å)	Z (Å)
1	O	3.9421	-0.0400	0.9061
2	C	1.1918	-1.0004	-0.0646
3	C	0.7411	0.4593	-0.0496
4	C	0.1536	-1.8190	-0.8660
5	C	2.5468	-1.1644	-0.7724
6	C	-1.6919	-0.1770	-0.3948
7	C	-0.6803	0.6476	0.4241
8	C	-1.2897	-1.6511	-0.3980
9	C	3.5647	-0.0755	-0.4596
10	C	1.5441	1.4816	-0.4021
11	C	2.9862	1.2833	-0.8117
12	C	1.2629	-1.5427	1.3780
13	C	-3.1265	0.0763	0.0278
14	C	1.1380	2.9339	-0.4262
15	C	-3.5311	1.5180	0.1964
16	C	-4.0915	-0.8884	-0.0190
17	H	0.4409	-2.8841	-0.8361
18	H	0.2133	-1.5048	-1.9226
19	H	2.3872	-1.1430	-1.8639
20	H	2.9638	-2.1610	-0.5418
21	H	-1.6329	0.1936	-1.4392
22	H	-0.7705	0.3280	1.4741
23	H	-0.9531	1.7068	0.3995
24	H	-1.9421	-2.2256	-1.0730
25	H	-1.4387	-2.0578	0.6150
26	H	4.4659	-0.2452	-1.0779
27	H	3.0739	1.4476	-1.9006
28	H	3.6093	2.0583	-0.3363
29	H	1.9301	-0.9245	1.9921
30	H	0.2756	-1.5569	1.8592

Table S72 (Mol 12 (ISO), RAF transition state, site 12 (M06-2X/ma-def2-SVP, SMD water)) – Continued

#	Atom	X (Å)	Y (Å)	Z (Å)
31	H	1.6412	-2.5783	1.3704
32	H	0.0556	3.0990	-0.4115
33	H	1.5364	3.4171	-1.3319
34	H	1.5758	3.4674	0.4336
35	H	4.2242	-0.9202	1.1758
36	H	-3.0010	1.9873	1.0356
37	H	-3.2726	2.0853	-0.7117
38	H	-4.6113	1.6112	0.3660
39	H	-5.1387	-0.6313	0.1537
40	H	-3.8610	-1.9380	-0.1972
41	O	-2.8732	-0.6121	2.0446
42	H	-3.5587	-0.0604	2.4552

Charge = 0, Multiplicity = 2, E_{el} = -736.95049797 Ha

E_{ZPE} = 0.37362393 Ha

10 lowest real freq. (cm^{-1}): 51.4, 83.4, 88.7, 121.6, 139.4, 145.1, 161.5, 180.1, 196.4, 224.8

Table S73: Mol 13 (LON), RAF transition state, site 0 (M06-2X/ma-def2-SVP, SMD water)

#	Atom	X (Å)	Y (Å)	Z (Å)
1	O	-3.7165	0.5926	0.8214
2	C	-0.3871	-1.3234	-0.2135
3	C	0.3007	0.7128	0.1922
4	C	-0.1821	-0.4733	1.0765
5	C	-0.5507	0.0607	-0.9362
6	C	1.7939	0.9705	-0.0726
7	C	0.8627	-2.1143	-0.6376
8	C	-1.5392	-0.0778	1.6682
9	C	2.4963	-0.1635	-0.8372
10	C	-1.9668	0.5633	-0.7763

Table S73 (Mol 13 (LON), RAF transition state, site 0 (M06-2X/ma-def2-SVP, SMD water)) – Continued

#	Atom	X (Å)	Y (Å)	Z (Å)
11	C	-1.5616	-2.3002	-0.2711
12	C	2.2413	-1.5604	-0.2880
13	C	-2.4460	0.4390	0.5841
14	C	1.8980	2.2455	-0.9213
15	C	2.4917	1.2188	1.2718
16	C	-2.7277	1.0191	-1.7995
17	H	-0.1148	1.6769	0.5333
18	H	0.4855	-0.8723	1.8553
19	H	-0.2050	0.1393	-1.9780
20	H	0.8034	-2.2696	-1.7303
21	H	0.7792	-3.1179	-0.1876
22	H	-1.4125	0.6926	2.4469
23	H	-2.0420	-0.9345	2.1509
24	H	3.5781	0.0510	-0.8384
25	H	2.1811	-0.1375	-1.8956
26	H	2.3863	-1.5704	0.8046
27	H	2.9926	-2.2559	-0.6924
28	H	-2.5421	-1.8395	-0.1039
29	H	-1.5951	-2.7862	-1.2590
30	H	-1.4267	-3.0908	0.4837
31	H	2.9464	2.4503	-1.1891
32	H	1.3234	2.1453	-1.8554
33	H	1.5087	3.1188	-0.3760
34	H	3.5312	1.5476	1.1150
35	H	1.9700	2.0064	1.8382
36	H	2.5156	0.3164	1.8999
37	H	-2.3350	1.0371	-2.8165
38	H	-3.7415	1.3771	-1.6254
39	O	-3.9353	1.5083	2.0819
40	H	-4.6583	0.9980	2.4775

Table S73 (Mol 13 (LON), RAF transition state, site 0 (M06-2X/ma-def2-SVP, SMD water)) – Continued

#	Atom	X (Å)	Y (Å)	Z (Å)
Charge = 0, Multiplicity = 2, E_{el} = -735.67487978 Ha				
E_{ZPE} = 0.35129877 Ha				
10 lowest real freq. (cm^{-1}): 53.2, 88.2, 98.7, 124.1, 150.6, 192.3, 197.5, 204.8, 222.7, 229.6				

Table S74: Mol 13 (LON), RAF transition state, site 12 (M06-2X/ma-def2-SVP, SMD water)

#	Atom	X (Å)	Y (Å)	Z (Å)
1	O	-3.8281	0.0969	0.7976
2	C	-0.3955	-1.3541	-0.2215
3	C	0.2203	0.6787	0.3163
4	C	-0.2351	-0.5719	1.1160
5	C	-0.5809	0.0647	-0.8657
6	C	1.7063	0.9953	0.0596
7	C	0.8680	-2.0854	-0.7011
8	C	-1.6159	-0.2648	1.7062
9	C	2.4285	-0.0613	-0.7950
10	C	-2.0191	0.4696	-0.7483
11	C	-1.5523	-2.3461	-0.3515
12	C	2.2338	-1.5042	-0.3478
13	C	-2.5930	0.2764	0.6461
14	C	1.7596	2.3287	-0.7009
15	C	2.4306	1.1711	1.4006
16	C	-2.7810	0.8680	-1.7656
17	H	-0.2305	1.6053	0.7092
18	H	0.4311	-0.9928	1.8829
19	H	-0.2061	0.2123	-1.8889
20	H	0.7906	-2.1796	-1.8002
21	H	0.8251	-3.1156	-0.3094
22	H	-1.5320	0.4736	2.5161
23	H	-2.0869	-1.1654	2.1268

Table S74 (Mol 13 (LON), RAF transition state, site 12 (M06-2X/ma-def2-SVP, SMD water)) – Continued

#	Atom	X (Å)	Y (Å)	Z (Å)
24	H	3.5022	0.1889	-0.7935
25	H	2.1003	0.0328	-1.8452
26	H	2.4105	-1.5919	0.7366
27	H	2.9979	-2.1368	-0.8259
28	H	-2.5423	-1.9315	-0.1303
29	H	-1.5953	-2.7353	-1.3811
30	H	-1.3909	-3.2000	0.3251
31	H	2.7948	2.5730	-0.9863
32	H	1.1570	2.2825	-1.6214
33	H	1.3715	3.1543	-0.0847
34	H	3.4523	1.5483	1.2355
35	H	1.9033	1.8968	2.0395
36	H	2.5098	0.2272	1.9590
37	H	-2.3584	0.9427	-2.7712
38	H	-3.8326	1.1209	-1.6192
39	O	-2.8918	2.0601	1.1719
40	H	-3.6258	1.9687	1.8099

Charge = 0, Multiplicity = 2, E_{el} = -735.70786262 Ha

E_{ZPE} = 0.35200499 Ha

10 lowest real freq. (cm^{-1}): 77.8, 96.0, 124.1, 152.5, 175.6, 199.4, 217.9, 223.0, 230.8, 248.3

Table S75: Mol 13 (LON), RAF transition state, site 15 (M06-2X/ma-def2-SVP, SMD water)

#	Atom	X (Å)	Y (Å)	Z (Å)
1	O	-3.8345	0.2635	0.7246
2	C	-0.4276	-1.3554	-0.1567
3	C	0.2327	0.6865	0.2895
4	C	-0.2729	-0.5083	1.1433
5	C	-0.5600	0.0309	-0.8814

Table S75 (Mol 13 (LON), RAF transition state, site 15 (M06-2X/ma-def2-SVP, SMD water)) – Continued

#	Atom	X (Å)	Y (Å)	Z (Å)
6	C	1.7357	0.9406	0.0661
7	C	0.8217	-2.1412	-0.5808
8	C	-1.6532	-0.1187	1.6880
9	C	2.4434	-0.1718	-0.7266
10	C	-1.9913	0.4675	-0.8179
11	C	-1.6095	-2.3205	-0.2598
12	C	2.1957	-1.5868	-0.2196
13	C	-2.5904	0.3863	0.5779
14	C	1.8601	2.2434	-0.7361
15	C	2.4280	1.1358	1.4225
16	C	-2.7395	0.8339	-1.8590
17	H	-0.1958	1.6461	0.6279
18	H	0.3658	-0.9078	1.9473
19	H	-0.1593	0.1173	-1.9030
20	H	0.7626	-2.2697	-1.6772
21	H	0.7380	-3.1550	-0.1565
22	H	-1.5576	0.6694	2.4481
23	H	-2.1631	-0.9708	2.1604
24	H	3.5245	0.0451	-0.7148
25	H	2.1374	-0.1164	-1.7862
26	H	2.3432	-1.6318	0.8717
27	H	2.9495	-2.2644	-0.6494
28	H	-2.5926	-1.8738	-0.0704
29	H	-1.6486	-2.7531	-1.2719
30	H	-1.4785	-3.1481	0.4541
31	H	2.9119	2.4471	-0.9907
32	H	1.2885	2.1839	-1.6755
33	H	1.4761	3.0996	-0.1607
34	H	3.4699	1.4634	1.2758

Table S75 (Mol 13 (LON), RAF transition state, site 15 (M06-2X/ma-def2-SVP, SMD water)) – Continued

#	Atom	X (Å)	Y (Å)	Z (Å)
35	H	1.9136	1.9058	2.0187
36	H	2.4505	0.2109	2.0174
37	H	-2.3154	0.8725	-2.8631
38	H	-3.7869	1.1046	-1.7207
39	O	-2.7653	2.2139	0.9559
40	H	-3.7065	2.3257	1.1887

Charge = 0, Multiplicity = 2, E_{el} = -735.70626296 Ha

E_{ZPE} = 0.35198408 Ha

10 lowest real freq. (cm^{-1}): 78.3, 96.4, 124.5, 152.2, 175.8, 199.2, 217.6, 223.2, 230.1, 248.4

11.5 RAF Pre-Reaction Complex (PRC) Geometries (M06-2X/ma-def2-SVP)

Pre-reaction complex geometries for the RAF mechanism, optimized at the M06-2X/ma-def2-SVP level. Energies E_{el} are from the same mid-method calculation (no separate TZVP single-point was performed for PRCs).

Table S76: Mol 1 (CAM), RAF pre-reaction complex (PRC), site 10 (M06-2X/ma-def2-SVP)

#	Atom	X (Å)	Y (Å)	Z (Å)
1	O	2.9532	0.3730	0.4192
2	C	-0.0348	-1.2303	-0.4656
3	C	-1.2681	-0.4234	0.0407
4	C	0.7215	0.8963	-0.4918
5	C	0.5844	-0.2665	-1.4966
6	C	1.0767	-1.2404	0.5996
7	C	1.5673	0.2331	0.6321
8	C	-0.6986	1.0005	0.0088

Table S76 (Mol 1 (CAM), RAF pre-reaction complex (PRC), site 10 (M06-2X/ma-def2-SVP)) – Continued

#	Atom	X (Å)	Y (Å)	Z (Å)
9	C	-1.7549	-0.8321	1.4346
10	C	-2.4393	-0.5642	-0.9441
11	C	-1.3560	2.1247	0.3207
12	H	-0.3062	-2.2297	-0.8347
13	H	1.1567	1.8412	-0.8412
14	H	-0.0645	-0.0295	-2.3500
15	H	1.5502	-0.6231	-1.8892
16	H	0.7560	-1.6120	1.5804
17	H	1.9069	-1.8842	0.2686
18	H	1.3676	0.7121	1.5989
19	H	-1.9968	-1.9071	1.4527
20	H	-1.0161	-0.6146	2.2150
21	H	-2.6671	-0.2745	1.6952
22	H	-3.2645	0.1034	-0.6557
23	H	-2.1524	-0.3141	-1.9745
24	H	-2.8176	-1.5984	-0.9317
25	H	-2.3928	2.1048	0.6649
26	H	-0.8842	3.1044	0.2051
27	H	3.2030	-0.0902	-0.3877
28	O	-0.5824	1.7565	2.8391
29	H	-0.1999	2.6201	2.6024

Charge = 0, Multiplicity = 2, E_{el} = -540.99319529 Ha

E_{ZPE} = 0.25298437 Ha

10 lowest real freq. (cm^{-1}): 55.1, 74.1, 92.7, 106.2, 136.0, 158.4, 207.6, 225.5, 242.7, 264.9

Table S77: Mol 1 (CAM), RAF pre-reaction complex (PRC), site 7 (M06-2X/ma-def2-SVP)

#	Atom	X (Å)	Y (Å)	Z (Å)
1	O	3.0265	0.2159	0.2719
2	C	-0.0293	-1.2731	-0.5474
3	C	-1.2263	-0.4750	0.0508
4	C	0.7671	0.8396	-0.4868
5	C	0.5629	-0.2622	-1.5491
6	C	1.1247	-1.3598	0.4683
7	C	1.6510	0.1001	0.5557
8	C	-0.6330	0.9400	0.0685
9	C	-1.6514	-0.9398	1.4480
10	C	-2.4446	-0.5557	-0.8807
11	C	-1.2629	2.0574	0.4529
12	H	-0.3340	-2.2460	-0.9594
13	H	1.2028	1.7959	-0.8005
14	H	-0.1188	0.0336	-2.3576
15	H	1.5025	-0.6133	-2.0049
16	H	0.8388	-1.7787	1.4410
17	H	1.9253	-2.0022	0.0691
18	H	1.5160	0.5400	1.5511
19	H	-1.9035	-2.0124	1.4390
20	H	-0.8676	-0.7692	2.1973
21	H	-2.5504	-0.3900	1.7695
22	H	-3.2444	0.1129	-0.5291
23	H	-2.1989	-0.2672	-1.9115
24	H	-2.8410	-1.5832	-0.8948
25	H	-2.3025	2.0341	0.7950
26	H	-0.7712	3.0307	0.4094
27	H	3.2139	-0.1890	-0.5818
28	O	-0.0766	1.7872	2.8005
29	H	-1.0183	1.6683	3.0175

Charge = 0, Multiplicity = 2, E_{el} = -540.99454949 Ha

Table S77 (Mol 1 (CAM), RAF pre-reaction complex (PRC), site 7 (M06-2X/ma-def2-SVP)) – Continued

#	Atom	X (Å)	Y (Å)	Z (Å)

$E_{\mathrm{ZPE}} = 0.25313281$ Ha

10 lowest real freq. (cm^{-1}): 54.5, 81.2, 85.5, 109.0, 140.0, 168.3, 208.8, 233.8, 242.6, 267.1

Table S78: Mol 2 (IPO), RAF pre-reaction complex (PRC), site 10 (M06-2X/ma-def2-SVP)

#	Atom	X (Å)	Y (Å)	Z (Å)
1	O	-2.6159	0.5922	-0.6843
2	C	1.4287	-0.0628	-0.5456
3	C	0.9403	1.2025	0.2254
4	C	0.6140	-0.8733	0.5350
5	C	0.8637	0.3175	1.4958
6	C	-0.4749	1.5993	-0.2086
7	C	-0.8143	-0.8923	0.0709
8	C	-1.4749	0.4765	0.1215
9	C	2.9342	-0.2841	-0.4036
10	C	1.0455	-0.2268	-2.0107
11	C	-1.4233	-1.9852	-0.4175
12	H	1.6208	2.0681	0.2337
13	H	0.9862	-1.8740	0.8020
14	H	0.0931	0.5484	2.2443
15	H	1.8279	0.2312	2.0075
16	H	-0.5110	1.8104	-1.2877
17	H	-0.7948	2.5174	0.3057
18	H	-1.7650	0.6015	1.1801
19	H	3.4799	0.4472	-1.0203
20	H	3.2925	-0.1826	0.6290
21	H	3.2071	-1.2900	-0.7585
22	H	1.3210	-1.2343	-2.3614
23	H	-0.0276	-0.0996	-2.1995
24	H	1.5926	0.5042	-2.6272

Table S78 (Mol 2 (IPO), RAF pre-reaction complex (PRC), site 10 (M06-2X/ma-def2-SVP)) – Continued

#	Atom	X (Å)	Y (Å)	Z (Å)
25	H	-2.3992	-1.9210	-0.9014
26	H	-0.9188	-2.9549	-0.4101
27	H	-3.3156	0.0645	-0.2781
28	O	-3.2688	-1.4331	1.3752
29	H	-3.0983	-2.3475	1.6605

Charge = 0, Multiplicity = 2, E_{el} = -540.97477572 Ha

E_{ZPE} = 0.25318593 Ha

10 lowest real freq. (cm^{-1}): 59.9, 91.5, 123.5, 156.7, 174.3, 201.8, 211.0, 243.6, 306.5, 314.9

Table S79: Mol 2 (IPO), RAF pre-reaction complex (PRC), site 6 (M06-2X/ma-def2-SVP)

#	Atom	X (Å)	Y (Å)	Z (Å)
1	O	-2.6027	0.4822	-0.2076
2	C	1.4455	-0.0606	-0.4071
3	C	0.9996	1.3184	0.1701
4	C	0.7931	-0.6676	0.8931
5	C	1.1033	0.6870	1.5816
6	C	-0.4698	1.6019	-0.1565
7	C	-0.6781	-0.8018	0.6176
8	C	-1.3858	0.5399	0.4812
9	C	2.9649	-0.2243	-0.4090
10	C	0.9019	-0.5041	-1.7591
11	C	-1.2958	-1.9784	0.4313
12	H	1.6417	2.1820	-0.0627
13	H	1.2336	-1.5930	1.2941
14	H	0.4142	1.0440	2.3602
15	H	2.1218	0.7151	1.9828
16	H	-0.6375	1.6095	-1.2436
17	H	-0.7662	2.5929	0.2174
18	H	-1.5747	0.8663	1.5222

Table S79 (Mol 2 (IPO), RAF pre-reaction complex (PRC), site 6 (M06-2X/ma-def2-SVP)) – Continued

#	Atom	X (Å)	Y (Å)	Z (Å)
19	H	3.4071	0.3896	-1.2092
20	H	3.4344	0.0734	0.5377
21	H	3.2353	-1.2736	-0.6044
22	H	1.1723	-1.5555	-1.9465
23	H	-0.1899	-0.4312	-1.8374
24	H	1.3465	0.1048	-2.5624
25	H	-2.3232	-2.0316	0.0662
26	H	-0.7605	-2.9201	0.5763
27	H	-3.2388	0.0310	0.3633
28	O	-3.2280	-1.2592	2.0975
29	H	-2.5095	-1.3642	2.7470

Charge = 0, Multiplicity = 2, E_{el} = -540.97162761 Ha

E_{ZPE} = 0.25354080 Ha

10 lowest real freq. (cm^{-1}): 56.0, 85.0, 121.6, 151.7, 175.9, 200.8, 206.8, 211.8, 245.3, 305.6

Table S80: Mol 3 (CYM), RAF pre-reaction complex (PRC), site 5 (M06-2X/ma-def2-SVP)

#	Atom	X (Å)	Y (Å)	Z (Å)
1	O	2.2566	-0.4698	1.4107
2	C	1.9043	0.0377	0.1103
3	C	0.3760	0.0600	-0.0063
4	C	2.5284	1.4263	0.0512
5	C	2.5272	-0.8542	-0.9645
6	C	-0.3636	1.2443	-0.0509
7	C	-0.3346	-1.1502	-0.0064
8	C	-2.4654	0.0156	-0.0996
9	C	-1.7596	1.2193	-0.0974
10	C	-1.7243	-1.1732	-0.0501
11	C	-3.9697	-0.0173	-0.1665
12	H	3.6168	1.3315	0.1617

Table S80 (Mol 3 (CYM), RAF pre-reaction complex (PRC), site 5 (M06-2X/ma-def2-SVP)) – Continued

#	Atom	X (Å)	Y (Å)	Z (Å)
13	H	2.3119	1.9093	-0.9115
14	H	2.1528	2.0609	0.8651
15	H	3.6192	-0.8727	-0.8422
16	H	2.2850	-0.4716	-1.9660
17	H	2.1544	-1.8872	-0.9018
18	H	0.1404	2.2102	-0.0349
19	H	0.1995	-2.1037	0.0305
20	H	-2.3074	2.1639	-0.1226
21	H	-2.2471	-2.1329	-0.0434
22	H	1.9804	-1.3917	1.4784
23	H	-4.3830	-0.7257	0.5652
24	H	-4.3989	0.9726	0.0345
25	H	-4.3099	-0.3362	-1.1638
26	O	0.5075	1.3122	2.8086
27	H	1.1664	0.6386	2.5240

Charge = 0, Multiplicity = 2, E_{el} = -539.82369195 Ha

E_{ZPE} = 0.22912543 Ha

10 lowest real freq. (cm^{-1}): 30.5, 50.8, 87.6, 98.9, 162.7, 202.2, 220.4, 252.8, 298.8, 310.5

Table S81: Mol 3 (CYM), RAF pre-reaction complex (PRC), site 6 (M06-2X/ma-def2-SVP)

#	Atom	X (Å)	Y (Å)	Z (Å)
1	O	2.4439	-1.2850	0.7933
2	C	1.9857	-0.2185	-0.0174
3	C	0.4559	-0.1498	0.0241
4	C	2.6355	1.0868	0.4499
5	C	2.4331	-0.5576	-1.4399
6	C	-0.2520	1.0402	-0.1944
7	C	-0.2843	-1.3187	0.2580
8	C	-2.3880	-0.1080	0.0607

Table S81 (Mol 3 (CYM), RAF pre-reaction complex (PRC), site 6 (M06-2X/ma-def2-SVP)) – Continued

#	Atom	X (Å)	Y (Å)	Z (Å)
9	C	-1.6463	1.0588	-0.1720
10	C	-1.6804	-1.2947	0.2741
11	C	-3.8944	-0.0872	0.0493
12	H	3.7229	0.9365	0.4934
13	H	2.4326	1.9207	-0.2362
14	H	2.2794	1.3615	1.4547
15	H	3.5299	-0.6271	-1.4736
16	H	2.0964	0.2128	-2.1478
17	H	2.0106	-1.5258	-1.7426
18	H	0.2837	1.9727	-0.3770
19	H	0.2493	-2.2535	0.4362
20	H	-2.1712	2.0032	-0.3367
21	H	-2.2278	-2.2201	0.4682
22	H	2.1307	-1.1305	1.6958
23	H	-4.3089	-0.8753	0.6919
24	H	-4.2842	0.8803	0.3935
25	H	-4.2769	-0.2547	-0.9698
26	O	0.4166	-0.5106	2.7589
27	H	-0.2005	0.2089	2.5250

Charge = 0, Multiplicity = 2, E_{el} = -539.83859440 Ha

E_{ZPE} = 0.22860868 Ha

10 lowest real freq. (cm^{-1}): 43.1, 56.2, 77.3, 131.0, 154.1, 181.9, 198.7, 220.1, 249.5, 285.1

Table S82: Mol 3 (CYM), RAF pre-reaction complex (PRC), site 7 (M06-2X/ma-def2-SVP)

#	Atom	X (Å)	Y (Å)	Z (Å)
1	O	2.3339	-0.7678	1.2214
2	C	1.9325	-0.0433	0.0610
3	C	0.4023	-0.0029	-0.0125
4	C	2.5504	1.3379	0.2330

Table S82 (Mol 3 (CYM), RAF pre-reaction complex (PRC), site 7 (M06-2X/ma-def2-SVP)) – Continued

#	Atom	X (Å)	Y (Å)	Z (Å)
5	C	2.4985	-0.7166	-1.1936
6	C	-0.3314	1.1857	-0.0477
7	C	-0.3169	-1.2093	-0.0333
8	C	-2.4437	-0.0315	-0.0674
9	C	-1.7291	1.1701	-0.0739
10	C	-1.7073	-1.2248	-0.0607
11	C	-3.9468	-0.0457	-0.0107
12	H	3.6364	1.2243	0.3476
13	H	2.3547	1.9754	-0.6398
14	H	2.1599	1.8273	1.1356
15	H	3.5953	-0.7540	-1.1291
16	H	2.2093	-0.1613	-2.0972
17	H	2.1223	-1.7458	-1.2969
18	H	0.1801	2.1480	-0.0380
19	H	0.2131	-2.1663	-0.0273
20	H	-2.2727	2.1174	-0.0799
21	H	-2.2350	-2.1812	-0.0613
22	H	1.9874	-1.6653	1.1770
23	H	-4.2815	-0.0740	1.0381
24	H	-4.3726	0.8537	-0.4740
25	H	-4.3583	-0.9283	-0.5184
26	O	-1.8030	0.3534	2.7758
27	H	-0.9475	0.1617	2.3422

Charge = 0, Multiplicity = 2, E_{el} = -539.82733929 Ha

E_{ZPE} = 0.22757460 Ha

10 lowest real freq. (cm^{-1}): 18.1, 33.6, 58.1, 87.1, 129.2, 173.3, 199.1, 223.7, 245.9, 265.9

Table S83: Mol 4 (MYR), RAF pre-reaction complex (PRC), site 6 (M06-2X/ma-def2-SVP)

#	Atom	X (Å)	Y (Å)	Z (Å)
1	O	2.7480	-1.2759	0.2644
2	C	-1.2010	-0.6942	0.1621
3	C	-1.6170	0.7745	-0.1652
4	C	-0.0080	-0.4310	-0.8203
5	C	-0.9394	0.5838	-1.5437
6	C	-0.7975	1.7813	0.6550
7	C	1.0724	0.3968	-0.1574
8	C	-2.1935	-1.7117	-0.4036
9	C	-0.9090	-1.0742	1.6064
10	C	0.6792	1.4856	0.5311
11	C	2.5069	-0.0202	-0.3281
12	H	-2.6924	1.0107	-0.1364
13	H	0.4029	-1.2891	-1.3712
14	H	-0.4527	1.4613	-1.9927
15	H	-1.5988	0.1160	-2.2823
16	H	-1.1069	1.7584	1.7162
17	H	-1.0052	2.8072	0.3058
18	H	-2.4914	-1.5020	-1.4383
19	H	-1.7561	-2.7216	-0.3778
20	H	-3.1074	-1.7264	0.2111
21	H	-0.1192	-0.4606	2.0514
22	H	-1.8203	-0.9754	2.2178
23	H	-0.5772	-2.1224	1.6638
24	H	1.4125	2.1727	0.9640
25	H	3.1746	0.7572	0.0815
26	H	2.7329	-0.1355	-1.4001
27	H	2.6262	-1.1612	1.2164
28	O	2.1788	0.4394	2.5451
29	H	1.3601	0.5957	3.0499

Charge = 0, Multiplicity = 2, E_{el} = -540.97140301 Ha

Table S83 (Mol 4 (MYR), RAF pre-reaction complex (PRC), site 6 (M06-2X/ma-def2-SVP)) – Continued

#	Atom	X (Å)	Y (Å)	Z (Å)

E_{ZPE} = 0.25382669 Ha

10 lowest real freq. (cm^{-1}): 60.2, 101.5, 104.1, 134.0, 136.4, 173.7, 199.4, 214.7, 238.9, 263.5

Table S84: Mol 4 (MYR), RAF pre-reaction complex (PRC), site 9 (M06-2X/ma-def2-SVP)

#	Atom	X (Å)	Y (Å)	Z (Å)
1	O	2.7805	-1.0900	0.4292
2	C	-1.2176	-0.6748	0.1393
3	C	-1.6619	0.7532	-0.3097
4	C	0.0296	-0.4270	-0.7770
5	C	-0.8842	0.5057	-1.6248
6	C	-0.9361	1.8373	0.5011
7	C	1.0346	0.4831	-0.1056
8	C	-2.1239	-1.7677	-0.4311
9	C	-1.0135	-0.9516	1.6213
10	C	0.5537	1.5919	0.4884
11	C	2.4942	0.1346	-0.1997
12	H	-2.7435	0.9514	-0.3686
13	H	0.5062	-1.3013	-1.2433
14	H	-0.3982	1.3720	-2.0963
15	H	-1.4740	-0.0322	-2.3742
16	H	-1.3089	1.8617	1.5403
17	H	-1.1527	2.8323	0.0758
18	H	-2.3223	-1.6586	-1.5047
19	H	-1.6648	-2.7559	-0.2735
20	H	-3.0930	-1.7623	0.0918
21	H	-0.3889	-0.2075	2.1253
22	H	-1.9896	-0.9887	2.1316
23	H	-0.5314	-1.9330	1.7571
24	H	1.2253	2.3530	0.9012

Table S84 (Mol 4 (MYR), RAF pre-reaction complex (PRC), site 9 (M06-2X/ma-def2-SVP)) – Continued

#	Atom	X (Å)	Y (Å)	Z (Å)
25	H	3.1060	0.9595	0.2111
26	H	2.7735	0.0123	-1.2586
27	H	2.5387	-0.9878	1.3601
28	O	1.7332	0.4223	2.6832
29	H	2.0850	1.3302	2.6869

Charge = 0, Multiplicity = 2, E_{el} = -540.97189442 Ha

E_{ZPE} = 0.25384694 Ha

10 lowest real freq. (cm^{-1}): 51.2, 97.7, 110.1, 131.8, 166.9, 181.0, 198.3, 204.5, 230.1, 261.4

Table S85: Mol 5 (VER), RAF pre-reaction complex (PRC), site 5 (M06-2X/ma-def2-SVP)

#	Atom	X (Å)	Y (Å)	Z (Å)
1	O	1.1109	2.5617	-0.5467
2	C	-1.2573	-0.0422	-0.3391
3	C	-0.3215	-0.9203	0.5707
4	C	-0.6044	1.1706	0.4088
5	C	-0.3971	0.2257	1.6188
6	C	1.0891	-0.9379	0.0178
7	C	-1.1234	-0.1238	-1.8549
8	C	-2.7312	-0.2174	0.0254
9	C	0.7690	1.4570	-0.1878
10	C	1.6289	0.2523	-0.3104
11	C	1.7734	-2.2552	-0.1524
12	H	-0.6765	-1.9258	0.8463
13	H	-1.1745	2.1029	0.5212
14	H	-1.2778	0.1718	2.2688
15	H	0.5057	0.4030	2.2237
16	H	-1.4830	-1.1007	-2.2148
17	H	-1.7478	0.6525	-2.3230
18	H	-0.0954	0.0142	-2.2101

Table S85 (Mol 5 (VER), RAF pre-reaction complex (PRC), site 5 (M06-2X/ma-def2-SVP)) – Continued

#	Atom	X (Å)	Y (Å)	Z (Å)
19	H	-3.0963	-1.1861	-0.3496
20	H	-2.9227	-0.1832	1.1051
21	H	-3.3320	0.5731	-0.4493
22	H	2.6336	0.3688	-0.7211
23	H	1.1999	-2.8816	-0.8554
24	H	1.7993	-2.7967	0.8059
25	H	2.7945	-2.1375	-0.5356
26	O	0.4129	-2.4908	2.9358
27	H	0.5013	-1.5802	3.2755

Charge = 0, Multiplicity = 2, E_{el} = -539.78166094 Ha

E_{ZPE} = 0.22787095 Ha

10 lowest real freq. (cm^{-1}): 39.9, 73.1, 99.2, 111.5, 118.2, 142.7, 154.8, 182.0, 193.3, 201.5

Table S86: Mol 6 (CRV), RAF pre-reaction complex (PRC), site 10 (M06-2X/ma-def2-SVP)

#	Atom	X (Å)	Y (Å)	Z (Å)
1	O	-2.0298	-1.8961	-0.0911
2	C	0.9548	0.3993	-0.3509
3	C	0.1783	-0.8949	-0.0823
4	C	-1.2808	-0.7689	-0.5091
5	C	0.3467	1.5253	0.4844
6	C	-1.9110	0.5303	-0.0414
7	C	-1.1558	1.5427	0.4060
8	C	2.4544	0.2466	-0.1932
9	C	-3.4076	0.6155	-0.1517
10	C	3.0828	-0.9316	-0.8876
11	C	3.2131	1.1346	0.4756
12	H	0.7997	0.6500	-1.4205
13	H	0.2275	-1.1107	0.9992
14	H	0.6165	-1.7559	-0.6062

Table S86 (Mol 6 (CRV), RAF pre-reaction complex (PRC), site 10 (M06-2X/ma-def2-SVP)) – Continued

#	Atom	X (Å)	Y (Å)	Z (Å)
15	H	-1.3429	-0.7979	-1.6123
16	H	0.7368	2.5016	0.1545
17	H	0.6622	1.4015	1.5363
18	H	-1.6552	2.4616	0.7314
19	H	-3.7282	0.4093	-1.1856
20	H	-3.7703	1.6108	0.1364
21	H	-3.8991	-0.1389	0.4793
22	H	2.8429	-1.8640	-0.3546
23	H	2.6972	-1.0336	-1.9144
24	H	4.1751	-0.8328	-0.9316
25	H	-1.9560	-1.9713	0.8675
26	H	4.3003	1.0243	0.5025
27	H	2.7890	1.9982	0.9878
28	O	2.2571	-0.5830	2.3060
29	H	3.1872	-0.4129	2.5373

Charge = 0, Multiplicity = 2, E_{el} = -540.98374064 Ha

E_{ZPE} = 0.25174335 Ha

10 lowest real freq. (cm^{-1}): 48.4, 68.3, 81.6, 109.2, 132.6, 138.5, 164.1, 170.7, 180.2, 224.2

Table S87: Mol 6 (CRV), RAF pre-reaction complex (PRC), site 6 (M06-2X/ma-def2-SVP)

#	Atom	X (Å)	Y (Å)	Z (Å)
1	O	-2.0540	-1.8661	-0.1250
2	C	1.0094	0.3588	-0.2874
3	C	0.1681	-0.9022	-0.0379
4	C	-1.3151	-0.6913	-0.3454
5	C	0.4532	1.5140	0.5591
6	C	-1.9066	0.4847	0.4615
7	C	-1.0110	1.6801	0.3370

Table S87 (Mol 6 (CRV), RAF pre-reaction complex (PRC), site 6 (M06-2X/ma-def2-SVP)) – Continued

#	Atom	X (Å)	Y (Å)	Z (Å)
8	C	2.4905	0.0988	-0.0918
9	C	-3.3324	0.7943	0.0236
10	C	3.0843	-0.9786	-0.9634
11	C	3.2461	0.7741	0.7818
12	H	0.8778	0.6334	-1.3528
13	H	0.2772	-1.2069	1.0174
14	H	0.5125	-1.7398	-0.6609
15	H	-1.4280	-0.4469	-1.4151
16	H	0.9908	2.4478	0.3412
17	H	0.6402	1.2721	1.6243
18	H	-1.4530	2.6698	0.2001
19	H	-3.3456	1.1444	-1.0188
20	H	-3.7622	1.5888	0.6543
21	H	-3.9580	-0.1039	0.1030
22	H	2.7464	-1.9775	-0.6473
23	H	2.7745	-0.8506	-2.0127
24	H	4.1809	-0.9645	-0.9202
25	H	-2.1307	-1.9742	0.8330
26	H	4.3131	0.5606	0.8760
27	H	2.8420	1.5539	1.4280
28	O	-1.9045	-0.0064	1.8147
29	H	-2.2937	0.6598	2.3917

Charge = 0, Multiplicity = 2, E_{el} = -540.98514788 Ha

E_{ZPE} = 0.25477376 Ha

10 lowest real freq. (cm^{-1}): 50.2, 72.1, 153.4, 161.4, 165.8, 197.4, 236.4, 243.0, 266.6, 274.5

Table S88: Mol 6 (CRV), RAF pre-reaction complex (PRC), site 7 (M06-2X/ma-def2-SVP)

#	Atom	X (Å)	Y (Å)	Z (Å)
1	O	-2.1001	-1.8893	-0.2355
2	C	0.9297	0.3743	-0.1862
3	C	0.1219	-0.9283	-0.0386
4	C	-1.3054	-0.7587	-0.5480
5	C	0.2816	1.4483	0.6908
6	C	-1.9445	0.5293	-0.0577
7	C	-1.2101	1.5043	0.4969
8	C	2.3938	0.1603	0.0739
9	C	-3.4277	0.6490	-0.2707
10	C	3.1695	-0.6885	-0.8801
11	C	2.9561	0.4103	1.4333
12	H	0.8251	0.6949	-1.2420
13	H	0.1056	-1.2024	1.0314
14	H	0.5999	-1.7588	-0.5798
15	H	-1.2970	-0.7317	-1.6528
16	H	0.7214	2.4359	0.4734
17	H	0.4995	1.2356	1.7526
18	H	-1.7201	2.4144	0.8310
19	H	-3.6760	0.4992	-1.3338
20	H	-3.7950	1.6358	0.0401
21	H	-3.9746	-0.1262	0.2852
22	H	3.1736	-1.7503	-0.5658
23	H	2.7437	-0.6496	-1.8939
24	H	4.2235	-0.3741	-0.9368
25	H	-2.0746	-2.0234	0.7191
26	H	4.0507	0.2673	1.4132
27	H	2.7617	1.4534	1.7471
28	O	2.3546	-0.4901	2.3636
29	H	2.6949	-0.3087	3.2462

Charge = 0, Multiplicity = 2, E_{el} = -541.03894201 Ha

Table S88 (Mol 6 (CRV), RAF pre-reaction complex (PRC), site 7 (M06-2X/ma-def2-SVP)) – Continued

#	Atom	X (Å)	Y (Å)	Z (Å)

E_{ZPE} = 0.25570361 Ha

10 lowest real freq. (cm^{-1}): 49.2, 65.3, 91.3, 117.6, 125.5, 142.0, 167.3, 199.1, 281.5, 283.8

Table S89: Mol 8 (CAR), RAF pre-reaction complex (PRC), site 11 (M06-2X/ma-def2-SVP)

#	Atom	X (Å)	Y (Å)	Z (Å)
1	O	0.0783	-0.6577	-1.2132
2	C	0.2558	-0.0931	0.0780
3	C	-0.3019	1.3872	0.1446
4	C	1.7938	0.0391	0.3614
5	C	0.7914	2.1378	0.9222
6	C	2.0922	1.5446	0.3956
7	C	-0.3846	-1.0156	1.1348
8	C	-1.6621	1.5099	0.8530
9	C	2.7091	-0.7835	-0.5558
10	C	-0.4237	1.9875	-1.2642
11	C	-1.7415	-1.6070	0.7496
12	C	-2.7662	0.6671	0.2637
13	C	-2.8180	-0.6832	0.2335
14	C	4.1794	-0.4520	-0.2899
15	C	2.4816	-2.2880	-0.3974
16	C	-4.0039	-1.3972	-0.3568
17	H	1.9508	-0.3327	1.3920
18	H	0.6985	1.9167	2.0001
19	H	0.7111	3.2295	0.8027
20	H	2.3133	1.9223	-0.6164
21	H	2.9589	1.7839	1.0278
22	H	-0.4428	-0.4834	2.0961
23	H	0.2978	-1.8626	1.3017
24	H	-1.9642	2.5682	0.8144

Table S89 (Mol 8 (CAR), RAF pre-reaction complex (PRC), site 11 (M06-2X/ma-def2-SVP)) – Continued

#	Atom	X (Å)	Y (Å)	Z (Å)
25	H	-1.5531	1.2658	1.9201
26	H	2.4686	-0.5076	-1.5959
27	H	-0.6272	3.0662	-1.1836
28	H	0.4882	1.8443	-1.8575
29	H	-1.2529	1.5364	-1.8297
30	H	-2.1628	-2.1466	1.6143
31	H	-1.5793	-2.3800	-0.0222
32	H	-0.8554	-0.5965	-1.4458
33	H	-3.6164	1.2001	-0.1789
34	H	4.4383	-0.6468	0.7646
35	H	4.8349	-1.0774	-0.9140
36	H	4.4172	0.5988	-0.5077
37	H	2.6897	-2.6022	0.6397
38	H	3.1588	-2.8530	-1.0554
39	H	1.4513	-2.5679	-0.6508
40	H	-4.4335	-2.0829	0.3898
41	H	-3.7022	-2.0082	-1.2228
42	H	-4.7862	-0.6973	-0.6790
43	O	-3.7143	-0.2612	2.5615
44	H	-4.3304	0.4733	2.3941

Charge = 0, Multiplicity = 2, E_{el} = -737.33255171 Ha

E_{ZPE} = 0.39666512 Ha

10 lowest real freq. (cm^{-1}): 36.6, 63.4, 91.0, 94.9, 99.4, 123.0, 131.7, 144.3, 178.0, 186.9

Table S90: Mol 9 (CAO), RAF pre-reaction complex (PRC), site 15 (M06-2X/ma-def2-SVP)

#	Atom	X (Å)	Y (Å)	Z (Å)
1	O	3.0143	-1.2137	-0.4234
2	C	-1.0338	-0.4352	-0.0694

Table S90 (Mol 9 (CAO), RAF pre-reaction complex (PRC), site 15 (M06-2X/ma-def2-SVP)) – Continued

#	Atom	X (Å)	Y (Å)	Z (Å)
3	C	-2.5669	-0.1819	-0.2000
4	C	-0.8789	1.0480	0.3821
5	C	-2.2380	1.3259	-0.3066
6	C	1.8635	-0.9652	0.3760
7	C	-0.5514	-1.5902	0.8083
8	C	2.0245	-0.3598	-0.9585
9	C	0.8641	-2.0950	0.4900
10	C	0.2766	1.9583	0.0455
11	C	2.2977	1.0932	-1.2299
12	C	-3.2406	-0.8294	-1.4006
13	C	-3.3361	-0.4988	1.0818
14	C	0.9689	1.8676	-1.3013
15	C	2.1424	-0.2202	1.6593
16	C	0.6672	2.8846	0.9303
17	H	-0.6426	-0.5511	-1.0944
18	H	-1.0295	1.0889	1.4715
19	H	-2.9120	2.0415	0.1884
20	H	-2.0984	1.6307	-1.3564
21	H	-1.2463	-2.4379	0.6845
22	H	-0.6274	-1.2947	1.8682
23	H	1.4459	-0.8382	-1.7624
24	H	1.1921	-2.8101	1.2623
25	H	0.8611	-2.6393	-0.4674
26	H	2.8386	1.1863	-2.1850
27	H	2.9411	1.5222	-0.4485
28	H	-3.2459	-1.9275	-1.3016
29	H	-4.2869	-0.4976	-1.4948
30	H	-2.7170	-0.5747	-2.3346
31	H	-4.3618	-0.1043	1.0113

Table S90 (Mol 9 (CAO), RAF pre-reaction complex (PRC), site 15 (M06-2X/ma-def2-SVP)) – Continued

#	Atom	X (Å)	Y (Å)	Z (Å)
32	H	-3.4062	-1.5854	1.2453
33	H	-2.8635	-0.0552	1.9709
34	H	0.3074	1.3898	-2.0438
35	H	1.1701	2.8831	-1.6722
36	H	2.5697	-0.9228	2.3922
37	H	2.8508	0.6022	1.5070
38	H	1.2106	0.2001	2.0658
39	H	0.2094	2.9514	1.9208
40	H	1.4550	3.6047	0.6916
41	O	-0.4031	0.4825	3.7351
42	H	0.4118	0.5003	4.2713

Charge = 0, Multiplicity = 2, E_{el} = -736.06911553 Ha

E_{ZPE} = 0.37207350 Ha

10 lowest real freq. (cm^{-1}): 32.1, 42.6, 80.1, 80.7, 88.6, 105.4, 111.5, 151.0, 166.0, 186.0

Table S91: Mol 9 (CAO), RAF pre-reaction complex (PRC), site 9 (M06-2X/ma-def2-SVP)

#	Atom	X (Å)	Y (Å)	Z (Å)
1	O	3.0080	-1.2510	-0.5801
2	C	-1.0319	-0.4718	-0.1459
3	C	-2.5643	-0.2025	-0.2582
4	C	-0.8719	0.9749	0.4079
5	C	-2.2287	1.3072	-0.2598
6	C	1.8682	-1.0582	0.2495
7	C	-0.5527	-1.6891	0.6452
8	C	2.0221	-0.3481	-1.0336
9	C	0.8566	-2.1830	0.2827
10	C	0.2845	1.9081	0.1468
11	C	2.3087	1.1191	-1.1864

Table S91 (Mol 9 (CAO), RAF pre-reaction complex (PRC), site 9 (M06-2X/ma-def2-SVP)) – Continued

#	Atom	X (Å)	Y (Å)	Z (Å)
12	C	-3.2403	-0.7625	-1.5005
13	C	-3.3352	-0.6019	0.9995
14	C	0.9878	1.9087	-1.1975
15	C	2.1707	-0.4283	1.5869
16	C	0.6493	2.7906	1.0863
17	H	-0.6402	-0.5125	-1.1761
18	H	-1.0362	0.9359	1.4983
19	H	-2.8982	1.9909	0.2836
20	H	-2.0830	1.6844	-1.2850
21	H	-1.2561	-2.5194	0.4655
22	H	-0.6212	-1.4684	1.7239
23	H	1.4326	-0.7530	-1.8693
24	H	1.1794	-2.9570	0.9980
25	H	0.8438	-2.6537	-0.7128
26	H	2.8499	1.2848	-2.1314
27	H	2.9575	1.4783	-0.3750
28	H	-3.2518	-1.8645	-1.4762
29	H	-4.2846	-0.4193	-1.5718
30	H	-2.7150	-0.4478	-2.4151
31	H	-4.3605	-0.2031	0.9524
32	H	-3.4064	-1.6966	1.0913
33	H	-2.8660	-0.2172	1.9174
34	H	0.3235	1.4950	-1.9751
35	H	1.1980	2.9475	-1.4896
36	H	2.6772	-1.1724	2.2208
37	H	2.8204	0.4493	1.4921
38	H	1.2384	-0.1308	2.0851
39	H	0.1517	2.8225	2.0599
40	H	1.4338	3.5299	0.9041
41	O	-0.7367	0.6089	3.5825

Table S91 (Mol 9 (CAO), RAF pre-reaction complex (PRC), site 9 (M06-2X/ma-def2-SVP)) – Continued

#	Atom	X (Å)	Y (Å)	Z (Å)
42	H	0.1486	1.0215	3.5926

Charge = 0, Multiplicity = 2, E_{el} = -736.05158895 Ha

E_{ZPE} = 0.37207084 Ha

10 lowest real freq. (cm^{-1}): 31.6, 61.4, 81.3, 82.5, 95.8, 114.6, 149.1, 161.7, 177.1, 187.1

Table S92: Mol 11 (DHE), RAF pre-reaction complex (PRC), site 11 (M06-2X/ma-def2-SVP)

#	Atom	X (Å)	Y (Å)	Z (Å)
1	O	-3.9977	1.4777	-1.2075
2	C	1.2719	-1.0063	-0.2525
3	C	0.3315	-2.0216	0.4137
4	C	-1.4343	-0.2908	0.9042
5	C	-1.1410	-1.6536	0.2660
6	C	0.9116	0.4030	0.2001
7	C	2.7167	-1.3124	0.1762
8	C	-0.3124	0.6897	0.6969
9	C	-2.7895	0.3232	0.4899
10	C	1.1580	-1.0805	-1.7861
11	C	3.7174	-0.3016	-0.3747
12	C	1.9548	1.4472	0.0675
13	C	3.2314	1.1086	-0.2066
14	C	-2.8056	0.7561	-0.9722
15	C	-3.9625	-0.6044	0.7974
16	C	1.5617	2.8895	0.2582
17	H	0.5816	-2.0656	1.4871
18	H	0.5237	-3.0210	-0.0104
19	H	-1.4922	-0.4401	1.9993
20	H	-1.4196	-1.6452	-0.8000
21	H	-1.7676	-2.4244	0.7385

Table S92 (Mol 11 (DHE), RAF pre-reaction complex (PRC), site 11 (M06-2X/ma-def2-SVP)) – Continued

#	Atom	X (Å)	Y (Å)	Z (Å)
22	H	2.9810	-2.3330	-0.1452
23	H	2.7557	-1.3038	1.2783
24	H	-0.5337	1.7166	1.0037
25	H	-2.9182	1.2474	1.0812
26	H	1.7687	-0.3064	-2.2730
27	H	1.4946	-2.0663	-2.1463
28	H	0.1202	-0.9286	-2.1147
29	H	3.9168	-0.4923	-1.4449
30	H	4.6922	-0.4167	0.1253
31	H	3.9684	1.9099	-0.3218
32	H	-1.9188	1.3809	-1.1862
33	H	-2.7556	-0.1327	-1.6292
34	H	-3.9493	-0.9195	1.8517
35	H	-3.9255	-1.5093	0.1705
36	H	-4.9147	-0.0966	0.5967
37	H	1.1988	3.0750	1.2816
38	H	0.7497	3.1736	-0.4285
39	H	2.4171	3.5529	0.0786
40	H	-4.0450	1.7280	-2.1344
41	O	1.0538	0.2446	2.8882
42	H	1.8362	0.6862	2.5097

Charge = 0, Multiplicity = 2, E_{el} = -736.12709676 Ha

E_{ZPE} = 0.37453765 Ha

10 lowest real freq. (cm^{-1}): 37.1, 59.6, 63.0, 64.9, 73.3, 118.7, 135.9, 150.3, 178.0, 203.4

Table S93: Mol 11 (DHE), RAF pre-reaction complex (PRC), site 12 (M06-2X/ma-def2-SVP)

#	Atom	X (Å)	Y (Å)	Z (Å)
1	O	-3.9879	1.5558	-1.1230
2	C	1.2546	-1.0601	-0.2523
3	C	0.2815	-2.1027	0.3178
4	C	-1.4710	-0.3842	0.8901
5	C	-1.1813	-1.7010	0.1608
6	C	0.8922	0.3258	0.2716
7	C	2.6819	-1.4026	0.2071
8	C	-0.3328	0.5948	0.7640
9	C	-2.8133	0.2732	0.5014
10	C	1.1915	-1.0497	-1.7900
11	C	3.7096	-0.3782	-0.2606
12	C	1.9417	1.3669	0.2001
13	C	3.2306	1.0251	-0.0466
14	C	-2.8059	0.8069	-0.9271
15	C	-4.0017	-0.6572	0.7311
16	C	1.5639	2.8044	0.4356
17	H	0.5022	-2.2361	1.3915
18	H	0.4699	-3.0743	-0.1676
19	H	-1.5614	-0.6176	1.9695
20	H	-1.4275	-1.6056	-0.9087
21	H	-1.8332	-2.4950	0.5544
22	H	2.9473	-2.4102	-0.1523
23	H	2.6975	-1.4389	1.3092
24	H	-0.5480	1.6060	1.1257
25	H	-2.9383	1.1568	1.1529
26	H	1.8186	-0.2516	-2.2126
27	H	1.5403	-2.0143	-2.1933
28	H	0.1656	-0.8773	-2.1449
29	H	3.9483	-0.5202	-1.3305

Table S93 (Mol 11 (DHE), RAF pre-reaction complex (PRC), site 12 (M06-2X/ma-def2-SVP)) – Continued

#	Atom	X (Å)	Y (Å)	Z (Å)
30	H	4.6582	-0.5158	0.2799
31	H	3.9742	1.8244	-0.1132
32	H	-1.9102	1.4347	-1.0864
33	H	-2.7579	-0.0346	-1.6439
34	H	-4.0052	-1.0459	1.7608
35	H	-3.9706	-1.5162	0.0425
36	H	-4.9446	-0.1230	0.5572
37	H	1.1899	2.9588	1.4597
38	H	0.7685	3.1241	-0.2553
39	H	2.4341	3.4586	0.3024
40	H	-4.0195	1.8779	-2.0282
41	O	3.4535	1.0439	2.4266
42	H	2.5164	0.8196	2.5695

Charge = 0, Multiplicity = 2, E_{el} = -736.13095243 Ha

E_{ZPE} = 0.37428554 Ha

10 lowest real freq. (cm^{-1}): 25.4, 40.0, 55.5, 66.2, 85.5, 120.9, 128.2, 152.6, 179.3, 200.8

Table S94: Mol 11 (DHE), RAF pre-reaction complex (PRC), site 7 (M06-2X/ma-def2-SVP)

#	Atom	X (Å)	Y (Å)	Z (Å)
1	O	-4.0308	1.5138	-1.2863
2	C	1.1890	-1.0324	-0.1954
3	C	0.2125	-2.0840	0.3524
4	C	-1.5786	-0.3812	0.8477
5	C	-1.2494	-1.7028	0.1438
6	C	0.8025	0.3481	0.3203
7	C	2.6074	-1.3673	0.2958
8	C	-0.4484	0.6080	0.7624
9	C	-2.9108	0.2598	0.4009

Table S94 (Mol 11 (DHE), RAF pre-reaction complex (PRC), site 7 (M06-2X/ma-def2-SVP)) – Continued

#	Atom	X (Å)	Y (Å)	Z (Å)
10	C	1.1625	-1.0129	-1.7346
11	C	3.6369	-0.3284	-0.1371
12	C	1.8506	1.3954	0.3086
13	C	3.1408	1.0706	0.0861
14	C	-2.8519	0.7771	-1.0324
15	C	-4.0978	-0.6810	0.5937
16	C	1.4458	2.8248	0.5623
17	H	0.4006	-2.1955	1.4335
18	H	0.4292	-3.0557	-0.1213
19	H	-1.6945	-0.5982	1.9268
20	H	-1.4667	-1.6275	-0.9338
21	H	-1.9017	-2.4993	0.5314
22	H	2.8905	-2.3679	-0.0694
23	H	2.5836	-1.4238	1.3970
24	H	-0.6879	1.6160	1.1149
25	H	-3.0696	1.1481	1.0380
26	H	1.7995	-0.2127	-2.1387
27	H	1.5186	-1.9763	-2.1344
28	H	0.1448	-0.8375	-2.1109
29	H	3.8967	-0.4567	-1.2037
30	H	4.5819	-0.4752	0.4096
31	H	3.8820	1.8760	0.0599
32	H	-1.9555	1.4112	-1.1638
33	H	-2.7677	-0.0724	-1.7362
34	H	-4.1366	-1.0586	1.6266
35	H	-4.0297	-1.5470	-0.0835
36	H	-5.0389	-0.1595	0.3768
37	H	1.0289	2.9510	1.5740
38	H	0.6706	3.1492	-0.1488

Table S94 (Mol 11 (DHE), RAF pre-reaction complex (PRC), site 7 (M06-2X/ma-def2-SVP)) – Continued

#	Atom	X (Å)	Y (Å)	Z (Å)
39	H	2.3087	3.4957	0.4662
40	H	-4.0321	1.8156	-2.1989
41	O	0.7754	0.0376	2.9993
42	H	1.5901	0.4785	2.6953

Charge = 0, Multiplicity = 2, E_{el} = -736.12812726 Ha

E_{ZPE} = 0.37519213 Ha

10 lowest real freq. (cm^{-1}): 54.5, 111.4, 163.3, 182.9, 230.2, 241.6, 260.6, 266.9, 289.0, 307.5

Table S95: Mol 12 (ISO), RAF pre-reaction complex (PRC), site 12 (M06-2X/ma-def2-SVP)

#	Atom	X (Å)	Y (Å)	Z (Å)
1	O	3.5534	-0.0914	1.0752
2	C	0.8243	-1.0037	-0.0018
3	C	0.3907	0.4612	0.0131
4	C	-0.1938	-1.8012	-0.8498
5	C	2.2016	-1.1789	-0.6636
6	C	-2.0420	-0.1516	-0.4147
7	C	-1.0467	0.6671	0.4354
8	C	-1.6459	-1.6265	-0.4164
9	C	3.2231	-0.1087	-0.3025
10	C	1.2202	1.4759	-0.2990
11	C	2.6739	1.2608	-0.6593
12	C	0.8430	-1.5601	1.4371
13	C	-3.4806	0.1146	-0.0130
14	C	0.8399	2.9351	-0.3256
15	C	-3.8962	1.5627	0.0440
16	C	-4.3577	-0.8616	0.2668
17	H	0.0836	-2.8688	-0.8265
18	H	-0.1004	-1.4721	-1.8992

Table S95 (Mol 12 (ISO), RAF pre-reaction complex (PRC), site 12 (M06-2X/ma-def2-SVP)) – Continued

#	Atom	X (Å)	Y (Å)	Z (Å)
19	H	2.0825	-1.1442	-1.7599
20	H	2.5951	-2.1840	-0.4283
21	H	-1.9448	0.2254	-1.4528
22	H	-1.1549	0.3642	1.4954
23	H	-1.3103	1.7284	0.4002
24	H	-2.2994	-2.1917	-1.0989
25	H	-1.7983	-2.0490	0.5911
26	H	4.1425	-0.2841	-0.8917
27	H	2.8025	1.4362	-1.7424
28	H	3.2894	2.0228	-0.1534
29	H	1.5070	-0.9611	2.0734
30	H	-0.1533	-1.5590	1.8993
31	H	1.2017	-2.6029	1.4294
32	H	-0.2390	3.1205	-0.3551
33	H	1.2854	3.4184	-1.2094
34	H	1.2517	3.4526	0.5567
35	H	3.8190	-0.9767	1.3454
36	H	-3.4565	2.0644	0.9202
37	H	-3.5453	2.1050	-0.8481
38	H	-4.9871	1.6630	0.1080
39	H	-5.3939	-0.6244	0.5195
40	H	-4.0867	-1.9180	0.2315
41	O	-2.4145	-0.7580	2.9406
42	H	-3.1535	-0.7163	2.2970

Charge = 0, Multiplicity = 2, E_{el} = -736.11278231 Ha

E_{ZPE} = 0.37377996 Ha

10 lowest real freq. (cm^{-1}): 59.6, 107.5, 118.7, 142.8, 148.8, 161.2, 195.3, 214.6, 229.3, 241.8

Table S96: Mol 13 (LON), RAF pre-reaction complex (PRC), site 0 (M06-2X/ma-def2-SVP)

#	Atom	X (Å)	Y (Å)	Z (Å)
1	O	-3.3924	0.9627	0.7951
2	C	-0.1753	-1.3290	-0.1096
3	C	0.5124	0.7171	0.2393
4	C	-0.0125	-0.4280	1.1516
5	C	-0.2880	0.0198	-0.9021
6	C	2.0151	0.9592	0.0237
7	C	1.0800	-2.1483	-0.4544
8	C	-1.3832	0.0042	1.6760
9	C	2.7319	-0.2082	-0.6749
10	C	-1.6959	0.5398	-0.8302
11	C	-1.3675	-2.2816	-0.1799
12	C	2.4500	-1.5841	-0.0849
13	C	-2.2728	0.5527	0.5642
14	C	2.1579	2.2030	-0.8644
15	C	2.6663	1.2488	1.3824
16	C	-2.4321	0.9913	-1.8489
17	H	0.0880	1.6975	0.5238
18	H	0.6235	-0.7996	1.9689
19	H	0.1093	0.0489	-1.9286
20	H	1.0553	-2.3502	-1.5407
21	H	0.9750	-3.1303	0.0359
22	H	-1.2831	0.7960	2.4374
23	H	-1.9288	-0.8227	2.1608
24	H	3.8141	-0.0002	-0.6451
25	H	2.4554	-0.2175	-1.7443
26	H	2.5647	-1.5567	1.0109
27	H	3.2098	-2.2953	-0.4429
28	H	-2.3451	-1.7971	-0.0600
29	H	-1.3804	-2.7903	-1.1567
30	H	-1.2807	-3.0555	0.5987

Table S96 (Mol 13 (LON), RAF pre-reaction complex (PRC), site 0 (M06-2X/ma-def2-SVP)) – Continued

#	Atom	X (Å)	Y (Å)	Z (Å)
31	H	3.2165	2.3894	-1.1025
32	H	1.6150	2.0764	-1.8142
33	H	1.7601	3.0978	-0.3618
34	H	3.7114	1.5677	1.2480
35	H	2.1318	2.0573	1.9057
36	H	2.6662	0.3678	2.0402
37	H	-2.0465	0.9933	-2.8703
38	H	-3.4394	1.3707	-1.6654
39	O	-3.7392	0.5943	3.5524
40	H	-3.8796	0.8308	2.6061

Charge = 0, Multiplicity = 2, E_{el} = -734.90924386 Ha

E_{ZPE} = 0.35155543 Ha

10 lowest real freq. (cm^{-1}): 26.6, 61.7, 78.0, 102.1, 120.5, 151.9, 165.8, 200.3, 204.5, 228.3

Table S97: Mol 13 (LON), RAF pre-reaction complex (PRC), site 12 (M06-2X/ma-def2-SVP)

#	Atom	X (Å)	Y (Å)	Z (Å)
1	O	-3.3108	0.9214	0.7486
2	C	-0.0870	-1.3545	-0.1651
3	C	0.5953	0.6927	0.1862
4	C	0.0755	-0.4554	1.0976
5	C	-0.2054	-0.0047	-0.9548
6	C	2.0966	0.9402	-0.0314
7	C	1.1704	-2.1692	-0.5135
8	C	-1.2948	-0.0277	1.6262
9	C	2.8163	-0.2239	-0.7324
10	C	-1.6145	0.5109	-0.8782
11	C	-1.2763	-2.3110	-0.2336

Table S97 (Mol 13 (LON), RAF pre-reaction complex (PRC), site 12 (M06-2X/ma-def2-SVP)) – Continued

#	Atom	X (Å)	Y (Å)	Z (Å)
12	C	2.5389	-1.6015	-0.1443
13	C	-2.1888	0.5174	0.5171
14	C	2.2342	2.1857	-0.9179
15	C	2.7488	1.2302	1.3268
16	C	-2.3547	0.9623	-1.8943
17	H	0.1682	1.6713	0.4732
18	H	0.7149	-0.8268	1.9123
19	H	0.1894	0.0277	-1.9822
20	H	1.1449	-2.3689	-1.6002
21	H	1.0691	-3.1526	-0.0253
22	H	-1.1965	0.7636	2.3872
23	H	-1.8370	-0.8525	2.1162
24	H	3.8980	-0.0129	-0.7032
25	H	2.5388	-0.2324	-1.8015
26	H	2.6546	-1.5757	0.9514
27	H	3.3006	-2.3099	-0.5042
28	H	-2.2554	-1.8293	-0.1141
29	H	-1.2883	-2.8213	-1.2096
30	H	-1.1862	-3.0835	0.5459
31	H	3.2917	2.3757	-1.1576
32	H	1.6899	2.0589	-1.8669
33	H	1.8346	3.0786	-0.4133
34	H	3.7923	1.5539	1.1915
35	H	2.2116	2.0356	1.8522
36	H	2.7534	0.3481	1.9832
37	H	-1.9712	0.9683	-2.9165
38	H	-3.3630	1.3376	-1.7080
39	O	-4.1157	0.6576	3.4511
40	H	-3.9482	0.8090	2.4909

Table S97 (Mol 13 (LON), RAF pre-reaction complex (PRC), site 12 (M06-2X/ma-def2-SVP)) – Continued

#	Atom	X (Å)	Y (Å)	Z (Å)
Charge = 0, Multiplicity = 2, E_{el} = -734.87675738 Ha				
E_{ZPE} = 0.35176324 Ha				
10 lowest real freq. (cm^{-1}): 24.6, 46.7, 79.9, 102.4, 120.0, 151.9, 162.5, 200.4, 204.9, 228.5				

Table S98: Mol 13 (LON), RAF pre-reaction complex (PRC), site 15 (M06-2X/ma-def2-SVP)

#	Atom	X (Å)	Y (Å)	Z (Å)
1	O	-3.4915	0.0589	0.7271
2	C	-0.1636	-1.3674	-0.1342
3	C	0.4990	0.6775	0.2969
4	C	-0.0169	-0.5113	1.1567
5	C	-0.2947	0.0113	-0.8668
6	C	2.0003	0.9381	0.0942
7	C	1.1045	-2.1545	-0.5062
8	C	-1.3878	-0.1373	1.7191
9	C	2.7348	-0.1919	-0.6450
10	C	-1.7077	0.5029	-0.7863
11	C	-1.3445	-2.3278	-0.2513
12	C	2.4668	-1.5919	-0.1084
13	C	-2.2772	0.6357	0.6497
14	C	2.1328	2.2171	-0.7445
15	C	2.6438	1.1842	1.4655
16	C	-2.4654	0.8634	-1.8219
17	H	0.0651	1.6387	0.6159
18	H	0.6171	-0.9121	1.9641
19	H	0.0920	0.0833	-1.8958
20	H	1.0862	-2.3118	-1.6000
21	H	1.0081	-3.1574	-0.0578

Table S98 (Mol 13 (LON), RAF pre-reaction complex (PRC), site 15 (M06-2X/ma-def2-SVP)) – Continued

#	Atom	X (Å)	Y (Å)	Z (Å)
22	H	-1.2800	0.5499	2.5697
23	H	-1.9239	-1.0315	2.0663
24	H	3.8147	0.0277	-0.6043
25	H	2.4605	-0.1646	-1.7147
26	H	2.5758	-1.6045	0.9885
27	H	3.2361	-2.2812	-0.4889
28	H	-2.3219	-1.8504	-0.1154
29	H	-1.3427	-2.7940	-1.2496
30	H	-1.2503	-3.1368	0.4909
31	H	3.1901	2.4251	-0.9705
32	H	1.5941	2.1203	-1.7000
33	H	1.7194	3.0860	-0.2103
34	H	3.6871	1.5164	1.3484
35	H	2.0988	1.9691	2.0133
36	H	2.6478	0.2803	2.0921
37	H	-2.0785	0.8047	-2.8415
38	H	-3.4790	1.2424	-1.6788
39	O	-2.3182	2.0151	0.9395
40	H	-2.8836	2.1343	1.7132

Charge = 0, Multiplicity = 2, E_{el} = -734.93599471 Ha

E_{ZPE} = 0.35444063 Ha

10 lowest real freq. (cm^{-1}): 73.2, 102.4, 123.1, 152.4, 188.1, 198.0, 224.8, 229.4, 243.3, 251.4

11.6 RAF Adduct Radical (PostRC) Geometries (M06-2X/ma-def2-SVP)

Post-reaction complex (adduct radical) geometries for the RAF mechanism, optimized at the M06-2X/ma-def2-SVP level.

Table S99: Mol 1 (CAM), RAF adduct radical (PostRC), site 10 (M06-2X/ma-def2-SVP)

#	Atom	X (Å)	Y (Å)	Z (Å)
1	O	3.0249	0.2606	0.2873
2	C	0.0365	-1.3581	-0.5912
3	C	-1.2035	-0.5075	-0.1763
4	C	0.8156	0.7560	-0.7234
5	C	0.7056	-0.4684	-1.6578
6	C	1.1141	-1.3116	0.5085
7	C	1.6316	0.1536	0.4626
8	C	-0.6127	0.8871	-0.2803
9	C	-1.7326	-0.8146	1.2325
10	C	-2.3566	-0.7098	-1.1758
11	C	-1.2373	2.1196	0.2726
12	H	-0.2312	-2.3772	-0.9061
13	H	1.2749	1.6721	-1.1212
14	H	0.0889	-0.2753	-2.5454
15	H	1.6784	-0.8645	-1.9923
16	H	0.7568	-1.6161	1.4998
17	H	1.9447	-1.9861	0.2480
18	H	1.4203	0.6894	1.3986
19	H	-1.9803	-1.8843	1.3276
20	H	-1.0145	-0.5448	2.0175
21	H	-2.6491	-0.2377	1.4279
22	H	-3.1666	0.0100	-0.9817
23	H	-2.0302	-0.5753	-2.2162
24	H	-2.7766	-1.7230	-1.0732
25	H	-2.3338	2.0298	0.2597
26	H	-0.9728	2.9927	-0.3515
27	H	3.2865	-0.2264	-0.5018
28	O	-0.8987	2.3779	1.6361
29	H	0.0374	2.5982	1.6868

Charge = 0, Multiplicity = 2, E_{el} = -541.04798271 Ha

Table S99 (Mol 1 (CAM), RAF adduct radical (PostRC), site 10 (M06-2X/ma-def2-SVP)) – Continued

#	Atom	X (Å)	Y (Å)	Z (Å)

E_{ZPE} = 0.25758727 Ha

10 lowest real freq. (cm^{-1}): 46.8, 73.8, 102.5, 181.7, 218.2, 231.7, 236.2, 255.0, 267.1, 303.1

Table S100: Mol 1 (CAM), RAF adduct radical (PostRC), site 7 (M06-2X/ma-def2-SVP)

#	Atom	X (Å)	Y (Å)	Z (Å)
1	O	3.0187	0.1228	0.2466
2	C	-0.0228	-1.2229	-0.8497
3	C	-1.2333	-0.4865	-0.2023
4	C	0.8046	0.8447	-0.5371
5	C	0.6070	-0.1174	-1.7175
6	C	1.1003	-1.4444	0.1763
7	C	1.6279	-0.0103	0.4495
8	C	-0.6478	0.9896	-0.0235
9	C	-1.6656	-1.0949	1.1333
10	C	-2.4300	-0.5656	-1.1590
11	C	-1.3702	2.0423	-0.8058
12	H	-0.3265	-2.1401	-1.3755
13	H	1.2793	1.8155	-0.7347
14	H	-0.0537	0.2813	-2.5006
15	H	1.5492	-0.4317	-2.1940
16	H	0.7834	-1.9747	1.0826
17	H	1.9074	-2.0406	-0.2777
18	H	1.4539	0.3125	1.4822
19	H	-1.8741	-2.1690	1.0054
20	H	-0.9105	-0.9731	1.9182
21	H	-2.5926	-0.6207	1.4932
22	H	-3.2981	-0.0098	-0.7736
23	H	-2.1976	-0.1795	-2.1617
24	H	-2.7392	-1.6166	-1.2659

Table S100 (Mol 1 (CAM), RAF adduct radical (PostRC), site 7 (M06-2X/ma-def2-SVP)) – Continued

#	Atom	X (Å)	Y (Å)	Z (Å)
25	H	-2.4483	2.0097	-0.9603
26	H	-0.8534	2.9832	-1.0019
27	H	3.2380	-0.1583	-0.6483
28	O	-0.5888	1.4078	1.3406
29	H	-1.4651	1.6974	1.6178

Charge = 0, Multiplicity = 2, E_{el} = -541.04252704 Ha

E_{ZPE} = 0.25597942 Ha

10 lowest real freq. (cm^{-1}): 70.2, 146.0, 175.1, 238.6, 241.6, 261.0, 274.4, 300.9, 309.4, 319.5

Table S101: Mol 2 (IPO), RAF adduct radical (PostRC), site 10 (M06-2X/ma-def2-SVP)

#	Atom	X (Å)	Y (Å)	Z (Å)
1	O	-2.2555	0.8351	-1.4520
2	C	1.5576	0.2148	-0.9891
3	C	1.1028	1.3083	0.0247
4	C	0.7233	-0.7736	-0.0964
5	C	0.9941	0.1955	1.0934
6	C	-0.2981	1.8218	-0.3157
7	C	-0.7173	-0.6841	-0.4666
8	C	-1.3471	0.6794	-0.3687
9	C	3.0579	-0.0583	-0.9046
10	C	1.1542	0.3583	-2.4518
11	C	-1.6049	-1.8765	-0.4922
12	H	1.8015	2.1408	0.2052
13	H	1.0721	-1.8147	-0.0179
14	H	0.2017	0.2841	1.8508
15	H	1.9456	-0.0127	1.5957
16	H	-0.2878	2.3254	-1.2946
17	H	-0.6067	2.5724	0.4291
18	H	-1.9294	0.6902	0.5764

Table S101 (Mol 2 (IPO), RAF adduct radical (PostRC), site 10 (M06-2X/ma-def2-SVP)) – Continued

#	Atom	X (Å)	Y (Å)	Z (Å)
19	H	3.6196	0.7687	-1.3673
20	H	3.4167	-0.1700	0.1272
21	H	3.3101	-0.9808	-1.4506
22	H	1.4355	-0.5494	-3.0095
23	H	0.0749	0.5096	-2.5899
24	H	1.6835	1.2079	-2.9135
25	H	-2.4897	-1.6607	-1.1139
26	H	-1.0640	-2.7330	-0.9334
27	H	-2.8266	1.5899	-1.2758
28	O	-1.9969	-2.1807	0.8482
29	H	-2.6281	-2.9075	0.8339

Charge = 0, Multiplicity = 2, E_{el} = -541.02633125 Ha

E_{ZPE} = 0.25700874 Ha

10 lowest real freq. (cm^{-1}): 36.2, 51.7, 84.9, 175.1, 206.1, 213.0, 248.7, 253.6, 278.4, 292.2

Table S102: Mol 2 (IPO), RAF adduct radical (PostRC), site 6 (M06-2X/ma-def2-SVP)

#	Atom	X (Å)	Y (Å)	Z (Å)
1	O	-2.7302	0.5277	-0.6366
2	C	1.2684	-0.1218	-0.9679
3	C	0.8933	1.2876	-0.4213
4	C	0.6700	-0.6558	0.3734
5	C	1.0416	0.7045	1.0082
6	C	-0.5806	1.5892	-0.6990
7	C	-0.8616	-0.7901	0.3323
8	C	-1.4990	0.6062	0.0435
9	C	2.7832	-0.3360	-1.0167
10	C	0.6929	-0.5462	-2.3165
11	C	-1.3010	-1.8773	-0.5914
12	H	1.5451	2.1247	-0.7170

Table S102 (Mol 2 (IPO), RAF adduct radical (PostRC), site 6 (M06-2X/ma-def2-SVP)) – Continued

#	Atom	X (Å)	Y (Å)	Z (Å)
13	H	1.1149	-1.5757	0.7930
14	H	0.3804	1.0853	1.7988
15	H	2.0736	0.7332	1.3736
16	H	-0.7962	1.5523	-1.7769
17	H	-0.8343	2.6071	-0.3671
18	H	-1.6711	1.0154	1.0517
19	H	3.2215	0.2480	-1.8411
20	H	3.2920	-0.0405	-0.0900
21	H	3.0113	-1.3977	-1.2006
22	H	0.9244	-1.6030	-2.5221
23	H	-0.3941	-0.4245	-2.3970
24	H	1.1576	0.0562	-3.1144
25	H	-2.2486	-1.8189	-1.1244
26	H	-0.7055	-2.7900	-0.6608
27	H	-3.3862	0.1535	-0.0381
28	O	-1.3461	-1.0971	1.6577
29	H	-1.1486	-2.0211	1.8504

Charge = 0, Multiplicity = 2, E_{el} = -541.02308127 Ha

E_{ZPE} = 0.25563293 Ha

10 lowest real freq. (cm^{-1}): 74.2, 172.2, 181.3, 206.5, 214.6, 235.2, 292.0, 299.8, 315.4, 316.7

Table S103: Mol 3 (CYM), RAF adduct radical (PostRC), site 5 (M06-2X/ma-def2-SVP)

#	Atom	X (Å)	Y (Å)	Z (Å)
1	O	2.3432	-0.0875	1.1349
2	C	1.9845	-0.0684	-0.2607
3	C	0.4639	-0.0830	-0.3753
4	C	2.5629	1.2432	-0.7931
5	C	2.6562	-1.2513	-0.9519
6	C	-0.2777	1.1157	0.1778

Table S103 (Mol 3 (CYM), RAF adduct radical (PostRC), site 5 (M06-2X/ma-def2-SVP)) – Continued

#	Atom	X (Å)	Y (Å)	Z (Å)
7	C	-0.2332	-1.0824	-1.0020
8	C	-2.4190	-0.0484	-0.4866
9	C	-1.7683	0.9742	0.1343
10	C	-1.6454	-1.0840	-1.0918
11	C	-3.9226	-0.1285	-0.5355
12	H	3.6577	1.2232	-0.7034
13	H	2.2906	1.3808	-1.8490
14	H	2.1908	2.1008	-0.2157
15	H	3.7394	-1.1906	-0.7809
16	H	2.4693	-1.2419	-2.0346
17	H	2.2985	-2.2095	-0.5464
18	H	-0.0237	1.9726	-0.4925
19	H	0.3034	-1.9180	-1.4536
20	H	-2.3254	1.7703	0.6353
21	H	-2.1499	-1.9079	-1.5997
22	H	2.0396	-0.9208	1.5171
23	H	-4.2802	-1.0592	-0.0703
24	H	-4.3834	0.7172	-0.0098
25	H	-4.2802	-0.1220	-1.5763
26	O	0.0944	1.4819	1.4965
27	H	0.9957	1.1695	1.6638

Charge = 0, Multiplicity = 2, E_{el} = -539.86116712 Ha

E_{ZPE} = 0.23211001 Ha

10 lowest real freq. (cm^{-1}): 49.8, 74.2, 99.9, 140.6, 206.4, 222.8, 260.6, 291.0, 299.1, 315.5

Table S104: Mol 3 (CYM), RAF adduct radical (PostRC), site 6 (M06-2X/ma-def2-SVP)

#	Atom	X (Å)	Y (Å)	Z (Å)
1	O	2.4506	-1.4115	0.1899
2	C	1.9750	-0.2268	-0.4225

Table S104 (Mol 3 (CYM), RAF adduct radical (PostRC), site 6 (M06-2X/ma-def2-SVP)) – Continued

#	Atom	X (Å)	Y (Å)	Z (Å)
3	C	0.4465	-0.1774	-0.4006
4	C	2.5467	0.9962	0.3093
5	C	2.4947	-0.2807	-1.8592
6	C	-0.2698	0.7829	-1.0696
7	C	-0.2627	-1.2106	0.4338
8	C	-2.4268	-0.1416	-0.2909
9	C	-1.6797	0.8256	-1.0351
10	C	-1.7556	-1.1115	0.3912
11	C	-3.9308	-0.0584	-0.2883
12	H	3.6452	0.9484	0.2954
13	H	2.2215	1.9335	-0.1642
14	H	2.2091	1.0053	1.3567
15	H	3.5904	-0.3649	-1.8400
16	H	2.2168	0.6216	-2.4203
17	H	2.0850	-1.1608	-2.3736
18	H	0.2589	1.5426	-1.6512
19	H	0.0562	-2.2136	0.0978
20	H	-2.2110	1.6076	-1.5807
21	H	-2.3024	-1.8624	0.9683
22	H	2.1025	-1.4493	1.0925
23	H	-4.3733	-0.8483	0.3317
24	H	-4.2673	0.9146	0.0998
25	H	-4.3281	-0.1596	-1.3094
26	O	0.2114	-1.1621	1.7958
27	H	-0.1530	-0.3674	2.2059

Charge = 0, Multiplicity = 2, E_{el} = -539.86402456 Ha

E_{ZPE} = 0.23198621 Ha

10 lowest real freq. (cm^{-1}): 46.0, 88.5, 107.9, 142.6, 198.1, 205.8, 241.5, 273.9, 282.4, 305.2

Table S105: Mol 3 (CYM), RAF adduct radical (PostRC), site 7 (M06-2X/ma-def2-SVP)

#	Atom	X (Å)	Y (Å)	Z (Å)
1	O	2.6321	-0.6968	0.9460
2	C	2.0859	-0.0239	-0.1879
3	C	0.5660	0.0269	-0.0771
4	C	2.7281	1.3571	-0.1648
5	C	2.4889	-0.7646	-1.4695
6	C	-0.1794	1.2370	-0.0473
7	C	-0.1646	-1.1988	0.0326
8	C	-2.3809	-0.0003	0.1267
9	C	-1.5410	1.2458	0.0533
10	C	-1.5190	-1.2342	0.1396
11	C	-3.3675	-0.0516	-1.0447
12	H	3.8192	1.2362	-0.1905
13	H	2.4202	1.9562	-1.0326
14	H	2.4648	1.8904	0.7586
15	H	3.5853	-0.8154	-1.5339
16	H	2.0994	-0.2472	-2.3581
17	H	2.0949	-1.7920	-1.4763
18	H	0.3462	2.1911	-0.0942
19	H	0.3779	-2.1494	0.0355
20	H	-2.0891	2.1911	0.0880
21	H	-2.0482	-2.1859	0.2371
22	H	2.2288	-1.5672	1.0312
23	H	-4.0000	-0.9463	-0.9520
24	H	-4.0140	0.8374	-1.0171
25	H	-2.8299	-0.0821	-2.0018
26	O	-3.2068	0.0322	1.2961
27	H	-2.6314	0.0781	2.0693

Charge = 0, Multiplicity = 2, E_{el} = -539.85701578 Ha

E_{ZPE} = 0.23134311 Ha

10 lowest real freq. (cm^{-1}): 43.2, 47.6, 142.6, 191.0, 215.6, 236.4, 253.1, 262.0, 271.9, 323.6

Table S105 (Mol 3 (CYM), RAF adduct radical (PostRC), site 7 (M06-2X/ma-def2-SVP)) – Continued

#	Atom	X (Å)	Y (Å)	Z (Å)

Table S106: Mol 4 (MYR), RAF adduct radical (PostRC), site 6 (M06-2X/ma-def2-SVP)

#	Atom	X (Å)	Y (Å)	Z (Å)
1	O	3.0355	-0.7921	-1.0657
2	C	-1.3272	-0.6017	-0.0274
3	C	-1.6107	0.6362	-0.9298
4	C	0.0658	-0.6504	-0.7422
5	C	-0.6094	0.0492	-1.9482
6	C	-0.9906	1.9012	-0.3298
7	C	1.0839	0.3150	-0.1178
8	C	-2.1829	-1.8070	-0.4204
9	C	-1.4339	-0.4241	1.4870
10	C	0.4367	1.6603	0.0647
11	C	2.3555	0.4297	-0.9760
12	H	-2.6519	0.8034	-1.2462
13	H	0.5271	-1.6375	-0.8922
14	H	-0.0072	0.7729	-2.5173
15	H	-1.0612	-0.6589	-2.6512
16	H	-1.5797	2.2499	0.5394
17	H	-1.0499	2.7212	-1.0695
18	H	-2.1657	-2.0068	-1.4994
19	H	-1.8228	-2.7128	0.0919
20	H	-3.2312	-1.6457	-0.1239
21	H	-0.9104	0.4645	1.8682
22	H	-2.4919	-0.3158	1.7737
23	H	-1.0522	-1.3149	2.0132
24	H	1.0281	2.4582	0.5206
25	H	2.9922	1.2117	-0.5217
26	H	2.1013	0.7462	-1.9961

Table S106 (Mol 4 (MYR), RAF adduct radical (PostRC), site 6 (M06-2X/ma-def2-SVP)) – Continued

#	Atom	X (Å)	Y (Å)	Z (Å)
27	H	3.1365	-1.1212	-0.1630
28	O	1.6005	-0.2047	1.1148
29	H	0.8793	-0.3466	1.7350

Charge = 0, Multiplicity = 2, E_{el} = -541.03656489 Ha

E_{ZPE} = 0.25697972 Ha

10 lowest real freq. (cm^{-1}): 108.3, 131.3, 184.3, 205.1, 218.3, 235.4, 253.4, 288.0, 336.4, 346.2

Table S107: Mol 4 (MYR), RAF adduct radical (PostRC), site 9 (M06-2X/ma-def2-SVP)

#	Atom	X (Å)	Y (Å)	Z (Å)
1	O	2.6454	-1.0723	1.0900
2	C	-1.1866	-0.8253	-0.1778
3	C	-1.5391	0.6374	-0.5853
4	C	0.0445	-0.6554	-1.1486
5	C	-0.8168	0.3807	-1.9274
6	C	-0.7339	1.6515	0.2313
7	C	1.1424	0.0647	-0.4490
8	C	-2.2000	-1.8345	-0.7140
9	C	-0.8993	-1.1341	1.2854
10	C	0.7978	1.4029	0.1457
11	C	2.5024	-0.5011	-0.2015
12	H	-2.6073	0.9060	-0.6091
13	H	0.4005	-1.5549	-1.6733
14	H	-0.2842	1.2261	-2.3892
15	H	-1.4615	-0.0862	-2.6803
16	H	-1.0288	1.6016	1.2908
17	H	-0.9651	2.6692	-0.1206
18	H	-2.4441	-1.6800	-1.7734
19	H	-1.8106	-2.8583	-0.6035
20	H	-3.1383	-1.7696	-0.1406

Table S107 (Mol 4 (MYR), RAF adduct radical (PostRC), site 9 (M06-2X/ma-def2-SVP)) – Continued

#	Atom	X (Å)	Y (Å)	Z (Å)
21	H	-0.1812	-0.4410	1.7420
22	H	-1.8313	-1.0998	1.8726
23	H	-0.4758	-2.1459	1.3797
24	H	1.2506	2.1870	-0.4972
25	H	3.2649	0.2883	-0.3488
26	H	2.7128	-1.3066	-0.9194
27	H	2.4167	-0.3783	1.7225
28	O	1.4267	1.4879	1.4273
29	H	1.4141	2.4000	1.7355

Charge = 0, Multiplicity = 2, E_{el} = -541.02665625 Ha

E_{ZPE} = 0.25770839 Ha

10 lowest real freq. (cm^{-1}): 62.9, 68.5, 143.3, 172.5, 206.0, 216.5, 248.9, 285.6, 298.1, 309.5

Table S108: Mol 5 (VER), RAF adduct radical (PostRC), site 5 (M06-2X/ma-def2-SVP)

#	Atom	X (Å)	Y (Å)	Z (Å)
1	O	1.1531	2.6267	-0.6848
2	C	-1.2277	0.0401	-0.9066
3	C	-0.5778	-0.8155	0.2331
4	C	-0.7613	1.2803	-0.0760
5	C	-0.8317	0.3791	1.1832
6	C	0.9418	-0.9902	0.0931
7	C	-0.7640	-0.0543	-2.3587
8	C	-2.7498	-0.1139	-0.8899
9	C	0.7042	1.5351	-0.3729
10	C	1.5420	0.3448	-0.2804
11	C	1.3326	-2.0993	-0.8835
12	H	-1.0417	-1.7852	0.4714
13	H	-1.3368	2.2139	-0.1345
14	H	-1.8302	0.3665	1.6322

Table S108 (Mol 5 (VER), RAF adduct radical (PostRC), site 5 (M06-2X/ma-def2-SVP)) – Continued

#	Atom	X (Å)	Y (Å)	Z (Å)
15	H	-0.0848	0.5446	1.9704
16	H	-1.0277	-1.0325	-2.7905
17	H	-1.2785	0.7178	-2.9513
18	H	0.3145	0.1016	-2.4937
19	H	-3.0316	-1.0890	-1.3168
20	H	-3.1778	-0.0540	0.1186
21	H	-3.2173	0.6709	-1.5037
22	H	2.6154	0.4461	-0.4630
23	H	0.9732	-1.9088	-1.9011
24	H	0.9124	-3.0516	-0.5293
25	H	2.4294	-2.1990	-0.9259
26	O	1.3992	-1.3540	1.3985
27	H	2.3394	-1.5632	1.3594

Charge = 0, Multiplicity = 2, E_{el} = -539.84447234 Ha

E_{ZPE} = 0.23281633 Ha

10 lowest real freq. (cm^{-1}): 111.6, 146.4, 183.2, 210.0, 210.6, 229.2, 258.7, 280.8, 308.9, 322.5

Table S109: Mol 6 (CRV), RAF adduct radical (PostRC), site 10 (M06-2X/ma-def2-SVP)

#	Atom	X (Å)	Y (Å)	Z (Å)
1	O	-2.1505	-1.9218	0.2575
2	C	0.8613	0.2311	-0.5155
3	C	0.0773	-0.9895	-0.0003
4	C	-1.3983	-0.9034	-0.3780
5	C	0.3154	1.4806	0.1798
6	C	-1.9796	0.4748	-0.1137
7	C	-1.1885	1.5271	0.1402
8	C	2.3479	0.0510	-0.3909
9	C	-3.4771	0.5830	-0.1934
10	C	3.0149	-0.9780	-1.2473

Table S109 (Mol 6 (CRV), RAF adduct radical (PostRC), site 10 (M06-2X/ma-def2-SVP)) – Continued

#	Atom	X (Å)	Y (Å)	Z (Å)
11	C	3.0837	0.6267	0.7766
12	H	0.6299	0.3196	-1.5960
13	H	0.1666	-1.0178	1.1013
14	H	0.4922	-1.9311	-0.3917
15	H	-1.5094	-1.1190	-1.4561
16	H	0.7255	2.3892	-0.2913
17	H	0.6543	1.4947	1.2310
18	H	-1.6590	2.4988	0.3251
19	H	-3.8366	0.2223	-1.1706
20	H	-3.8064	1.6217	-0.0602
21	H	-3.9660	-0.0444	0.5656
22	H	3.0363	-1.9781	-0.7696
23	H	2.4914	-1.1015	-2.2075
24	H	4.0620	-0.7105	-1.4582
25	H	-2.0742	-1.8064	1.2119
26	H	4.1703	0.4779	0.6494
27	H	2.9128	1.7110	0.8608
28	O	2.6537	0.0956	2.0298
29	H	2.7718	-0.8605	2.0164

Charge = 0, Multiplicity = 2, E_{el} = -541.03773208 Ha

E_{ZPE} = 0.25568008 Ha

10 lowest real freq. (cm^{-1}): 42.6, 70.9, 87.1, 97.1, 123.1, 140.5, 166.9, 195.6, 273.3, 285.1

Table S110: Mol 6 (CRV), RAF adduct radical (PostRC), site 6 (M06-2X/ma-def2-SVP)

#	Atom	X (Å)	Y (Å)	Z (Å)
1	O	-2.0840	-1.9457	0.4286
2	C	0.8848	0.1804	-0.4290
3	C	0.1731	-1.1068	0.0113
4	C	-1.3118	-1.0592	-0.3749

Table S110 (Mol 6 (CRV), RAF adduct radical (PostRC), site 6 (M06-2X/ma-def2-SVP)) – Continued

#	Atom	X (Å)	Y (Å)	Z (Å)
5	C	0.3379	1.3504	0.3960
6	C	-1.8589	0.3410	-0.3204
7	C	-1.1788	1.2629	0.6421
8	C	2.3967	0.0648	-0.4252
9	C	-3.2513	0.5895	-0.7896
10	C	2.9674	-1.0598	-1.2529
11	C	3.2019	0.9117	0.2268
12	H	0.6042	0.3496	-1.4882
13	H	0.2601	-1.1970	1.1074
14	H	0.6257	-2.0072	-0.4251
15	H	-1.4336	-1.4431	-1.4029
16	H	0.5823	2.3023	-0.0986
17	H	0.8138	1.3645	1.3879
18	H	-1.6196	2.2725	0.5834
19	H	-3.4505	0.0635	-1.7358
20	H	-3.4456	1.6623	-0.9368
21	H	-3.9997	0.2085	-0.0681
22	H	2.8036	-2.0354	-0.7703
23	H	2.4853	-1.1043	-2.2425
24	H	4.0480	-0.9352	-1.3984
25	H	-1.9542	-1.6742	1.3466
26	H	4.2871	0.8023	0.1674
27	H	2.8244	1.7354	0.8328
28	O	-1.3628	0.7596	1.9829
29	H	-2.2889	0.8725	2.2251

Charge = 0, Multiplicity = 2, E_{el} = -541.03543434 Ha

E_{ZPE} = 0.25578621 Ha

10 lowest real freq. (cm^{-1}): 44.0, 55.7, 72.8, 97.8, 109.9, 159.0, 162.5, 200.6, 265.5, 299.1

Table S111: Mol 6 (CRV), RAF adduct radical (PostRC), site 7 (M06-2X/ma-def2-SVP)

#	Atom	X (Å)	Y (Å)	Z (Å)
1	O	-2.0866	-1.8705	-0.3679
2	C	0.9264	0.4092	-0.3592
3	C	0.1246	-0.8860	-0.2065
4	C	-1.3182	-0.7208	-0.6716
5	C	0.2939	1.4922	0.5171
6	C	-1.9556	0.5441	-0.1268
7	C	-1.2070	1.5175	0.4095
8	C	2.4026	0.1672	-0.0615
9	C	-3.4488	0.6455	-0.2645
10	C	3.1418	-0.7125	-1.0443
11	C	3.1883	1.1082	0.7566
12	H	0.8599	0.7354	-1.4134
13	H	0.1316	-1.1730	0.8610
14	H	0.5763	-1.7172	-0.7662
15	H	-1.3408	-0.6653	-1.7750
16	H	0.6871	2.4837	0.2408
17	H	0.5850	1.3236	1.5702
18	H	-1.7101	2.4137	0.7883
19	H	-3.7458	0.5176	-1.3178
20	H	-3.8139	1.6191	0.0877
21	H	-3.9583	-0.1501	0.2977
22	H	2.7091	-1.7221	-1.0712
23	H	3.0917	-0.2867	-2.0571
24	H	4.1958	-0.8057	-0.7524
25	H	-2.0554	-2.0167	0.5849
26	H	4.2733	1.1509	0.6124
27	H	2.7345	2.0196	1.1558
28	O	2.6975	-0.1008	1.2992
29	H	1.1471	-1.8122	5.2750

Charge = 0, Multiplicity = 2, E_{el} = -540.96517268 Ha

Table S111 (Mol 6 (CRV), RAF adduct radical (PostRC), site 7 (M06-2X/ma-def2-SVP)) – Continued

#	Atom	X (Å)	Y (Å)	Z (Å)

$E_{\mathrm{ZPE}} = 0.24665645$ Ha

10 lowest real freq. (cm^{-1}): 30.7, 47.5, 78.4, 121.2, 154.4, 166.7, 187.0, 242.6, 288.8, 298.9

Table S112: Mol 8 (CAR), RAF adduct radical (PostRC), site 11 (M06-2X/ma-def2-SVP)

#	Atom	X (Å)	Y (Å)	Z (Å)
1	O	0.2871	-0.8351	-1.5903
2	C	0.3819	-0.1771	-0.3368
3	C	-0.2612	1.2805	-0.3785
4	C	1.9025	0.0487	-0.0308
5	C	0.8309	2.1653	0.2535
6	C	2.1438	1.5518	-0.2098
7	C	-0.2454	-1.0543	0.7587
8	C	-1.5662	1.4377	0.4188
9	C	2.8801	-0.8503	-0.8025
10	C	-0.4909	1.7363	-1.8271
11	C	-1.6503	-1.5985	0.4320
12	C	-2.7866	0.6958	-0.1213
13	C	-2.5521	-0.7545	-0.4244
14	C	4.3270	-0.4418	-0.5147
15	C	2.6908	-2.3348	-0.4864
16	C	-3.6395	-1.4555	-1.1747
17	H	2.0385	-0.1691	1.0463
18	H	0.7678	2.1003	1.3541
19	H	0.7075	3.2251	-0.0190
20	H	2.3365	1.7848	-1.2706
21	H	3.0082	1.9121	0.3652
22	H	-0.2438	-0.4896	1.7019
23	H	0.4195	-1.9134	0.9278
24	H	-1.8091	2.5155	0.4495

Table S112 (Mol 8 (CAR), RAF adduct radical (PostRC), site 11 (M06-2X/ma-def2-SVP)) – Continued

#	Atom	X (Å)	Y (Å)	Z (Å)
25	H	-1.4202	1.1343	1.4657
26	H	2.6809	-0.7036	-1.8767
27	H	-0.8735	2.7691	-1.8280
28	H	0.4370	1.7064	-2.4127
29	H	-1.2209	1.1076	-2.3566
30	H	-2.1745	-1.8195	1.3835
31	H	-1.5423	-2.5753	-0.0667
32	H	-0.6460	-0.9385	-1.8127
33	H	-3.1180	1.1800	-1.0605
34	H	4.5408	-0.4987	0.5661
35	H	5.0274	-1.1170	-1.0281
36	H	4.5475	0.5813	-0.8501
37	H	2.8369	-2.5252	0.5906
38	H	3.4303	-2.9405	-1.0313
39	H	1.6913	-2.6830	-0.7754
40	H	-4.4485	-1.7630	-0.4850
41	H	-3.2732	-2.3667	-1.6719
42	H	-4.0989	-0.8002	-1.9295
43	O	-3.8639	0.7565	0.8171
44	H	-4.0741	1.6805	0.9930

Charge = 0, Multiplicity = 2, E_{el} = -737.38779578 Ha

E_{ZPE} = 0.40070095 Ha

10 lowest real freq. (cm^{-1}): 41.6, 68.0, 96.6, 114.6, 124.0, 129.9, 164.2, 200.1, 218.2, 241.5

Table S113: Mol 9 (CAO), RAF adduct radical (PostRC), site 15 (M06-2X/ma-def2-SVP)

#	Atom	X (Å)	Y (Å)	Z (Å)
1	O	3.0022	-1.4322	-0.3794
2	C	-1.0772	-0.6291	-0.1411
3	C	-2.6018	-0.3456	-0.3019

Table S113 (Mol 9 (CAO), RAF adduct radical (PostRC), site 15 (M06-2X/ma-def2-SVP)) – Continued

#	Atom	X (Å)	Y (Å)	Z (Å)
4	C	-0.9129	0.8073	0.4389
5	C	-2.2637	1.1634	-0.2564
6	C	1.8115	-1.2243	0.3712
7	C	-0.6318	-1.8624	0.6449
8	C	2.0576	-0.5222	-0.9018
9	C	0.7961	-2.3465	0.3459
10	C	0.2724	1.6781	0.1960
11	C	2.3553	0.9448	-1.0399
12	C	-3.2353	-0.8722	-1.5811
13	C	-3.4231	-0.7700	0.9145
14	C	1.0162	1.7113	-1.1066
15	C	2.0363	-0.5917	1.7239
16	C	0.5177	2.8143	1.1478
17	H	-0.6486	-0.6613	-1.1576
18	H	-1.0907	0.7694	1.5259
19	H	-2.9516	1.8288	0.2866
20	H	-2.0918	1.5658	-1.2678
21	H	-1.3211	-2.6920	0.4135
22	H	-0.7546	-1.6699	1.7242
23	H	1.5182	-0.9253	-1.7718
24	H	1.0857	-3.1286	1.0668
25	H	0.8316	-2.8073	-0.6536
26	H	2.9402	1.1173	-1.9571
27	H	2.9634	1.3047	-0.1969
28	H	-3.2548	-1.9746	-1.5831
29	H	-4.2738	-0.5196	-1.6839
30	H	-2.6738	-0.5406	-2.4677
31	H	-4.4467	-0.3726	0.8335
32	H	-3.4944	-1.8664	0.9858
33	H	-2.9940	-0.3969	1.8561

Table S113 (Mol 9 (CAO), RAF adduct radical (PostRC), site 15 (M06-2X/ma-def2-SVP)) – Continued

#	Atom	X (Å)	Y (Å)	Z (Å)
34	H	0.4027	1.2818	-1.9177
35	H	1.2200	2.7556	-1.3985
36	H	2.5183	-1.3289	2.3842
37	H	2.6946	0.2846	1.6585
38	H	1.0783	-0.3008	2.1788
39	H	-0.2362	3.6116	0.9974
40	H	1.5011	3.2730	0.9292
41	O	0.4168	2.4686	2.5133
42	H	1.0773	1.7942	2.7050

Charge = 0, Multiplicity = 2, E_{el} = -736.13286094 Ha

E_{ZPE} = 0.37738978 Ha

10 lowest real freq. (cm^{-1}): 62.8, 73.1, 80.9, 94.1, 136.3, 149.2, 178.3, 189.8, 213.7, 215.0

Table S114: Mol 9 (CAO), RAF adduct radical (PostRC), site 9 (M06-2X/ma-def2-SVP)

#	Atom	X (Å)	Y (Å)	Z (Å)
1	O	2.9389	-1.6659	-0.3340
2	C	-1.0086	-0.5552	-0.4460
3	C	-2.4678	-0.2441	-0.9034
4	C	-0.9534	0.8843	0.1522
5	C	-2.0997	1.2539	-0.8106
6	C	1.7415	-1.4808	0.4166
7	C	-0.7565	-1.8183	0.3821
8	C	2.0791	-0.6187	-0.7306
9	C	0.6207	-2.4684	0.1817
10	C	0.2540	1.8543	0.2423
11	C	2.4939	0.8217	-0.6597
12	C	-2.8502	-0.7793	-2.2754
13	C	-3.5265	-0.6262	0.1297
14	C	1.3029	1.7611	-0.8988

Table S114 (Mol 9 (CAO), RAF adduct radical (PostRC), site 9 (M06-2X/ma-def2-SVP)) – Continued

#	Atom	X (Å)	Y (Å)	Z (Å)
15	C	1.9799	-1.1083	1.8578
16	C	-0.3169	3.2449	0.2693
17	H	-0.4101	-0.5830	-1.3692
18	H	-1.3482	0.8462	1.1803
19	H	-2.8687	1.9538	-0.4488
20	H	-1.7075	1.6220	-1.7737
21	H	-1.5186	-2.5668	0.1082
22	H	-0.9256	-1.5988	1.4493
23	H	1.5605	-0.8543	-1.6703
24	H	0.7275	-3.3353	0.8535
25	H	0.7052	-2.8461	-0.8496
26	H	3.2466	1.0101	-1.4417
27	H	2.9716	1.0388	0.3045
28	H	-2.8871	-1.8811	-2.2689
29	H	-3.8434	-0.4140	-2.5814
30	H	-2.1218	-0.4686	-3.0396
31	H	-4.4957	-0.1818	-0.1454
32	H	-3.6635	-1.7173	0.1778
33	H	-3.2706	-0.2748	1.1400
34	H	0.7881	1.4907	-1.8380
35	H	1.7092	2.7718	-1.0617
36	H	2.3283	-2.0079	2.3889
37	H	2.7467	-0.3313	1.9515
38	H	1.0642	-0.7384	2.3346
39	H	-0.9087	3.5463	1.1364
40	H	-0.3741	3.8467	-0.6386
41	O	0.8652	1.6013	1.5060
42	H	1.4536	2.3368	1.7141

Charge = 0, Multiplicity = 2, E_{el} = -736.11717104 Ha

Table S114 (Mol 9 (CAO), RAF adduct radical (PostRC), site 9 (M06-2X/ma-def2-SVP)) – Continued

#	Atom	X (Å)	Y (Å)	Z (Å)

$E_{\text{ZPE}} = 0.37536957$ Ha

10 lowest real freq. (cm^{-1}): 67.1, 89.7, 116.0, 142.4, 171.4, 184.6, 193.9, 212.7, 222.6, 224.0

Table S115: Mol 11 (DHE), RAF adduct radical (PostRC), site 11 (M06-2X/ma-def2-SVP)

#	Atom	X (Å)	Y (Å)	Z (Å)
1	O	-4.0241	1.4834	-1.5232
2	C	1.1519	-1.0760	-0.5217
3	C	0.2026	-2.1147	0.0957
4	C	-1.5676	-0.3929	0.6249
5	C	-1.2679	-1.7365	-0.0511
6	C	0.7981	0.3080	0.0124
7	C	2.5979	-1.4017	-0.1060
8	C	-0.4189	0.5777	0.5041
9	C	-2.8955	0.2561	0.1763
10	C	1.0287	-1.0954	-2.0572
11	C	3.6109	-0.4389	-0.7320
12	C	1.9229	1.3582	0.0796
13	C	3.0841	0.9555	-0.7809
14	C	-2.8334	0.7699	-1.2580
15	C	-4.0883	-0.6763	0.3736
16	C	1.4496	2.7595	-0.3059
17	H	0.4457	-2.2124	1.1683
18	H	0.3937	-3.0976	-0.3656
19	H	-1.6886	-0.5882	1.7084
20	H	-1.5346	-1.6947	-1.1187
21	H	-1.9024	-2.5206	0.3883
22	H	2.8329	-2.4409	-0.3880
23	H	2.6624	-1.3395	0.9916
24	H	-0.6305	1.5824	0.8907

Table S115 (Mol 11 (DHE), RAF adduct radical (PostRC), site 11 (M06-2X/ma-def2-SVP)) – Continued

#	Atom	X (Å)	Y (Å)	Z (Å)
25	H	-3.0487	1.1480	0.8104
26	H	1.6375	-0.3086	-2.5253
27	H	1.3616	-2.0693	-2.4524
28	H	-0.0106	-0.9304	-2.3738
29	H	3.8767	-0.7665	-1.7530
30	H	4.5572	-0.4713	-0.1615
31	H	3.6748	1.7415	-1.2563
32	H	-1.9481	1.4195	-1.3827
33	H	-2.7277	-0.0802	-1.9586
34	H	-4.1274	-1.0521	1.4075
35	H	-4.0276	-1.5441	-0.3020
36	H	-5.0266	-0.1495	0.1579
37	H	0.6609	3.1207	0.3680
38	H	1.0627	2.7688	-1.3354
39	H	2.2966	3.4558	-0.2329
40	H	-4.0112	1.8056	-2.4288
41	O	2.4340	1.3831	1.4288
42	H	1.6831	1.3340	2.0321

Charge = 0, Multiplicity = 2, E_{el} = -736.16790957 Ha

E_{ZPE} = 0.37677579 Ha

10 lowest real freq. (cm^{-1}): 29.0, 55.6, 73.3, 95.4, 131.6, 158.0, 183.2, 185.1, 231.2, 241.7

Table S116: Mol 11 (DHE), RAF adduct radical (PostRC), site 12 (M06-2X/ma-def2-SVP)

#	Atom	X (Å)	Y (Å)	Z (Å)
1	O	-4.1227	1.2678	-1.5079
2	C	1.1028	-1.0254	-0.4497
3	C	0.2353	-2.0327	0.3234
4	C	-1.5708	-0.3142	0.7595
5	C	-1.2550	-1.7222	0.2322

Table S116 (Mol 11 (DHE), RAF adduct radical (PostRC), site 12 (M06-2X/ma-def2-SVP)) – Continued

#	Atom	X (Å)	Y (Å)	Z (Å)
6	C	0.8117	0.3875	0.0654
7	C	2.5838	-1.3715	-0.2369
8	C	-0.4575	0.6721	0.5401
9	C	-2.9297	0.2462	0.2816
10	C	0.7599	-1.0826	-1.9502
11	C	3.5142	-0.3166	-0.8216
12	C	1.8210	1.3727	0.0454
13	C	3.2768	1.0379	-0.1737
14	C	-2.9219	0.5875	-1.2042
15	C	-4.0908	-0.6836	0.6250
16	C	1.5315	2.7889	0.4601
17	H	0.5492	-2.0201	1.3816
18	H	0.4377	-3.0475	-0.0575
19	H	-1.6711	-0.3997	1.8610
20	H	-1.5899	-1.8303	-0.8111
21	H	-1.8282	-2.4643	0.8080
22	H	2.7906	-2.3583	-0.6829
23	H	2.7810	-1.4559	0.8445
24	H	-0.6743	1.6869	0.8861
25	H	-3.0872	1.2037	0.8098
26	H	1.3789	-0.3848	-2.5302
27	H	0.9223	-2.0998	-2.3434
28	H	-0.2868	-0.8035	-2.1333
29	H	3.3742	-0.2300	-1.9092
30	H	4.5668	-0.5869	-0.6549
31	H	3.7300	1.8281	-0.8032
32	H	-2.0425	1.2164	-1.4346
33	H	-2.8407	-0.3395	-1.8035
34	H	-4.0963	-0.9277	1.6983
35	H	-4.0205	-1.6276	0.0619

Table S116 (Mol 11 (DHE), RAF adduct radical (PostRC), site 12 (M06-2X/ma-def2-SVP)) – Continued

#	Atom	X (Å)	Y (Å)	Z (Å)
36	H	-5.0485	-0.2117	0.3697
37	H	1.2874	2.8703	1.5348
38	H	0.6698	3.2043	-0.0856
39	H	2.3932	3.4430	0.2608
40	H	-4.1544	1.4607	-2.4491
41	O	3.9680	1.0143	1.0796
42	H	3.7047	1.7788	1.6027

Charge = 0, Multiplicity = 2, E_{el} = -736.18918437 Ha

E_{ZPE} = 0.37772995 Ha

10 lowest real freq. (cm^{-1}): 39.9, 54.2, 73.0, 78.2, 110.3, 148.6, 169.5, 173.6, 194.6, 228.6

Table S117: Mol 11 (DHE), RAF adduct radical (PostRC), site 7 (M06-2X/ma-def2-SVP)

#	Atom	X (Å)	Y (Å)	Z (Å)
1	O	-4.3366	1.2901	-1.4291
2	C	1.4494	-0.9916	-0.4485
3	C	0.3175	-2.0240	-0.3371
4	C	-1.4043	-0.3262	0.2626
5	C	-1.0419	-1.4490	-0.7061
6	C	1.0876	0.3370	0.2029
7	C	2.6974	-1.5636	0.2466
8	C	-0.3556	0.8027	0.2698
9	C	-2.8271	0.2374	0.0805
10	C	1.7586	-0.7421	-1.9420
11	C	3.8826	-0.6078	0.1849
12	C	2.1519	1.2319	0.5064
13	C	3.4644	0.8012	0.4608
14	C	-3.1542	0.5195	-1.3818
15	C	-3.8891	-0.6705	0.6991
16	C	1.9075	2.6941	0.8256

Table S117 (Mol 11 (DHE), RAF adduct radical (PostRC), site 7 (M06-2X/ma-def2-SVP)) – Continued

#	Atom	X (Å)	Y (Å)	Z (Å)
17	H	0.2723	-2.3971	0.7010
18	H	0.5674	-2.8887	-0.9733
19	H	-1.3621	-0.7650	1.2790
20	H	-1.0284	-1.0767	-1.7443
21	H	-1.8112	-2.2363	-0.6702
22	H	2.9551	-2.5351	-0.2064
23	H	2.4395	-1.7558	1.3014
24	H	-0.5513	1.4648	-0.5988
25	H	-2.8616	1.2085	0.6003
26	H	2.5385	0.0219	-2.0648
27	H	2.1004	-1.6744	-2.4209
28	H	0.8689	-0.3884	-2.4826
29	H	4.3692	-0.6585	-0.8076
30	H	4.6625	-0.9163	0.9004
31	H	4.2554	1.5363	0.6330
32	H	-2.3179	1.0612	-1.8633
33	H	-3.2921	-0.4345	-1.9261
34	H	-3.7320	-0.7757	1.7827
35	H	-3.8653	-1.6780	0.2529
36	H	-4.8915	-0.2525	0.5360
37	H	1.5846	2.8431	1.8638
38	H	1.1182	3.1231	0.1939
39	H	2.8328	3.2641	0.6674
40	H	-4.5922	1.4340	-2.3446
41	O	-0.5902	1.6320	1.3963
42	H	-0.3346	1.1370	2.1841

Charge = 0, Multiplicity = 2, E_{el} = -736.18494001 Ha

E_{ZPE} = 0.37808005 Ha

10 lowest real freq. (cm^{-1}): 42.5, 51.6, 79.0, 104.6, 146.3, 163.2, 189.2, 195.5, 218.1, 224.6

Table S118: Mol 12 (ISO), RAF adduct radical (PostRC), site 12 (M06-2X/ma-def2-SVP)

#	Atom	X (Å)	Y (Å)	Z (Å)
1	O	3.6186	-0.1136	0.8955
2	C	0.8983	-1.0688	-0.1466
3	C	0.4313	0.3853	-0.0884
4	C	-0.1237	-1.8695	-0.9868
5	C	2.2643	-1.1981	-0.8413
6	C	-2.0013	-0.2646	-0.4618
7	C	-1.0004	0.5486	0.3756
8	C	-1.5689	-1.7310	-0.5204
9	C	3.2667	-0.1114	-0.4777
10	C	1.2281	1.4241	-0.4052
11	C	2.6786	1.2505	-0.7995
12	C	0.9634	-1.6569	1.2782
13	C	-3.4643	-0.1278	0.0447
14	C	0.8118	2.8738	-0.4054
15	C	-3.9036	1.3455	0.1131
16	C	-4.3921	-0.8714	-0.8634
17	H	0.1761	-2.9317	-0.9921
18	H	-0.0568	-1.5166	-2.0307
19	H	2.1192	-1.1430	-1.9335
20	H	2.6859	-2.1984	-0.6367
21	H	-1.9821	0.1464	-1.4890
22	H	-1.0596	0.2085	1.4271
23	H	-1.2798	1.6065	0.3794
24	H	-2.2287	-2.2879	-1.2037
25	H	-1.7139	-2.1822	0.4737
26	H	4.1803	-0.2531	-1.0845
27	H	2.7816	1.4463	-1.8819
28	H	3.2861	2.0186	-0.2932
29	H	1.6100	-1.0463	1.9215
30	H	-0.0290	-1.7119	1.7451

Table S118 (Mol 12 (ISO), RAF adduct radical (PostRC), site 12 (M06-2X/ma-def2-SVP)) – Continued

#	Atom	X (Å)	Y (Å)	Z (Å)
31	H	1.3623	-2.6841	1.2425
32	H	-0.2717	3.0328	-0.4015
33	H	1.2179	3.3750	-1.2983
34	H	1.2381	3.3950	0.4678
35	H	3.9360	-0.9906	1.1353
36	H	-3.3421	1.8945	0.8851
37	H	-3.7434	1.8480	-0.8522
38	H	-4.9716	1.3986	0.3660
39	H	-4.3579	-0.6884	-1.9378
40	H	-5.2180	-1.4378	-0.4342
41	O	-3.6090	-0.7193	1.3316
42	H	-3.1402	-0.1788	1.9765

Charge = 0, Multiplicity = 2, E_{el} = -736.17000827 Ha

E_{ZPE} = 0.37529907 Ha

10 lowest real freq. (cm^{-1}): 45.8, 54.0, 79.4, 84.0, 123.2, 141.9, 164.9, 167.9, 183.6, 227.3

Table S119: Mol 13 (LON), RAF adduct radical (PostRC), site 0 (M06-2X/ma-def2-SVP)

#	Atom	X (Å)	Y (Å)	Z (Å)
1	O	-3.5394	0.6335	0.4031
2	C	-0.1423	-1.3673	-0.5859
3	C	0.5050	0.6386	-0.0032
4	C	-0.0220	-0.6065	0.7694
5	C	-0.2800	0.0621	-1.2187
6	C	2.0072	0.9231	-0.1615
7	C	1.1388	-2.1156	-0.9967
8	C	-1.4126	-0.2456	1.3106
9	C	2.7606	-0.1445	-0.9703
10	C	-1.7103	0.5459	-1.0970
11	C	-1.2993	-2.3469	-0.7778

Table S119 (Mol 13 (LON), RAF adduct radical (PostRC), site 0 (M06-2X/ma-def2-SVP)) – Continued

#	Atom	X (Å)	Y (Å)	Z (Å)
12	C	2.4922	-1.5796	-0.5386
13	C	-2.2204	0.3928	0.2170
14	C	2.1441	2.2608	-0.9022
15	C	2.6292	1.0711	1.2336
16	C	-2.4213	1.0573	-2.1456
17	H	0.0609	1.5738	0.3788
18	H	0.5998	-1.0561	1.5588
19	H	0.1141	0.2169	-2.2350
20	H	1.1378	-2.1900	-2.0995
21	H	1.0454	-3.1504	-0.6272
22	H	-1.3166	0.4377	2.1706
23	H	-1.9453	-1.1410	1.6819
24	H	3.8383	0.0778	-0.8983
25	H	2.4998	-0.0417	-2.0389
26	H	2.5838	-1.6690	0.5562
27	H	3.2702	-2.2380	-0.9551
28	H	-2.2907	-1.9033	-0.6277
29	H	-1.2750	-2.7565	-1.8002
30	H	-1.1934	-3.1932	-0.0804
31	H	3.2033	2.4903	-1.0970
32	H	1.6193	2.2319	-1.8700
33	H	1.7192	3.0867	-0.3114
34	H	3.6722	1.4160	1.1571
35	H	2.0716	1.8105	1.8300
36	H	2.6282	0.1234	1.7913
37	H	-1.9662	1.1382	-3.1331
38	H	-3.4447	1.4079	-2.0141
39	O	-3.7575	1.1550	1.7164
40	H	-4.6029	0.7362	1.9298

Charge = 0, Multiplicity = 2, E_{el} = -734.86565046 Ha

Table S119 (Mol 13 (LON), RAF adduct radical (PostRC), site 0 (M06-2X/ma-def2-SVP)) – Continued

#	Atom	X (Å)	Y (Å)	Z (Å)

$E_{\text{ZPE}} = 0.35325506$ Ha

10 lowest real freq. (cm^{-1}): 52.3, 96.0, 100.8, 121.5, 149.2, 155.9, 194.6, 203.5, 218.8, 229.9

Table S120: Mol 13 (LON), RAF adduct radical (PostRC), site 12 (M06-2X/ma-def2-SVP)

#	Atom	X (Å)	Y (Å)	Z (Å)
1	O	-3.4983	0.3044	0.3478
2	C	-0.0899	-1.0998	-1.2315
3	C	0.7071	0.4896	0.0294
4	C	0.4563	-1.0336	0.2281
5	C	-0.2478	0.4659	-1.1957
6	C	2.1406	0.9476	-0.3055
7	C	1.0056	-1.5101	-2.2306
8	C	-0.5164	-1.3482	1.3063
9	C	2.6678	0.4002	-1.6518
10	C	-1.6661	0.9612	-1.0588
11	C	-1.3476	-1.9127	-1.4962
12	C	2.4490	-1.0888	-1.9252
13	C	-2.4436	0.8690	0.2257
14	C	2.1264	2.4795	-0.3932
15	C	3.0772	0.5283	0.8349
16	C	-2.3436	1.4524	-2.1026
17	H	0.3521	1.0938	0.8754
18	H	1.3687	-1.6403	0.3838
19	H	0.1595	0.9509	-2.0948
20	H	0.7141	-1.1195	-3.2225
21	H	0.9877	-2.6094	-2.3205
22	H	-0.4053	-0.8725	2.2828
23	H	-1.1489	-2.2355	1.2463
24	H	3.7490	0.6146	-1.6884

Table S120 (Mol 13 (LON), RAF adduct radical (PostRC), site 12 (M06-2X/ma-def2-SVP)) – Continued

#	Atom	X (Å)	Y (Å)	Z (Å)
25	H	2.2275	0.9807	-2.4808
26	H	2.8413	-1.6815	-1.0834
27	H	3.0651	-1.3762	-2.7913
28	H	-2.1917	-1.6128	-0.8604
29	H	-1.6646	-1.7878	-2.5439
30	H	-1.1516	-2.9838	-1.3291
31	H	3.1107	2.8633	-0.7040
32	H	1.3804	2.8262	-1.1258
33	H	1.8759	2.9276	0.5805
34	H	4.0686	0.9903	0.7087
35	H	2.6743	0.8523	1.8076
36	H	3.2205	-0.5613	0.8780
37	H	-1.8598	1.5758	-3.0743
38	H	-3.3920	1.7427	-2.0126
39	O	-1.8796	1.5481	1.2386
40	H	-2.4525	1.4331	2.0122

Charge = 0, Multiplicity = 2, E_{el} = -734.95570848 Ha

E_{ZPE} = 0.35209533 Ha

10 lowest real freq. (cm^{-1}): 57.3, 67.3, 102.9, 112.8, 157.3, 163.9, 173.8, 217.1, 220.6, 224.2

Table S121: Mol 13 (LON), RAF adduct radical (PostRC), site 15 (M06-2X/ma-def2-SVP)

#	Atom	X (Å)	Y (Å)	Z (Å)
1	O	-3.5078	0.0074	0.5253
2	C	-0.0041	-1.7657	-0.4151
3	C	0.4542	0.3036	0.1290
4	C	0.0273	-0.9645	0.9212
5	C	-0.2584	-0.3684	-1.0938
6	C	1.9254	0.7202	-0.0278
7	C	1.3417	-2.4063	-0.7963

Table S121 (Mol 13 (LON), RAF adduct radical (PostRC), site 15 (M06-2X/ma-def2-SVP)) – Continued

#	Atom	X (Å)	Y (Å)	Z (Å)
8	C	-1.3985	-0.7198	1.4247
9	C	2.7799	-0.2964	-0.8009
10	C	-1.6995	0.0019	-0.9841
11	C	-1.0750	-2.8389	-0.6073
12	C	2.6351	-1.7393	-0.3372
13	C	-2.3089	-0.1937	0.3146
14	C	1.9487	2.0459	-0.8013
15	C	2.5127	0.9584	1.3699
16	C	-2.4884	0.4368	-2.1780
17	H	-0.0747	1.2053	0.4836
18	H	0.6787	-1.3372	1.7261
19	H	0.1482	-0.2059	-2.1044
20	H	1.3605	-2.5089	-1.8965
21	H	1.3334	-3.4352	-0.4005
22	H	-1.4045	0.0205	2.2406
23	H	-1.8635	-1.6341	1.8257
24	H	3.8321	0.0226	-0.7219
25	H	2.5274	-0.2422	-1.8750
26	H	2.7214	-1.7957	0.7600
27	H	3.4735	-2.3342	-0.7302
28	H	-2.1038	-2.4863	-0.4614
29	H	-1.0099	-3.2508	-1.6270
30	H	-0.9065	-3.6697	0.0954
31	H	2.9848	2.3663	-0.9892
32	H	1.4450	1.9460	-1.7755
33	H	1.4427	2.8444	-0.2376
34	H	3.5185	1.3991	1.2933
35	H	1.8819	1.6558	1.9432
36	H	2.5965	0.0286	1.9507
37	H	-1.8249	0.9928	-2.8614

Table S121 (Mol 13 (LON), RAF adduct radical (PostRC), site 15 (M06-2X/ma-def2-SVP)) – Continued

#	Atom	X (Å)	Y (Å)	Z (Å)
38	H	-2.7770	-0.4896	-2.7286
39	O	-3.5991	1.2371	-1.9120
40	H	-4.0046	0.9189	-1.0896

Charge = 0, Multiplicity = 2, E_{el} = -734.98144941 Ha

E_{ZPE} = 0.35625564 Ha

10 lowest real freq. (cm^{-1}): 40.0, 77.5, 102.8, 108.8, 141.5, 188.7, 199.5, 223.0, 241.4, 252.9

11.7 HAT Transition State Geometries (M06-2X/def2-TZVP, Water SMD)

Transition state geometries for the HAT mechanism were taken from the NEB-CI converged structures at the "mid-method" level; single-point energies are from M06-2X/def2-TZVP with SMD water solvation.

Table S122: Mol 1 (CAM), HAT transition state, site 26 (M06-2X/ma-def2-SVP, SMD water)

#	Atom	X (Å)	Y (Å)	Z (Å)
1	O	3.0270	0.4582	0.2223
2	C	-0.0907	-1.0232	-0.4016
3	C	-1.2451	-0.1982	0.2366
4	C	0.7263	1.0792	-0.4710
5	C	0.4382	-0.0536	-1.4780
6	C	1.1141	-1.0902	0.5539
7	C	1.6652	0.3665	0.5341
8	C	-0.6329	1.2103	0.1872
9	C	-1.6107	-0.6146	1.6655
10	C	-2.5063	-0.3065	-0.6335
11	C	-1.2090	2.3310	0.6263
12	H	-0.4186	-2.0108	-0.7561
13	H	1.1463	2.0224	-0.8435

Table S122 (Mol 1 (CAM), HAT transition state, site 26 (M06-2X/ma-def2-SVP, SMD water)) – Continued

#	Atom	X (Å)	Y (Å)	Z (Å)
14	H	-0.3120	0.2344	-2.2253
15	H	1.3326	-0.4244	-1.9926
16	H	0.8666	-1.4410	1.5639
17	H	1.8865	-1.7613	0.1516
18	H	1.6000	0.8325	1.5340
19	H	-1.8250	-1.6945	1.7134
20	H	-0.8155	-0.3822	2.3866
21	H	-2.5142	-0.0797	1.9947
22	H	-3.2885	0.3766	-0.2710
23	H	-2.3101	-0.0607	-1.6853
24	H	-2.9019	-1.3339	-0.5900
25	H	-2.2084	2.3156	1.0691
26	H	-0.7116	3.2998	0.5491
27	H	3.2847	0.1804	-0.7641
28	O	3.5671	-0.7357	-1.6736
29	H	3.9457	-1.4963	-1.2028

Charge = 0, Multiplicity = 2, E_{el} = -541.59570171 Ha

E_{ZPE} = 0.24921467 Ha

10 lowest real freq. (cm^{-1}): 54.9, 87.6, 117.6, 163.3, 202.5, 228.2, 247.9, 257.7, 280.5, 311.1

Table S123: Mol 2 (IPO), HAT transition state, site 26 (M06-2X/ma-def2-SVP, SMD water)

#	Atom	X (Å)	Y (Å)	Z (Å)
1	O	-2.5177	0.6571	-0.6192
2	C	1.4173	-0.0259	-0.3816
3	C	0.9824	1.3199	0.2749
4	C	0.6404	-0.7024	0.8077
5	C	0.9395	0.5769	1.6315
6	C	-0.4407	1.6994	-0.1409

Table S123 (Mol 2 (IPO), HAT transition state, site 26 (M06-2X/ma-def2-SVP, SMD water)) – Continued

#	Atom	X (Å)	Y (Å)	Z (Å)
7	C	-0.8084	-0.7663	0.4152
8	C	-1.4662	0.6048	0.2866
9	C	2.9211	-0.2763	-0.2815
10	C	0.9605	-0.3220	-1.8047
11	C	-1.4452	-1.9056	0.1276
12	H	1.6672	2.1766	0.1624
13	H	1.0139	-1.6734	1.1677
14	H	0.1968	0.9022	2.3747
15	H	1.9204	0.5308	2.1172
16	H	-0.5108	1.8314	-1.2297
17	H	-0.7393	2.6530	0.3179
18	H	-1.8208	0.8831	1.2976
19	H	3.4638	0.3680	-0.9904
20	H	3.3253	-0.0881	0.7218
21	H	3.1433	-1.3226	-0.5428
22	H	1.1960	-1.3653	-2.0684
23	H	-0.1178	-0.1867	-1.9572
24	H	1.4937	0.3307	-2.5145
25	H	-2.4574	-1.9141	-0.2803
26	H	-0.9389	-2.8648	0.2592
27	H	-3.4535	0.2529	-0.2903
28	O	-4.3676	-0.5780	0.1035
29	H	-4.1974	-0.7950	1.0361

Charge = 0, Multiplicity = 2, E_{el} = -541.56376042 Ha

E_{ZPE} = 0.24805958 Ha

10 lowest real freq. (cm^{-1}): 68.7, 74.9, 138.8, 151.9, 185.4, 208.8, 237.6, 245.8, 303.2, 308.7

Table S124: Mol 4 (MYR), HAT transition state, site 26 (M06-2X/ma-def2-SVP, SMD water)

#	Atom	X (Å)	Y (Å)	Z (Å)
1	O	2.6995	-1.1569	0.1233
2	C	-1.1242	-0.5847	-0.1026
3	C	-1.4659	0.9152	-0.3610
4	C	0.0874	-0.3471	-1.0716
5	C	-0.7829	0.7716	-1.7394
6	C	-0.6008	1.8208	0.5240
7	C	1.2031	0.3946	-0.4149
8	C	-2.1517	-1.5305	-0.7245
9	C	-0.8402	-1.0414	1.3214
10	C	0.8453	1.4363	0.4407
11	C	2.6257	-0.0173	-0.6598
12	H	-2.5245	1.2126	-0.3334
13	H	0.4496	-1.1912	-1.6734
14	H	-0.2555	1.6478	-2.1453
15	H	-1.4423	0.3620	-2.5123
16	H	-0.9310	1.8060	1.5771
17	H	-0.6957	2.8743	0.2040
18	H	-2.3805	-1.3058	-1.7742
19	H	-1.7867	-2.5673	-0.6729
20	H	-3.0937	-1.4786	-0.1572
21	H	-0.2145	-0.3414	1.8855
22	H	-1.7871	-1.1853	1.8642
23	H	-0.3049	-2.0029	1.3033
24	H	1.6139	2.0254	0.9384
25	H	3.3254	0.7782	-0.3346
26	H	2.7942	-0.2385	-1.7295
27	H	2.5448	-0.7246	1.1807
28	O	2.2429	0.0717	2.1286
29	H	1.9502	-0.4030	2.9152

Table S124 (Mol 4 (MYR), HAT transition state, site 26 (M06-2X/ma-def2-SVP, SMD water)) – Continued

#	Atom	X (Å)	Y (Å)	Z (Å)
Charge = 0, Multiplicity = 2, E_{el} = -541.57991372 Ha				
E_{ZPE} = 0.25049149 Ha				
10 lowest real freq. (cm^{-1}): 107.3, 110.5, 147.6, 171.4, 195.5, 218.7, 232.2, 282.2, 295.7, 334.0				

Table S125: Mol 12 (ISO), HAT transition state, site 34 (M06-2X/ma-def2-SVP, SMD water)

#	Atom	X (Å)	Y (Å)	Z (Å)
1	O	3.5868	-0.3315	0.9453
2	C	0.8001	-0.8525	-0.1558
3	C	0.3951	0.6034	0.0745
4	C	-0.2585	-1.5297	-1.0567
5	C	2.1324	-0.9571	-0.9095
6	C	-2.0592	0.0754	-0.3693
7	C	-1.0346	0.7735	0.5493
8	C	-1.6883	-1.3991	-0.5484
9	C	3.2247	-0.0357	-0.3811
10	C	1.2618	1.6211	-0.0933
11	C	2.7173	1.4075	-0.4567
12	C	0.8708	-1.5869	1.1984
13	C	-3.4909	0.2802	0.1004
14	C	0.9100	3.0833	0.0403
15	C	-4.3159	1.2269	-0.7287
16	C	-4.0010	-0.3264	1.1768
17	H	0.0173	-2.5901	-1.1819
18	H	-0.2011	-1.0674	-2.0576
19	H	1.9729	-0.6784	-1.9649
20	H	2.4928	-1.9972	-0.8888
21	H	-1.9677	0.5539	-1.3609
22	H	-1.1266	0.3342	1.5588

Table S125 (Mol 12 (ISO), HAT transition state, site 34 (M06-2X/ma-def2-SVP, SMD water)) – Continued

#	Atom	X (Å)	Y (Å)	Z (Å)
23	H	-1.3029	1.8310	0.6618
24	H	-2.3838	-1.8731	-1.2581
25	H	-1.8114	-1.9335	0.4071
26	H	4.1194	-0.1276	-1.0226
27	H	2.8758	1.7491	-1.4958
28	H	3.3509	2.0584	0.1682
29	H	1.5600	-1.0702	1.8777
30	H	-0.1121	-1.6362	1.6844
31	H	1.2257	-2.6183	1.0457
32	H	-0.1683	3.2765	0.0460
33	H	1.3412	3.6486	-0.8018
34	H	1.3381	3.5141	0.9604
35	H	4.0589	-1.2841	0.9798
36	H	-3.8715	2.2357	-0.7212
37	H	-4.3391	0.8966	-1.7796
38	H	-5.3481	1.2975	-0.3615
39	H	-5.0365	-0.1471	1.4779
40	H	-3.4208	-1.0160	1.7957
41	O	4.0269	-2.5894	0.9873
42	H	3.6594	-2.8197	1.8581

Charge = 0, Multiplicity = 2, E_{el} = -736.92550274 Ha

E_{ZPE} = 0.36943754 Ha

10 lowest real freq. (cm^{-1}): 40.4, 54.5, 69.5, 71.8, 77.9, 107.8, 146.3, 154.3, 174.8, 188.5

11.8 HAT Pre-Reaction Complex (PRC) Geometries (M06-2X/ma-def2-SVP)

Pre-reaction complex geometries for the HAT mechanism, optimized at the M06-2X/ma-def2-SVP level.

Table S126: Mol 1 (CAM), HAT pre-reaction complex (PRC), site 26 (M06-2X/ma-def2-SVP)

#	Atom	X (Å)	Y (Å)	Z (Å)
1	O	3.0009	0.3577	0.2146
2	C	-0.0590	-1.2096	-0.4918
3	C	-1.2454	-0.3853	0.0913
4	C	0.7242	0.9053	-0.5751
5	C	0.5050	-0.2568	-1.5640
6	C	1.1200	-1.2311	0.4977
7	C	1.6301	0.2389	0.4973
8	C	-0.6577	1.0319	0.0251
9	C	-1.6373	-0.7798	1.5192
10	C	-2.4839	-0.5185	-0.8065
11	C	-1.2533	2.1551	0.4269
12	H	-0.3675	-2.2069	-0.8384
13	H	1.1482	1.8425	-0.9562
14	H	-0.1974	-0.0116	-2.3718
15	H	1.4379	-0.6282	-2.0178
16	H	0.8641	-1.6071	1.4963
17	H	1.9222	-1.8783	0.1083
18	H	1.4870	0.7289	1.4709
19	H	-1.8655	-1.8567	1.5760
20	H	-0.8465	-0.5505	2.2459
21	H	-2.5377	-0.2290	1.8305
22	H	-3.2746	0.1694	-0.4720
23	H	-2.2629	-0.2844	-1.8566
24	H	-2.8802	-1.5453	-0.7567

Table S126 (Mol 1 (CAM), HAT pre-reaction complex (PRC), site 26 (M06-2X/ma-def2-SVP)) – Continued

#	Atom	X (Å)	Y (Å)	Z (Å)
25	H	-2.2573	2.1465	0.8598
26	H	-0.7573	3.1238	0.3342
27	H	3.2046	-0.1020	-0.6105
28	O	3.7879	-1.1047	-2.3119
29	H	4.2049	-1.9871	-2.2858

Charge = 0, Multiplicity = 2, E_{el} = -540.99730409 Ha

E_{ZPE} = 0.25264472 Ha

10 lowest real freq. (cm^{-1}): 18.9, 31.8, 76.4, 117.6, 137.7, 176.5, 194.9, 201.9, 229.4, 247.7

Table S127: Mol 2 (IPO), HAT pre-reaction complex (PRC), site 26 (M06-2X/ma-def2-SVP)

#	Atom	X (Å)	Y (Å)	Z (Å)
1	O	-2.7343	0.7471	-0.7996
2	C	1.2281	-0.1578	-0.3423
3	C	0.7920	1.2022	0.2852
4	C	0.3030	-0.8102	0.7548
5	C	0.5857	0.4444	1.6213
6	C	-0.5658	1.6504	-0.2640
7	C	-1.0991	-0.7802	0.2142
8	C	-1.6627	0.6295	0.0930
9	C	2.7049	-0.4633	-0.0946
10	C	0.9092	-0.4293	-1.8066
11	C	-1.7630	-1.8723	-0.1949
12	H	1.5309	2.0180	0.2582
13	H	0.5901	-1.8041	1.1304
14	H	-0.2046	0.7964	2.2994
15	H	1.5123	0.3417	2.1956
16	H	-0.5294	1.7667	-1.3572
17	H	-0.8480	2.6296	0.1501

Table S127 (Mol 2 (IPO), HAT pre-reaction complex (PRC), site 26 (M06-2X/ma-def2-SVP)) – Continued

#	Atom	X (Å)	Y (Å)	Z (Å)
18	H	-2.0124	0.8823	1.1127
19	H	3.3321	0.1726	-0.7387
20	H	3.0146	-0.2934	0.9448
21	H	2.9245	-1.5127	-0.3449
22	H	1.1318	-1.4802	-2.0515
23	H	-0.1417	-0.2515	-2.0665
24	H	1.5360	0.2057	-2.4529
25	H	-2.7080	-1.7954	-0.7358
26	H	-1.3388	-2.8697	-0.0553
27	H	-3.4980	0.3133	-0.3962
28	O	-3.9047	-1.1122	1.1763
29	H	-3.3246	-1.3411	1.9249

Charge = 0, Multiplicity = 2, E_{el} = -540.97426383 Ha

E_{ZPE} = 0.25325813 Ha

10 lowest real freq. (cm^{-1}): 79.9, 111.0, 152.0, 173.3, 204.4, 211.6, 232.5, 240.7, 301.3, 316.2

Table S128: Mol 4 (MYR), HAT pre-reaction complex (PRC), site 26 (M06-2X/ma-def2-SVP)

#	Atom	X (Å)	Y (Å)	Z (Å)
1	O	2.8471	-1.4717	0.0983
2	C	-1.1748	-0.7322	0.2886
3	C	-1.5225	0.7603	-0.0142
4	C	0.0441	-0.5029	-0.6652
5	C	-0.8264	0.5688	-1.3836
6	C	-0.6706	1.7070	0.8450
7	C	1.1521	0.2592	0.0299
8	C	-2.1806	-1.7107	-0.3206
9	C	-0.9234	-1.1518	1.7335

Table S128 (Mol 4 (MYR), HAT pre-reaction complex (PRC), site 26 (M06-2X/ma-def2-SVP)) – Continued

#	Atom	X (Å)	Y (Å)	Z (Å)
10	C	0.7913	1.3289	0.7644
11	C	2.5759	-0.1364	-0.2535
12	H	-2.5852	1.0481	-0.0020
13	H	0.4235	-1.3696	-1.2256
14	H	-0.2915	1.4330	-1.8029
15	H	-1.4928	0.1492	-2.1449
16	H	-1.0191	1.7157	1.8928
17	H	-0.7958	2.7430	0.4855
18	H	-2.3657	-1.5342	-1.3874
19	H	-1.8111	-2.7425	-0.2130
20	H	-3.1455	-1.6426	0.2057
21	H	-0.4835	-0.3450	2.3334
22	H	-1.8679	-1.4344	2.2224
23	H	-0.2548	-2.0278	1.7642
24	H	1.5427	1.9611	1.2464
25	H	3.2645	0.5656	0.2479
26	H	2.7508	-0.0627	-1.3398
27	H	2.8012	-1.5124	1.0627
28	O	2.2104	-0.2159	2.5944
29	H	1.3186	-0.4539	2.9048

Charge = 0, Multiplicity = 2, E_{el} = -540.97459761 Ha

E_{ZPE} = 0.25399246 Ha

10 lowest real freq. (cm^{-1}): 49.6, 98.6, 102.2, 121.8, 183.3, 189.3, 211.9, 214.5, 236.6, 255.9

Table S129: Mol 12 (ISO), HAT pre-reaction complex (PRC), site 34 (M06-2X/ma-def2-SVP)

#	Atom	X (Å)	Y (Å)	Z (Å)
1	O	3.7019	-0.1523	0.9178

Table S129 (Mol 12 (ISO), HAT pre-reaction complex (PRC), site 34 (M06-2X/ma-def2-SVP)) – Continued

#	Atom	X (Å)	Y (Å)	Z (Å)
2	C	0.9436	-1.0465	-0.1002
3	C	0.4877	0.4068	0.0306
4	C	-0.1003	-1.8097	-0.9491
5	C	2.2957	-1.1597	-0.8242
6	C	-1.9501	-0.2136	-0.3567
7	C	-0.9380	0.5594	0.5156
8	C	-1.5353	-1.6795	-0.4479
9	C	3.3137	-0.0921	-0.4407
10	C	1.2874	1.4516	-0.2579
11	C	2.7260	1.2843	-0.6964
12	C	1.0352	-1.6952	1.2968
13	C	-3.3733	0.0141	0.1141
14	C	0.8828	2.9028	-0.1850
15	C	-3.8378	1.4491	0.1287
16	C	-4.1896	-0.9768	0.4911
17	H	0.1933	-2.8721	-1.0013
18	H	-0.0542	-1.4181	-1.9798
19	H	2.1290	-1.0648	-1.9106
20	H	2.7152	-2.1681	-0.6600
21	H	-1.8828	0.2192	-1.3746
22	H	-1.0212	0.1755	1.5488
23	H	-1.2171	1.6161	0.5643
24	H	-2.2139	-2.2220	-1.1246
25	H	-1.6407	-2.1520	0.5430
26	H	4.2083	-0.2164	-1.0795
27	H	2.7984	1.5213	-1.7731
28	H	3.3517	2.0291	-0.1780
29	H	1.7365	-1.1351	1.9300
30	H	0.0630	-1.7247	1.8069

Table S129 (Mol 12 (ISO), HAT pre-reaction complex (PRC), site 34 (M06-2X/ma-def2-SVP)) – Continued

#	Atom	X (Å)	Y (Å)	Z (Å)
31	H	1.3789	-2.7399	1.1891
32	H	-0.1983	3.0701	-0.1380
33	H	1.2652	3.4379	-1.0686
34	H	1.3425	3.3822	0.6952
35	H	3.9872	-1.0528	1.1191
36	H	-3.3680	2.0130	0.9494
37	H	-3.5668	1.9587	-0.8096
38	H	-4.9259	1.5143	0.2580
39	H	-5.2130	-0.7663	0.8100
40	H	-3.8784	-2.0220	0.4939
41	O	3.7384	-3.0304	1.6411
42	H	3.3737	-3.1286	2.5416

Charge = 0, Multiplicity = 2, E_{el} = -736.11943865 Ha

E_{ZPE} = 0.37363368 Ha

10 lowest real freq. (cm^{-1}): 38.6, 51.0, 58.3, 72.6, 85.2, 92.5, 116.5, 145.1, 152.1, 175.0

11.9 HAT Post-Reaction Complex (PostRC) Geometries (M06-2X/ma-def2-SVP)

Post-reaction complex geometries for the HAT mechanism, taken from the NEB-CI product endpoint at the M06-2X/ma-def2-SVP level.

Table S130: Mol 1 (CAM), HAT post-reaction complex (PostRC), site 26 (M06-2X/ma-def2-SVP)

#	Atom	X (Å)	Y (Å)	Z (Å)
1	O	3.2239	0.0230	0.2168
2	C	0.1477	-1.1685	-0.7318
3	C	-0.9614	-0.3254	-0.0379

Table S130 (Mol 1 (CAM), HAT post-reaction complex (PostRC), site 26 (M06-2X/ma-def2-SVP)) – Continued

#	Atom	X (Å)	Y (Å)	Z (Å)
4	C	0.3686	1.0964	-1.4112
5	C	0.5879	-0.2935	-1.9259
6	C	1.3682	-1.4977	0.1576
7	C	2.1276	-0.2844	0.6268
8	C	-0.4840	1.1082	-0.3032
9	C	-1.1741	-0.6490	1.4406
10	C	-2.2925	-0.5397	-0.7804
11	C	-0.8741	2.2127	0.4246
12	H	-0.2672	-2.1326	-1.0646
13	H	0.8032	1.9927	-1.8560
14	H	-0.0427	-0.4873	-2.8136
15	H	1.6242	-0.4975	-2.2403
16	H	1.0379	-2.0572	1.0476
17	H	2.0623	-2.1289	-0.4162
18	H	1.6449	0.3504	1.4036
19	H	-1.3797	-1.7227	1.5749
20	H	-0.3081	-0.3827	2.0629
21	H	-2.0428	-0.0997	1.8333
22	H	-3.0583	0.1520	-0.3987
23	H	-2.1864	-0.3584	-1.8597
24	H	-2.6537	-1.5701	-0.6357
25	H	-1.5447	2.1265	1.2804
26	H	-0.5167	3.2088	0.1606
27	H	3.8444	-0.9050	-1.3122
28	O	3.8148	-1.5002	-2.0802
29	H	4.7071	-1.5254	-2.4352

Charge = 0, Multiplicity = 2, E_{el} = -540.99929657 Ha

E_{ZPE} = 0.24921467 Ha

10 lowest real freq. (cm^{-1}): 54.9, 87.6, 117.6, 163.3, 202.5, 228.2, 247.9, 257.7, 280.5, 311.1

Table S130 (Mol 1 (CAM), HAT post-reaction complex (PostRC), site 26 (M06-2X/ma-def2-SVP)) – Continued

#	Atom	X (Å)	Y (Å)	Z (Å)

Table S131: Mol 2 (IPO), HAT post-reaction complex (PostRC), site 26 (M06-2X/ma-def2-SVP)

#	Atom	X (Å)	Y (Å)	Z (Å)
1	O	-3.0588	0.3407	-0.1638
2	C	0.9297	-0.1384	-0.4326
3	C	0.5203	1.1661	0.3171
4	C	0.3172	-0.8897	0.7998
5	C	0.6391	0.3736	1.6405
6	C	-0.9462	1.5114	0.0612
7	C	-1.1707	-1.0127	0.6172
8	C	-1.8767	0.3453	0.4985
9	C	2.4470	-0.3022	-0.5039
10	C	0.3340	-0.4177	-1.8065
11	C	-1.8318	-2.1692	0.5645
12	H	1.1671	2.0461	0.1743
13	H	0.7699	-1.8509	1.0855
14	H	-0.0496	0.6317	2.4596
15	H	1.6595	0.3592	2.0386
16	H	-1.1193	1.7115	-1.0064
17	H	-1.2305	2.4226	0.6072
18	H	-2.2165	0.5831	1.5415
19	H	2.8684	0.3938	-1.2461
20	H	2.9446	-0.1093	0.4559
21	H	2.7058	-1.3250	-0.8183
22	H	0.6230	-1.4255	-2.1432
23	H	-0.7632	-0.3778	-1.8249
24	H	0.7167	0.3055	-2.5445

Table S131 (Mol 2 (IPO), HAT post-reaction complex (PostRC), site 26 (M06-2X/ma-def2-SVP)) – Continued

#	Atom	X (Å)	Y (Å)	Z (Å)
25	H	-2.9123	-2.2205	0.4125
26	H	-1.2912	-3.1123	0.6716
27	H	-4.4608	-0.9822	-0.4615
28	O	-5.0482	-1.7511	-0.4358
29	H	-5.9246	-1.4183	-0.6460

Charge = 0, Multiplicity = 2, E_{el} = -540.98248759 Ha

E_{ZPE} = 0.24805958 Ha

10 lowest real freq. (cm^{-1}): 68.7, 74.9, 138.8, 151.9, 185.4, 208.8, 237.6, 245.8, 303.2, 308.7

Table S132: Mol 4 (MYR), HAT post-reaction complex (PostRC), site 26 (M06-2X/ma-def2-SVP)

#	Atom	X (Å)	Y (Å)	Z (Å)
1	O	1.9502	-1.1019	0.8119
2	C	-1.2443	-0.7881	0.1865
3	C	-1.4574	0.7460	-0.0207
4	C	0.0299	-0.6021	-0.6955
5	C	-0.6654	0.6185	-1.3456
6	C	-0.6242	1.5590	0.9741
7	C	1.1657	-0.0804	0.1557
8	C	-2.3114	-1.6093	-0.5362
9	C	-1.0804	-1.3271	1.6037
10	C	0.8016	1.0984	0.9636
11	C	2.5716	-0.3486	-0.2097
12	H	-2.4972	1.1043	-0.0664
13	H	0.3550	-1.4472	-1.3209
14	H	-0.0237	1.4673	-1.6279
15	H	-1.2914	0.3421	-2.2011
16	H	-1.0449	1.4734	1.9933

Table S132 (Mol 4 (MYR), HAT post-reaction complex (PostRC), site 26 (M06-2X/ma-def2-SVP)) – Continued

#	Atom	X (Å)	Y (Å)	Z (Å)
17	H	-0.6821	2.6321	0.7164
18	H	-2.4827	-1.2759	-1.5686
19	H	-2.0197	-2.6706	-0.5679
20	H	-3.2696	-1.5406	0.0019
21	H	-0.3315	-0.7897	2.2003
22	H	-2.0417	-1.2720	2.1396
23	H	-0.7765	-2.3848	1.5701
24	H	1.5411	1.5327	1.6378
25	H	3.3506	0.3429	0.1287
26	H	2.7893	-0.9032	-1.1289
27	H	2.1246	-0.7046	2.6313
28	O	2.0895	-0.2668	3.4982
29	H	2.3896	-0.9229	4.1326

Charge = 0, Multiplicity = 2, E_{el} = -540.98230219 Ha

E_{ZPE} = 0.25049149 Ha

10 lowest real freq. (cm^{-1}): 107.3, 110.5, 147.6, 171.4, 195.5, 218.7, 232.2, 282.2, 295.7, 334.0

Table S133: Mol 12 (ISO), HAT post-reaction complex (PostRC), site 34 (M06-2X/ma-def2-SVP)

#	Atom	X (Å)	Y (Å)	Z (Å)
1	O	3.6898	-1.1134	1.3388
2	C	0.9941	-1.2770	0.0237
3	C	0.7091	0.1868	0.3650
4	C	-0.0615	-1.7656	-0.9947
5	C	2.3651	-1.4459	-0.6423
6	C	-1.7398	-0.0249	-0.2683
7	C	-0.7226	0.4482	0.7760
8	C	-1.5069	-1.5112	-0.5778

Table S133 (Mol 12 (ISO), HAT post-reaction complex (PostRC), site 34 (M06-2X/ma-def2-SVP)) – Continued

#	Atom	X (Å)	Y (Å)	Z (Å)
9	C	3.4579	-0.6329	0.0771
10	C	1.6584	1.1439	0.3412
11	C	3.1118	0.8476	0.0391
12	C	0.9321	-2.1420	1.2965
13	C	-3.1836	0.2494	0.1035
14	C	1.4245	2.6045	0.6371
15	C	-4.2040	-0.0870	-0.9548
16	C	-3.5586	0.7534	1.2851
17	H	0.1008	-2.8400	-1.1825
18	H	0.1166	-1.2446	-1.9513
19	H	2.3281	-1.0585	-1.6729
20	H	2.6431	-2.5095	-0.6855
21	H	-1.5412	0.5355	-1.2036
22	H	-0.9179	-0.0929	1.7192
23	H	-0.8876	1.5087	0.9961
24	H	-2.1728	-1.8446	-1.3876
25	H	-1.7739	-2.1056	0.3127
26	H	4.4047	-0.8368	-0.4736
27	H	3.3722	1.2637	-0.9511
28	H	3.7472	1.3772	0.7684
29	H	1.6412	-1.7718	2.0534
30	H	-0.0670	-2.1359	1.7523
31	H	1.1982	-3.1836	1.0589
32	H	0.3747	2.9099	0.5786
33	H	1.9918	3.2226	-0.0763
34	H	1.8002	2.8590	1.6422
35	H	3.9324	-3.0318	1.2152
36	H	-3.9071	0.3260	-1.9320
37	H	-4.3020	-1.1763	-1.0818

Table S133 (Mol 12 (ISO), HAT post-reaction complex (PostRC), site 34 (M06-2X/ma-def2-SVP)) – Continued

#	Atom	X (Å)	Y (Å)	Z (Å)
38	H	-5.1932	0.3111	-0.6949
39	H	-4.6130	0.9373	1.5026
40	H	-2.8445	0.9995	2.0718
41	O	3.9118	-3.9229	0.8320
42	H	4.4106	-4.4812	1.4342

Charge = 0, Multiplicity = 2, E_{el} = -736.12746852 Ha

E_{ZPE} = 0.36943754 Ha

10 lowest real freq. (cm^{-1}): 40.4, 54.5, 69.5, 71.8, 77.9, 107.8, 146.3, 154.3, 174.8, 188.5

References

.
